# A Review of Ammonia (NH_3_) Adsorption in Porous Solids

**DOI:** 10.1021/acs.chemrev.6c00035

**Published:** 2026-08-17

**Authors:** Cristian Aristizabal-González, Dinesh Mullangi, Juan C. Muñoz-Senmache, Jesse L. Prelesnik, Prerna, Nayeon Kang, Arturo J. Hernández-Maldonado, J. Ilja Siepmann, Michael Tsapatsis

**Affiliations:** † Department of Chemical Engineering, 16146 University of Puerto Rico Mayagüez Campus, Mayagüez, Puerto Rico 00680, United States; ‡ Institute for NanoBioTechnology, 1466 Johns Hopkins University, 3400 N. Charles Street, Baltimore, Maryland 21218, United States; § Department of Chemistry and Chemical Theory Center, 5635 University of Minnesota, 207 Pleasant Street SE, Minneapolis Prerna, Minnesota 55455, United States; ∥ Department of Chemical Engineering and Materials Science, 5635 University of Minnesota, 421 Washington Avenue SE, Minneapolis, Minnesota 55455, United States; ⊥ Department of Chemical and Biomolecular Engineering, 1466 Johns Hopkins University, 3400 N. Charles Street, Baltimore, Maryland 21218, United States; ¶ Applied Physics Laboratory, 1466 Johns Hopkins University, 11100 Johns Hopkins Road, Laurel, Maryland 20723, United States

## Abstract

Ammonia (NH_3_) plays a crucial role in the agricultural sector as a fertilizer and is a dense hydrogen carrier that offers a potential renewable and carbon-free energy source. These significant advantages necessitate the development of efficient NH_3_ synthesis and separation systems. Conventionally, the Haber–Bosch (HB) process involves synthesis at high temperature (>673 K, 100–200 bar) and separation of NH_3_ from the equilibrium-limited reaction mixture through condensation at low temperatures. Among various alternative NH_3_ separation techniques, adsorption approaches in porous solids have been investigated as a potentially more energy-efficient method. However, identifying the optimal porous materials for reversible NH_3_ separation with high NH_3_ capacity remains challenging. This review discusses the potential of porous solid adsorbents, such as zeolites, metal–organic frameworks (MOFs), covalent organic frameworks (COFs), hydrogen-bonded organic frameworks (HOFs), porous organic polymers (POPs), carbons, and their composite materials with NH_3_ absorbents like ionic liquids and metal halides, for NH_3_ separation and other potential uses like storage and sensing. The review also discusses active sites for adsorption, and adsorbent structural and mechanical properties. It also emphasizes NH_3_ selectivity over N_2_ and H_2_, highlighting the solid sorbent’s reversibility, stability, and regeneration conditions. Future directions for NH_3_ adsorption research are highlighted.

## Graphical abstract

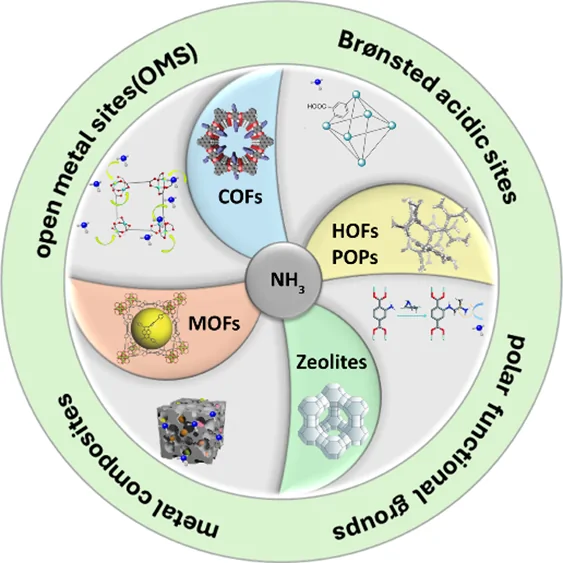

## 1 Introduction

Ammonia (NH_3_) plays a critical role as an agricultural fertilizer and serves as a feedstock in diverse industries, encompassing chemical, pharmaceutical, refrigeration, and water treatment applications.^1,2^ The substantial demand across diverse sectors has positioned NH_3_ as the second-largest chemical commodity globally, with over 200 million tons produced annually.^3^ The global demand for NH_3_ remains consistently high, driven primarily by its indispensable role in agriculture and its widespread industrial applications.^4^ Recent efforts toward green fuels consider NH_3_ as a versatile and eco-friendly resource due to its high hydrogen content and energy density (5.8 kWh/kg or 3.6 kWh/L in liquid form), positioning it as a promising clean energy carrier with zero carbon emissions after combustion in air (2NH_3_ + $\frac{3}{2}$ O_2_ → 3H_2_O + N_2_ + ΔH (where ΔH = −802.6 kJ/mol)).^5,6^ Considering a transition to a greener energy landscape, the demand for NH_3_ could triple by 2050, with a market cap of 224 billion dollars.^7^ Despite its current and potential applications, NH_3_ is extremely volatile and highly toxic under ambient conditions. It manifests as a pungent, corrosive gas that causes severe respiratory issues, temporary blindness, pulmonary edema, and even death with concentrations greater than 50 ppm and prolonged exposure.^8^ Furthermore, NH_3_ in the atmosphere reacts with NO_*x*_ and SO_*x*_, generating highly toxic PM2.5 particles.^9,10^ Therefore, NH_3_ production, transportation, and storage require methods for its safe handling, including purification, capture, and detection.

The global NH_3_ production mostly relies on the energy-intensive Haber–Bosch (HB) catalytic process, which contributes approximately 1–2% of total global CO_2_ emissions.^11^ The HB process has been a significant accomplishment utilized for over a century, enabling large quantities of NH_3_ production through the reaction of nitrogen (N_2_) and hydrogen (H_2_) at high pressure (100–200 bar) and temperature (>673 K/400 °C) in the presence of a catalyst (N_2_ + 3H_2_ ⇌ 2NH_3_ + 92.2 kJ/mol).^12,13^ Thermodynamic equilibrium at HB reaction conditions limits the NH_3_ synthesis reaction to low single-pass conversion (12–20%), requiring downstream separation of NH_3_ from the reactor effluent.^14^ In this context, porous materials have emerged as promising candidates for selective adsorption and separation of NH_3_, owing to their tunable pore structures and surface chemistry.^15,16^ This review provides a comprehensive overview of the experimental methodologies, structural characteristics, and NH_3_ adsorption properties of porous sorbents, including zeolites, metal–organic frameworks (MOFs), covalent organic frameworks (COFs), hydrogen-bonded organic frameworks (HOFs), porous organic polymers (POPs), carbon-based materials, and their composite materials. Given the extensive literature on zeolites and MOFs, these classes are discussed in greater detail, whereas COFs, HOFs, POPs, and other emerging materials are presented more concisely, as studies on NH_3_ adsorption in these systems are comparatively limited.

### 1.1 Overview of NH_3_ Separation Processes

Conventionally, the effluent stream of the ammonia synthesis reactor goes through a cryogenic condenser, typically operating between 240 and 253 K, depending on the composition of the reactor effluent gas. Condensation at these low temperatures^17^ represents a significant portion of the cost driver of the plant operation. Once liquefied, the ammonia enters a flash drum, and the remaining gaseous ammonia alongside unreacted nitrogen, hydrogen, and inert gas are recycled back to the reactor, forming a reaction–condensation synthesis loop. A certain portion of this recycle stream must be purged to prevent the accumulation of inert gas components. There are a variety of methods to recover ammonia and hydrogen from the purge gas stream, such as cryogenic separation, membrane filtration, pressure swing adsorption, or a metal hydride-based system.^18–20^ Following its production, the most common industrial method of storing and transporting ammonia is in refrigerated form, maintained at 243 K in insulated tanks under ambient pressure.^21^ Since this review focuses on ammonia separation by adsorption, it is essential to establish the importance of the separation process within the broader context of ammonia plant operations, which are sketched as a block flow diagram in Figure 1a, along with the corresponding temperature profile.

**Figure 1:**
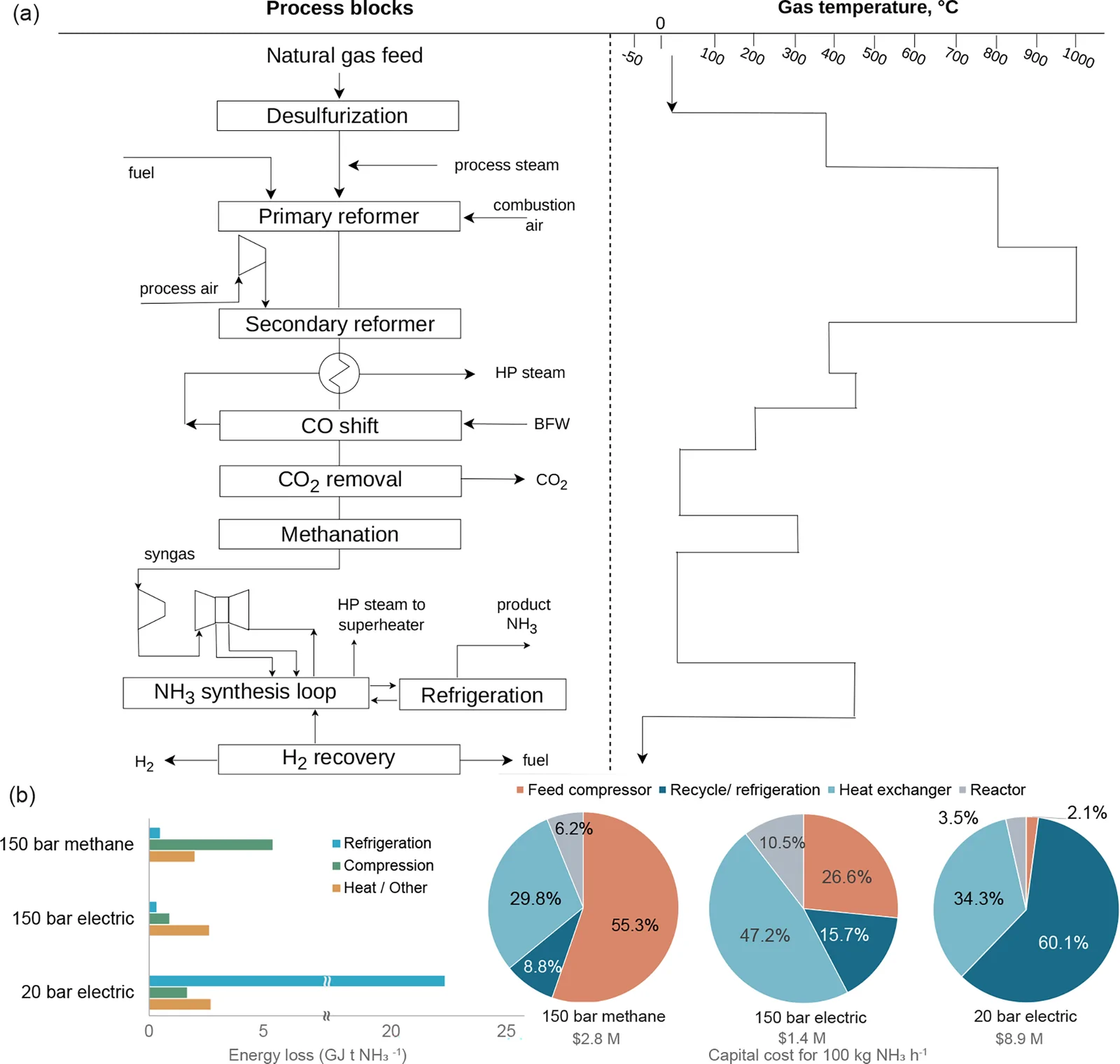
(a) Block flow diagram and corresponding gas temperature profile for a steam reforming ammonia plant. Adopted with permission from ref 34. Copyright 2011 Wiley-VCH. (b) Comparison of energy losses and capital cost requirements of different HB synthesis loop configurations for 100 kg NH_3_/h production. In each case, the reactor temperature is set as 673 K and the condenser at 248 K for 150 bar cases, and 240 K for 20 bar cases. Methane-fed configuration means the hydrogen is coming from the steam reforming process, while the electric process utilizes electrolysis for hydrogen production energy and cost estimates are from literature. Data source: refs 27, 34 .

The energy demand of the HB process is dominated by the energy needed for hydrogen production.^22^ Natural gas, via steam methane reforming (SMR), is the most common feedstock for hydrogen,^23^ which accounts for ∼80% of the total energy input.^24,25^ In contrast, the synthesis loop—primarily compression and refrigeration—contribute only ∼71% of the overall energy consumption.^26,27^ Despite this smaller share, the absolute energy use associated with ammonia separation remains substantial due to the large global production scale (about 170 million tons of ammonia per year^28^). For a representative energy intensity of 32 GJ/t_NH3_,^29–31^ the energy consumption in ammonia separation and refrigeration corresponds to on the order of 10^11^ kWh annually. Even modest improvements—for example, a 10% reduction in synthesis loop and refrigeration energy—would translate into savings of ∼11 TWh yr^–1^, comparable to annual electricity consumption of 1 million U.S. households^32^ or power output of four modern natrual gas power plants.^33^ Indeed, a technoeconomic analysis by Palys et al. demonstrated that opting for an absorptive removal of ammonia from the reaction mixture in place of refrigerative separation can lead to ∼ 25% capital cost reduction in small-scale (<100,000 t/y) ammonia plants.^35^ Hence, exploring and pursuing more energy-efficient ammonia separation alternatives is a high-impact priority for both research and industry.

Other strategies to reduce the energy intensity of the HB process have focused on low-pressure operation and the development of more active catalysts. Lowering the reactor pressure reduces the compression duty^36^ but practical benefits are limited: compressor power scales nonlinearly with pressure ratio and reduced single-pass conversion at low pressure increases recycle and refrigeration loads. Operation at 20–30 bar is therefore uneconomical, with capital costs roughly 20-fold higher than a conventional 150 bar synthesis loop (Figure 1b).^27^ Advanced Ammonia Process (KAAP), offer more than a 10-fold increase in activity than iron-based catalysts^37^ and can enable operation as low as near 90 bar.^38^ Nonetheless, the high cost has constrained deployment to less than 5% of global capacity. More broadly, the performance of transition-metal catalysts at mild conditions is limited by linear scaling relationship between nitrogen activation energy and surface intermediate bonding energy.^39^ While ongoing efforts aim to discover catalysts that break this scaling constraint, most remain at the computational screening stage with an uncertain commercialization timeline.^40^ As a result, recent progress in ammonia synthesis has largely relied on process-level optimization rather than transformative catalyst breakthroughs.^13,41–43^

#### 1.1.1 Different Processes for NH_3_ Separation

Commercial Haber–Bosch plants typically operate at 100–200 bar and 573–753 K. Leading proprietary processes include those of Braun, ICI, M.W. Kellogg, Haldor Topso̷e A/S, Uhde GmbH, and Exxon, with detailed comparisons shown in Rossetti’s work.^38^ Accordingly, evaluations of alternative postreactor ammonia separation technologies should reflect realistic effluent conditions within this pressure–temperature window. Reactor off-gas generally contains 10–15% NH_3_,^44^ which at 150 bar corresponds to an NH_3_ partial pressure of 15–22.5 bar. Sorption-based separation using metal halides or porous adsorbents has been proposed; however, most studies have been conducted near ambient temperature with ammonia pressure up to 1 bar, far from process-relevant conditions.^45–47^ Exceptions include studies by Aristizabal-González *et al*., reporting adsorption isotherms at up to 16 bar and 473 K,^48^ as well as work by Tokushige and Ryu,^49^ San *et al*.,^50^ and An *et al*.^51^

A major drawback of conventional condensation-based separation (Figure 2a) is its energy-consumption, as achieving the required ∼ 450 K temperature drop (Figure 1a), demands significant refrigeration duty.^52,53^ Alternative noncryogenic methods for separating ammonia from the reaction mixture include absorption-, adsorption-, and membrane-based separations (Figure 2b), along with process intensification strategies such as integrated reaction–separation systems (Figure 2c), where continuous removal of ammonia can help overcome thermodynamic equilibrium limitations^54^ and enhance overall reaction performance.^55^ Hybrid schemes combining cryogenic separation with adsorption also have been explored (Figure 2d).^56^ The following sections provide a brief discussion of three separation strategies: absorption, adsorption, and membrane separation, focusing on recent advances and remaining challenges.

**Figure 2:**
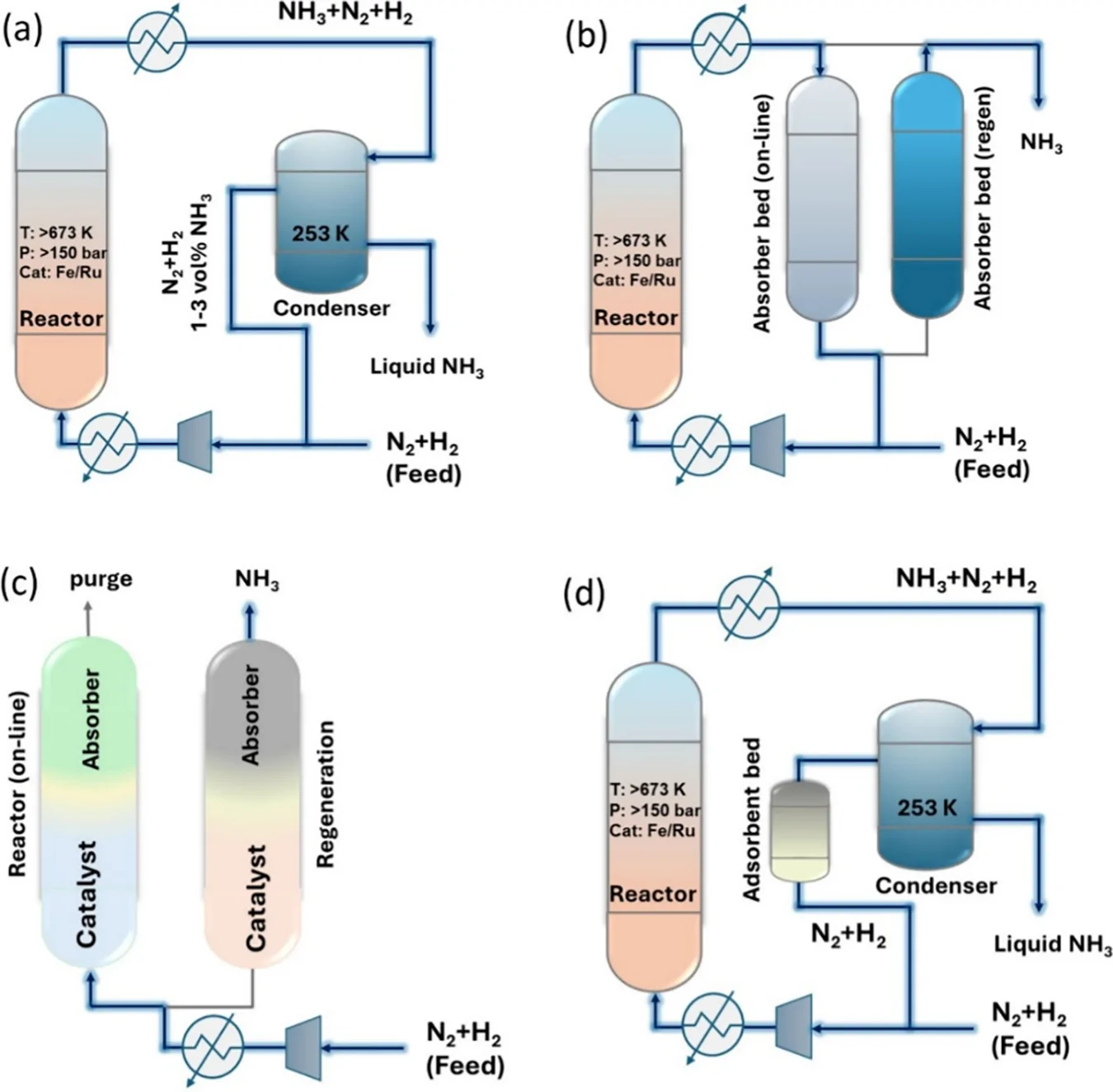
Schematic representations of the Haber–Bosch process for NH_3_ synthesis and separation processes: (a) Conventional condensation process for NH_3_ separation (NH_3_ liquification). (b) Metal halide-based absorption process or porous solid adsorbent-based pressure swing adsorption (PSA) or temperature swing adsorption (TSA) process (chemical or physical adsorption process), (c) In situ absorption and separation process, and (d) An example of a hybrid separation process consisting of condensation and subsequent physical adsorption.

#### 1.1.2 Absorbent-Based Separation

A clear distinction between the absorption and adsorption of NH_3_ in solids is vital. Absorption involves bulk uptake by chemical bonding within the solid, whereas adsorption is an interfacial phenomenon; depending on the interaction strength, adsorption may be classified as physisorption or chemisorption.^57^

Metal halide salts have attracted significant attention as ammonia absorbents due to their ability to form metal-ammine complexes.^58–64^ Various metal chlorides have been investigated,^42,59,65–70^ including their use in sorption-enhanced HB processes.^54,71,72^ Among these, MgCl_2_ and CaCl_2_ offer high NH_3_ capacities and high thermal stability,^73^ but practical deployment typically requires dispersion on porous supports such as silica or alumina with 30–40 wt % loadings.^11,74–76^ Beyond separation, in situ ammonia removal can shift thermodynamic equilibrium toward product formation, and alleviate ammonia inhibition on catalyst surface, thereby increasing single-pass conversion. MgCl_2_, CaCl_2_, and MnCl_2_ have been studied in this context (Table 1), with both experimental and modeling efforts demonstrating enhanced conversion.^13,54,77^

**Table 1:** Literature on Combined/Integrated Reaction**–**Separation Scheme for Ammonia Synthesis^a^

**Catalyst**	**Separation material**	**Configuration**	***T***_**rxn**_ **(K)**	***T***_**sep**_ **(K)**	***P***_**H2**_ **(bar)**	***P***_**N2**_ **(bar)**	**Performance (overall NH_3_ production rate)**	**Notes**	**Ref.**
Ru/CeO_2_ (lab-made)	Zeolite 4A	Physically mixed	473	473	0.05	0.75 (Ar 0.20)	Enhanced production rate from 128 to 566 μmol/g_cat_/h with in situ adsorption	Nonstochiometric feed, Zeolite 4A dried at 973 K under Ar	^80^
AmoMax-10 (Fe-based)	MgCl_2_	Separate beds	673	473	6	2	Improved N_2_ conversion from 20% to 95%, 22300 μmol/g_cat_/h^a^	Stoichiometric feed	^65^
AmoMax-10 (Fe-based)	CaCl_2_	Separate beds	673	453	15	5	6300 μmol/g_cat_/h	Stoichiometric feed	^42^
Cs/Ru/CeO_2_ (lab-made)	MnCl_2_/SiO_2_	Alternating layers in a PFR	613	613	6.7	13.3	H_2_ conversion 21.5%, 115 μmol/cm^3^/h^b^	Nonstochiometric feed, lab-made highly active catalyst	^54^
Ru on support	Zeolite 4A, 5A, 13X	Separate beds	n.d.	n.d.	7.5	2.5	7.34 mol/h (using 13X molecular sieve)	Stochiometric feed, commercial zeolite	^81^
Ru(10 wt %)/ Cs/MgO	sulfonated MPTMS membrane	Membrane reactor	573	573	1.5	0.5	Improved H_2_ conversion from 0.5% (packed-bed counterpart) to 9%	Stoichiometric feed, slow reaction kinetics at 573 K	^55^
Fe-based catalyst (commercial)	MnCl_2_/AC	Alternating layers in a PFR	603	603	60	20	Sorption enhancing effect (SEE) of 1.3	Stoichiometric feed, breakthrough analysis, theoretical modeling	^78^
**Reaction rates from ruthenium–based catalysts for comparison**									
RuCo DSAC^c^	-		473	-	7.5	2.5	1240 μmol/g_cat_/h	13.6 times higher rate than 5 wt % Cs–Ru/C at 473 K	^82^
Ba_2_RuH_6_/MgO	-		573	-	0.4	0.6	13800 μmol/g_cat_/h (9500 for stoichiometric feed)	Nonstoichiometric feed. One bar total pressure	^83^
Ru/La_0.5_Ce_0.5_O_1.75_	-		573		7.5	2.5	11000 μmol/g_cat_/h	Stoichiometric feed	^84^

a *T*_rxn_ and *T*_sep_ denote the temperature at which ammonia synthesis occurs, and at which ammonia is separated from the mixture, respectively.

b This value is from a data set with a different mass of absorbent than the reported conversion of 95%.

c From 1:1 N_2_:H_2_ feed at 583 K, normalized by the system volume.

d DSAC: dual single-atom catalysts.

Different process configurations have been explored. In systems with spatially separated reactors and absorbers,^13,70^ Himstedt *et al*.,^65^ and Malmali *et al*.,^69^ reported 80–95% N_2_ conversion at 55–80 bar usi ng MgCl_2_ or CaCl_2_ absorbents at ∼ 460–473 K. Integrated reactor-absorber designs have also been investigated, such as the MnCl_2_–Ru catalyst system proposed by Smith and Torrente-Murciano, which exceeded equilibrium conversion at 613 K and 20 bar.^54^ However, integration is challenging for conventional iron catalysts that require temperatures (>673 K) unfavorable for absorption.^44^ More recently, Cholewa *et al*. examined sorption-enhanced ammonia synthesis using Fe-based catalyst with MnCl_2_/AC, under an inlet pressure of 80 bar, reporting a 1.3-fold increase in the effective reaction rate.^78^

Some optimization studies continue to use separate temperatures for the reaction and sorption steps, typically around 673 K for the reaction using a commercial iron catalyst, and 453–473 K for absorption.^65,70^ In contrast, other efforts have attempted to integrate the reactor and absorber into a single vessel operating at uniform temperature and pressure.^54,79^ Cyclic and process-optimized configurations have further been examined. Ojha *et al*.^13^ reported ∼ 67% conversion in a semibatch setup using NiCl_2_, CaCl_2_, and MgCl_2_, while Nowrin *et al*.^70^ showed that cyclic reaction-absorption scheme improved production rates under continuous operation. Technoeconomic analysis by Lin *et al*.^85^ suggested that absorber-based configuration could reduce the ammonia synthesis loop energy consumption by nearly half in small-scale systems.

Despite these advantages, significant challenges remain. The primary limitation is the high energy required for regeneration, with desorption enthalpies of ∼ 70–100 kJ/mol range,^86,87^ leading to typical desorption temperatures near 673 K.^35,42,66,88^ In a number of studies, performance is evaluated primarily on uptake, and practical NH_3_ recovery and absorbent regeneration pathways are not fully addressed in these proof-of-concept studies.^65,68,69,88^ Regeneration is further complicated by discrete ammine stoichiometries^63,89^ and slow kinetics, which hinder complete NH_3_ release and reduce working capacity during cycling.^59,66,68,90–92^ For instance, silica-supported MgCl_2_ (33 wt %) exhibits only 50% of its initial capacity under 473 K absorption and 673 K desorption conditions.^88^ Additional constraints arise from process integration and material stability. Absorption at near ambient temperature improves capacity but requires significant cooling of reactor effluent, whereas higher temperature operation is more compatible with process conditions.^66^ Moisture sensitivity also necessitates rigorous pretreatment, as metal halides are highly hydroscopic and prone to decomposition^11,85,93^ and residual moisture can severely hinder catalyst performance.^44,80^

Mechanical and transport limitations further restrict the practical deployment. NH_3_ uptake induces substantial lattice expansion (up to 5–6 times for MgCl_2_, at ambient temperature) and particle agglomeration, reducing bulk density and thermal conductivity while compromising structural integrity.^94–96^ These effects are often overlooked in small-sample studies.^88,97^ Strategies such as supporting MgCl_2_ on thermally conductive substrates (e.g., aluminum fibers) have shown promise, improving bed thermal conductivity by 10 times with enhanced cyclic working capacity.^98^ Continued development of mechanically robust supports and materials with moderate binding strength will be essential for translating absorption-based systems beyond laboratory demonstration.

#### 1.1.3 Adsorbent-Based Separation

While interactions between adsorbents and ammonia are weaker than those of metal halide salts, porous materials such as zeolites and MOFs have been widely explored for ammonia-selective separation.^46,99^ These materials offer advantages in regeneration and cyclic operation, motivating their use in adsorption-based separation concepts. Several modeling and proof-of-concept studies have examined the integration of adsorption with that of ammonia synthesis. Petkovska *et al*. developed a MATLAB-based model using Cu-Y zeolite, predicting an increase in N_2_ conversion from 16% to 46% at 120 bar.^77^ Similarly, Monte Carlo simulations have identified narrow-pore zeolites, such as FER and MFI, as promising candidates for enhancing conversion via in situ ammonia removal.^100^ Experimental efforts remain limited; for example, Movick *et al*.^80^ demonstrated a hybrid system using zeolite 4A with a Ru/CeO_2_ catalyst at 473 K under low-pressure conditions, achieving high H_2_ conversion through periodic regeneration.

Adsorbents have also been investigated in pressure-swing adsorption (PSA) processes. SmartKoncept Technology demonstrated a four-column PSA system achieving ammonia purity and recovery of 99.6% and 99.7%, respectively, using a proprietary 12-step heavy reflux cycle.^101^ For successful integration of PSA separation into the HB reaction–separation loop as an alternative to conventional cryogenic separation, both purity and recovery performance metrics must meet acceptable thresholds. In related work, Bhadra demonstrated the inherent complexity of PSA cycles for ammonia separation, implementing a 16-step heavy reflux PSA cycle that achieved purity and recovery values exceeding 97% using zeolite 4A as the adsorbent.^102^ Although this study employed a realistic feed composition, the experimental conditions of 533 K and 20–30 bar total pressure remained far from typical reactor off-gas conditions. In contrast to studies assuming isothermal conditions, experiments conducted with a 50 cm length column revealed temperature variations up to 30 K between the top and bottom due to thermal wave propagation,^103^ highlighting the importance of accounting for thermal effects in PSA operation. Many modeling studies neglect adsorption kinetics and energy balances or rely on isotherms measured at mild conditions (<2 bar, near ambient temperature), limiting their relevance to industrial applications.^88,97,104^

At the material level, zeolites such as 4A, 5A, 13X, and Y have shown promising ammonia uptake and cycling stability.^81^ Tokushige and Ryu^49^ reported stable cyclic adsorption–desorption performance for Na-Y at 473 K with working capacity of 5 mmol/g (5↔1 bar swing) with a gravimetric measurement, while Aristizabal-Gonzalez *et al*.^48^ demonstrated working capacities up to 5.7 mmol/g (9.5↔0.001 mbar swing) and 1.60 mmol/g (11↔1 bar) using a volumetric measurement. Differences in experimental methodology and operating conditions, however, lead to significant variability in reported capacities.

Despite these advances, adsorption-based ammonia separation remains largely at the laboratory and modeling stage, with no demonstrated pilot-scale or industrial implementation under realistic Haber–Bosch conditions. From a process perspective, directly replacing conventional cryogenic condensation under typical operating conditions (*T* = 573–673 K, *P* ∼ 100 bar for reactor effluent) is challenging, as most adsorbents exhibit diminished NH_3_ uptake at elevated temperatures. These intrinsic material limitations are further compounded by process-level constraints, including increased mass transfer resistance at high pressure, heat effects arising from exothermic adsorption, and uncertainties associated with extrapolating adsorption data measured under mild conditions to industrial regimes. While adsorption may offer potential reduction in refrigeration and compressor duty, these benefits must be balanced against regeneration energy requirements, additional capital costs, and cycle complexity; comprehensive technoeconomic analysis under realistic conditions remain limited. Accordingly, current research has increasingly explored integrated approaches, such as sorption-enhanced reaction,^77^ and decoupled reactor-separator configurations^80^ to mitigate constraints imposed by individual material classes and leverage process and reactor design with the HB system.

#### 1.1.4 Membrane-Based Separation

Early work on ammonia separation using polymeric membranes dates back to Brubaker *et al*.[Bibr ref105] (1954), who used polyethylene at ∼ 306 K. While subsequent studies have reported high ammonia permeability and selectivity,^106^ performance typically deteriorates at elevated temperatures relevant to HB conditions (>573 K). For instance, Bhown^107^ observed an approximately 8-fold decrease in ammonia permeance when temperature increased from 294 to 383 K. To address the thermal limitations of polymeric membranes, other research has focused on inorganic alternatives such as zeolites, organosilica or ceramic, and molten salts, where separation arise from a combination of selective diffusion and competitive adsorption. LTA-type zeolitic imidazolate framework membranes have demonstrated ammonia permeance of 1727 GPU and selectivities of 35 and 12 for NH_3_/N_2_ and NH_3_/H_2_, respectively, at room temperature.^108^ MFI zeolite nanosheet membranes have shown separation factors up to 220 at 323 K and 7 bar,^109^ while simulations suggest performance can be retained at elevated conditions (523 K, 80 bar) with separation factors of 8.6 and 12.5 for NH_3_/N_2_ and NH_3_/H_2_ mixtures, respectively.^110^ LTA-type Na^+^-gated nanochannel membranes achieved significantly higher selectivities (NH_3_/N_2_ = 1106, NH_3_/H_2_ = 328) at temperatures as high as 473 K and pressures up to 35 bar.^111^

Organosilica membranes incorporating sulfonic acid functionalities further enhance the ammonia affinity.^53,112,113^ For instance, a 3-(trihydroxysilyl)-1-propanesulfonic acid (TPS)-based silica membrane exhibited an ammonia permeance of 765 GPU and NH_3_/N_2_ selectivity of 266 at 573 K.^112^ Alternative approaches using molten salt membranes, such as the LiNO_3_–ZnCl_2_ system, have demonstrated reversible NH_3_ absorption/desorption with permeance up to 700 GPU and with NH_3_/N_2_ and NH_3_/H_2_ selectivities both exceeding 1000 at 523–623 K.^114^ More recently, ZnCl_2_-based membranes achieved a permeance of 182 GPU at 573 K (single gas), and up to 1100 GPU was observed in a ternary mixture (NH_3_:H_2_:N_2_ = 11:67:23%) at 598 K, with a permeate ammonia purity reaching 99.9%.^115^ Membrane integration with reaction has also been explored. Tsuru *et al*. demonstrated a catalytic membrane reactor combining a Ru/Cs/MgO catalyst with a sulfonated silica membrane at 573 K and 2–3 bar, achieving a 10-fold increase in ammonia concentration in the permeate and improved nitrogen conversion (6–9%) relative to a packed-bed reactor.^55^

Although significant progress has been made in membrane materials in terms of selectivity and permeance, their performance under realistic process conditions (*T* > 573 K, *P* > 15 bar) remains uncertain. Issues related to thermal stability, pressure tolerance, and scalable implementation have yet to be resolved. At the same time, recent literature highlights multiple pathways to address these challenges. Rather than a single ideal material, different membrane classes offer distinct advantages, and effective implementation will likely require coordinated advances in both material design and reactor engineering to mitigate their respective limitations. For instance, emerging membrane reactor concepts —including operation under milder conditions, spatial or temporal decoupling of reaction and separation, and localized or electrified heating — may offer routes to reconcile the conflicting requirements of catalysts and membranes.^116^ Together, these developments highlight the need for integrated material-process design to enable practical membrane-based ammonia separation.

#### 1.1.5 Scope and Future Directions

So far, absorbents such as metal halides exhibit high NH_3_ absorption capacity. However, issues, including slow absorption kinetics, substantial volume expansion, and the need for high-temperature (453–603 K) desorption processes, hinder their industrial implementation.^11,42,117^ On the contrary, porous solid adsorbents such as zeolites, metal–organic frameworks, covalent organic frameworks, hydrogen-bonded organic frameworks, and porous organic polymers can capture gas molecules (NH_3_, H_2_, and N_2_) through weaker physical or chemical adsorption (Figure 3). This mode of binding offers advantages such as lower regeneration temperature and higher regeneration pressure in Temperature Swing Adsorption (TSA), Pressure Swing Adsorption (PSA), and temperature–pressure swing adsorption (TPSA) processes (Figure 2b), at the cost of reduced capacity and selectivity.^11,118–123^ Therefore, research is needed to deepen our understanding of how solid porous materials—such as zeolites, MOFs, COFs, HOFs, POPs, and composite absorbent-adsorbent materials—can be tailored to achieve high NH_3_ selectivity relevant to Haber-Bosch conditions. Efficient NH_3_ separation across a range of pressures and temperatures demands materials that not only exhibit superior selectivity for NH_3_ over N_2_ and H_2_, but also maintain structural integrity through repeated adsorption–desorption cycles, which are criteria met by only a limited subset of porous frameworks.^11,13,124^

**Figure 3:**
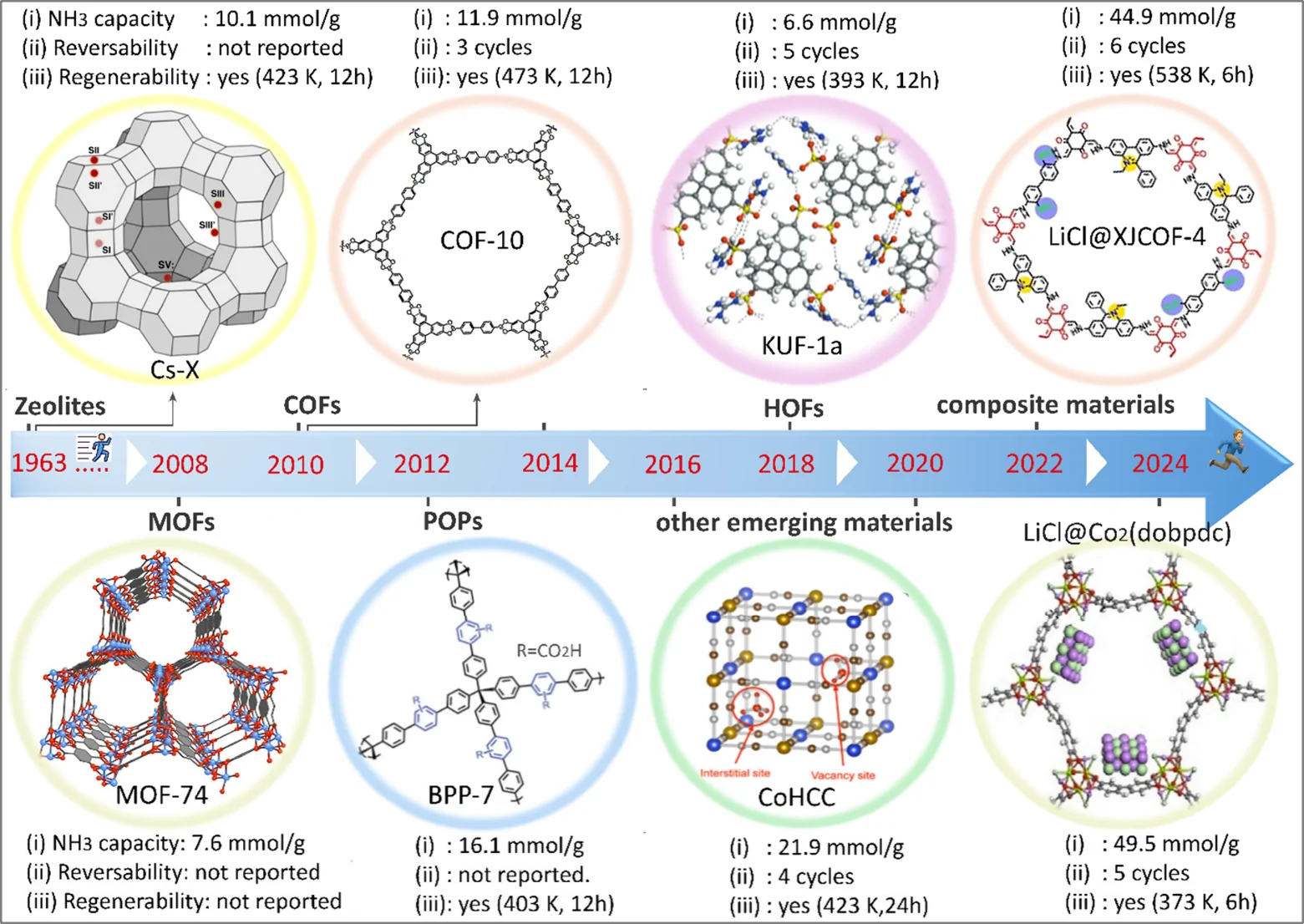
Timeline illustrates key developments in NH_3_ adsorption in porous solids. The figure was constructed using data from the literature.^125–132^ Images are adapted with permission from refs 126–132 . Copyright 2019 National Academy of Sciences.^126^ Copyright 2010 Springer Nature.^127^ Copyright 2017 Royal Society of Chemistry.^128^ Copyright 2019 Wiley-VCH.^129^ Copyright 2016 American Chemical Society.^130^ Copyright 2023 Wiley-VCH.^131^ Copyright 2024 Elsevier.^132^

### 1.2 Benchmarks for NH_3_ Adsorption Performance

A timeline of studies of ammonia adsorption in porous solids is shown in Figure 3, including a brief reference to important properties used to assess the potential of these materials for applications. While many studies focus on one or two of these properties (e.g., adsorption capacity and reversibility), it is the combination of all these properties, considered in the context of specific process conditions, that are important for evaluating the practical use of these materials.

#### 1.2.1 Adsorption Capacity

The adsorbed amount per unit mass of the adsorbent (mmol/g) under specific temperature and pressure conditions (e.g., 298 K, 1 bar) varies widely among different sorbents. It is usually assumed to represent the equilibrium amount adsorbed that corresponds to a certain adsorbate pressure; however, kinetic limitations often prohibit the determination of the equilibrium amount. Although the equilibrium adsorbed amount is fundamentally important, kinetically determined adsorbed amounts are more relevant for practical use.

#### 1.2.2 Reversibility

Except for use in guard beds or other applications that do not require adsorbate recovery and adsorbent bed cycling, the desorption isotherm is equally important as determining the adsorption isotherm. Characteristics like hysteresis between adsorption and desorption isotherms and irreversibly bound adsorbates that cannot be removed by pressure decrease alone (i.e., their removal requires higher temperature, often prolonged activation) are of fundamental significance. They reveal kinetic and thermodynamic limitations and possible adsorbate-induced structural changes of the adsorbent. They are also necessary to define the usable (working) capacity of an adsorbent in a cyclic pressure-swing adsorption (PSA) and temperature-swing adsorption (TSA) process that operates under certain time constraints. This working capacity of the adsorbent is an important metric for the economic assessment of the PSA/TSA separation processes. In this Review, we provide both adsorption and desorption isotherms whenever available, and we provide details on regeneration conditions in the text, figure captions, and Table 4. Adsorption isotherms are provided as filled symbols, and desorption isotherms are provided as open symbols. The adsorption isotherms extracted from the respective literature sources have been converted into adsorption information files (AIFs), which are freely available online at 10.34863/jdke-fe61.

#### 1.2.3 Regeneration Conditions

In an ideal adsorbent, regeneration may be accomplished by reducing the adsorbate pressure isothermally. However, along with pressure reduction, prolonged heating at temperatures much higher than the adsorption temperature is often required, rendering regeneration impractical for a cyclic separation process.

#### 1.2.4 Selectivity (NH_3_ vs N_2_ and H_2_)

Mixed gas adsorption isotherms or other data to experimentally determine selectivity under process conditions are seldom available. Therefore, ideal adsorbed solution theory (IAST) or other models are used for selectivity calculations from single-component isotherms.^133^

#### 1.2.5 Process Considerations

Although the above properties are important in qualitatively assessing adsorbent potential, a complete picture that fully assesses its potential in a real industrial process must include process design and optimization, process scale assessment, and techno-economic analysis. For example, it is important to consider that after its production, the most common industrial method of storing and transporting ammonia is in refrigerated form; therefore, comparisons of adsorption vs condensation processes could be biased toward adsorption if the need for condensation is neglected. It is also important to consider that adsorption beds contain significant bed porosity, which at high operating pressures during the adsorption cycle of the bed is occupied by mixtures (NH_3_, N_2_, and H_2_) enriched in the less adsorbing gases, complicating the process for recovering high-purity adsorbate during the desorption cycle.

## 2 Zeolites, Aluminophosphates, and Carbons

Zeolites are porous crystalline materials composed of tetrahedra (SiO_4_ and/or AlO_4_) arranged in a three-dimensional framework with interconnected channels. The term “zeolite”, coined in 1756 by the mineralogist Axel Fredrik Cronstedt, derives from Greek words meaning “boiling stone,” referencing their ability to release adsorbed water upon heating.^134^ Due to their thermal and chemical stability, well-defined pore structure, and tunable (e.g., by ion exchange) properties, zeolites have found widespread use in catalysis and separations.^135,136^

Lin, Hsieh, and Malmali,^104^ based on mathematical modeling using pure component NH_3_ and N_2_ equilibrium adsorption isotherm data for LTA and Faujasite (FAU) zeolites proposed pressure swing adsorption (PSA) to recover NH_3_ at 298 K.^104^ The high-pressure adsorption amounts for both gases were estimated via extrapolation of low-pressure adsorption data and Freundlich isotherm models, and the PSA modeling predicted a recovery of up to 95% NH_3_ for a setup of beds in rapid adsorption and desorption cycles (2–14 s) at 298 K. For studies like the one cited above and for integration of reaction and separation, either through membranes or adsorption, there is a need for experimental measurements of adsorption data at higher temperatures and pressures. Molecular simulations could also be utilized to predict adsorption behavior under high temperature and pressure conditions that pose challenges for experimentation.

Many adsorption processes rely on surface potentials to attract targeted adsorbate molecules based on differences in physicochemical properties. Among the porous materials tested for NH_3_ adsorption, those with microporous frameworks and narrow pore dimensions appear to facilitate higher selectivities. The kinetic diameters commonly used to infer gas sorption and transport in porous materials are 2.60, 2.89, and 3.64 Å for NH_3_, H_2_, and N_2_, respectively.^137^ However, the very small kinetic diameter deduced for NH_3_ appears inconsistent with the size of its electron cloud.^138^ Among the three molecules, NH_3_ is the only one with a permanent dipole moment and the ability to act as both acceptor and donor for hydrogen bonds, whereas N_2_ and H_2_ possess only small quadrupole moments. Furthermore, NH_3_ has the largest polarizability, followed by N_2_, whereas that of H_2_ is very small. As a result, the guest–host interactions are much stronger for NH_3_, particularly for adsorbent materials with bond dipoles (e.g., the Si–O bond in zeolites), hydrogen-bonding functional groups (e.g., silanol nests in zeolites or carbonyl groups in carbonaceous materials), or ionic moieties. These stronger adsorbate–adsorbent interactions can yield high NH_3_ adsorption selectivity, while allowing the option to remain closer to the physisorption realm, which is critical for reversibility and multiple-cycle operations. A strong NH_3_ affinity has been reported for zeolites containing extra-framework sites based on transition metals. This usually involves a portion of NH_3_ that cannot be desorbed even after intense thermal or vacuum treatments or a combination of both.^139^ On the other hand, materials containing alkali or alkaline earth cations appear to offer a moderate affinity toward NH_3_ but, unlike transition metals, contribute more to reversible NH_3_ adsorption. Furthermore, reactions with acidic groups leading to the formation of NH_4_^+^ can also result in strong and irreversible adsorption of NH_3_.

The locations of adsorbed NH_3_ depend on the relative stabilities of each substitution site, as has been studied via simulation in titanium silicalite-1.^140^ Signorile *et al*. described the relative stability of Ti substitution sites via the chemical reaction silicalite-1 + TiO_2_(rutile) → titanium silicalite-1 + SiO_2_(α -quartz), with energies obtained via DFT. They reported that the relative stabilities of the Ti substitution sites vary by more than 30 kJ/mol, indicating that there is an energetic driving force for preferential Ti localization.^140^ Figure 4 shows the T-sites within silicalite-1 colored according to their substitution energies, illustrating how cage structures and site preferences align. Earlier studies in silicalite-1,^141^ using the same definition of the substitution energy, report similar findings, albeit with a spread closer to 10 kJ/mol across substitution sites. In agreement with Zhakisheva’s findings in MFI, Signorile *et al*. observe that the first ammonia binding enthalpy varies from −66 to −83 kJ/mol as a function of T-site position, and add important information that some T-sites (T9 in this case) can support two distinct NH_3_ binding events with similar enthalpies in the dilute loading limit, while others (T4) are unlikely to be doubly bound even near *P*_sat_, because a second binding event is 70 kJ/mol higher in energy than the first.

**Figure 4:**
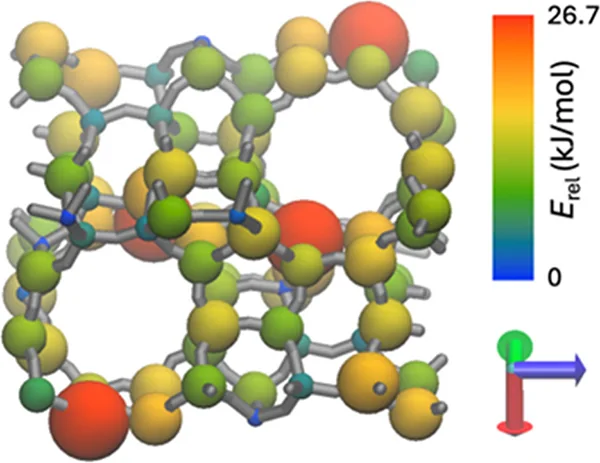
Silicalite-1 with the 24 unique Si sites colored according to the substitution energy corresponding to the hypothetical chemical reaction, silicalite-1 + TiO_2_(rutile) → titanium silicalite-1 + SiO_2_ (α-quartz), as calculated by DFT. Data source: ref 140.

Previous reviews on NH_3_ adsorption have compiled and discussed adsorption data for different classes of porous materials, reporting capacities over a range of temperatures and pressures, and examining the interaction mechanisms between NH_3_ and adsorbent frameworks. Motivated by the growing relevance of NH_3_ both as an energy carrier and as a hazardous pollutant, these studies have aimed to summarize the state of the art and identify material properties that are beneficial for NH_3_ capture and separation.^120,142,143^ For instance, Kang *et al*. reviewed the NH_3_ adsorption performance of emerging adsorbents under various operating conditions and discussed regeneration behavior as well as the role of active sites and framework chemistry in governing adsorption.^120^ Likewise, He *et al*. provided a broad conceptual overview of NH_3_ adsorption across several adsorbent families and summarized experimental results, mainly under near-ambient conditions. However, their analysis does not comprehensively cover the wide range of reported data available for conventional and emerging porous solids, including many studies on zeolites, MOFs, COFs, and carbons. Moreover, key comparative descriptors such as isosteric heats of adsorption, selectivity, regeneration performance, and relevant adsorbent properties are often only partially addressed, limiting a more systematic understanding of structure–property/performance relationships.^143^ Finally, Zheng *et al*. compared NH_3_ adsorbents primarily in the context of trace-level sensing applications, with emphasis on the advantages and disadvantages of emerging materials such as MOFs, COFs, HOFs, and POPs relative to more traditional adsorbents, including zeolites and carbons.^142^

Although these reviews provide valuable insights into NH_3_ adsorption, most remain focused on selected classes of materials, emerging adsorbents, or specific application windows such as near-ambient adsorption or trace-level sensing. As a result, the currently available literature lacks a truly comprehensive comparison spanning the full range of porous adsorbents and operating conditions reported to date. In addition, important metrics for practical evaluation, including isosteric heats of adsorption, selectivity, regeneration, and the influence of key material properties, are often not systematically compiled or discussed. Therefore, this portion of the Review presents a discussion about zeolites and carbons that have been tested so far for the uptake of NH_3_ at various temperatures (mainly at low pressures) and, from the adsorption and relevant material characterization data, attempts to establish guidelines about the potential use of these materials for the separation of NH_3_ at conditions pertinent to the HB process. The benchmarking is mostly based on NH_3_ adsorption capacity and reversibility, selectivity, regeneration conditions, and heat of adsorption. However, it should be stressed that since the vast majority of the reports available in the literature for NH_3_ adsorption onto porous materials are for 1.0 bar of NH_3_ pressure or lower, there remains an opportunity to measure the adsorption of NH_3_ at higher pressures and also much higher temperatures to benchmark adsorbents for implementation in the HB process. An overview of computational data is also presented in tandem, providing consistent information about adsorption thermodynamics between experimental measurements and computational methodologies.

### 2.1 Adsorption at Ambient Temperature

Adsorbent materials with well-known structural stability at high temperature and pressure, such as zeolite-, zeolitic- (nonaluminosilicate-), and carbon-based adsorbents, have been chosen for discussion in this section. Further, most of the work on NH_3_ adsorption at ambient conditions reported in the literature is based on zeolites with a faujasite (FAU; types Y and X) topology, so a considerable portion of the discussion below will revolve around those observations. Table 2 presents a compilation of experimental and simulation data for the adsorbed amount of NH_3_ and the conditions under which the data were gathered. The adsorbed amounts shown here have been normalized by either unit cell or weight of adsorbent; however, if desired, capacities on a volumetric basis could be readily estimated using the reported framework densities also included in Table 2. In addition, some properties, including pore size, pore volume, framework density, and Si/Al ratio of the materials, have been included. Furthermore, the reports of adsorbed amounts measured via gravimetric methods are indicated by (*g*) near the material name, while the rest of them were measured via volumetric methods.

**Table 2:** Reported Amount of NH_3_ Adsorbed onto Selected Porous Zeolitic and Carbon Adsorbents^a^

									**Adsorbed amount**		
**Material**	**Pore Size (Å)**	**Pore Volume** (cm^3^/g)	**Framework Density** (g/cm^3^)	**Si/Al ratio**	**M** ^**n+**^ **(u.c.^–1^)**	***P*** **(bar)**	***T*** **(K)**	***Q*_st_** (kJ mol^–1^)	(mmol/g)	**molec (u.c.^–1^)**	**Ref.**
Na-X	7.4	0.22–0.35	n.a.	n.a.	n.a.	1.0	298	n.a.	9.4	n.a.	^45,127,152,153^
				n.a.	n.a.	1.0	298		9.3	n.a.	
							323		7.8	n.a.	
							343		6.8	n.a.	
							393		4.9	n.a.	
Na-X(g)	7.4	0.22–0.35	1.55	1.15	89.4	0.6	303	45–70	10.8	146.4	^125,149,152–154^
							333		9.5	127.7	
							363		8.8	119.3	
							393		7.7	103.5	
							423		6.7	90.0	
							453		4.9	66.8	
			1.54	1.23	86.0	0.4	423	n.a.	5.8	78.7	
			1.53	1.34	82.2	0.7	373	n.a.	9.0	119.7	
							393		8.2	108.9	
							413		7.4	98.8	
							433		6.4	85.9	
							453		5.7	76.7	
							473		5.0	66.6	
							493		4.2	55.5	
							513		3.4	45.7	
							533		2.7	36.6	
Na-X	7.4	0.46	1.54	1.26	85.0	1.0	323	19–33	12.6	168.5	^48^
							373		11.5	153.8	
							423		9.0	120.4	
							473		7.6	102.1	
						5.0	323		14.5	194.3	
							373		13.5	180.9	
							423		11.3	151.0	
							473		10.3	137.8	
						12.0	323		15.2	203.0	
							373		14.4	193.4	
							423		12.3	165.4	
							473		11.2	150.7	
						15.0	373		14.6	195.4	
							423		12.5	167.2	
							473		11.4	153.1	
Na-X	7.4	0.42	n.a.	n.a.	n.a.	3.0	298	n.a.	9.9	n.a.	^155^
							313		9.6	n.a.	
							333		9.1	n.a.	
							353		8.9	n.a.	
							373		7.3	n.a.	
K-X(g)	7.4	0.22	1.64	1.15	47.4	0.6	303	30–55	10.3	147.8	^149^
							333		9.3	132.6	
							363		7.8	111.3	
							393		6.7	95.1	
							423		5.9	84.0	
							453		4.8	68.8	
Rb-X(g)	7.4	0.22	1.89	1.15	47.4	0.6	303	40–55	11.5	188.9	^149^
							333		10.3	168.6	
							363		9.1	150.3	
							393		7.3	119.8	
							423		6.3	103.6	
							453		4.4	72.1	
Cs-X(g)	7.4	0.22	2.21	1.15	51.8	0.6	303	19–45	10.1	194.4	^149^
							333		9.0	174.2	
							363		7.7	148.9	
							393		6.2	120.5	
							423		4.9	94.2	
							453		3.4	65.8	
Cs-X(g)	7.4	0.22	2.13	1.34	46.9	0.7	373	50–52	5.6	104.1	^125^
							393		4.8	88.2	
							413		4.0	74.3	
							433		3.3	61.3	
							453		2.6	49.2	
							473		2.0	37.2	
							493		1.3	23.1	
							513		0.8	16.1	
							533		0.5	10.0	
Cr(III)-X(g)	7.4	0.22	1.53	1.23	8.0	0.4	423	62–67	5.4	71.2	^154^
Y(III)-X(g)	7.4	0.22	1.56	1.23	8.0	0.4	423	50–58	5.2	69.1	^154^
H-Y	7.4	0.28	1.34	2.8	37.2	0.7	323	n.a.	6.0	70.6	^46,152,156^
						0.4	373		3.6	42.9	
							473		1.6	19.0	
H-Y(g)	7.4	0.28	1.34	3.0	37.2	5	473	n.a.	3.1	36.2	^49^
Li-Y(g)	7.4	0.28	1.40	2.31	41.5	0.4	303	36–42	11.5	139.0	^139^
							473		3.8	46.3	
Na-Y	7.4	0.28	1.47	2.43	56.0	1.2	303	39–74	9.4	119.9	^46,156,157^
				2.8	50.5	0.7	323	n.a.	6.6	82.7	
						0.4	373		4.3	53.2	
							473		2.1	26.6	
Na-Y(g)	7.4	0.28	1.47	2.31	58.0	0.5	303	40–45	10.0	128.1	^49,139,154,158^
							473		3.7	48.1	
						0.4	423		4.7	59.7	
						0.5	333		8.7	111.7	
							363		7.4	94.4	
							393		6.1	79.0	
							423		5.4	69.3	
							453		4.6	59.7	
							483		4.0	51.0	
			1.45	3	48.0	5	473		5.1	64.4	
Na-Y	7.4	0.36	1.45	2.8	50.6	1.0	323	23–28	13.3	115.5	^48^
							373		11.3	98.4	
							423		9.0	78.5	
							473		7.9	68.9	
						5.0	323		15.5	135.0	
							373		14.0	121.6	
							423		12.2	105.6	
							473		11.0	95.5	
						12.0	323		16.3	142.0	
							373		14.7	128.1	
							423		13.3	115.3	
							473		12.2	106.4	
						15.0	373		15.0	130.6	
							423		13.4	116.3	
							473		12.5	108.7	
Br,Na-Y	7.4	0.28	n.a.	n.a.		1.2	303	28–75	8.9	n.a.	^157^
K-Y(g)	7.4	0.28	1.58	2.31	55.5	0.5	303	37–45	9.0	122.5	^139^
							473		2.7	55.5	
K-Y	7.4	0.28	1.50	2.8	47.2	0.7	323	37–45	6.5	87.2	^46,156^
						0.4	373		4.3	57.8	
							473		1.9	25.6	
Rb-Y	7.4	0.28	1.61	2.8	47.4	0.7	323		5.3	104.2	^46,156^
						0.4	373	n.a.	3.7	73.4	
							473		1.6	30.8	
Cs-Y	7.4	0.28	1.73	2.8	45.5	0.7	323		5.0	89.2	^46,156^
						0.4	373	n.a.	3.2	57.9	
							473		1.4	24.7	
Ag(I)-Y(g)	7.4	0.24	2.04	2.31	57.8	0.4	303	42–61	9.3	163.5	^139^
							333		8.1	143.3	
							363		7.2	127.9	
							393		6.4	113.5	
							423		5.8	102.9	
							448		4.9	85.6	
							473		4.6	80.8	
Ca(II)-Y(g)	7.4	0.28	1.46	2.31	25.5	0.5	303	42–65	12.2	153.6	^139,158^
							473		4.7	59.9	
					24.5	0.5	333		10.3	111.1	
							363		9.2	93.9	
							393		8.2	78.5	
							423		7.0	69.0	
							453		5.9	59.4	
							483		5.0	50.8	
Sr(II)-Y(g)	7.4	0.28	1.58	2.31	22.7	0.5	303	42–74	10.9	150.0	^139^
							473		4.9	64.4	
Ba(II)-Y(g)	7.4	0.28	1.70	2.31	21.3	0.5	303	41–71	9.5	141.6	^139^
							473		4.4	64.5	
Co(II)-Y	7.4	0.33	1.49	2.8	20.7	0.7	323	n.a.	9.4	123.0	^46,156^
				2.31	20.7	0.4	373		6.8	88.7	
							473		4.4	58.2	
Cu(II)-Y	7.4	0.33	1.50	2.31	23.1	0.7	323	n.a.	9,9	130.3	^46,156^
						0.4	373		7.8	102.7	
							473		4.4	58.0	
Mn(II)-Y(g)	7.4	0.28	1.49	2.31	20.9	0.5	473	50–54	4.4	56.7	^139^
Cr(III)-Y(g)	7.4	0.22	1.46	2.31	4.8	0.4	423	59–63	4.9	62.7	^154^
Y(III)-Y(g)	7.4	0.22	1.48	2.31	4.1	0.4	423	59–63	4.7	60.6	^154^
Ti(III)-Y(g)	7.4	0.22	1.45	2.31	9.9	0.4	423	52–56	2.8	51.1	^154^
Fe(III)-Y(g)	7.4	0.22	1.46	2.31	6.8	0.4	423	53–74	5.2	66.4	^154^
La(III)-Y(g)	7.4	0.28	1.52	2.31	6.0	0.5	333	23–57	9.2	120.7	^158^
							363		8.4	110.1	
							393		7.2	94.6	
							423		6.0	79.2	
							453		5.1	66.5	
							483		4.4	57.9	
FAU(sim.)	7.4	0.36	1.33	Inf	-	1.0	298	26–35	2.3	26.9	^151^
						4.0	298		9.7	111.5	
						1.0	300		2.3	26.2	^100^
						10.0	300		4.2	48.9	
						1.0	298		1.5	17.1	^159^
						4.0	298		11.5	132.9	
						10.0	298		13.3	154.1	
Na-LTA	4.0	0.16	1.53	1.0	96.0	1.0	298	47–61	8.7	120.1	^45,160^
							323		7.9	107.7	
							343		7.0	96.3	
							393		5.0	66.7	
Na-LTA	4.0	0.25	1.53	1.0	96.0	1.0	323	27–29	13.2	118.1	^48^
							373		11.5	102.9	
							423		9.7	86.5	
							473		8.0	71.6	
						5.0	323		14.7	131.3	
							373		13.4	119.2	
							423		11.9	106.8	
							473		10.6	94.6	
						12.0	323		15.3	136.8	
							373		14.1	126.1	
							423		13.3	118.5	
							473		12.2	108.6	
						15.0	373		14.2	126.7	
							423		13.6	121.2	
							473		12.6	112.1	
Na-LTA(g)	4.0	0.16^§^	1.53	1.0	96.0	0.5	303	42–77	9.8	134.4	^49,150,154,160^
							423		5.3	73.4	
						0.4	423		5.2	69.6	
						5	473		4.4	60.0	
Na-LTA(sim.)	4	-	1.53	1.0	96.0	1.0	298	n.a.	9.3	107.1	^161^
							323		8.6	98.5	
							353		7.6	87.8	
							393		5.5	64.1	
							298	n.a.	8.6	122.6	^155^
							313		6.9	98.4	
							333		6.7	95.5	
							353		6.0	85.5	
							373		5.9	84.1	
LTA(sim.)	4.2	0.15	1.41	Inf	-	1.0	298	20–38	1.4	15.6	^100,151,159^
						4.0	298		8.8	101.3	
						1.0	300		1.4	15.7	
						10.0	300		10.2	117.6	
						1.0	298		1.2	14.3	
						4.0	298		8.9	102.0	
						10.0	298		10.6	122.3	
Ag(I)-LTA(g)	-	0.16^§^	2.44	1.0	95.8	0.5	303	59–69	7.2	155.7	^150,162^
							423		4.5	97.4	
Ca(II)-LTA	5.0	0.28	1.50	1.0	46.4	1.0	298	41–91	7.8	105.0	^150,162^
Co(II)-LTA(g)	--	-	1.60	1.0	44.8	0.5	303	44–67	8.7	123.6	^150^
							333		7.4	105.5	
							363		7.9	97.9	
							393		5.8	81.7	
							423		4.9	69.4	
							448		4.3	60.8	
							473		3.5	49.4	
Ni(II)-LTA(g)	-	-	1.57	1.0	32.8	0.5	303	48–85	9.3	130.6	^150^
							423		5.4	74.4	
Zn(II)-LTA(g)	-	0.30	1.63	1.0	48.0	0.3	303	56–94	10.4	151.4	^150,162^
							423		6.0	87.6	
Cd(II)-LTA(g)	-	-	1.88	1.0	47.2	0.5	303	45–85	9.0	151.3	^150^
							423		6.4	106.6	
Cr(III)-LTA(g)	-	-	1.53	1.0	7.2	0.4	423	45–46	5.2	69.3	^154^
Y(III)-LTA(g)	-	-	1.57	1.0	8.8	0.4	423		5.0	69.0	^154^
Clinoptilolite	4.0	-	1.75	5.0	8.0	1.0	298	n.a.	5.9	110.1	^163^
MFI	5.5*×*5.1	0.37	1.84	Inf	-	1.0	288	n.a.	1.0	13.5	^151,164–166^
				Inf	-	1.0	298		0.9	17.2	
				20	4.6	1.0	298	n.a.	3.0	17.9	
				32	2.9	1.0	303	30–91	3.9	22.8	
MFI(sim.)	5.5*×*5.1	0.055	1.80	Inf	-	1.0	298	21–32	1.8	10.3	^100,110,151,159^
						4.0	298		3.6	20.5	
						1.0	300		2.2	12.2	
						10.0	300		4.6	26.0	
						1.0	298		2.3	13.0	
						4.0	298		4.0	22.6	
						10.0	298		4.7	26.8	
						1.0	373		0.3	1.9	
						4.0	373		1.2	6.8	
H-MOR(g)	7	-	1.69	90.0	-	5	473	n.a.	0.8	2.3	^49^
Na-MOR(g)	7	-	1.76	9.0	-	5	473	n.a.	2.4	7.3	^49^
MOR(sim.)	7	0.073	1.69	Inf	-	1.0	300	n.a.	1.9	5.6	^100,159^
						10.0	300		5.8	16.7	
						1.0	298		2.1	5.9	
						4.0	298		5.5	15.9	
						10.0	298		6.9	20.0	
La-MOR	-	-	1.78	5.0	1.1	0.7	303	32–84	6.3	19.0	^167^
							333		5.3	16.0	
							363		4.8	14.5	
							393		4.1	12.5	
							423		3.6	11.0	
							448		3.2	9.7	
Ru-MOR	-	-	1.76	5.0	1.1	0.7	303	37–76	5.8	17.3	^167^
							333		5.0	14.9	
							363		4.5	13.3	
							393		3.8	11.4	
							423		3.4	10.2	
							448		2.9	8.8	
							473		2.6	7.9	
H–Al–Omega (MAZ)	-	0.12	1.66	4.5	6.5	0.5	323	50–72	6.7	14.5	^168^
							373		5.5	11.8	
							423		4.6	9.7	
							473		3.6	7.7	
							523		3.2	6.7	
Na–Al–Omega (MAZ)	7.5	0.12	1.77	4.5	6.5	0.5	323	40–46	6.1	14.2	^168^
							373		5.0	11.5	
							423		4.2	9.6	
							473		3.3	7.4	
							523		2.4	5.3	
La(III)-Al-Omega (MAZ)	-	0.12	1.84	4.5	1.3	0.5	323	53–66	6.0	14.4	^168^
							373		5.2	12.5	
							423		4.4	10.5	
							473		3.6	8.6	
							523		2.9	6.8	
H–Ga–Omega (MAZ)	-	0.12	1.88	4.5	6.6	0.5	323	48–59	5.9	14.5	^168^
							373		5.0	12.2	
							423		4.2	10.2	
							473		3.3	8.1	
							523		2.4	5.8	
Na–Ga–Omega (MAZ)	-	0.12	1.99	4.5	6.6	0.5	323	48–56	6.5	16.6	^168^
							373		5.6	14.3	
							423		4.4	11.2	
							473		3.4	8.6	
							523		2.4	6.0	
La(III)-Ga-Omega (MAZ)	-	0.12	2.06	4.5	1.4	0.5	323	48–62	6.2	16.1	^168^
							373		5.4	13.5	
							423		4.5	11.4	
							473		3.3	8.9	
							523		2.4	6.5	
AlPO_4_-11	6.6 × 4.4	0.23–0.25	1.95	-	-	0.7	333	29–31	7.9	19.3	^169,170^
							363		6.9	16.6	
							393		4.2	10.0	
							423		2.2	5.4	
							453		1.4	3.3	
							483		0.8	2.0	
						1.0	298	52	9.5	23.0	
CoAPO-11	6.6 × 4.4	0.23	1.99	-	1.4	0.7	333	29–32	6.1	15.0	^169^
							363		5.4	13.5	
							393		3.3	8.2	
							423		2.4	5.9	
							453		1.8	4.5	
							483		1.4	3.6	
MnAPO-11	6.6 × 4.4	0.23	1.97	-	0.6	0.7	333	29–32	5.1	12.5	^169^
							363		4.2	10.7	
							393		2.9	7.2	
							423		1.9	4.6	
							453		1.2	2.9	
							483		0.9	2.1	
SAPO-11	6.6 × 4.4	0.23	1.95	0.16	3.2	0.7	333	37–47	7.8	18.9	^169^
							363		6.3	15.4	
							393		4.7	11.6	
							423		2.7	6.6	
							453		2.0	4.7	
							483		1.4	3.4	
AlPO-5	7.3	0.38	1.71	-	-	1.0	298	n.a.	8.5	12.5	^170^
AlPO-17	5.1 × 3.6	0.37	1.63	-	-	1.0	298	n.a.	10.6	23.2	^170^
AlPO-18	3.8	0.58	1.52	-	-	1.0	298	87	13.2	38.6	^170^
AlPO-34	3.8	0.29	1.50	-	-	1.0	298	n.a.	13.2	28.9	^170^
H-ETS-10	8.0	0.21	1.91	-	-	1.0	283	n.a.	6.2		^171,172^
							293		5.6		
							303		4.7		
Na/K-ETS-10	8.0	0.21	1.91	-	-	1.0	283	n.a.	3.4		^171,172^
							293		3.0		
							303		2.2		
MCM-41	30	0.8–1.0	0.34^b^	-	-	1.0	298		7.8	-	^127^
SBA-15	100	1.25	0.1^b^	-	-	1.0	298	n.a.	6.5	-	^173^
Alumina silica gel	n.a.	0.42	n.a.	-	-	3.0	298	n.a.	10.1	-	^155^
							313		7.2	-	
							333		5.7	-	
							353		5.8	-	
							373		4.7	-	
Carbon molecular sieve	n.a.	0.28	0.3^b^	-	-	3.0	298	n.a.	9.8	-	^155^
							313		7.1	-	
							333		5.1	-	
							353		4.3	-	
							373		2.9	-	
Microporous and mesoporous activated carbon (7.7Å)	7.7	0.79	0.3^b^	-	-	0.9	298	25–27	16.3	-	^174^
						9.3	298		39.7	-	
Microporous activated carbon (20Å)	20	0.92	0.3^b^	-	-	0.7	298	n.a.	14.0	-	^174^
						9.4	298		31.3	-	
Microporous activated carbon (6.7Å)	6.7	0.68	0.3^b^	-	-	0.7	298	n.a.	14.3	-	^174^
						9.1	298		24.7	-	
Mesoporous activated carbon	32	1.2	0.3^b^	-	-	1.0	298	n.a.	10.0	-	^174^
						9.2	298		46.3	-	
Ordered mesoporous activated carbon	55,120	0.93	0.2^b^	-	-	1.0	298	n.a.	10.5	-	^174^
						9.5	298		52.5	-	
Ordered mesoporous carbon	85	0.68	0.2^b^	-	-	1.0	298	n.a.	7.5	-	^174^
						9.4	298		43.5	-	
Activated carbon	23	1.22	n.a.	-	-	3.0	298	n.a.	11.8	-	^175^
Mg(2)-AC	21	0.72	n.a.	-	-	3.0	298	n.a.	11.4	-	^175^
Mg(4)-AC	22	0.71	n.a.	-	-	3.0	293	n.a.	18.0	-	^175^
							298		16.6		
							313		16.5		
							333		12.4		
							353		9.4		
							373		8.0		
Mg(6)-AC	22	0.72	n.a.	-	-	3.0	298	n.a.	15.1	-	^175^
Mg(8)-AC	22	0.44	n.a.	-	-	3.0	298	n.a.	12.5	-	^175^
Mg(10)-AC	22	0.37	n.a.	-	-	3.0	298	n.a.	12.9	-	^175^
Activated carbon	n.a.	0.63	n.a.	-	-	1.0	278	26	11.9	-	^176^
							293		7.9	-	
							303		6.5	-	
							318		4.3	-	
AC-ZnSO_4_	n.a.	0.63	n.a.	-	-	1.0	278	28–38	12.9	-	^176^
							293		10.5	-	
							303		9.4	-	
							318		8.0	-	
Mg(0.3)-Z-13X	7.4	0.42	n.a.	n.a.	n.a.	3.0	298	n.a.	10.6	n.a.	^155^
							313		10.2		
							333		8.6		
							353		8.3		
							373		7.3		
Mg(0.5)-Z-13X	7.4	0.42	n.a.	n.a.	n.a.	3.0	298	n.a.	11.8	n.a.	^155^
							313		9.1		
							333		8.6		
							353		8.3		
							373		7.3		
Mg(0.75)-Z-13X	7.4	0.42	n.a.	n.a.	n.a.	3.0	298	n.a.	10.0	n.a.	^155^
							313		8.8		
							333		8.9		
							353		8.4		
							373		7.1		
Mg(1)-Z-13X	7.4	0.42	n.a.	n.a.	n.a.	3.0	298	n.a.	9.7	n.a.	^155^
							313		9.2		
							333		8.7		
							353		7.7		
							373		6.8		

a Some of the zeolite adsorbent properties, including framework densities, were obtained from crystallographic information from Baerlocher and McCusker, Database of Zeolite Structures: http://www.iza-structure.org/databases/.

b Average Bulk Density

Generally, at ambient temperature and low pressure of NH_3_, the adsorbed amounts on microporous materials are superior to those of mesoporous materials, including carbons (Figure 5). The adsorbate–adsorbent interactions are stronger in narrow pore architectures, and the mesoporous materials reported do not have specific adsorption sites (e.g., cations). Another general trend that can be observed from Figure 5 is the influence of the Si/Al ratio in zeolites, which is related to the number of cations that counterbalance the framework charge. The affinity toward NH_3_ is inversely proportional to the ratio, evidencing the importance of the NH_3_–cation interactions. Kanazirev and Borisova made a similar observation when evaluating the affinity of faujasite zeolites, with Si/Al ratios between 1.0 and 3.1.^144^ Interestingly, aluminophosphate and silicoaluminophosphate materials, where some of the tetrahedral sites are occupied by phosphorus atoms, show the largest adsorbed amount, suggesting that adsorbate–cation interactions are not the sole driving force for the higher adsorption. For aluminosilicates and silicoaluminophosphates, when extraframework cations are present, steric considerations must also be considered, given that not all cations are located in sites that would be geometrically favorable for NH_3_ adsorption.

**Figure 5:**
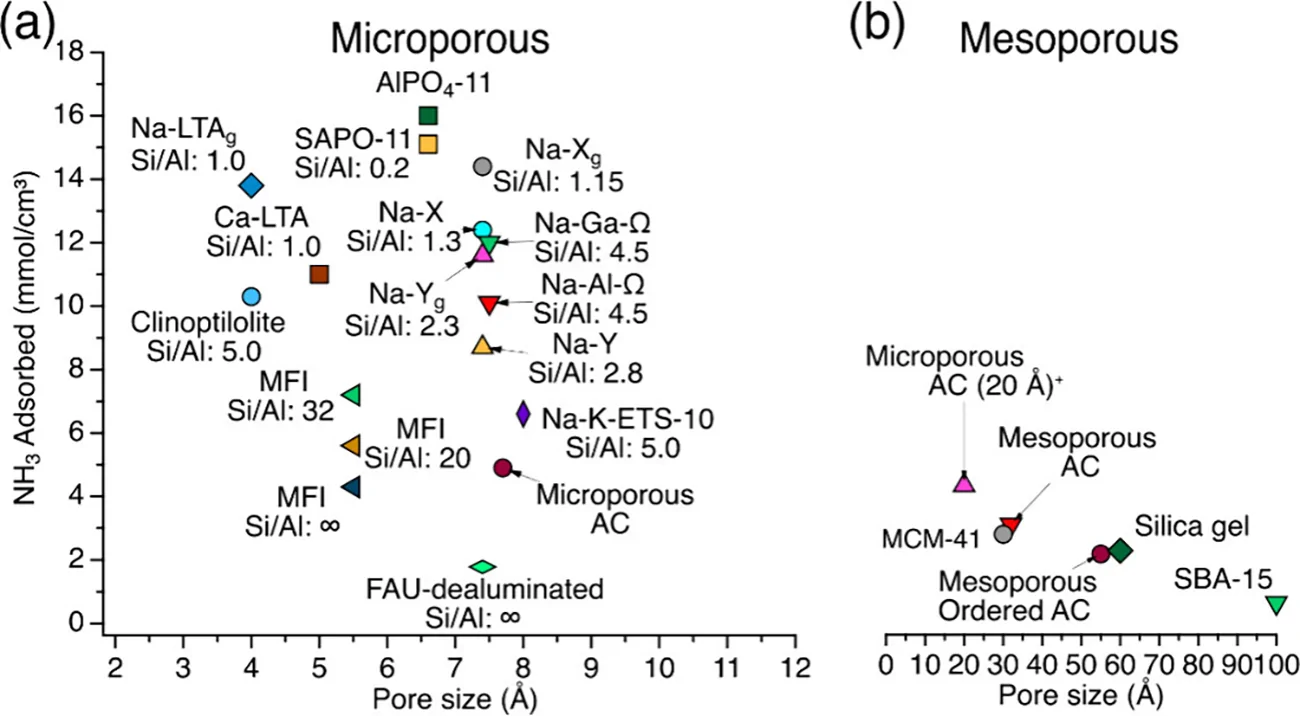
NH_3_ equilibrium adsorbed amounts reported at ambient temperature as a function of the pore size of selected materials. Data for adsorption at 1 bar partial pressure of NH_3_. (a) microporous materials with <10 Å pore sizes, (b) micro- and mesoporous materials with 20–100 Å pore sizes. Subscript g refers to gravimetric equilibrium adsorption methodology; the rest of the data were gathered using volumetric equilibrium adsorption. Only the larger pore width was considered for materials with a nonsymmetrical pore geometry (e.g., elliptical). Data sources are included in Table 2.

#### 2.1.1 Aluminosilicates

##### 2.1.1.1 Faujasite (FAU)

A comparison of NH_3_ adsorbed amounts for Na^+^-containing FAU types (i.e., Na-X and Na-Y) clearly shows that the adsorbate has a stronger affinity toward Na-X (Table 2).^145^ A greater Na^+^ content also results in a higher probability of having the extra-framework cations located in positions that would be sterically accessible to NH_3_. However, it has been reported via neutron diffraction that NH_3_ not only interacts with the counterions but also with the framework oxygen atoms via the formation of weak hydrogen bonds.^146^ Framework charge distribution, estimated by the Sanderson equalization principle, can also be used to quantify the affinity of FAU X toward NH_3_. Gilles *et al*. have correlated the negative charge of the oxygen atoms from FAU structures with the interaction strength toward NH_3_ via Fourier transform infrared (FTIR)^147^ and NMR^148^ spectroscopy. The FT-IR study results showed that the larger (the magnitude of) the negative charge, the higher the shift of the vibrational bands of adsorbed NH_3_ compared with gaseous NH_3_, indicating a stronger interaction.

Further supporting this conclusion are ^1^H NMR analyses, which showed that the transition temperature between the rotational and isotropic motions of NH_3_ adsorbed was 208 K for Na-X and 179 K for Na-Y.^148^ This can also be related to the strength of interaction between the adsorbent and NH_3_; the higher the transition temperature, the stronger the interaction. Reported isosteric heat of adsorption data show stronger interactions for Na-X than Na-Y at low NH_3_ adsorption loadings.^149,150^ Simulation results with all-silica FAU frameworks show correspondingly underpredicted values of the isosteric heat of adsorption due to the absence of strong adsorption sites, neither from counterions nor defects in a synthesized material.^151^ Relatedly, simulated isotherms are typically fully reversible in the absence of chemisorption sites, and all guest molecules can be removed at reduced pressures (Figure 6j). The presence or absence of Al substitutions has little impact on the accessible volume (Table 2); however, at high pressures, the NH_3_ occupancy of the all-silica zeolite becomes comparable to its Al-substituted variants. The absence of strong physisorption sites shifts the adsorption isotherm toward higher pressures, and so at ambient conditions (298 K and 1 bar), simulation loading of NH_3_ adsorption in FAU underpredicts experimental counterparts measured on Na-X/Y. At pressures closer to saturation (∼10 bar), the simulated loading within FAU is in closer agreement with experiment. There is remarkably good agreement between simulated adsorption isotherms from different research groups and force fields, as seen in Figure 6j. Due to inconsistencies between experimental isotherms at Si/Al ∞, where even low concentrations of silanol defects lead to a significant lowering of the onset pressure for the steep rise in the isotherm, only simulated isotherms are compared in Figure 6j.

**Figure 6:**
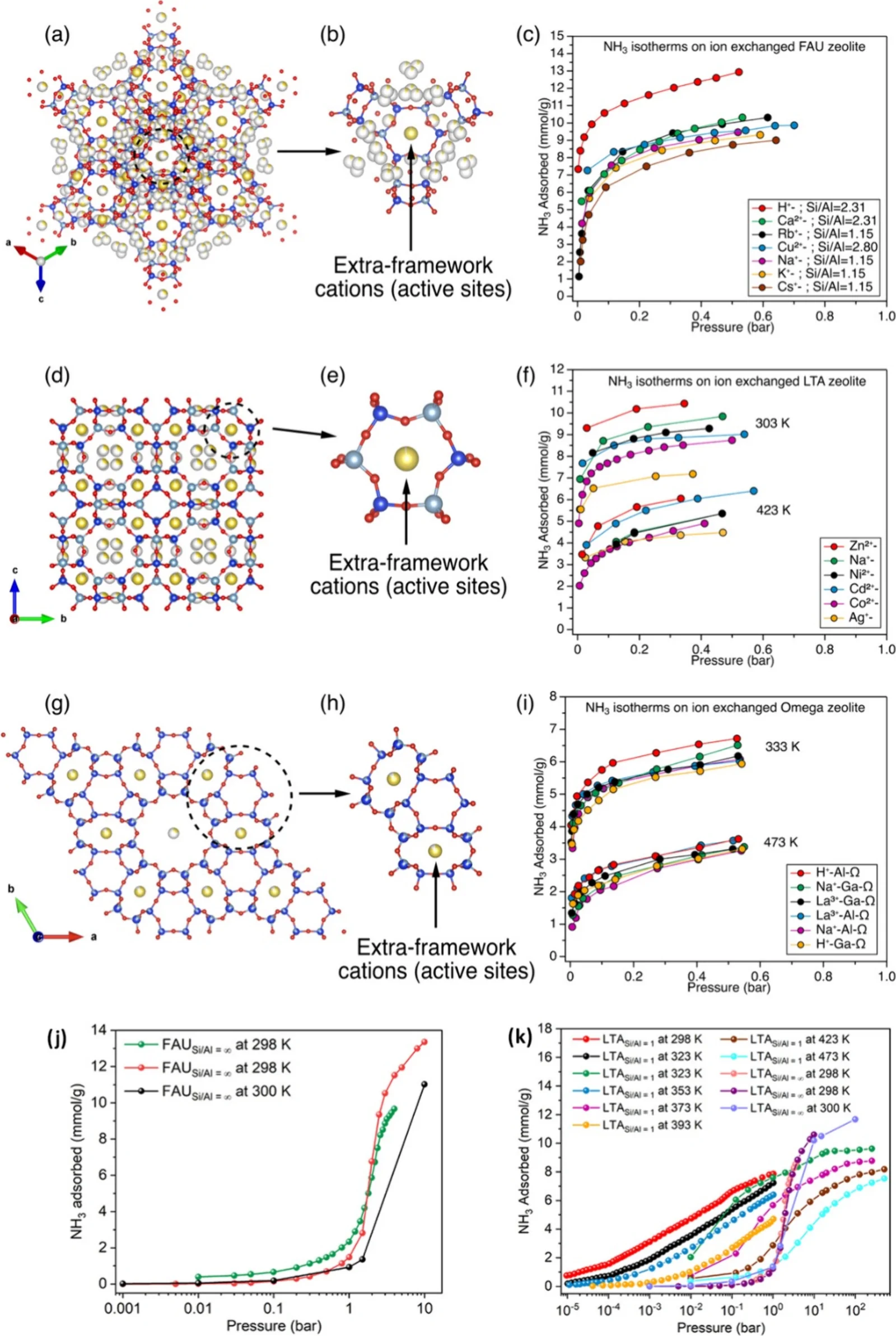
(a) Crystal structure of Na-X zeolite including cation locations. (b) Extra-framework cations are active sites for NH_3_ adsorption. (c) NH_3_ adsorption isotherms onto ion-exchanged FAU zeolite at 323 K with Si/Al ratios as indicated in the legend. (d) Crystal structure of Na-LTA zeolite, including cation locations. (e) Extra-framework cations are active sites for NH_3_ adsorption. (f) NH_3_ adsorption isotherms onto ion-exchanged LTA zeolite at 303 and 423 K. The activation temperature was 620 K. (g) Crystal structure of Na-Omega (Ω) zeolite including cation locations. (h) Extra-framework cations are active sites for NH_3_ adsorption. (i) NH_3_ adsorption isotherms on Na-, La-, and H- Ω and Ga substituted analogues at 323 and 473 K. The activation temperature was 673 K. Si/Al ratio of 4.45 for Al-Ω and Si/Ga ratio of 4.31 for Ga-Ω. Data source: refs 149, 168 . (j) Simulation data of NH_3_ adsorption in all-silica FAU. Data source: refs 100, 151, 159 . (k) Simulation data of NH_3_ adsorption in all-silica and aluminosilicate LTA zeolites. Data source: refs 48, 100, 151, 161 .

An aspect that should be highlighted is the choice of equilibrium adsorption measurement techniques (i.e., volumetric or gravimetric) for gathering the NH_3_ uptake data available in the literature. For instance, Joshi *et al*. used gravimetric measurements to report 10.8 mmol/g of NH_3_ adsorbed at 303 K and 0.7 bar onto the Na-X (Si/Al = 1.15) and about 7.7 mmol/g at 393 K.^149^ The latter datum agrees with the uptake amount of 8.2 mmol/g at 393 K and 0.7 bar, as reported by Barrer and Gibbons for Na-X (Si/Al = 1.34), who also employed gravimetric measurements.^125^ Helminen *et al*. used volumetric measurements to find NH_3_ uptake of 9.3 mmol/g at 298 K and 1.0 bar.^45^ Such differences could be attributed to adsorbent differences or sample activation but could also be associated with the amount of equilibration time intervals used by the researchers when capturing the NH_3_ uptakes. For instance, Joshi *et al*. and Barrer and Gibbons used equilibration times of 2 h per isotherm datum; Helminen *et al*. offered no details about the equilibration time used but indicated that equilibrium was reached. For FAU Y, Shiralkar and Kulkarni^158^ reported an equilibrium gravimetric adsorption amount of 8.8 mmol/g for Na-Y (Si/Al = 2.31) at 333 K and 0.5 bar, while Liu and Aika^46,156^ and Boddenberg *et al*.^157^ reported an equilibrium volumetric uptake of 6.6 mmol/g at 323 K and 0.7 bar.

Extra-framework cations (including Na^+^) enhance the first-order electrostatic interactions with NH_3_’s dipole, with the effect depending on the cation’s charge, diameter, and location. Several monovalent, divalent, and trivalent cations, including alkali, alkaline earth, and transition metals, have been incorporated into the FAU structure and tested for NH_3_ adsorption (Table 2 and Figure 6). Among alkali metals exchanged into FAU X, Rb and Cs (i.e., Rb-X and Cs-X) exhibit the largest NH_3_ adsorbed amount (compared per unit cell since there are significant differences in atomic mass of some cations), even when compared to Na-X. At first, this seems surprising because (a) the first-order ion-dipole interactions should be weaker for larger cations, and (b) larger cations cause a larger reduction of the accessible pore volume. Computational investigations will be needed to explain these observations. Here, we speculate that the larger adsorbed amounts of NH_3_ obtained for Rb-X and Cs-X may reflect that the larger cations occupy locations that are sterically more accessible (i.e., the large cages), allowing for higher coordination numbers, and are easier to displace from the framework surface. This increases their accessibility for additional NH_3_ molecules. Ring puckering has been observed in geometry optimizations using density functional theory (DFT) of FAU with alkaline earth cations from Be to Ba in site SII.^177^ In terms of cation location in dehydrated FAU, Na^+^ prefers sites SII, SI, and SI’ (SIII and SIII’ are only occupied when the number of cations per unit cell is higher than 64; (Figure 6).^178^ K^+^ is frequently found in SII and SI, while Rb^+^ prefers SII and SIII,^178,179^ and Cs^+^ prefers SIII and SII’.^178,180,181^ These alkali cation position distributions, along with the NH_3_ uptake data (Table 2 and Figure 6), indicate that the most suitable sites to adsorb NH_3_ follow the order III, III’ > II, II’ > I’ > I. SI would be expected to be the weakest cation position in terms of cation–NH_3_ binding, given their location within the sodalite building blocks, which are sterically restricted for the access of NH_3_. Finally, it is worth noting that the cation preferences for sites described above are for dehydrated FAU samples; it has been reported that even traces of water could reposition the cations to locations within the supercage or even the sodalite cages.^178^ The polar NH_3_ molecule could produce a similar trend.

Simulation studies have attempted to quantify the impact of Al substitutions at distinct T-sites both through (a) binding energies of NH_3_ to each of these sites and (b) the relative energies of Si → Al exchange reactions at the T-site. Zhakisheva *et al*. used force-field-based simulations to observe zeolite-specific differences in the binding energies of NH_3_ to Al sites in model systems that have replaced one Si atom with Al + Na.^182^ Within FAU, the enthalpy of adsorption for NH_3_ varies from site to site by less than 1 kJ/mol, while for both MFI and MOR, there is a spread of 4–6 kJ/mol.^182^ The inclusion of multiple Al substitutions was considered for FAU, where the adsorption enthalpy of NH_3_ changes from −29 kJ/mol to −85 kJ/mol as the number of substitutions increases from 1 to 96. While the absolute value of the adsorption energy is underestimated with respect to experiment (∼60 vs 75 kJ/mol), trends in NH_3_ adsorption are reflected when H_2_O is considered instead. It is worth noting that in quantum mechanical calculations for embedded cluster models, where various levels of electronic structure theory are applied, the binding energy of NH_4_^+^ in H-FAU falls within experimental bounds,^145^ and methods comparison studies show that careful consideration of dispersion interactions is needed.^183^

Experimentally, alkaline earth metal cations in FAU are preferentially located in SII or SI, and reports have not identified SIII hosting these cations. Given these cations’ similar charge and polarizabilities, their location is linked to showcasing similar interactions with NH_3_. The intensity of the interaction of Ca-Y with NH_3_ is evident in the level of irreversibility of about 3.2 mmol/g at 333 K.^158^ Sr^2+^, however, produces a larger uptake of NH_3_, plausibly due to the preferential location of the cation on FAU sites that are sterically favored for interactions (i.e., SII). Meanwhile, La^3+^ is found to be located in SI’, SI, SII, and SV;^178,184,185^ SV could exhibit favorable interactions with adsorbates given that it is positioned at the center of the 12-ring window of the FAU supercage, but for NH_3_, the interactions are found to contain a high degree of irreversibility and the capacity might be impacted by effective cage blockage by La^3+^.

For FAU Y, the cation associated with the largest observed NH_3_ uptake was Ag^+^, followed by alkaline earth metals, other transition metals, and alkali metals. Ag^+^ can be exchanged to all FAU sites except for SIII.^178^ Most occupy SII in dehydrated FAU,^178,186^ but simultaneous occupancy of SI, SI’, and SII’ is also possible.^187,188^ Besides the advantageous location of Ag^+^ in FAU, the cation holds significant chemical activity that allows for additional specific interactions, particularly with adsorbates with unpaired electrons (i.e., NH_3_). In the case of transition metals Co and Cu, these have been reported to strongly interact with multiple NH_3_ molecules to form complexes like [Co­(NH_3_)_6_]^2+^ or [Cu­(NH_3_)_4_]^2+^,^189,190^ with considerable levels of irreversibility.

##### 2.1.1.2 Linde Type A (LTA)

Similarly to FAU, the location of the cation in LTA is of great relevance for the interactions with NH_3_ (Figure 6d and 6e). LTA zeolites are also very well-known for showcasing modulation of pore diffusion based on the existing extra-framework cation,^191–193^ which impacts the transport of NH_3_. Mechanistic simulation studies of Na-LTA with CO_2_ and CH_4_ guests revealed a “trap door” mechanism whereby Na^+^ can be temporarily displaced from its Al site by the permanently (quadru)­polar CO_2_, but cannot be moved by the nonpolar CH_4_.^194^ Since NH_3_ has a polar nature and can bind strongly to Na^+^, Na-LTA is expected to selectively promote the diffusion of NH_3_ over less polar adsorbates such as H_2_ and N_2_. The LTA variants reported to have the largest NH_3_ uptake capacities at ambient conditions experimentally are those containing transition metal cations (Figure 6f; i.e., Ag­(I)-LTA, Cd­(II)-LTA, and Zn­(II)-LTA).^150,162^ Even though in Figure 6f data are presented in mmol/g, considering the unit cell composition, transition metal-LTA materials exhibit a higher NH_3_ adsorbed amount per unit cell due to significant differences in atomic mass of the cations. The distribution of Ag^+^ in LTA is favorable for NH_3_ adsorption due to accessibility. Kim and Seff found that, from 12 Ag^+^ per unit cell, around 75% are located in SI and SII, located near the wall of the main LTA cage. In addition, some Ag^+^ have been found in SIII, at the very center of the pore.^195^ The favorable location of Ag^+^, as well as its higher electronegativity compared with, for example, Na^+^ (1.93 and 0.93 Pauling, respectively), are therefore consistent with the more significant NH_3_ adsorbed amounts among the LTA variants studied (Figure 6).

Zn^2+^ is usually preferentially located in SI and SIII sites in dehydrated LTA, coordinated tetrahedrally to 3 and 4 framework oxygens, respectively.^196^ Remarkably, from 6 Zn^2+^ per unit cell, only one cation has been found within a sodalite cage. The exposed location of most of these Zn^2+^ cations might explain the observed significant NH_3_ uptake compared with divalent cations containing LTA variants.^150,162^ However, NH_3_ uptake is still lower than that in Ag-LTA, perhaps because of the smaller number of Zn^2+^ cations per unit cell. Seff *et al*. found that Cd^2+^ cations are preferentially located within the LTA main cage, with about 26% at the center of the 8-membered ring (SII).^197^ However, the Cd­(II)-LTA samples in that study were overexchanged with Cd^2+^ to yield about 9.5 cations per unit cell. This makes it challenging to elucidate the source of NH_3_ uptake capacity seen in Figure 6f since the uptake data obtained by Coughlan and Shaw were for Cd-LTA samples containing about six cations per unit cell.^150^

Crystallographic studies have found that only 25% of Co^2+^ cations are in the LTA sodalite cages.^150,198^ Coughlan and Shaw^150^ concluded that the strong interactions between NH_3_ and transition metal cations are based on the formation of complexes. They reported color changes in the LTA samples upon NH_3_ adsorption onto Co­(II)-LTA, Ni­(II)-LTA, and Ag­(I)-LTA due to changes in coordination. This has also been observed by Liu *et al*. for Co^2+^ and Cu^2+^ in FAU-Y, in the form of complex type M­(NH_3_)_*x*_(OH)^+^.^46^ The complexation of NH_3_ could also lead to irreversibility, even after degassing under vacuum-assisted thermal swing. The maximum enthalpy of adsorption of NH_3_ observed for transition metal-containing LTA is in the range of 50–70 kJ/mol.Simulated adsorption isotherms of NH_3_ for all-silica and Na-exchanged aluminosilicate zeolites show that the presence of strong adsorption sites in Na-LTA results in a lower adsorption onset pressure compared to all-silica LTA (Figure 6k).^161^

##### 2.1.1.3 MFI Zeolites

For zeolites with mid to high Si/Al ratios, there are only a few experimental reports on the adsorption of NH_3,_ with the data primarily available for MFI-type zeolites. In contrast, all-silica zeolites are the most well-represented group in simulation studies because the inclusion of multiple Al substitutions leads to a combinatorial complexity of their possible arrangements, while all-silica zeolites possess a well-defined structure. The availability of empirical adsorption data within MFI-type zeolites with high Si/Al ratios (particularly Si/Al ∞) allows for close comparisons between methodologies. Experimentally, Boddenberg *et al*. reported a NH_3_ adsorbed amount of 3.9 mmol/g at 1.0 bar and 303 K for Na-ZSM-5 with Si/Al l:32. Using a statistical thermodynamics model and calorimetric data, they concluded that the interactions of NH_3_ molecules with extra-framework Na^+^ were responsible for the total uptake and lead to the possible formation of Na­(NH_3_)_s_^+^—complexes (s: 1–6).^166^ They also found evidence of hydrogen bonding interactions between NH_3_ and the framework oxygen.^166^ In another study, Zarębska *et al*. evaluated the NH_3_ adsorption at 298 K and 1.0 bar onto all-silica and Si/Al l:20 MFI zeolites.^165^ Although both materials exhibit similar NH_3_ adsorbed amounts (i.e., 3.01 and 3.00 mmol/g, respectively), the isotherm data at lower pressures exhibit distinct behavior (Figure 7c).

**Figure 7:**
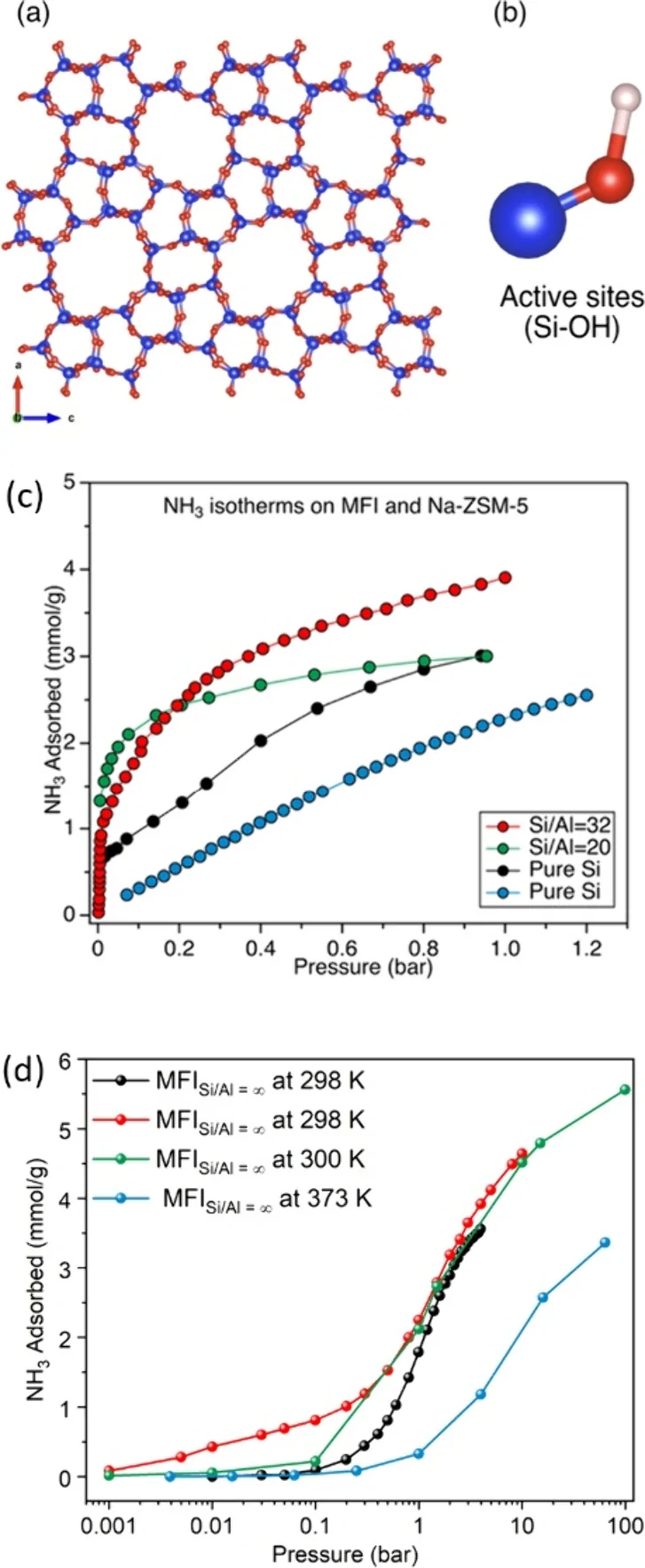
(a) Crystal structure of (pure Si) MFI zeolite. (b) Silanol groups (Si-OH) in pure Si MFI zeolite can act as sites for NH_3_ adsorption. (c) NH_3_ adsorption isotherms on MFI zeolite with different Si/Al ratios at 288 K (blue) and 298 K (red, green, and black). The activation temperatures were 623 K (blue) and 723 K (red). Data source: refs 151, 165, 166 . (d) Simulated NH_3_ adsorption isotherms in all-silica MFI. Data source: refs 100, 110, 151, 159 .

The material with Si/Al:20 adsorbs 50% of the total capacity at an NH_3_ pressure lower than 0.02 bar, while the pure silica MFI needs pressures of almost 0.5 bar. Jänchen *et al*. correlated the population and strength of Brønsted sites with the affinity toward NH_3_, concluding that the surface content of those sites decreases with an increase in the Si/Al ratio.^199,200^ For pure silica MFI, it has been reported that silanol groups behave as weak Bro̷nsted sites^201^ and also interact with NH_3_ molecules. However, the difference in the strength of this type of site can explain the larger NH_3_ capacities observed for low Si/Al ratios, as corroborated by the heat of adsorption data.^166^

Simulated adsorption isotherms in MFI feature a perfect, defect-free crystal. Correspondingly, Henry’s constant is dramatically underrepresented in simulation results compared to experiments, and at the state point 298 K and 1 bar (Table 2), simulated adsorption is less than half of its synthetic counterparts (Figure 7d). This fact was carefully considered during the validation of the TraPPE-zeo force field,^202^ where experimental data for water adsorption in all-silica MFI with extremely low defect density include a step change in loading that occurs at a pressure more than 4 orders of magnitude lower than for other all-silica samples.^203^ The TraPPE-zeo force field applied to a defect-free MFI structure agrees only with the data for the highest-quality synthesized material.

Matito-Martos *et al*. combined experimental methodology with classical molecular simulations to evaluate the adsorption of NH_3_ onto pure-silica MFI (Figure 7d) and found 2.3 mmol/g adsorbed at 298 K and 1.2 bar,^151^ similar to what was observed by Zarębska *et al*.^165^ The computational effort by Matito-Martos *et al*. suggests that the interaction mechanism governing this system is related to hydrogen bonding and the presence of small concentrations of extra-framework H^+^. Although low-pressure NH_3_ loading varies for MFI across research groups, emphasizing the differences in the hydrophobicity of various force fields, the agreement is good at high pressure due to identical pore volume.

In the absence of metal substitutions, the interaction mechanism of NH_3_ and all-silica zeolites is heavily influenced by the presence of silanol groups over the exposed surfaces of these materials. The ability of NH_3_ to hydrogen bond with silanol moieties (binding energy −13.6 kJ/mol at 300 K both experimentally and via first-principles MP2/6-31++G**)^204^ can lead to preferential adsorption of NH_3_ to these sites over either H_2_ or N_2_, enhancing the adsorption selectivity of NH_3_ in binary mixture simulations comparing the bulk MFI framework to a nanosheet that terminates with surface silanols (surface density ∼ 3 OH/nm^2^).^110^ This effect is easily observed via density profiles of MFI bulk and nanosheet, where the surface NH_3_ density is greater than the corresponding location in the bulk, and the surface N_2_ density simultaneously decreases (Figure 8). Due to surface silanol groups in MFI nanosheet, the overall adsorption selectivity for NH_3_ over N_2_ increases by almost a factor of 2 at 373 K and 5 bar, from 8.92 to 16.3, while for NH_3_/H_2_ the effect is milder, increasing slightly from 60.2 to 69. At elevated temperatures, this adsorption selectivity is of course diminished as entropic contributions become more prominent.

**Figure 8:**
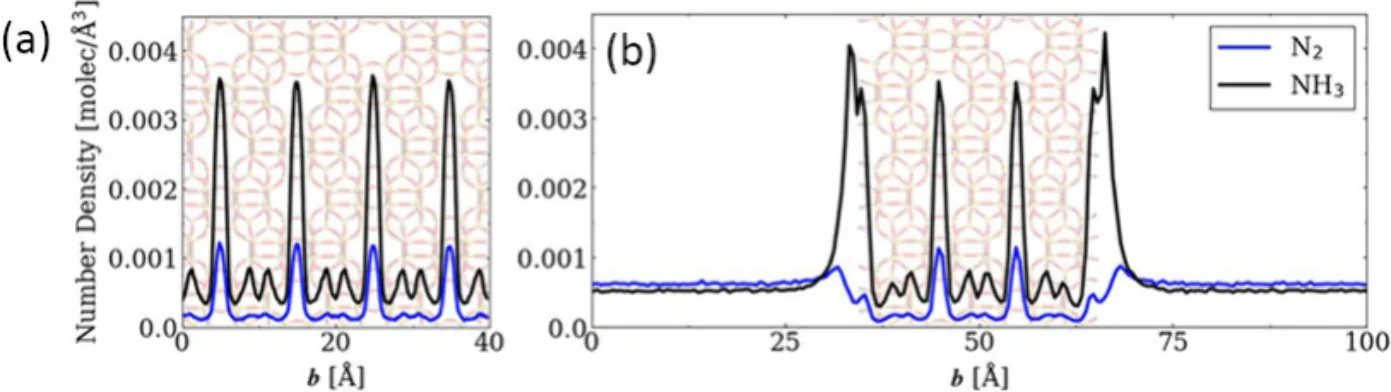
Simulated number density profiles (molecules per Å^3^) of NH_3_ (black) and N_2_ (blue) along the b direction at *T* = 523 K and *P* = 80 bar in bulk (pure Si) MFI zeolite (a), and MFI nanosheet (b). The background shows the bulk MFI (left) and zeolite MFI nanosheet (right) models with the c direction as the vertical axis. The bin width in the b direction is 0.5 Å. 8a and 8b adopted with permission from ref 110. Copyright 2022 American Chemical Society.

##### 2.1.1.4 Omega (MAZ)

Zeolite omega (Ω), which possesses a Mazzite (MAZ) topology with 12-membered cylindrical channels with a pore size of 7.5Å, is among the mid silica-containing zeolite showing a significant uptake of NH_3_ at ambient conditions, only surpassed by certain FAU and LTA variants and AlPO-11/SAPO-11 (Table 2, and Figure 5a). Mirajkar *et al*. developed aluminum and gallium isomorphs of zeolite omega (Ga-Ω refers to the structure where the Al^3+^ atoms were replaced by Ga^3+^ atoms in the same ratio and crystallographic positions). Both were postfunctionalized to include H^+^ protons and the trivalent cation, La^3+^, for NH_3_ adsorption at 0.6 bar and temperatures ranging from 323 to 523 K.^168^ When comparing the extra-framework Na^+^ containing variants of both isomorphic materials, Ga-Ω showed a slightly larger NH_3_ capacity than Al-Ω (6.5 and 6.1 mmol/g, respectively), with the affinity of the former reflected somewhat in the enthalpy of adsorption (Figure 9). The difference could be ascribed to the superior electronegativity of Ga^3+^ compared to Al^3+^ (1.82 and 1.47 Pauling, respectively).^205^

**Figure 9:**
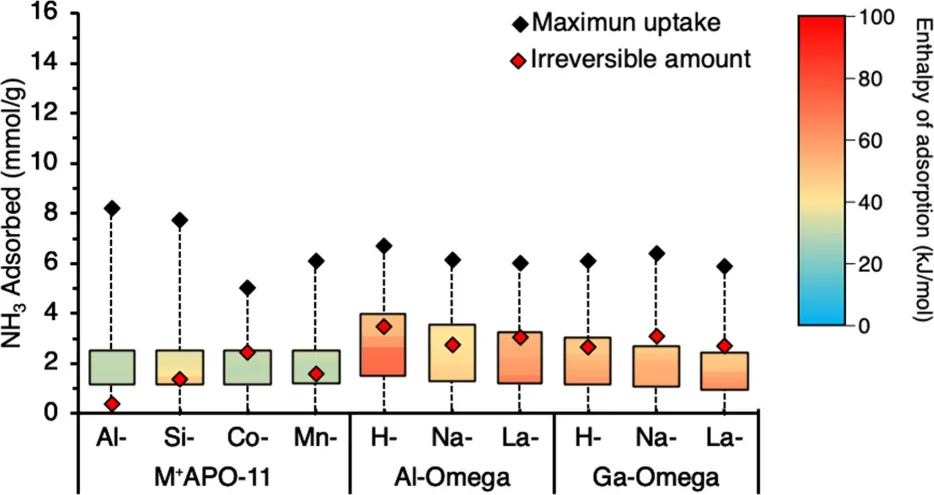
NH_3_ adsorbed amount for AlPO_4_-11 and analogous structures containing Si, Mn, or Co, as well as Ω zeolite and modifications. Al-Ω refers to the original structure containing Si and Al, while Ga-Ω refers to the material obtained after Ga replaces all the Al atoms. H^+^ or La^3+^ replaced extra-framework cations that balanced the charges in the Ω zeolite and were introduced via ion exchange. Black diamonds represent the maximum adsorption capacity reported at low temperatures (323*–*333 K) and 0.5 bar. Isosteric heat of adsorption as a function of the NH_3_ loading is included as a color bar. The scale color bar has been included as a visual guide to correlate the enthalpy of adsorption values. Red diamonds were used to point to the irreversible amount calculated by the difference of the adsorbed amount at the same temperature between fresh and materials after adsorption. Materials after adsorption were thermally treated (M^+^APO-11 at 723 K for 10 h, and Ω variants at 673 K for 1 h) under vacuum (10^–6^ Torr). Data source: refs 168, 169 .

Penchev *et al*. used NH_3_ TPD experiments to show that acid zeolite omega (H–Al-Ω) contains stronger Bro̷nsted sites than the Na^+^ and La^3+^ forms.^206^ On the other hand, a similar trend in the NH_3_ isosteric heat on Ga-based Ω zeolites (Figure 9) suggests that acid sites in H–Ga-Ω are not as strong. A decrease in acid site strength might be expected upon the replacement of Al^3+^ by Ga^3+^_,_ and this has been observed for isomorphs of ZSM-5, namely H^+^-Al-ZSM-5, H^+^-Ga-ZSM-5, H^+^-Al-MFI, and H^+^-Ga-MFI.^205,207,208^ Interestingly, the irreversible NH_3_ adsorbed amount remaining across the zeolite omega variants after vacuum-assisted thermal treatment (10^–6^ Torr and 673 K for 1 h) remains practically identical (red diamonds data in Figure 9; ca. 44–52%). This suggests that other interactions are taking place, plausibly including hydrogen bonding.

Simulation results for MAZ-like zeolites are comparatively underrepresented in the literature, even in high-throughput applications.^159^ Only 95 of the 256 zeolites in the IZA database have been synthesized in their all-silica forms, which tend to be emphasized computationally because the remaining 161 zeolites require an explicit consideration of Al substitutions to compare with experiment. As discussed earlier, the inclusion of Al substitutions is a combinatorically complex modeling problem because the experimental site–site distribution cannot be feasibly captured by a single structure that is small enough to run particle-based simulations on. This challenge can be addressed by incorporating existing data about which T-sites are most prone to substitution, modeling larger zeolite supercells such that individual simulations contain a more representative distribution of Al sites, including many simulation replicas, and/or performing systematic studies. There is also a question of force field suitability for the Al sites and its counterion. The Calero group has developed a zeolite force field that includes Al and Na parameters, which was benchmarked against CO_2_ adsorption data.^209^ Such force fields are intended to be transferable and perform well when the guest species is relatively nonpolar (e.g., CO_2_ swapped for CH_4_). Strongly interacting polar species like H_2_O or, to a lesser extent, NH_3_, are highly sensitive to the interaction parameters, and in the absence of robust experimental data (multiple groups reproducing the same isotherm) it is difficult to assess the validity of the model for NH_3_ separation applications.

#### 2.1.2 Aluminosphosphates

Aluminophosphate AlPO_4_-11, a structure characterized by not having extra framework cations, and silicoaluminophosphate SAPO-11 appear to have the strongest affinity and reported NH_3_ uptakes at ambient or near ambient conditions among all zeolite-like materials considered in this review, adsorbing up to 15.4 and 15.1 mmol/cm^3^ (Table 2 and Figure 10).^169^ These zeolitic materials are isostructural with an AEL topology and exhibit elliptical pore windows of size 6.5 × 4.0 Å that give access to 1D channels sufficiently wide to allow for multiple orientations of NH_3_ molecules. The larger electronegativity difference between Al and O atoms (compared to Si and O atoms) leads to larger bond dipoles in the framework that provide stronger dipole–dipole interactions with NH_3_. These oxygen atoms with a larger negative (partial) charge are also better hydrogen-bond acceptors. Prasad and Vetrivel found that oxygen atoms from the 10-member channel in AlPO-11 interact with hydrogen atoms from the NH_3_ molecule (Figure 10), particularly at sites that display the lowest P–O–Al angles, resulting in the lowest steric hindrance.^210^ On the other hand, the isomorphous substitution of phosphorus by silicon that yields SAPO-11 results in Bro̷nsted sites capable of interacting strongly with the lone-pair electrons of the NH_3_ molecules.

**Figure 10:**
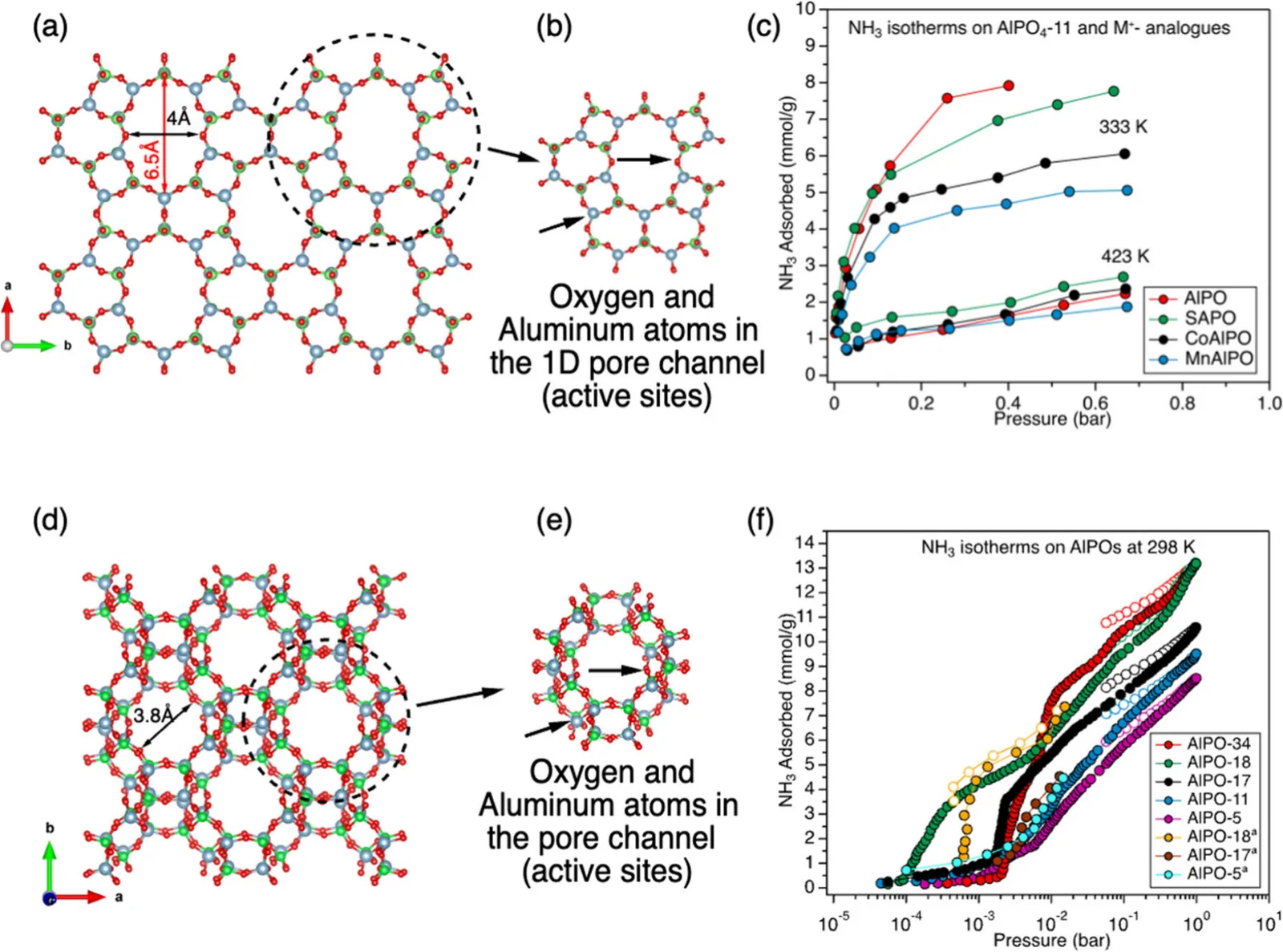
(a) Crystal structure of AlPO_4_-11 (AEL) with 10MR 1D pore system along *c*-axis. (b) sites for NH_3_ adsorption: oxygen, and aluminum atoms. (c) NH_3_ adsorption isotherms on AlPO_4_-11 and Si-, Co- and Mn- substituted analogues at 333 and 423 K. The activation temperature was 723 K. (d) Crystal structure of AlPO_4_-18 (AEI) showing the 3.8Å channel along the *c*-axis. (e) Active sites for NH_3_ adsorption. (f) NH_3_ adsorption isotherms (*x*-axis in log-scale) at 298 K and up to 1 bar onto AlPO_4_-34, −18, −17, −11, and −5. Open symbols represent desorption data. Data source: refs 169, 170, 211 .

At the chosen conditions, AlPO_4_-11 adsorbs slightly more NH_3_, but SAPO-11 displays larger heats of adsorption (Figure 9), i.e., it offers fewer but stronger adsorption sites.^169,210^ This is also supported by the amount of irreversibly adsorbed NH_3_ held after vacuum-assisted thermal treatment (723 K for 1 h) (see red diamonds data in Figure 9). Interestingly, the amount of NH_3_ held irreversibly at 333 K by SAPO-11 is equivalent to about 3.2 molecules of NH_3_ per unit cell, which coincides with the content of Bro̷nsted sites per unit cell.

Izmailova *et al*. studied NH_3_ adsorption in aluminophosphate materials, such as AlPO_4_-18, AlPO_4_-17, and AlPO_4_-5, under low-pressure and room-temperature conditions (Figure 10).^211^ Under these conditions, they observed some inflections in the isotherm at specific pressure values. A similar behavior was observed for the CH_3_OH and H_2_O adsorption onto the same materials. The ability of Al atoms to form complexes with water molecules within the aluminophosphate framework has been demonstrated through ^27^Al NMR and XRD studies.^212–214^ Those changes in the Al coordination with polar molecules, such as CH_3_OH, H_2_O, and NH_3_, are responsible for the inflection points in the isotherms of polar adsorbates on aluminophosphate materials. To the best of our knowledge, no simulation studies have been conducted for the adsorption of NH_3_ in SAPOs.

In a recent study, Yue *et al*.^170^ extended Izmailova’s et al.^211^ work, evaluating the performance of several topologies of aluminophosphate frameworks (i.e., AlPO-5, AlPO-11, AlPO-17, AlPO-18, and AlPO-34) as a promising alternative for NH_3_ capture at room temperature and up to 1 bar. Results corroborated the high affinity of NH_3_ toward AlPOs reaching a batch equilibrium adsorption capacity of >13 mmol/g for both AlPO-34 and AlPO-18, and 11.5 mmol/g for AlPO-11 at 1 bar. Interestingly, no saturation plateau was observed for any of the studied materials, leaving the possibility to offer higher adsorption capacities at higher pressure. Spectroscopic analyses and theoretical calculations were used to elucidate the adsorption mechanism, revealing the selective coordination of NH_3_ onto framework aluminum sites, consistent with Izmailova’s *et al*. earlier findings. Specifically, for AlPO-11, it was demonstrated that a quarter of the framework Al^IV^ sites can be transformed to Al^V^, and a quarter to Al^VI^ sites. In the case of AlPO-18, > 40% of Al^IV^ sites can be transformed toward Al^VI^ upon ammonia saturated adsorption, which resulted in framework collapse. The potential stability of AlPO-11 was further supported via multicycle dynamic breakthrough adsorption, where the capacity was maintained throughout 10 cycles.

#### 2.1.3 Carbons

Carbon-based adsorbents are another well-established class of materials with good potential to adsorb NH_3_ at harsh conditions due to excellent mechanical strength and high chemical and physical stabilities. However, these materials are still poorly understood due to the lack of chemical and structural composition information. Qajar *et al*. studied the influence of morphologies and textural properties of micro- and mesoporous (either amorphous or ordered) carbon materials that were activated with CO_2_ and then treated with concentrated nitric acid before the uptake of NH_3_ (Table 2).^174^ The results showed that the microporous carbons perform better at 298 K and 1 bar than the mesoporous counterparts, whether with amorphous or ordered structures (Figure 11a). The NH_3_ uptake mechanism, as deduced from X-ray photoelectron spectroscopy (XPS) and FTIR data analyses, is related to interactions of NH_3_ with oxygen-containing functional groups in the effective surface, which results in the formation of amines and a small number of amides.^174^ Although the stronger dispersion interactions afforded by microporous carbons might enhance the uptake at low-to-moderate pressures, uptake at high NH_3_ pressure ultimately benefits from mesoporosity (Figure 11a). Adsorbate–adsorbate interactions become a significant driving force in large pores. Interestingly, most mesoporous carbon materials depict a type IV NH_3_ adsorption isotherm characterized by capillarity condensation. Qajar *et al*. did not show equilibrium desorption leg data in their report, but did quantify irreversible NH_3_ uptake in the carbons based on dynamic tests.

**Figure 11:**
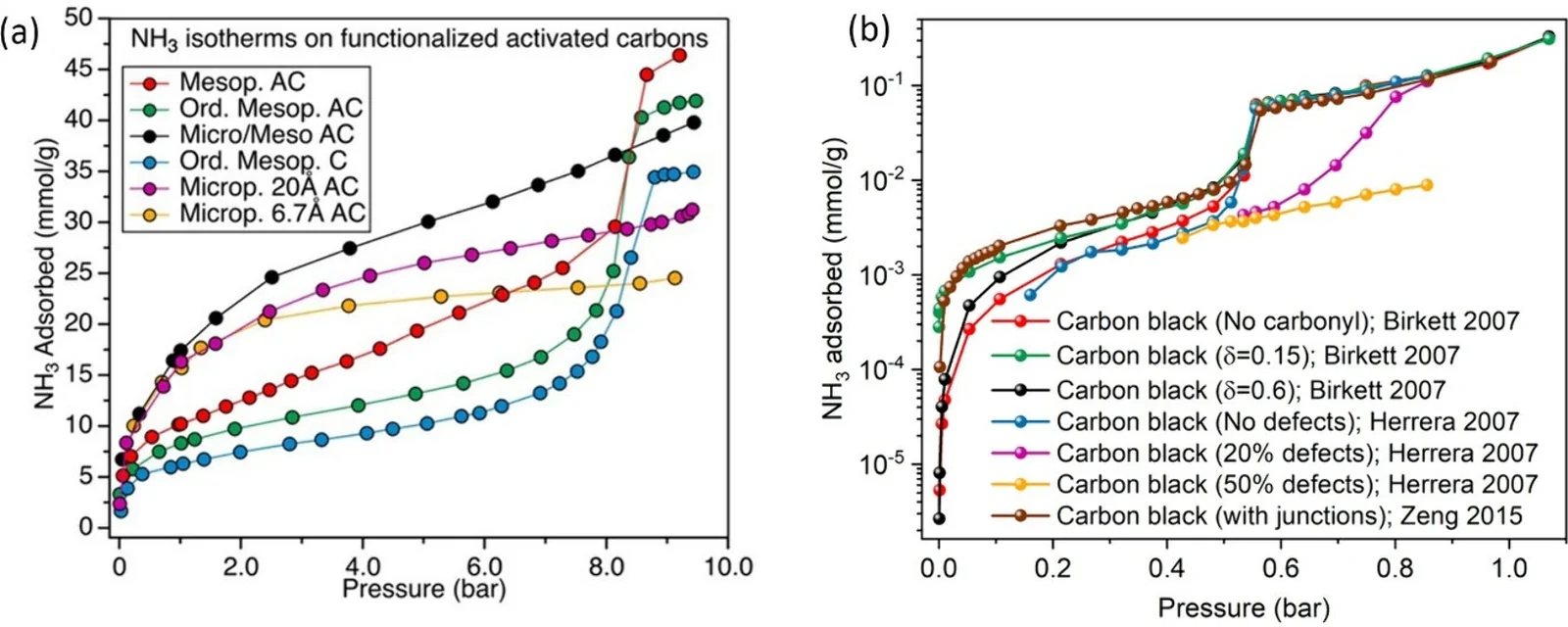
(a) Ammonia adsorption isotherms onto carbon-based materials at 298 K. All materials were activated with CO_2_ and concentrated nitric acid. (b) Simulated adsorption data at 240 K. Data source: refs 174,215–217 .

Simulation results for NH_3_ adsorption in black carbons generally agree with expectations regarding polar sites driving adsorption (Figure 11b). Within graphitized carbon black, graphite sheets are modeled as terminating in hydroxyl groups, forming favorable NH_3_ adsorption sites at sheet–sheet junctions.^215^ The presence of an ultrafine functionalized pore leads to a distinct step in the isotherm at 240 K (the normal boiling point of NH_3_) as the ultrafine pore fills with NH_3_ and 2D condensation across the plane occurs. There is good qualitative agreement with experimental measurements on Carbopack F. At significantly reduced temperatures (195 K, the NH_3_ triple point), the presence of the ultrafine pore leads to an adsorption hysteresis loop, both in simulation and experiment, but the hysteresis vanishes at 240 K. The adsorption isotherm at low pressure and the low loading isosteric heats of adsorption have also been found to be sensitive to the spatial patterning of carbonyl groups on the graphene surface at 240 K.^216^ Of particular interest is the finding that carbonyl groups are most impactful when densely clustered, with the strongest effect observed when adjacent carbonyl groups are separated by less than 0.15 nm. By the time carbonyl groups are separated by 0.6 nm, their influence on the adsorption isotherm is nearly negligible even at 10^–4^ bar. In the high-pressure limit, where full surface coverage of NH_3_ is achieved, the isotherms converge. Other studies report, however, that the ability for 2D condensation to occur is related to surface roughness, with a surface defect percentage that relates to the number of missing carbon atoms from the uppermost graphene layer.^217^ At defect concentrations as low as 10%, the adsorption isotherm no longer features a step, and at 50% very little adsorption is observed at all.

While it is valuable to understand the physicochemical mechanisms leading to the isotherm’s attributes, simulation approaches to studying black carbon materials suffer from spatiotemporal limitations where the system sizes needed to directly compare with experiment are inaccessible. That is, the structural complexity of black carbon on the mesoscale is absent from model surfaces that are limited to the nanometer regime. Consequently, these simulation studies report adsorbed amounts normalized by surface area rather than volume, because the mesoporous character that gives synthetic samples their volume is inadequately captured. Nonetheless, there is remarkable agreement between simulated and experimental results (in units normalized by area) considering the partial disorder inherent to these systems. In Figure 11b we have used the BET area of Carbopack F, 4.9 m^2^/g,^215^ to convert loadings from per-area to per-volume units.

### 2.2 Adsorption at High Temperature

Studies for the uptake of NH_3_ at high temperatures are very scarce, with most focusing on zeolites or zeolitic adsorbent materials. The following sections present a compilation of the high-temperature data available.

#### 2.2.1 Aluminosilicates

##### 2.2.1.1 Faujasite (FAU)

Figure 12a–c shows reported NH_3_ equilibrium uptake capacities as a function of temperature for several FAU X and Y variants. As expected, the adsorption amounts decrease considerably with increasing size of the cation and with increasing temperature. The most interesting observation is that, at a given temperature, the adsorbed amount of NH_3_ increases with increasing Si/Al ratio for Na-X, whereas the reverse is found for Cs-X. As the temperature increases, the entropic contributions dominate the free energy, and enthalpic differences, as related to binding energies to the various ions, become less important. Barrer and Gibbons observed the adsorption of NH_3_ in sodalite cages and suggested that the rate of NH_3_ migration into the cages increases considerably at temperatures higher than 370 K.^125^ Another observation from Figure 12 is the effect of the Si/Al ratio (i.e., the amount of cation sites). Simulated temperature-dependent studies of FAU in the literature, for example from Matito-Martos *et al*.,^151^ span temperature ranges that do not exceed 298 K and so are not included in this section. In principle, however, simulation studies are capable of probing HB-relevant state points, and the focus on the low-temperature regime is likely intended to facilitate closer comparison with available experiments.

**Figure 12:**
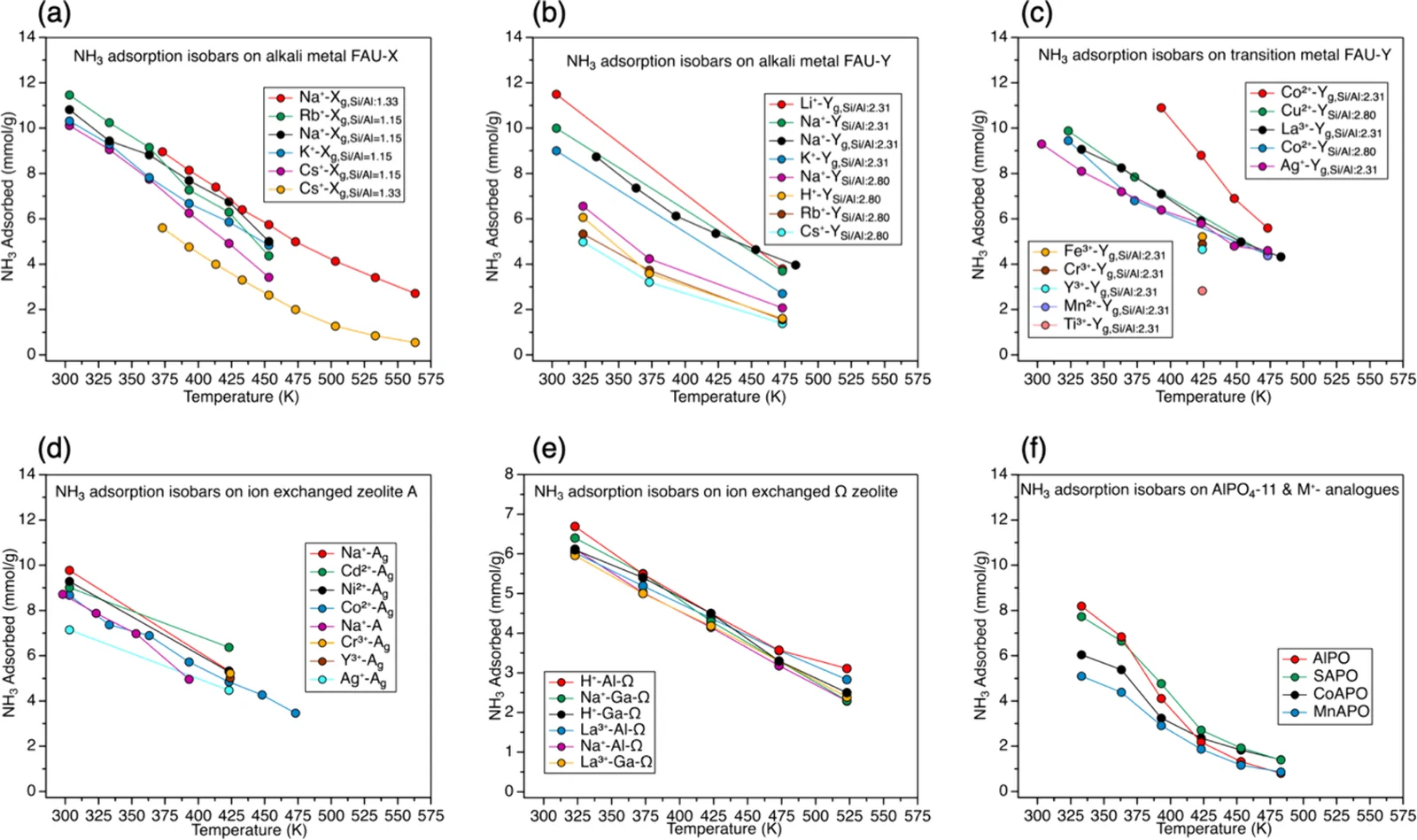
NH_3_ isobars onto: (a) Na-X and ion-exchanged FAU-X. (b) Alkali and earth alkaline metal exchanged FAU-Y. (c) Transition metal exchanged FAU-Y. (d) Ion-exchanged zeolite LTA. (e) Ion-exchanged omega zeolite. (f) AlPO_4_-11 and Si-, Co-, and Mn- analogues. The NH_3_ equilibrium pressure was around 0.6–1.0 bar. Subscript g refers to adsorption data gathered via gravimetric method. Data source: refs 45, 46, 125, 127, 139, 149, 150, 154,^156–158^,^161^,^162^,^168^,^169^ .

Figure 12 b–c shows the reported NH_3_ equilibrium uptake capacities for FAU Y zeolites containing alkali, alkaline earth metal, and transition metal cations. Similar to what has been observed for FAU X, smaller Si/Al ratios in FAU Y result in larger NH_3_ uptake across the whole temperature range. For alkali or alkaline earth metal FAU Y, adsorption uptake data generally follow the trend expected from ion–dipole interactions, i.e., larger valence and smaller size of the cation favor higher uptake.

At a temperature of ca. 400 K, Co­(II)-Y (Si/Al:2.31) has a superior NH_3_ equilibrium adsorption capacity compared to other FAU zeolites containing extra-framework transition metals (Figure 12c). When comparing the data reported for uptakes in Co-FAU for two different Si/Al ratios, the increase in uptake capacity could be due to the location of Co^2+^ in the FAU lattice, in sites that would be favorable to interact with NH_3_. However, the interaction between NH_3_ and Co^2+^ leads to the formation of complexes like Co­(NH_3_)_6_^2+^,^190^ with considerable levels of irreversibility. It should be noted that the NH_3_ capacity of Ag­(I)-Y also stands out for the whole range of temperatures reported in mmol/u.c. (Table 2), and this could be associated with the ease of Ag^+^ cations to settle in different locations. Among the zeolites containing trivalent extra-framework transition metals, La­(III)-Y has the highest reported NH_3_ capacity, probably because of the location of some La^3+^ cations in the FAU supercage (SV). The other trivalent cations have been reported to be in SI or SI’. We note that only single point, high-temperature NH_3_ adsorption data have been reported for most trivalent cations-containing FAU zeolites.

Although many FAU zeolites withstand high temperatures based on thermogravimetric data and structural analysis, it is known that they go through a pore-breathing phenomenon while adsorbing and desorbing guest species. Tokushige and Ryu reported data from experiments for the gravimetric adsorption of NH_3_ onto materials that included FAU (H- and Na-Y) and LTA (Na-A) at 473 K and 5 bar, including X-ray diffraction data from the adsorbents prior to and after desorption.^49^ The adsorption/desorption of NH_3_ resulted in the zeolite crystal’s expansion and contraction, but the zeolite particles’ morphologies were not affected after five cycles. Most importantly, the cycling process did not impact the NH_3_ working adsorption capacities.

Simulation studies have considered the impact of zeolite framework flexibility on the adsorption of small, relatively nonpolar molecules (N_2_, CO_2_, alkanes) and found that while the accessible volumes of all 11 zeolites considered (including FAU) were unchanged, loadings could differ by more than 10% for the most polar species, CO_2_.^218^ While we are unaware of studies that quantify the impact of flexibility on NH_3_ adsorption within zeolites, it is reasonable to expect comparable or larger differences than those seen for nonpolar species, especially at elevated temperatures.^219^

##### 2.2.1.2 Linde Type A (LTA)

Figure 12d shows the NH_3_-adsorbed amount as a function of temperature for LTA zeolites.^150,220^ At 423 K, both Cd- and Ag-A offer comparable NH_3_ adsorption capacities, as anticipated given that the interactions between NH_3_ and the cations, as shown by the isosteric heats of adsorption at low coverage, are similar (80 and 70 kJ/mol, respectively, at about 1.5 mmol/g).^220^ However, the rest of the materials present lower capacities, probably because the type of cation is not the main factor contributing to the interactions. Rather, the cation location distribution of cations or other aspects of the surface is more important. As discussed in section 2, for Co- and Ni-A, some of the cations are located in sites unfavorable for interaction with NH_3_, like the sodalite cages.^193,198^

Simulation results for the temperature dependence of NH_3_ adsorption into LTA agree well with experimental results, slightly overpredicting the loading at all temperatures but displaying a similar slope (Figure 12). As seen in Figure 6k, the NH_3_ occupancy is still clearly increasing at 1 bar for temperatures 298–393 K, indicating that LTA is far from saturation. The overprediction of the loading is likely attributed to the force field, where the parametrization of Al and Na attracts NH_3_ too strongly.

##### 2.2.1.3 Omega (MAZ)

Among the zeolite Omega (Ω) variants that have been reported in the literature for the adsorption of NH_3_ at high temperatures (and ca. 1 bar), there is not much variation in uptake amounts (Figure 12e). However, one aspect that needs to be considered in this discussion is the level of NH_3_ irreversibility. When Ga substitutes for Al, the strength of acid sites (i.e., H–Ga- vs H–Al-Ω) decreases,^205,207^ and the amount of irreversible NH_3_ adsorption decreases.^206^ Penchev *et al*. employed temperature-programmed desorption (TPD) measurements to show that NH_3_ desorption in H–Al-Ω takes place between 543 and 823 K, while in Na–Ga-Ω, NH_3_ desorption happens between 443 to 643 K.^206^ Therefore, the Al-Ω variants would not be ideal for ammonia recovery applications but they could serve as adsorbents for NH_3_capture.

##### 2.2.1.4 Aluminophosphates

AlPO_4_-11 and SAPO-11 exhibit significant NH_3_ uptakes at 333 K; however, a marked decay trend is observed as the temperature increases (Figure 12f). As discussed in Section 2.1.2, the adsorption of NH_3_ onto AlPO_4_-11 is dominated by hydrogen-bonding interactions with the oxygens of the 10-membered ring. The strength of these interactions may be insufficient to keep the NH_3_ molecules adsorbed at elevated temperatures. On the other hand, the Bro̷nsted sites in SAPO-11 may promote additional interactions with NH_3_ molecules compared to AlPO_4_-11, and this might explain the slightly larger adsorbed amount of NH_3_ at high temperatures. The isomorphous inclusion of Co^2+^ into the AlPO_4_-11 helps to sustain NH_3_ loadings at higher temperatures, but the variant also shows considerable irreversibility; at 483 K CoAPO-11 retains the equivalent of 1.4 molecules of NH_3_ per unit cell or a ratio of 1 NH_3_/Co, even upon degassing at 723 K for 10 h.^169^ Similar to Co-Y,^46^ it is expected for the structural Co^2+^ tetrahedra to form complexes with NH_3_ even at high temperatures. In the case of MnAPO-11, the NH_3_ uptake at high temperatures is similar to AlPO_4_-11 possibly due to the similarity of acid site types. Wei *et al*. found NH_3_ TPD profiles with a peak for AlPO_4_-11 and MnAPO-11 at temperatures around 450 K, but for SAPO-11 at around 570 K.^221^ The former represents weak Lewis acid sites while the latter Bro̷nsted acid types.

#### 2.2.2 Metal-Salt Composites

Metal salts have been tested for NH_3_ separation under various conditions including MgCl_2_, CaCl_2_, CaBr_2_, and LiCl. Metal salts exhibit a high capacity for NH_3_ absorption even at high temperatures, with demonstrated improvements in reaction separation tests.^13,54^ However, limitations in cyclability, mass and heat transfer, and the need for thermal regeneration have been identified and hinder their practical application.^91^ Therefore, the use of supports based on porous materials has been proposed as an alternative to overcome these limitations. Lapasov *et al*. evaluated the integration of metal salts into the structure of adsorbent materials such as silica gel, zeolites, and carbon molecular sieves via impregnation with MgCl_2_ solutions.^155^ Among the tested supports, zeolite 13X was selected as the optimal support because it exhibited better performance at elevated temperatures, retaining a higher adsorption capacity than the others, reaching 7.3 mmol/g at 373 K and 3 bar. A synergistic effect was observed upon the integration of MgCl_2_ into the zeolite structure, providing active sites for interacting with NH_3_ molecules and improving the adsorption capacity. However, the improvement was primarily observed at 298 K, where the base zeolite 13X adsorbed 9.88 mmol/g, and the sample Z-13X-Mg(0.5) exhibited 11.84 mmol/g. On the contrary, at elevated temperature, the adsorption capacity of zeolite 13X was found to be almost the same or even higher than that of the impregnated samples, as shown in Table 2. Therefore, for room-temperature applications, composite materials with MgCl_2_ exhibit enhanced performance compared to zeolites alone, but at conditions required for HB intensification, such enhancement has not yet been fully established.

Similarly, Hong *et al*. reported MgCl_2_/AC-impregnated materials for NH_3_ separation across various temperatures.^175^ They found an increase of about 40% in the adsorption capacity of the MgCl_2_-impregnated samples, reaching 16.6 mmol/g in Mg(4)-AC compared with 11.8 mmol/g in the bare AC at 298 K and 3 bar. The long-term operation was evaluated via PSA at 313 K using an N_2_ flow for regeneration, demonstrating a stable NH_3_ working capacity over 15 cycles. However, long desorption times (between 60 and 135 min) were needed for Mg-AC composites.

### 2.3 NH_3_ Adsorption at High Pressure and Temperature

Adsorbents for HB application may exhibit high stability under harsh industrial process conditions, such as elevated temperature and pressure. For zeolite materials, almost all of the NH_3_ adsorption data experimentally have been evaluated at low pressure (i.e., 1 bar or lower), as presented in the previous sections of this review. However, San *et al*. assessed NH_3_ adsorption onto AC/Na-FAU composites up to 3 bar, but at 298 K.^50^ They found that the composite with a Si/Al ratio of 1.6 exhibits the highest adsorbed amount of 15.62 mmol/g. In addition, Tokushige and Ryu evaluated NH_3_ adsorption via pressure swing between 5 to 1 bar at 473 K.^49^ They suggested that the application of commercial zeolites for NH_3_ separation under conditions close to the HB synthesis process is feasible. However, for a total HB reactor pressure of 100 bar and a 15% NH_3_ molar composition, the NH_3_ partial pressure is 15 bar. Therefore, data using higher pressures are needed to assess the viability of adsorption as a promising method for separating NH_3_ under industrial conditions.

Aristizabal-González *et al*. evaluated NH_3_ equilibrium adsorption up to 15 bar and 473 K onto commercial Na-X, Na-Y, and Na-LTA zeolites.^48^ Figure 13 shows typical isotherms up to 15 bar and different temperatures onto commercial zeolites. Additionally, in this work the prediction of NH_3_ adsorption in Na-LTA at pressures beyond 15 bar was provided by simulation. At elevated temperature and pressure (473 K and 15 bar), these commercial zeolites exhibit a high NH_3_ adsorbed amount of about 11.4 mmol/g for Na-X. However, this material showed a strong interaction with the cations, which imposes challenges to desorb the NH_3_ and recover it after adsorption. Pressure-, vacuum-, and temperature swings (PSA, VSA, and TSA, respectively) could be implemented for industrial applications. Regarding significant energy saving, the ideal adsorbent should exhibit a high working capacity using a slight pressure swing close to the operating pressure. The authors also performed 5 cycles of PSA and VSA at 473 K. Using VSA, they obtained promising working capacity for all the materials, ca. 4–6 mmol/g, Na-X being the adsorbent with the highest value. Because the VSA requires more energy than the PSA, a direct correlation was found between the working capacity and the Si/Al ratio using a PSA at 473 K, ranging from 11 to 1.1 bar. Na-Y (Si/Al = 2.8) showed the best performance under PSA because, being the material with the lowest cation content (about 50 Na^+^ per unit cell), NH_3_ can desorb more easily from the structure. Therefore, for applications such as PSA, the materials should be able to adsorb a high quantity of NH_3_ at elevated temperatures but in a reversible way to obtain a good working capacity. In simulation, the NH_3_ adsorption isotherm of Na-LTA shows a significant increase in the onset pressure observed with increasing temperature.^161^ The experimental onset pressure remains similar even at high temperatures, indicating that defects and/or chemisorption contributes to the high NH_3_ loading in the high-temperature and low-pressure regime of the experimental isotherm.

**Figure 13:**
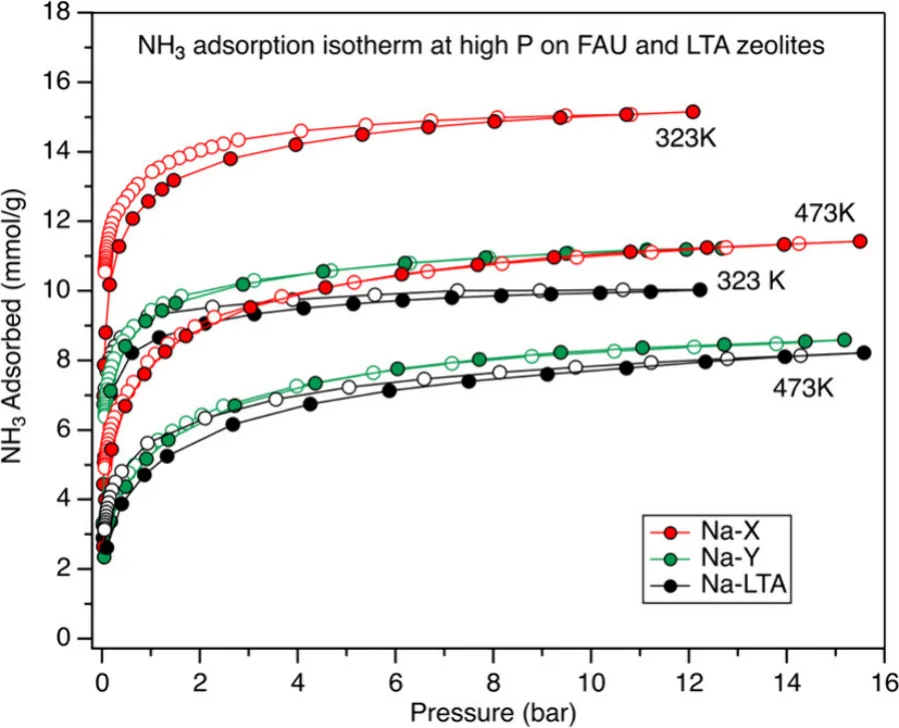
High-pressure NH_3_ adsorption and desorption isotherms of commercial Na-LTA, Na-X, and Na-Y at 323 and 473 K. The activation temperature was 673 K. Data source: ref 48.

### 2.4 NH_3_ Adsorption Selectivity via Ideal Adsorbed Solution Theory (IAST)

The literature available for adsorption uptake of mixtures including NH_3_ is very sparse. Given the lack of experimental data, the ideal adsorbed solution theory (IAST) was employed here in an attempt to use the reported unary NH_3_ adsorption data in conjunction with data from reports on the adsorption of unary N_2_ or H_2_ to estimate the selectivity potential of some porous materials for the separation of these gases. In some systems, such as in MOFs and zeolites containing cations that interact strongly with NH_3_, the widely used IAST predictions^133^ may not be accurate. However, there are no systematic studies that address the accuracy of IAST vs RAST (Real Adsorbed Solution Theory) thermodynamic predictions for NH_3_-containing mixtures. Given that the NH_3_ uptake data available in the literature is limited in terms of temperature and pressure (i.e., most are far from HB conditions), the estimation of selectivity via IAST was performed using the average molar composition of the gas reported at the exit of the HB reactors (i.e., ca. 16, 22, and 62 mol % NH_3_, N_2_, and H_2_, respectively) as the basis for the calculations. That is, for a partial pressure of 1.0 bar of NH_3_, the corresponding partial pressures for N_2_ and H_2_ will be 1.4 and 3.9, respectively. Single-component N_2_ and H_2_ uptake amounts reported for adsorbents that have also been employed for single-component NH_3_ adsorption reports are shown in Table 3.

**Table 3:** Observed Nitrogen or Hydrogen Uptake Capacities for Selected Porous Adsorbents and IAST Binary Selectivity (S_IAST_) for Selected Adsorbents and *P*_NH3_ = 1 bar, *P*_N2_ = 1.4 bar, and *P*_H2_ = 4 bar

**Material**	*T* **(K)**	*P* **(bar)**	**N**_**2**_ **Uptake** **(mmol/g)**	***S***_***NH***_**3**_/***N***_**2**__	*T* **(K)**	*P* **(bar)**	**H**_**2**_ **Uptake** **(mmol/g)**	***S***_***NH***_**3**_/***H***_**2**__	**Ref.**
Na-X	298	1.4	0.51	108	295	4.2	0.20	872	^222,223^
	323	0.7	0.05	312	-	-	-	-	^224^
	343	0.7	0.04	423	-	-	-	-	^224^
Na-Y	298	1.3	0.22	166	298	3.6	0.09	892	^225^
	338	1.5	0.11	417	-	-	-	-	^225^
	358	1.5	0.07	475	-	-	-	-	^225^
Na-LTA	303	1.4	0.40	150	298	3.8	0.21	822	^226,227^
	323	1.3	0.20	224	-	-	-	-	^226^
	343	1.5	0.19	259	-	-	-	-	^226^
Ca-LTA	303	1.4	0.69	74	303	5.3	0.11	2129	^226,228^
Clinoptilolite	298	1.4	1.23	-	298	4.2	0.05	-	^229,230^
Na-ETS-10	298	1.2	0.60	-	303	4.2	0.73	-	^231,232^
MFI (Si/Al:∞)	305	1.2	0.21	27	303	4.1	0.18	-	^233,234^
MFI (Si/Al: 20 −30)	298	1.1	0.38	25	298	4.1	0.04	893	^233,234^
ALPO_4_-11	323	1.2	0.05	788	-	-	-	-	^235^

Specific details on the equations and calculations for IAST can be found elsewhere.^236,237^ For a binary mixture, the IAST selectivity (*S*_*IAST*_) is defined as follows:

SIAST=x1x2y1y2 1

where *x*_1_ and *x*_2_ are the adsorbed phase mole fractions, and *y*_1_ and *y*_2_ are the mole fractions in the bulk phase. *S*_*IAST*_ for NH_3_/N_2_ and NH_3_/H_2_, as estimated for selected adsorbents are shown in Table 3. As expected, the selectivity for NH_3_ over the other gases is considerable, due to the stronger adsorbate–adsorbent interactions discussed above. Further, interactions at the electrostatic level between NH_3_ and porous materials such as zeolites will include specific interactions between the significant permanent quadrupole moment of the adsorbate and the electric field gradient of the surface of the adsorbent, in addition to interactions with the adsorbate permanent dipole moment. The adsorption selectivity for NH_3_ over H_2_ is greater compared to that over N_2_ because of H_2_’s smaller polarizability volume (0.81 *x* 10^–24^ cm^3^ for H_2_ vs 1.74 *x* 10^–24^ cm^3^ for N_2_, resulting in weaker dispersion interactions) and smaller permanent quadrupole moment (0.65 × 10^–26^ esu cm^2^ for H_2_ vs −1.5 × 10^–26^ esu cm^2^ for N_2_, resulting in weaker first-order electrostatic interactions).

Although the bulk of the data shown in Table 3 is limited to ambient adsorption tests that have been reported for NH_3_, the few instances of the studies that have included higher temperatures remain somewhat informative, although they are far from the traditional HB temperature range (i.e., 473–673 K). The trend of increasing selectivity with increasing temperature is unusual; a plausible explanation may be an increase in the accessibility of cations with increasing temperature.

As described in Section 2.1.1.3 (MFI Zeolites), the adsorption selectivity (defined from mole fractions in the same way as in IAST) can be computed directly from Monte Carlo adsorption simulations. Literature on the simulation of binary mixtures of NH_3_ and N_2_/H_2_ is relatively sparse and unavailable at the state points included in Table 3. For the purposes of comparison, what follows are two selectivity measurements calculated directly from simulation. In bulk MFI with Si/Al **∞**, it has been reported that at 373 K and 5 bar total pressure (*P*_NH3_ = 2.5 bar and *P*_H2/N2_ = 2.5 bar) *S*_NH3/N2_ = 8.92 and *S*_NH3/H2_ = 60.2;^110^ these values are significantly lower than those observed at ambient temperature in experiments. The simulation data at 373 K also indicate that IAST may somewhat underpredict the selectivity for the defect-free all-silica MFI material.^238^ Simulation data for selectivity of the equimolar mixtures were also reported at 523 and 623 K and *P*_total_ = 80 bar with *S*_NH3/N2_ = 4.06 and 2.95, respectively, and *S*_NH3/H2_ = 12.7 and 7.04.^110^

### 2.5 Diffusion Kinetics

Few reports in the literature have focused on studying the diffusion of NH_3_ at temperatures relevant to HB production, and those that exist are still at NH_3_ pressures far below the target. For instance, Hagelstein *et al*. studied NH_3_ microporous diffusion in Cu­(II)-Y Faujasite at 373–573 K using a dispersive extended X-ray absorption fine structure (DEXAFS) analysis and observed an average diffusivity of 2.4(±0.5) *×* 10^–9^ m^2^ s^–1^ at 0.023 bar NH_3_.^239^ Vaylon, Onyestyak, and Rees studied NH_3_ microporous diffusion in Na-A at 373 K using a frequency-response (FR) technique and obtained a diffusivity of 1.6 *×* 10^–13^ m^2^ s^–1^ at 0.019 bar NH_3_.^240^ The impact of the effective pore size of FAU and LTA is apparent in the aforementioned NH_3_ diffusivity data, with the former being advantageous for the transport of the adsorbate at low pressures. However, the same would be expected for N_2_ or H_2_, so a kinetic selectivity criterion must be considered similar to the one discussed before for pure component equilibrium uptake data (i.e., IAST). The scarcity of high-temperature kinetic data also applies to N_2_ or H_2_ and, therefore, the estimation of binary NH_3_ kinetic separation factors (i.e., *D*_*NH3*_ */D*_*i*_ where *i* is N_2_ or H_2_) from experimental data is not possible.

Molecular dynamics (MD) simulations allow for the direct calculation of diffusion coefficients from mean-square displacements (via the Einstein formalism), and the quality of the statistics improves with increasing temperature because diffusion becomes more facile. However, the vast majority of the literature covering NH_3_ in nanoporous materials utilize only Monte Carlo simulations. In one example where both MC and MD were employed, Patel *et al*.^110^ reported diffusion coefficients for NH_3_, N_2_, and H_2_ in bulk MFI and an MFI nanosheet. At 373 K and 5 bar and 623 K and 80 bar in bulk MFI, the diffusion coefficients for these species range from 0.8 to 13 × 10^–8^ m^2^ s^–1^, and diffusion selectivity favors H_2_ over NH_3_ and slightly favors N_2_ over NH_3_.

## 3 Metal–Organic Frameworks (MOFs)

Metal–Organic Frameworks are porous crystalline materials formed by combining metal ions or clusters and organic linkers.^241^ Over the last two decades, MOFs have garnered significant attention in research due to their inherent physicochemical properties and structural characteristics, such as high surface area, tunable pore size, structural diversity, and versatile modifications, making them promising candidates for selective gas adsorption, small molecule separation, heterogeneous catalysis, and diverse energy and environmental applications.^241–244^ The 2025 Nobel prize in chemistry recognized the pioneering contributions of Susumu Kitagawa, Richard Robson, and Omar Yaghi for the creation of this class of materials.

In recent years, there has been a notable surge in the study of MOFs for (a) NH_3_ adsorption and separation applications,^122,123,245–249^ (b) MOF-NH_3_ working pairs in adsorption chillers (refrigerant in air conditioning systems),^51,122,250,251^ and (c) fast-response sensors in NH_3_ detection applications.^252–256^ The design and selection of MOFs for each application depended on the physicochemical properties and stability of MOFs. For instance, NH_3_ adsorption and separation applications require MOFs with high NH_3_ uptake working capacity and selectivity over N_2_ and H_2_ gas mixtures. MOF-NH_3_ working pairs in adsorption chillers necessitate MOFs with excellent adsorption–desorption properties under ambient conditions.^145–147^ NH_3_ sensing, detection, and removal applications require strong affinity at low NH_3_ concentrations (<ppb).

MOFs consisting of various metal centers (e.g., Zr-, Cr-, Al-, and Zn-MOFs) and organic linkers have been tested for these applications with the primary focus on assessing the NH_3_ adsorption capacity under ambient conditions.^51,122,245,253,257–259^ The efficiency of a given MOF for NH_3_ adsorption is dependent on a variety of physicochemical factors. Ammonia (NH_3_), being a Lewis base, can form coordination or hydrogen-bonding interactions with Lewis acidic open metal sites or polar functional groups.^51,245^ Hence, several approaches have been employed to enhance the NH_3_ adsorption efficiency of the MOFs. This section reviews MOFs and their composite materials for NH_3_ adsorption and separation along with their selectivity for NH_3_ over N_2_ and H_2_, highlighting reversibility, stability, and regeneration conditions.

In 2008, Yaghi and co-workers reported the sorption behavior of various MOFs for NH_3_ adsorption and the capture of other harmful gases (sulfur dioxide, chlorine, tetrahydrothiophene, benzene, dichloromethane, ethylene oxide, and carbon monoxide).^260^ The study included MOFs containing Lewis acidic open metal sites (HKUST-1/Basolite C300/Cu-BTC/MOF-199), polar amine (−NH_2_), and hydroxyl (−OH) functional groups (IRMOF-3 and MOF-74 (Zn)), microporous and mesoporous structures without any specific functional groups or sites (MOF-5, MOF-177, and IRMOF-62). The NH_3_ adsorption behavior of these MOFs was evaluated using kinetic breakthrough measurements under NH_3_ (0.99%) with N_2_ as the carrier gas at room temperature. The gravimetric NH_3_ adsorption capacity of each MOF was determined, with MOF-5 (0.35 mmol/g), IRMOF-3 (6.17 mmol/g), MOF-74­(Zn) (5.46 mmol/g), MOF-177 (2.46 mmol/g), MOF-199 (5.10 mmol/g), IRMOF-62 (1.35 mmol/g), and BPL carbon (0.058 mmol/g) at 298 K. The total adsorption capacities of NH_2_-functionalized IRMOF-3 and OH- containing MOF-74­(Zn) were higher than commercial BPL carbon and MOF-5 due to the presence of hydrogen bonding and cooperative interactions between NH_3_ and functional sites in the framework.^260^ However, the predominant Lewis acidic open metal MOF, HKUST-1, exhibited low adsorption capacity and displayed irreversible chemisorption phenomena under the same conditions.

In continuation of this study, the same group later investigated the significance of Lewis acidity and the impact of an open metal center on NH_3_ adsorption by employing various Lewis acidic open metals, MOFs-74 (M) (M = Zn, Co, Ni, and Mg).^265^ Breakthrough experiments were conducted using a 4 mm sorbent bed filled with 20 mg of MOF-74; the MOFs were prehumidified in air (80% RH) at 293 K for 2 h prior to the adsorption measurements. The results showed that the breakthrough NH_3_ adsorption capacity of Mg-MOF-74 (7.62 mmol/g) and Co-MOF-74 (6.66 mmol/g) outperforms that of Zn-MOF-74 (3.70 mmol/g), Ni-MOF-74 (2.26 mmol/g), and traditional materials such as 13X Zeolite (2.86 mmol/g) and BPL activated carbon (0.17 mmol/g) under dry conditions at 298 K. However, Mg-MOF-74 and Co-MOF-74 isotherms are irreversible and contain ∼ 70% and 83% of undesorbed NH_3_, even after exposure to dry air during the desorption experiments. A similar phenomenon was observed for Cu-MOF-74, where a high uptake capacity was attributed to the irreversible chemical reaction and formation of ammonium carboxylate salt and a Cu­(OH)_2_ species by framework decomposition (Cu-MOF-74 → Cu­(OH)_2_.(NH_4_)^+^COO^–^).^266,267^

The stability of MOF-5 and MOF-177 upon NH_3_ adsorption was investigated using various analytical techniques that revealed that both MOFs exhibit minimal chemical degradation at low pressures.^268^ However, at pressure above 0.2 bar, NH_3_ molecules chemically interfere with these MOFs, deteriorating the linkage between the organic linkers and the metal center. As a result, both MOFs exhibit similar saturation uptake capacities at ∼ 1 bar (12.2 mmol/g) despite large differences in surface area and pore volumes. Furthermore, these materials after NH_3_ adsorption show a pronounced drop in BET surface area (3275 to 10 m^2^/g for MOF-5; 3275 to 4 m^2^/g for MOF-177) and loss of crystallinity (amorphization) as determined by powder-XRD patterns. FT-IR and Raman spectra indicate free-organic ligands present in each MOF. Similarly, adsorption and NMR studies reveal that HKUST-1 reacts with NH_3_ and forms a diamine-copper­(II) complex under dry conditions and Cu­(OH)_2_ and (NH_4_)_3_BTC species under humid/aqueous conditions.^269^ Addressing this topic, Kajiwara *et al*. further studied the effect of functional groups, low- and high-valent metal cations, open metal sites containing nitrogen–donor and oxygen–donor organic linkers on NH_3_ adsorption stability by examining 21 known MOFs.^257^ The stability of each MOF was assessed by comparing powder-XRD patterns before and after exposure to NH_3_. This study reveals that MOFs constructed from divalent metal cations (e.g., Cu^II^ and Zn^II^) were more reactive with NH_3_ and therefore, unstable against NH_3_ compared to high valent metal MOFs (e.g., Cr^III^ and Zr^IV^) under ambient conditions. Organic linkers containing oxygen and anionic nitrogen donor linkers exhibited improved stability than those with neutral nitrogen donor linkers in MOFs.

These early studies demonstrate that MOF-based materials exhibit a wide range of stability and adsorption performance. Since then, substantial research efforts have focused on enhancing the stability and NH_3_ adsorption capacity of MOFs by optimizing their physicochemical properties through various strategies, including: (1) MOFs containing micro- and/or mesoporous structures, (2) MOFs with Lewis acidic open metal sites, (2) incorporation of polar functional groups in MOFs, (4) synthesis of MOFs containing Bro̷nsted acidic sites, (5) MOF-based hybrid composite materials synthesis (Figure 14). These approaches involve either the direct synthesis of target MOFs or the engineering of frameworks and pores through postsynthetic modifications.^15,253,263,270,271^ Each of the above strategies possesses unique advantages. For instance, MOFs containing open metal sites within the framework exhibit enhanced NH_3_ adsorption capacity and selectivity by facilitating Lewis acid–base interactions between NH_3_ molecules and the metal sites.^253,261^ MOFs containing polar functional groups (e.g., −NH_2_, −NH–, −SO_3_H, −Cl, −OH, etc.) and Bro̷nsted acidic (e.g., −COOH, μ-OH, etc.) sites elevate pore polarity and strengthen interactions between the framework and NH_3_ through hydrogen bonding or acid–base interactions.^272–274^

**Figure 14:**
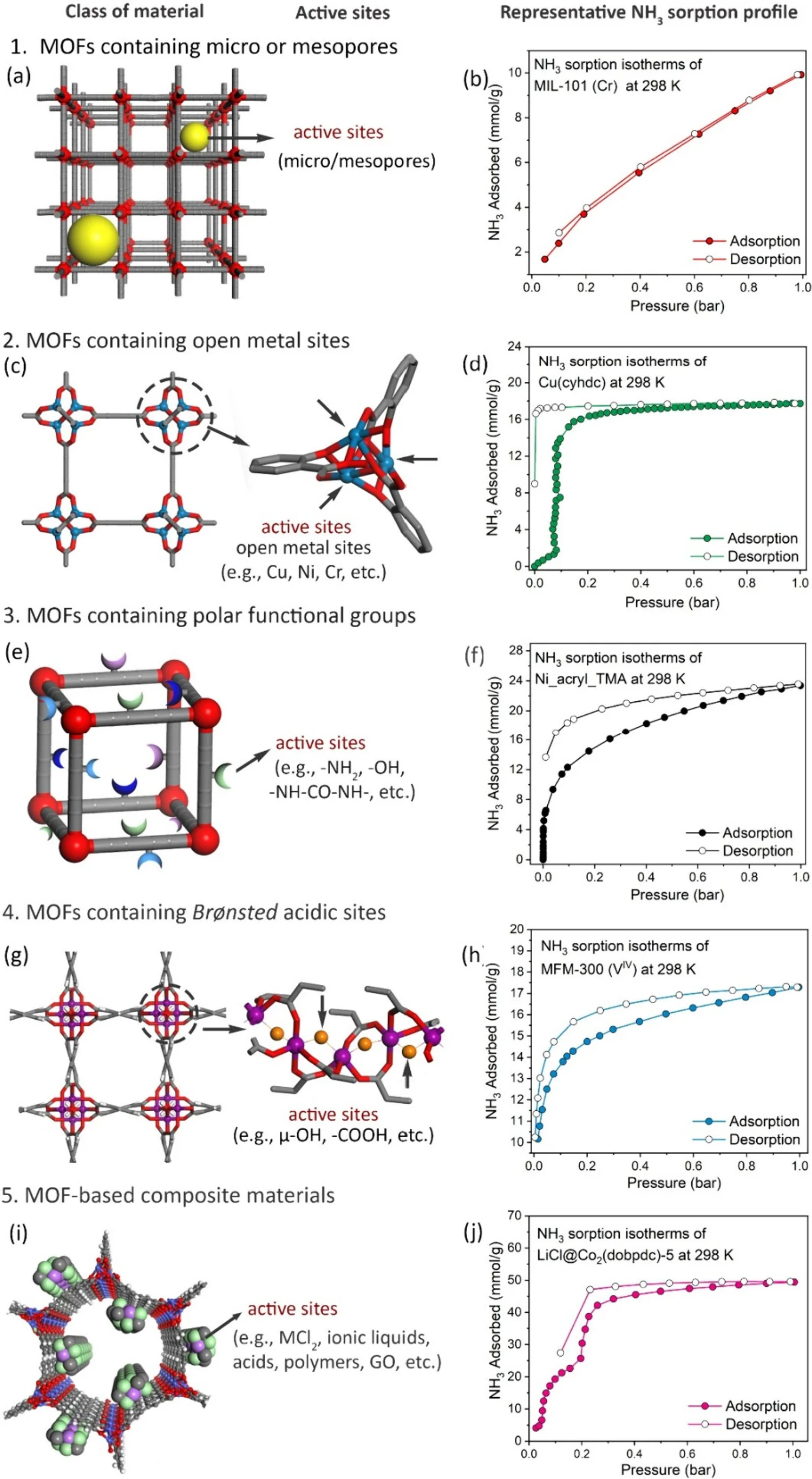
Illustration depicting MOF material design approaches for enhancing NH_3_ adsorption for separation applications: (a) Combination of micro- and mesopores in MOFs, (b) Representative NH_3_ adsorption and desorption isotherms of micro- and mesoporous MOFs showing typical desorption reversibility without hysteresis. Data source: ref 261. (c) Lewis acidic open metal or coordinatively unsaturated MOFs. (d) Representative NH_3_ adsorption and desorption isotherms of Lewis acidic open metal MOFs depicting the irreversibility in the desorption isotherm at low pressures. Data source: ref 262. (e) MOFs containing polar functional groups. (f) Representative NH_3_ adsorption and desorption isotherms of MOFs containing polar functional groups depicting irreversibility in the desorption isotherm along with hysteresis. Data source: ref 263. (g) MOFs containing Bro̷nsted acidic sites at metal centers. (h) Representative NH_3_ adsorption and desorption isotherms of MOFs containing Bro̷nsted acidic sites showing typical desorption reversibility along with hysteresis. Data source: ref 264. (i) MOF-based composite material hosting ionic liquids, metal halides, etc. (j) Representative NH_3_ adsorption and desorption isotherms of MOF-based composite materials depicting the irreversibility in the desorption isotherms at low pressures (desorption data at low pressures are not given). Data source: ref 132. Note: The reversibility of NH_3_ adsorption isotherms is highly dependent on the interactions between NH_3_ and active sites in MOFs. Weaker interacting sites enable complete adsorption–desorption reversibility, whereas stronger interactions cause irreversibility in the desorption isotherm. Typically, to achieve complete desorption requires heating of the adsorbent at temperatures higher than the adsorption temperature, often under vacuum for a long time. Closed symbols represent adsorption and open symbols represent desorption, following the convention throughout this manuscript.

### 3.1 MOFs Containing Microporous and Mesoporous Structures

Porosity in metal–organic frameworks can be tuned precisely by selecting appropriate organic building blocks and metal centers.^275,276^ Recently, micro- and mesoporous MOFs have attracted considerable attention.^277–279^

In 2018, Chen *et al*. studied the influence of pore sizes in MOFs for NH_3_ adsorption by employing two independent isostructural MOFs (MIL-101 (Cr) and MIL-100­(Cr); both MOFs also contain open metal sites.^261^ The coordination environment and connectivity of metal centers in both MOFs are the same except for the pore or cage size differences. Both MIL-100­(Cr) and MIL-101­(Cr) MOFs consist of 3D cage structures comprising small and large cages with pore sizes of 25 and 29 Å for MIL-100­(Cr) and 29 and 34 Å for MIL-101 (Figure 15a). MIL-101­(Cr) possesses ∼ 4–5 Å larger pores due to the longer benzene dicarboxylic (BDC) ligand compared to the shorter benzene tricarboxylic acid (BTC) ligand in MIL-100 (Figure 15a).^261,280^ Ammonia adsorption studies reveal that MIL-100 (Cr) and MIL-101­(Cr) exhibited 8.0 and 10.0 mmol/g of uptake capacity at 298 K, respectively (Figure 15b).^261^ Notably, both MOFs maintained adsorption–desorption reversibility under ambient conditions and retained their total adsorption capacity after five consecutive cycles, setting them apart from other MOFs that often exhibit irreversible adsorption or decomposition under similar conditions (Table 4). The smaller cage MIL–100 exhibited slightly higher NH_3_ adsorption capacity than MIL-101 at lower pressures (<0.1 bar). However, at higher pressure (>1 bar), MIL-101­(Cr) enables higher NH_3_ adsorption capacity.

**Figure 15:**
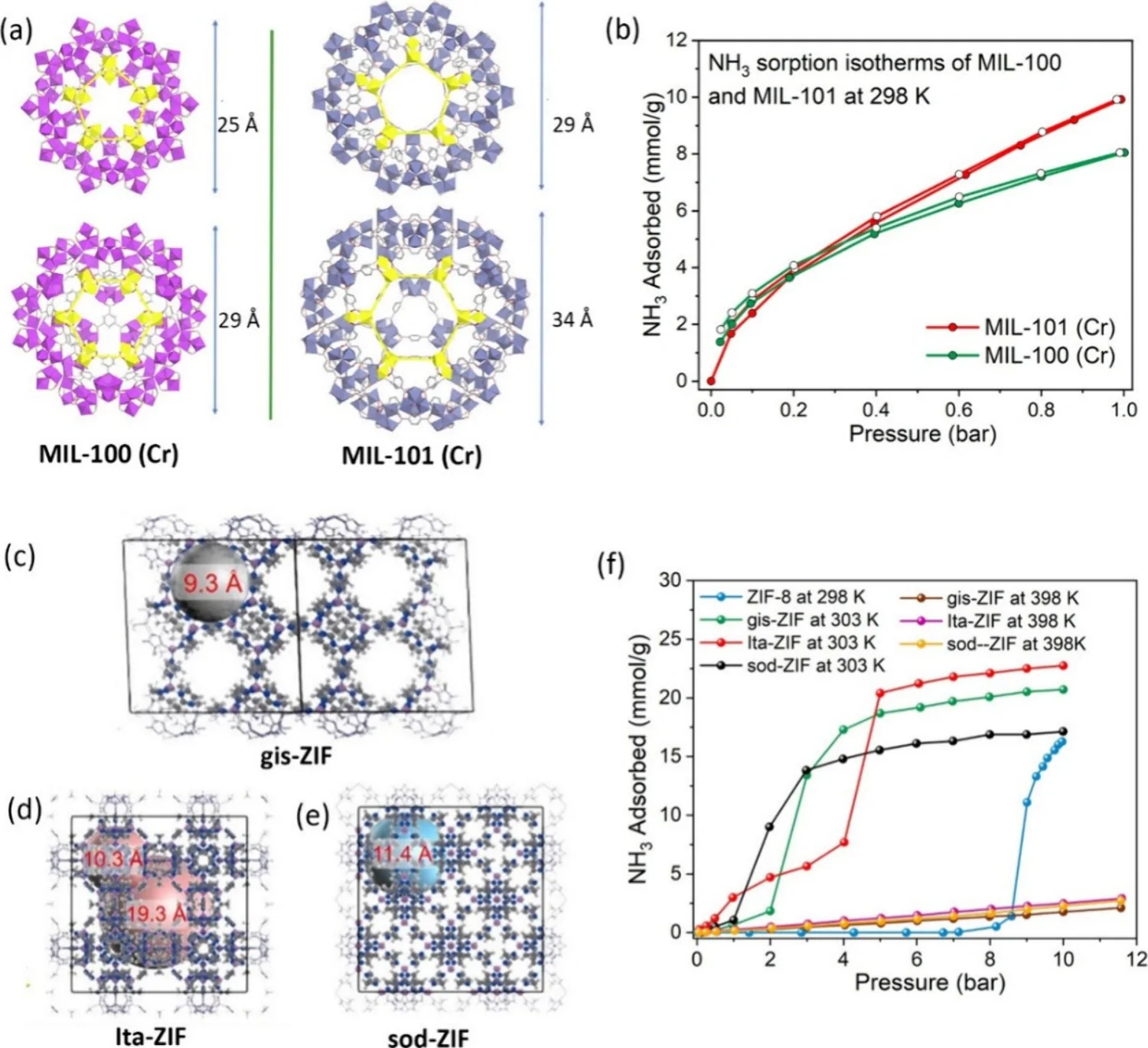
(a) Crystal structure and pore size comparisons of MIL-100 (Cr) and MIL-101­(Cr). Adopted with permission from ref 261. Copyright 2018 Elsevier. (b) NH_3_ adsorption–desorption isotherms for MIL-100 (Cr) and MIL-101 (Cr) at 298 K. Data source: ref 261. (c) Structure and pore size of gis-ZIF, (d) lta-ZIF, and (e) sod-ZIF. 15c–e adopted with permission from ref 251. Copyright 2023 American Chemical Society. (f) Simulated NH_3_ adsorption isotherms of ZIFs (gis-ZIF, lta-ZIF, and sod-ZIF) at various temperatures and pressures 1–10 bar. Data source: ref 251.

**Table 4:** Crystallographic and NH_3_ Adsorption Properties, Including Total Uptake Capacity, Selectivity, Reversibility, Regeneration Condition, and HOA of Best Performing Porous Materials (MOFs, COFs, HOFs, POPs, and Their Composite Materials) at 1 bar and 298 K^a^

**Material (active site)**	**NH**_**3**_ **Uptake** (mmol/g)	**Pore Size (Å)**	**Pore Volume (cm** ^**3**^ **/g)**	**Density (ρ)** (g/cm^3^)	**HOA/** *Q*_**st**_ **(kJ/mol)**	**Reversibility and Regeneration Condition**	**Stability**	**Sel. (IAST)**	**Ref.**
**Metal–Organic Frameworks (MOFs)**									
LiCl@Co_2_(dobpdc)-5	49.58	18.5	0.204	1.363	n.a.^b^	5 adsorption–desorption (ads–des) reversible cycles; (∼4% diminution of NH_3_ capacity after 5 cycles); regeneration at 373 K under vacuum for 6 h	PXRD stable after 5 cycles (Mn, Mg)	n.a.	^132^
LiCl@Mg_2_(dobpdc)-5	48.28								
LiCl@Ni_2_(dobpdc)-5	42.54								
LiCl@Mn_2_(dobpdc)-5 (Composite material)	37.85								
LiCl@MIL-53-(OH)_2_-43.4 (Composite material)	33.9	∼8.0	0.18	1.860	n.a.	15 adsorption–desorption reversible cycles; (∼8% diminution of NH_3_ capacity); regeneration at 373 K under vacuum for 2 h	PXRD stable after 15 cycles	3571 (NH_3_/H_2_)	^271^
MNi-Pyz (M = Co, Ni) (open metal sites/OMS)	29.1	6.8	n.a.	n.a.	n.a..	5 adsorption–desorption reversible cycles; (<5% diminution of NH_3_ capacity); regeneration at 353 K under vacuum for 2 h	stable in pH = 1–13 and boiling water	n.a.	^253^
	21.5	7.2							
[Zn_2_Cl_5_] @MIL-101(Cr) MIL-101 (ionic liquids)	24.12	∼15–25	0.375	n.a.	n.a.	5 adsorption–desorption reversible cycles; (∼5% diminution of NH_3_ capacity); regeneration at 373 K under vacuum for 12 h	PXRD stable after 5 cycles	n.a.	^286,287^
	10.0	∼20.1	1.306						
M_2_(dobpdc) (M = Mg, Ni, Zn, Co and Mn) (OMS)	23.90	18–22	1.6	0.564	90.0^c^	3 isothermal adsorption cycles and 5 breakthrough adsorption cycles under wet conditions; (∼8% diminution of NH_3_ capacity); regeneration under vacuum for next cycle	NH_4_OH vapor stable for 24 h	n.a..	^270^
	20.82		1.11	0.675	93.0^c^				
	15.24		1.07	0.708	66.6^c^				
	13.34		1.06	0.685	83.3^c^				
	13.26		1.18	0.672	79.4^c^				
CuBTC	23.88	n.a.	n.a	n.a	n.a.	n.a.	n.a.	n.a.	^15^
ZnBTC	11.33								
FeBTC (OMS)	9.5								
Ni_2_Cl_2_BTDD-arcyl-TMA	23.54	∼7, ∼ 18	0.57	0.97	n.a.	5 adsorption–desorption reversible cycles (∼2% diminution of NH_3_ capacity); regeneration under high vacuum at RT	PXRD stable to 5 cycles	n.a.	^263^
Ni_2_Cl_2_BTDD-arcyl-TGA	17.41	∼7, ∼ 18	0.56	0.89					
Ni_2_Cl_2_BTDD-acrylate	13.09	∼7, ∼ 18	0.9	0.75					
Ni_2_Cl_2_BTDD (OMS/COOH)	11.70	21.4	1.1	0.85					
MUV-102(Cu–Ti) (heterometallic Cu^2+^/Ti^4+^ OMS)	22.4	n.a.	0.33	n.a.	n.a.	9 ads-des cycles (∼37% diminution of NH_3_ capacity after 9 cycles); regeneration at 373 K under dynamic vacuum for 2 h	PXRD and SEM morphology retained after cycling	n.a.	^288^
[Cd_4_(pc1)_3_Cl_6_]·CdCl_4_·guest	22.3	7.5	n.a.	1.723	n.a.	3 adsorption (only) cycles (∼22% diminution of capacity); regeneration at 473 K under vacuum for 12 h	PXRD amorphous after NH_3_ adsorption (1st cycle)	n.a.	^289^
[C_2_N][Zn_3_Cl_7_]@Al-fum-60%	22.3	∼10–20	0.23	n.a.	n.a.	4 adsorption–desorption cycles (∼20% diminution of NH_3_ capacity)	IL leaching and partial framework decomposition	n.a..	^290^
[NC_2_N][Zn_3_Cl_7_]_2_@Al-fum-60%	21.7								
[NC_2_NC_2_N][Zn_3_Cl_7_]_3_@Al-fum-60% (ionic liquids)	18.1								
NiCl_2_(pyz)_2_ (OMS)	21.7	7.2	n.a.	n.a.	>45	5 ads–des cycles (∼22% diminution of capacity); regeneration via acetonitrile solvent treatment at 373 K for ∼ 20 min	PXRD stable to 5 cycles	>15 (NH_3_/N_2_)	^291^
Cu@Th-BPYDC	20.55	7.0	n.a.	n.a..	n.a.	Irreversible NH_3_ adsorption (no desorption); regeneration at 373 K under dynamic vacuum for 6 h	PXRD stable after NH_3_ adsorption	n.a.	^292^
Th-BPYDC	5.90	10.0							
M_2_Cl_2_BBTA (M = Cu, Co, Ni) (OMS/azolate ligand)	19.79	13.0	0.5	1.138	n.a.	3 adsorption–desorption reversible cycles (∼6% diminution of NH_3_ capacity (Co-MOF)); regeneration at 473 K under dynamic vacuum	Ni-MOF only retains its crystallinity and porosity. Cu- and Co-MOFs unstable	n.a.	^293^
	17.95	13.0							
	14.68	13.0							
MOF-303(Al) (Bro̷nsted acidic −OH sites)	19.7	5.3	1.48	1.064	n.a.	20 adsorption–desorption cycles (<2% diminution of NH_3_ capacity); regeneration at 423 K under vacuum	PXRD broadens and decreases in BET surface area and crystallinity after NH_3_ ads–des	n.a.	^294^
MOF-303	18.5^f^	5.5–6.5	n.a.	n.a.	∼39–45	>95% retention over 3 cycles; regeneration at 323 K under vacuum for 12 h	Partial structural degradation and BET lost	n.a.	^295^
CAU-23	17.4^f^				∼34				
KMF-1	16.6^f^				∼39–45				
MIL-160	14.4^f^				∼39–45				
MOF-303(Al)	18.1	4.3	0.63	n.a.	n.a.	10 adsorption cycles only (∼7% diminution of NH_3_ capacity (rGO@MOF-303(Al)-5%)); regeneration at 393 K under vacuum for 8 h	Reduction in the characteristic PXRD intensity after exposing to hot water at 373 K for 24 h	n.a.	^296^
rGO@MOF-303(Al)-5%,	17.5	4.2	0.50						
–8%, −15%.	17.0	4.1	0.52						
(composite materials)	16.8	4.3	0.51						
Fe-MIL-101-SO_3_H (OMS)	18.0	∼26–35	n.a.	n.a.	n.a.	Irreversible	n.a.	n.a.	^297^
						Regeneration at 398 K for 24 h			
MOF-253(Al)-NiCl_2_-2	18.0	6.5	0.42	n.a.	45.8–48.1	6 adsorption–desorption reversible cycles (6% diminution of NH_3_ capacity); regeneration at 403 K under vacuum	significantly lost its crystallinity after 6 ads–des cycles	28513 (NH_3_/H_2_) 33132 (NH_3_/N_2_)	
MOF-253(Al)-CoCl_2_-2	16.3	6.4	0.10		N.A.				
MOF-253(Al)-SnCl_2_-2	10.4	6.9	0.23		N.A.				
MOF-253(Al) (composite materials)	5.5	6.9	0.52		30.3–31.2				
3D-[Zn_2_(L1)_2_(bpe)]	17.8	4.5–6.5, ∼ 8.0	0.654	0.84	n.a.	Desorption is not completely reversible	Not stable and collapsed	n.a.	^298^
3D-[Zn_2_(L1)_2_(bipy)] (Free urea groups)	14.3	4.5–6.5, ∼ 8.0	0.458	0.97		Regeneration at 313 K under vacuum for 4 h			
MFU-4	17.7	12.3	n.a.	n.a.	n.a.	Desorption is not completely reversible	n.a.	n.a.	^299,300^
MFU-4l	13.0	15.0							
MFU-4l-(OH)	9.0	15.0							
M(NA)_2_ (M = Co, Cu, Zn, Cd) (OMS)	17.5	3.6	0.5	n.a.	n.a..	3 adsorption–desorption reversible cycles but the desorption is not completely reversible; (<1% diminution of NH_3_ adsorption capacity); regeneration at 423 K under dynamic vacuum	Uptakes retained for three cycles, but PXRD crystallinity lost	n.a.	^301^
	13.4	3.6	0.5						
	10.2	3.6	0.5						
	6.0	3.6	0.5						
M(cyhdc) (M = Cu, Fe, Cr) (OMS)	17.5	n.a.	n.a.	n.a.	∼25–35	3 adsorption–desorption TGA reversible cycles (largely retained its NH_3_ capacity) TSA between 373–458 K at 0.75 mbar and PSA between 1.2 and 0.05 bar at 423 K; regeneration at 433 K under dynamic vacuum	Reversable structural changes upon NH_3_ adsorption–desorption	NH_3_ selective over H_2_ and N_2_	^262^
	17.0								
	6.2								
MFM-300(M) (M = V^IV^, Fe, Al, V^III^, Cr) (bridging hydroxyl), (data at 273 K)	17.3	6.7	0.48	1.333	30–60	20 adsorption–desorption reversible cycles; (<3% diminution of NH_3_ capacity); regeneration at 373–473 K under dynamic vacuum	Hysteresis loop and Residual NH_3_ content is ∼ 9%, after NH_3_	n.a.	^264^
	16.1		0.46	1.342	35–40				
	15.7		0.37	1.040	30–50				
	15.6		0.49	1.154	35–45				
	14.0		0.47	1.130	35–65				
M(BDC) M = Cu, Zn, Cd (OMS)	17.2	∼5.7	n.a.	n.a.	n.a.	3 adsorption–desorption reversible cycles; regeneration at 523 K under vacuum but the desorption is not completely reversible; (<1% diminution of NH_3_ adsorption capacity)	PXRD unstable and structural transformation observed after NH_3_ adsorption	n.a.	^258^
	14.1	∼5.4							
	7.4	∼6.2							
UiO-66-Cu^II^	16.9	∼6.0	0.388	n.a.	Up to 55	15 adsorption–desorption reversible cycles between 0 and 0.15 bar (largely retained its capacity); regeneration under vacuum	PXRD stable to 15 cycles	n.a.	^302^
UiO-66-Cu^I^	11.8	7.7, 12 (cage)	0.388		40				
UiO-66-defect (OMS)	12.6^d^		0.388		35				
OH-DUT-6	16.4	23,16,13	2.01	0.391	n.a..	Not completely reversible; regeneration under supercritical CO_2_ for 5 h	Lost crystallinity after NH_3_ adsorption	n.a.	^303^
DUT-6	12.0	∼13	2.02	0.485					
CAU-1-NH_2_	16.5	∼5–11	0.55	n.a.	47.6–32	Reversible for 10 adsorption–desorption cycles	Retained PXRD and BET surface area after 10 cycles	1168 (NH_3_/N_2_)	^304^
CAU-1-OH	16.3		0.49		41.2–25	Regeneration at 393 K for 5 h under vacuum (0.005 mbar)			
PA-PEI-MOF-303(Al)	16.07	∼5–6	0.28	n.a.	n.a.	10 breakthrough cycles; regeneration at 423 K for 2 h under vacuum	PXRD stable after NH_3_ ads–des cycles	n.a.	^305^
M_2_Cl_2_BTDD (M = Mn, Co, Ni) (OMS)	15.47	21.4	1.37	0.695	60–120	3 adsorption–desorption reversible cycles (∼3% diminution of NH_3_ capacity); regeneration at 150 K under dynamic vacuum	BET Surface area decreased after NH_3_ adsorption	n.a.	^306^
	12.02	21.4	1.36	n.a.	40–70				
	12.00	21.4	n.a.	n.a.	∼40–45				
UiO-67-bpy-Mn (PSF-Mn)	14.5	12–16	0.19	n.a.	n.a.	5 adsorption–desorption cycles (∼3% diminution of NH_3_ capacity); regeneration at 393 K under vacuum for 12 h	PXRD stable after NH_3_ ads–des	n.a.	^307^
UiO-67-bpy-CO	14.0								
UiO-67-bpy-Ni	13.5								
MFM-300(Al)	13.9	7.5	0.37	1.01^e^	>40	50 adsorption–desorption reversible cycles (293 K); (<1% diminution of NH_3_ capacity); regeneration at 473 K under vacuum for 24 h	PXRD stable to 50 cycles	NH_3_ selective over N_2_	^308^
UiO-66-[ImAc]Cl/UiO-66–1	13.3	5.1	5.1	n.a.	32–36	30 adsorption–desorption reversible cycles (∼4% diminution of NH_3_ capacity); regeneration at 393 K under vacuum	PXRD stable and BET surface are retained after NH_3_ ads–des cycles	n.a.	^309,310^
UiO-66-[Hbet]Cl/UiO-66–2	11.6	5.4	4.6		25–36				
UiO-66-PyAc]Cl/UiO-66–3	10.5	5.4	3.1		23–30				
UiO-66	8.3	5.1	5.7		20–23				
UiO-66–2.5	12.6	14.1	0.514		∼66				
MFM-300(Sc)	13.1	8.1	0.56	n.a.	48.3	5 adsorption–desorption cycles (3.8% diminution of NH_3_ capacity)	PXRD stable after NH_3_ ads/des	n.a.	^311^
H-RhMOP	12.6	n.a.	n.a.	n.a.	n.a.	5 adsorption–desorption cycles (∼6% diminution of NH_3_ capacity)	Acidic water stable	n.a.	^312^
						Regeneration at 403 K under vacuum for 12 h			
MOF-5	12.2	∼8.0	1.39	0.767	n.a.	No reversibility and structural degradation	Not stable/collapses in NH_3_	n.a.	^268,274^
MOF-177	12.2	11.8	2.65	0.31	n.a.				
HKUST-1 (OMS)	12.1	6.0	0.333	0.88	50–60				
[M_2_(adc)_2_(dabco)] M = Ni, Co, Zn, Cu (OMS)	12.1	n.a.	0.25	n.a.	18	3 adsorption–desorption cycles (stable cyclic performance for three cycles); regeneration at 393 K under vacuum for 12 h	stable after NH_3_ ads/des	n.a.	^313^
	11.2		0.26						
	8.3		0.23						
	6.5		0.25						
HKUST-1(OMS)	12.1^d^	6.0	0.333	0.879	50–60	No reversibility	Not stable/collapses in NH_3_	n.a.	^274^
						Regeneration at 393 K for 6 h			
UiO-66-NH_3_Cl	12.0	8–11	n.a.	n.a.	n.a.	Irreversible	n.a.	n.a.	^297^
UiO-66-NH_2_	10.3					Regeneration at 373 K 24 h			
[CAM][Cl]@MIL-101(Cr)-30%	11.61	∼20.1	0.83	n.a.	n.a.	5 adsorption–desorption reversible cycles; (stable performance for five cycles)	PXRD stable to 5 NH_3_ ads–des cycles	3266 (NH_3_/CO_2_)	^314^
Ga-PMOF	10.5	10	0.48	n.a.	n.a.	2 consecutive NH_3_ sorption cycles (∼27% diminution of NH_3_ capacity)	Diminish of PXRD crystallinity	n.a.	^315^
In-PMOF	9.41								
Al-PMOF	7.67								
PFC-27/CF_3_SO_3_-	10.44	12 ∼ 20	n.a.	∼1.173	10.16	5 adsorption–desorption reversible cycles (stable cyclic performance for five cycles); regeneration at 393 K under vacuum for 12 h	PXRD stable after NH_3_ ads–des	n.a.	^316^
PFC-27/CF_3_COO-	8.92			∼1.173	9.64				
PFC-27/CH_3_COO-	8.44			∼1.173	8.99				
PFC-27	4.58			∼1.173	7.03				
Othe famous MOFs: ZIF-8, NU-300, MIL-53, MIL-100, MIL-101, Al-MIL-101-NH_2,_ MOF-199, UiO-66 and etc	<10	-	-	-	-	Low NH_3_ adsorption capacity and largely Reduced the capacity after 1st cycle; regeneration at >373 K under vacuum for 12 h	-	-	^249,257,261,317–333^
**Covalent Organic Frameworks (COFs)**									
LiCl@XJCOF-4–44.1 (composite materials)	44.98	16.1	n.a.	n.a.	n.a.	6 adsorption–desorption reversible cycles; (∼2% diminution of NH_3_ capacity); regeneration at 353 K under vacuum	Boiling H_2_O and 3 M HCL stable.	NH_3_ selective over H_2_ and N_2_ (breakthrough)	^131^
NiCl_2_@XJCOF-2	30.3	17	0.07	n.a.	43.6–105.1	12 cycles ∼ 75% capacity retained after initial activation cycles; regeneration at 423 K under vacuum for 12 h	PXRD unchanged after cycling	3.39 × 10^7^ (NH_3_/N_2_) 4.67 × 10^7^ (NH_3_/H_2_)	^334^
CaCl_2_@COF-34% (composite materials)	26.5	34.0	0.654	n.a.	n.a.	7 adsorption–desorption reversible cycles (∼8% diminution of NH_3_ capacity); regeneration at 353 K under vacuum for 2 h	PXRD stable to NH_3_ adsorption–desorption cycles	n.a.	^47^
[SrOOC]_17_-COF	14.30	∼15	n.a.	n.a	91.2	5 adsorption–desorption reversible cycles (∼4% diminution of NH_3_ capacity); regeneration at 474 K under vacuum for 12 h	Retains the PXRD crystallinity after NH_3_ ads–des	n.a.	^335^
[CaOOC]_17_-COF	12.25	∼15							
[MnOOC]_17_-COF	11.38	∼15							
TPPa-1/[HOOC]x-COF x = 0	9.23	∼15							
[HOOC]_100_-COF (composite materials)	4.14	9.7							
COF-10	11.9	34.0	0.82	n.a.	n.a.	3 adsorption–desorption reversible cycles; (∼3% diminution of NH_3_ capacity); regeneration at 473 K	Retains the crystallinity of PXRD but lost surface area and N_2_ capacity 2nd cycle	n.a.	^127^
COF-102	12.1	10–12							
COF-6	∼8.5								
TpBD-(SO_3_H)_2_	11.2	12.9	n.a.	n.a.	n.a.	5 adsorption–desorption reversible cycles (∼20% diminution of NH_3_ capacity)	Retains the PXRD crystallinity after NH_3_ adsorption	n.a.	^336^
Cis-COF-HNU38	7.7	20, 53	n.a.	n.a.	20–22	5 adsorption–desorption reversible cycles	Retains the PXRD crystallinity after NH_3_ adsorption	158(NH_3_/N_2_)	^337^
Trans-COF-HNU38	6.0								
FB-TAPB COF	4.7								
**Hydrogen Organic Frameworks (HOFs)**									
HOF-102	11.16	25, 30	1.28	n.a.	n.a.	n.a.	n.a.	n.a.	^338^
FDU-HOF-3	9.34	4.8	n.a.	1.335	29	10 adsorption–desorption reversible cycles	lost their crystalline states when NH_3_ entered the HOFs. PXRD retained after NH_3_ desorption)	n.a.	^339^
HOF-101	8.44	18, 24	1.1			Regeneration at 323–363 K under vacuum for 24 h			
KUF-1a	6.67	n.a.	n.a.	1.258	n.a.	5 adsorption–desorption reversible cycles; regeneration at 393 K under vacuum at room temperature or under vacuum for 12 h	PXRD patterns changed after NH_3_ adsorption (PXRD retained after NH_3_ desorption)	NH_3_ selective over N_2_, and H_2_	^129^
**Porous Organic Frameworks (POFs)**									
PAA–PVIm	19.3	20–100	n.a.	n.a.	n.a.	8 adsorption–desorption reversible cycles; regeneration at 353 K under vacuum	Stable to NH_3_ adsorption–desorption	1068(NH_3_/N_2_)	^340^
PVBA–P4VP	18.4							254(NH_3_/N_2_)	
PAA-P4VP	18.2							408(NH_3_/N_2_)	
PVBA–PVIm	15.8							331(NH_3_/N_2_)	
PAA	6.0							n.a.	
P1-PO_3_H_2_	18.7	∼11	0.49	n.a.	n.a.	4 adsorption–desorption reversible cycles at 1 mbar (∼5% diminution of NH_3_ capacity, P2-COOH (∼3.6 mmol/g); regeneration at 403 K under vacuum for 12 h	Stable under humid conditions	n.a.	^128^
P2-NH_3_Cl	16.3	∼6–10	0.45						
P2-SO_3_H (Bro̷nsted acidic sites)	13.1	∼6–10	0.20						
BPP-5	17.7	<6.0	n.a.	n.a.	n.a.	Regeneration at 403 K under vacuum for 12 h between each cycle	Stable to NH_3_ adsorption–desorption	n.a.	^128,297^
BPP-7(P_2_–CO_2_H)	16.1	∼5–11	0.30						
PPN-6-SO_3_H(P1-SO_3_H)	12.1	∼5–14	0.64						
BPP-2(P1-NH_3_Cl)	11.2	∼5–12	0.53						
BPP-1	6.2	∼5–15	0.50						
PAF-1 (Bro̷nsted acidic −OH sites)	3.0	14.1	n.a.						
SOMPs (OMP-SO_3_H-220) Bro̷nsted acidic −OH sites	15.09	∼6.3, 11.7, 136	0.63	∼0.33–0.37	45–65	30 adsorption–desorption reversible cycles; regeneration at 353 K under vacuum for 6 h	Stable to NH_3_ ads/des	>100 (NH_3_/N_2_) IAST	^341^
1	14.0	∼8–10	n.a.	n.a.	n.a.	5 adsorption–desorption reversible cycles (breakthrough); regeneration at 393 K under vacuum for 10h	Stable to NH_3_ ads-des	n.a.	^342^
1S	12.9	∼8–10							
1E	14.0	∼8–10							
1ES	12.1	∼8–10							
PAA	10.7	5–6	n.a.	n.a.	n.a.	5 adsorption–desorption cycles at 1 mbar	Stable to NH_3_ adsorption–desorption	n.a.	^343^
PI	9.0	9.0				Regeneration at 353 K under vacuum 4–18 h			
MPOP-1.0-SO_3_H	10.96	61.2	0.51	n.a.	n.a.	10 adsorption–desorption reversible cycles; regenerated by purging with N_2_ at 433 K	Stable to NH_3_ adsorption–desorption	1196 (NH_3_/N_2_)	^344^
MPOP-0.5-SO_3_ Bro̷nsted acidic −OH sites	7.62	62.4	0.82					2524(NH_3_/H_2_)	
Other POPs NU-POP-1, A_2_B_1_, ZnA_2_B_1_, CuA_2_B_1_, P(DVB-VPA)-4.0, PTS-1, PIPX	<10	-	-	-	-	-	-	-	^345–348^
**Hyper-Cross-Linked Polymers (HCPs)**									
TCS@PDMS10	8.52	∼5, 15	n.a.	n.a.	n.a.	10 adsorption–desorption reversible cycles; regeneration at 393 K under vacuum	n.a.	n.a.	^349^
1TCS	8.52	∼5, 15			120–60				
1TC	6.41	∼4–10			45				
1T	3.8	∼4–10			∼30				
HCP-BPA-Na	7.2	63.3	1.41	n.a.	n.a.	-	n.a.	n.a.	^350^
HCP	7.2	63.3	1.41	n.a.	n.a.	10 adsorption–desorption cycles. Regeneration at 353 K under N_2_ flow	Stable to NH_3_ adsorption–desorption	n.a.	
LiCl-HCP-5.35	34.49								
Li–Cl–HCP-2.28	23.86								
LiNTf2-HCP-2.28	12.46								
LiBr-HCP-2.28	21.8								
HCP-1	4.85	30–70	1.85	n.a.	n.a.	10 adsorption–desorption cycles; regeneration at 353 K under N_2_ flow	Stable to NH_3_ adsorption–desorption	n.a.	^351^
HCP-2	3.74		0.75						
HCP-3	2.83		1.37						
sHCP-1	11.54		0.93						
sHCP-2	8.05		0.48						
sHCP-3	10.38		0.79						
**Other Materials**									
ZnIL@FDU-12–60	25.2	∼38, 300	0.1	n.a.	n.a.	8 adsorption–desorption reversible cycles (∼20% capacity drop)	Stable to NH_3_ adsorption–desorption cycles	n.a.	^352^
						Regeneration at 323 K under vacuum for 6 h			
PVIm-Co(II)-PVBA	23.7	20–600	n.a.	n.a.	n.a.	10 adsorption–desorption reversible cycles; (∼3% capacity drop) desorption at 353 K vacuum	Stable under moisture and NH_3_ adsorption–desorption	93 (NH_3_/N_2_)	^353^
PVIm-Cu(II)-PVBA	20.9							187 (NH_3_/N_2_)	
PVIm-Ni(II)-PVBA	20.8							127 (NH_3_/N_2_)	
BiI_3_ nano sheets	22.6	n.a.	n.a.	n.a.	163	10 adsorption–desorption reversible cycles. (<1% capacity drop); regeneration at 353 K under vacuum for 2 h	Stable to NH_3_ ads-des cycles	NH_3_ selective over CO_2_	^354^
CoHCC	21.9	n.a.	n.a	n.a.	n.a.	4 adsorption cycles; regeneration at 423 K under vacuum for 24 h	Stable to NH_3_ adsorption	n.a.	^130,355^
CuHCC	20.2								
Prussian blue	12.5								
NiHCF									
S-AMNP	17.0	6–12	0.35	n.a.	n.a.	n.a.	Stable to NH_3_ adsorption	n.a.	^356^
L-AMNP	12.6	6–12	0.60						
H-AMNP	11.6	6–12	0.36						
[Ph_3_ImH][Tf_2_N]_2_	15.65	20–300	0.177	n.a.	n.a.	10 adsorption–desorption cycles	PXRD changes were observed after NH_3_ adsorption	438 (NH_3_/N_2_)	^357^
[PhImH][Tf_2_N]	10.0					Regeneration at 353 K under vacuum (<1 mbar)			
SPF-Naresin	12.87	n.a.	n.a.	n.a.	n.a.	20 adsorption–desorption reversible cycles; regeneration at 353 K under cacuum for 2 h	Stable to NH_3_ adsorption	5076(NH_3_/N_2_)	^358^
								2619(NH_3_/H_2_)	
MOS-1	11.5	5–6	n.a.	n.a.	37.1	5 adsorption–desorption reversible cycles; regeneration under vacuum	Stable to NH_3_ adsorption	n.a.	^359^
MOS-2	5.2	3–9			43.6				
MOS-3	3.8	3–6			45.6				
Amberlyst 15 (Rohm & Haas Co)	11.34	400	0.3–0.4	n.a.	n.a.	Regeneration at 388 K under vacuum >1 h	Commercial sorbents	181(NH_3_/N_2_)	^163^
								201(NH_3_/H_2_)	
13X zeolite (Na) (Lancaster 6149)	9.3	8.5	0.34	∼0.682	n.a.	Regeneration at 573 K under vacuum (<1 mbar)	Commercial sorbents	n.a.	^163^
Activated carbon. (Merck 1.09624)	5.08	n.a.	n.a.	∼0.43	n.a.	Regeneration at >423 K under vacuum (<1 mbar)	Commercial sorbents	n.a.	^163^

a Reversibility and regeneration, stability, selectivity, and all other data are given for the best class of NH_3_ adsorption materials in each subsection.

b n.a.: not available/not discussed in the respective articles.

c HOA calculated using computation data.

d 273 K;

e The density of the structure, from which iodine was removed from the pores, was obtained from the CIF file.

f 303 K, 7 bar.

In 2023, Liu *et al*. investigated the adsorption performance of various pore sizes of ZIF materials using GCMC simulations (Figure 15c–f).^251^ The structures and pore sizes reveal that Ita-ZIF has two types of pores (10.3 and 19.3 Å), while gis-ZIF (9.3 Å) and sod-ZIF (11.4 Å) have uniform pores in the structures (Figure 15c–e). Ammonia adsorption simulations were performed at various temperatures and pressures (1 to 10 bar). Among simulated ZIFs, lta-ZIF exhibited 22.8 mmol/g of NH_3_ adsorption capacity at 303 K, 10 bar (Figure 15f). Furthermore, Figure 15f highlights the difference in the onset pressure of the ZIF-8 or sod-ZIF at ambient temperature, due to the distinction in the ZIF force field.^251,281^ However, experimental isotherms of ZIF-8 indicate very low adsorption capacity under ambient conditions raising questions regarding ZIF stability during NH_3_ adsorption.^257,282^

### 3.2 MOFs Containing Lewis Acidic Open Metal Sites

The concept of open metal sites or coordinatively unsaturated metal centers in MOFs was first introduced in 1998.^283^ Since then, significant advancements have led to the development of high-performance open metal site MOFs such as HKUST-1, M-MOF-74, M_2_Cl_2_BBTA, M_2_(dobpdc) (M = Mg^2+^, Ni^2+^, Co^2+^, Zn^2+^), etc., for various applications (Table 4).^283–285^ These open metal sites act as Lewis acids, which are generated by removing weakly coordinated solvent molecules or ligands in the metal centers of MOFs through activation at higher temperatures or solvent exchange methods. MOFs with open metal sites often exhibit enhanced porosity and a strong affinity for guest molecules due to strong Lewis acid–base interactions.

In 2016, Rieth *et al*. reported a series of mesoporous azolate MOFs containing coordinatively unsaturated metal sites (Mn, Co, and Ni).^306^ These MOFs were synthesized by using an extended bisbenzotriazole ligand, bis­(1H-1,2,3-triazolo­[4,5-*b*],[4′,5′-i])­dibenzo-[1,4]­dioxin (H_2_BTDD), and various metal chloride salts (Mn, Co, and Ni) at 338 K, resulting in the respective MOFs, M_2_Cl_2_(BTDD)­(H_2_O)_2_ (M = Mn (1); Co (2); Ni (3)). The single crystal X-ray structure of Mn_2_Cl_2_(BTDD)­(H_2_O)_2_ reveals a 3D honeycomb network along the *c*-axis consisting of 2.2 nm mesopores (Figure 16a). In the crystal structure, each Mn ion is coordinated with three nitrogen atoms of three independent BTDD^2–^ linkers, with chloride bridging between two metal atoms and a terminal labile water molecule pointing toward pores (removing these water molecules creates open metal sites). Analogous structures for 2 and 3 are obtained as microcrystalline powders, and their powder patterns match well with the Mn_2_Cl_2_(BTDD)­(H_2_O)_2_ (1) structure. Activation of as-made M_2_Cl_2_(BTDD)­(H_2_O)_2_ at 373 K creates open metals sites by loss of coordinated water molecules (Figure 16a and 16b).

**Figure 16:**
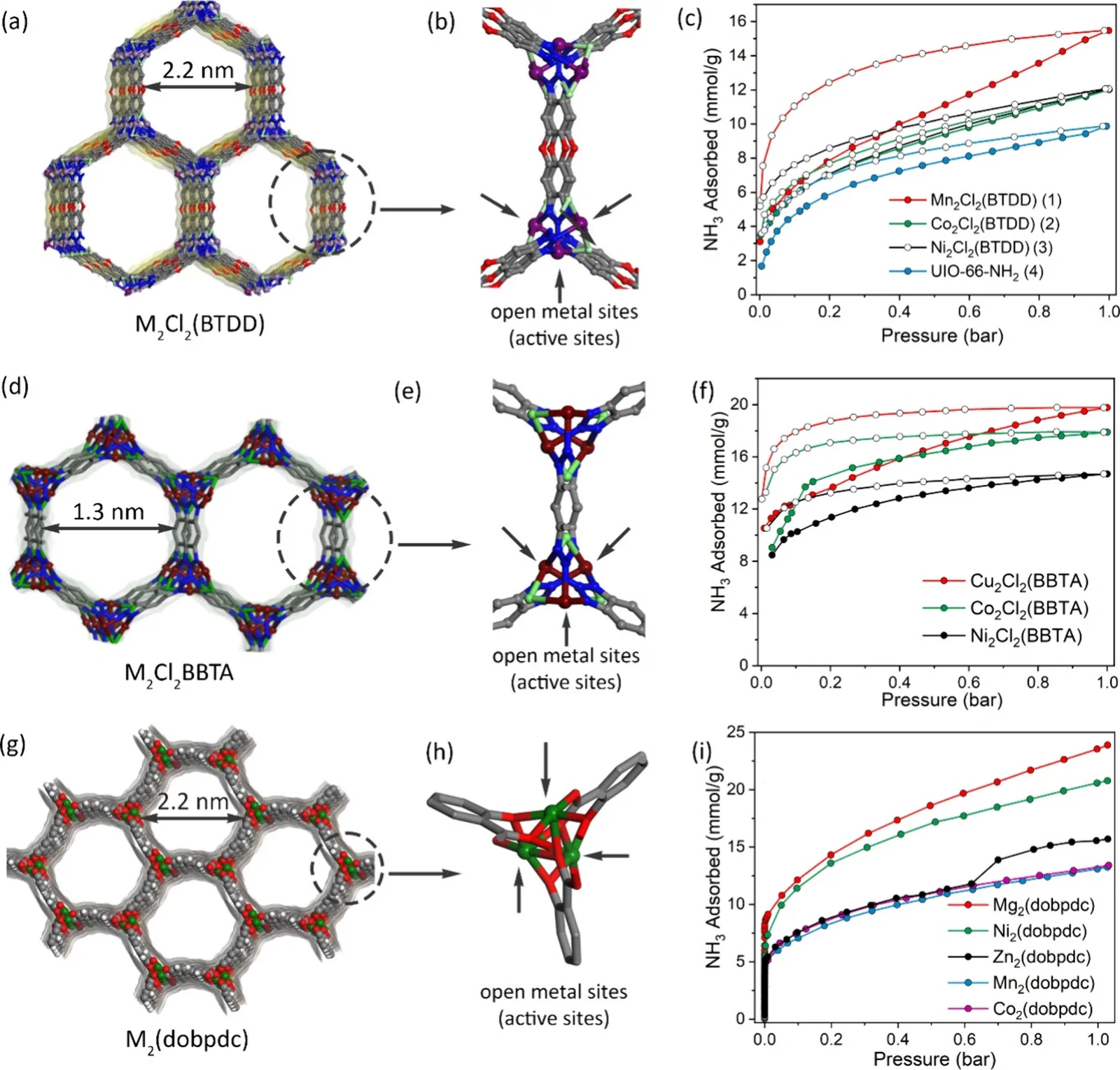
(a) Single-crystal X-ray structure of M_2_Cl_2_(BTDD) shows the ∼ 2.2 nm honeycomb network along the *c*-axis. (b) Lewis acidic open metal centers of M_2_Cl_2_(BTDD). (c) NH_3_ adsorption–desorption isotherms of Mn_2_Cl_2_(BTDD) (1), Co_2_Cl_2_(BTDD) (2), Ni_2_Cl_2_(BTDD) (3) and UiO-66-NH_2_ (4). Data source: ref 306. (d) Single-crystal X-ray structure of M_2_Cl_2_(BBTA) shows the ∼ 1.3 nm honeycomb network along the *c*-axis. Data source: ref 293. (e) Lewis acidic open metal centers of M_2_Cl_2_(BBTA). (f) NH_3_ adsorption–desorption isotherms of Cu_2_Cl_2_(BBTA), Co_2_Cl_2_(BBTA), and Ni_2_Cl_2_(BBTA). Data source: ref 293. (g) Single-crystal structure of M_2_(dobpdc) shows 1D 2.2 nm pores. (h) Lewis acidic open metal centers of M_2_(dobpdc). (i) NH_3_ adsorption isotherms of M_2_(dobpdc) (M = Mg^2+^, Mn^2+^, Co^2+^, Ni^2+^, and Zn^2+^) at 298 K. Data source: ref 270.

At 298 K, the ammonia uptake capacities of M_2_Cl_2_(BTDD) were found to be 15.47, 12.00, and 12.02 mmol/g for 1, 2, and 3, respectively (Figure 16c). Additionally, the ammonia adsorption isotherms at 293 and 283 K show capacities of 17.86 and 22.43 mmol/g for Mn_2_Cl_2_(BTDD); 12.36 and 16.24 mmol/g for Co_2_Cl_2_(BTDD); and 12.37 and 16.58 mmol/g for Ni_2_Cl_2_(BTDD) at 1 bar. Notably, these MOFs retained 97% of their initial capacity over three consecutive cycles after being activated at 423 K. In comparison, UiO-66-NH_2_ dropped more than 50% in its uptake capacity after the first cycle (9.84 mmol/g) under the same conditions. However, the BET surface area and powder XRD crystallinity of post-NH_3_ adsorption tested samples showed a reduction of peak intensity and surface area for the Mn_2_Cl_2_(BTDD), while Co_2_Cl_2_(BTDD) and Ni_2_Cl_2_(BTDD) remain unchanged.

Subsequently, in 2018, the same group further investigated the combined effect of pore size and open metal site densities and their cooperative interactions on NH_3_ adsorption performance and adsorption kinetics by employing smaller-pore open metal MOFs, M_2_Cl_2_BBTA (M = Co, Ni, Cu; BBTA = bis­(1H-1,2,3-triazolo­[4,5-*b*],[4′,5′-i])­dibenzo­[1,4]­dioxin).^293^ The crystal structure and coordination environment of M_2_Cl_2_BBTA (pore size ∼ 1.3 nm) is analogous to M_2_Cl_2_BTDD (pore size ∼ 2.2 nm) with only pore size differences (Figure 16a and 16d).^293^ Ammonia adsorption studies demonstrated that the total uptake capacities of Co_2_Cl_2_BBTA, Ni_2_Cl_2_BBTA, and Cu_2_Cl_2_BBTA were 7.95, 14.68, and 19.79 mmol/g, respectively, at 298 K and 1 bar (Figure 16e). Analogous large-pore M_2_Cl_2_BTDD has 15.47, 12.00, and 12.02 mmol/g of NH_3_ capacity for the Mn, Co, and Ni-MOFs under the same conditions.^293,306^

Furthermore, NH_3_ breakthrough (1000 ppm of NH_3_ feed) measurements of microporous Co_2_Cl_2_BBTA displayed an adsorption capacity of 8.56 mmol/g at 0% RH, which is 27% higher than the larger pore Co_2_Cl_2_BTDD (4.78 mmol/g).^360^ These uptake capacities correspond to 1.48 molecules of NH_3_ per cobalt site in Co_2_Cl_2_BBTA and 1.08 molecules of NH_3_ per cobalt site in Co_2_Cl_2_BTDD. Deviations between equilibrium and breakthrough capacities highlight the impact of heat generation, nonisothermal temperature effects under flow conditions, and mass-transfer resistances in dynamic breakthrough operations. However, the total isothermal NH_3_ uptake capacities of Co_2_Cl_2_BBTA gradually decreased over three consecutive cycles (∼6% decrease of its original capacity), even after activation at 473 K under a dynamic vacuum between each cycle. Additionally, NH_3_ adsorption capacities and stabilities of both the MOFs (Co_2_Cl_2_BBTA and Co_2_Cl_2_BTDD) were significantly affected under wet conditions. At 80% RH, the Co_2_Cl_2_BBTA has an adsorption capacity of 4.36 mmol/g, whereas Co_2_Cl_2_BTDD has 3.38 mmol/g, corresponding to 0.76 NH_3_ molecules per open metal site in Co_2_Cl_2_BBTA and 0.77 NH_3_ molecules in Co_2_Cl_2_BTDD, respectively.

Optical calorimetric analysis was employed to investigate NH_3_ adsorption kinetics in Co_2_Cl_2_BBTA and Co_2_Cl_2_BTDD. These studies reveal that large-pore M_2_Cl_2_BTDD (2.3 nm) exhibits more rapid NH_3_ adsorption kinetics than the small-pore M_2_Cl_2_BBTA (1.3 nm) due to the shorter distance between neighboring chains (ligands) in small-pore M_2_Cl_2_BBTA, possibly enhancing hydrogen–bonding interactions between the first metal-bound NH_3_ molecules and the subsequent adsorbed NH_3_ molecules (2nd to third NH_3_ etc.), hindering NH_3_ diffusion. This phenomenon is less pronounced in the larger-pore M_2_Cl_2_BTDD series due to large pores (2.2 nm vs 1.3 nm). Additionally, the higher NH_3_ capacity of M_2_Cl_2_BBTA is attributed to its greater density of open metal sites over M_2_Cl_2_BTDD.

The influence of open metal sites in the microporous MOF (M­(NA)_2,_ M = Zn, Cu, Co, Cd, and NA = 3-pyridinecarboxylate) on NH_3_ adsorption was investigated using isothermal gas adsorption–desorption analysis.^301^ NH_3_-assisted synthesis, followed by solvent-evaporated conversion activation, yields flexible microporous frameworks (M­(NA)_2_) of 2D (Zn, Co) and 3D (Cu, Cd) materials with a pore size ranging from 3.4 to 3.8 Å. The NH_3_ adsorption capacities of the MOFs were found to be 17.5 mmol/g for Co­(NA)_2_, 13.4 mmol/g for Cu­(NA)_2_, and 6 mmol/g for Cd­(NA)_2_ at 1 bar. However, none of the MOFs desorbs NH_3_ under ambient conditions, and desorption requires ∼ 423 K activation. Powder-XRD patterns after NH_3_ adsorption reveal a peak shift, and the presence of additional peaks possibly due to the partial decomposition of the framework. The analogous MOF, Zn­(INA)_2_ exhibited an adsorption capacity of 6 mmol/g at 1 bar, which remained consistent for three adsorption cycles upon activation at 423 K.^326^ PXRD analysis revealed peak shift upon saturation with NH_3_, attributed to formation of a Zn­(INA)_2_(H_2_O)_2_(NH_3_)_2_ complex, which can be transformed back to pristine Zn­(INA)_2_ through activation at 423 K.

In 2020, Kim *et al*. reported on an analogous MOF-74 framework with extended porous structure (M_2_(dobpdc), M = Mg^2+^, Mn^2+^, Co^2+^, Ni^2+^, and Zn^2+^) made using 4,4’-dioxidobiphenyl-3,3′-dicarboxylate linker.^270^ The crystal structure of M_2_(dobpdc) shows the presence of Lewis acidic open metal centers and large pores (∼1.8 to 2.2 nm) over isostructural MOF-74 (∼1.2–1.5 nm) (Figure 16g and 16h).^265^ Ammonia adsorption results revealed distinct adsorption capacities for each metal of the M_2_(dobpdc) family. At low pressure, Mg_2_(dobpdc) demonstrated adsorption of 7.82 mmol/g at 0.072 mbar, which reached the capacity of 23.9 mmol/g at 1 bar. The isostructural Mn_2_(dobpdc) exhibited 4.77 mmol/g at 0.36 mbar; Co_2_(dobpdc) displayed 4.72 mmol/g at 0.54 mbar; Ni_2_(dobpdc) displayed 5.10 mmol/g at 0.58 mbar; and Zn_2_(dobpdc) showcased 4.42 mmol/g at 0.42 mbar. At 298 K, the total adsorption capacities of M_2_(dobpdc) were found to be 23.90, 13.26, 13.34, 20.82, and 15.24 mmol/g for Mg, Mn, Co, Ni, and Zn, respectively, at 1 bar (Figure 16i and Table 4). Cycling experiments show that M_2_(dobpdc) frameworks (M = Mg^2+^, Mn^2+^, Co^2+^, Ni^2+^, and Zn^2+^) exhibited NH_3_ adsorption profiles closely resembling those of the first adsorption cycle at low pressure, except for the Zn_2_(dobpdc), which shows noticeable deviation upon cycling. The high uptake capacity of Mg- and Ni_2_(dobpdc) MOFs are ascribed to the higher Lewis acidities among the other metals. However, all of these MOFs (M_2_(dobpdc), M = Mg^2+^, Mn^2+^, Co^2+^, Ni^2+^, and Zn^2+^) exhibited large fractions of irreversible NH_3_ adsorption (∼30–35%) upon pressure decrease.^270^

Breakthrough experiments (1000 ppm of NH_3_ feed) revealed that Mg_2_(dobpdc) displayed capacities of 8.37 and 6.14 mmol/g at 298 K, 0% RH, and 80% RH, respectively.^297^ The total NH_3_ uptake capacity under wet breakthrough conditions remains ∼ 8% lower than its dry breakthrough capacity. However, the total capacity of the wet breakthrough remained constant (∼6.14 mmol/g for each cycle) over five consecutive cycles under vacuum treatments between the cycles. Powder-XRD, FT-IR, and N_2_ sorption results after NH_3_ adsorption (three cycles) indicated that all the samples retained their structural integrity, except for Zn-MOFs, which lost crystallinity.

The affinity of NH_3_ to the open metal sites in the Mg_2_(dobpdc) was further investigated using temperature-programmed desorption (TPD) analysis and an evaluation of the activation energy for NH_3_ desorption.^270,306,361^ The NH_3_-TPD desorption peak temperature for the Mg, Mn, Co, and Ni MOFs were 522, 482, 505, and 553 K, respectively. The activation energy of the desorption was estimated to be 159.0 (Mg), 146.7 (Mn), 153.8 (Co), and 168.6 kJ/mol (Ni). The NH_3_ desorption temperature and activation energy trends follow the Lewis acidity strength of the metal ions (Ni > Mg > Co > Mn).^270^ Additionally, the absolute binding energies obtained from the vdW-corrected Density Functional Theory (DFT) calculations were consistent with the experimental trend of NH_3_ desorption.^270^ The adsorption mechanism of Mg_2_(dobpdc) was further validated using an in situ FT-IR analysis, which indicates that NH_3_ exposed samples displayed new peaks at 1625 and 1140 cm^–1^, assigned to the Mg-NH_3_ adduct.^130,287,362^

In 2023, Long and co-workers presented a detailed study of the NH_3_ insertion mechanism into the Cu-carboxylate bonds within coordinatively saturated copper­(II) sites of Cu­(cyhdc) (Figure 17a).^262^ The pristine Cu­(cyhdc) demonstrates reversible cooperative phase transitions and structural transformations during the adsorption and desorption of NH_3_. Additionally, it exhibits temperature and pressure swing reversible NH_3_ adsorption performance providing remarkable insights into how NH_3_ can alter the structure of MOFs.^262^ Cu­(cyhdc) is constructed using trans-1,4-cyclohexanedicarboxylate (cyhdc^2–^) and copper­(II) salts, featuring 1D chains of dicopper paddlewheel units connected by ditopic cyhdc linkers (Figure 17a).^262^ This forms a 3D structure with rhombic channels along the *c*-axis. Isothermal NH_3_ adsorption studies reveal that the pristine Cu­(cyhdc) exhibits a stepwise uptake of ∼ 16 mmol/g at 0.08 bar and 18.7 mmol/g at 298 K and 1 bar (Figure 17d). Notably, Cu­(cyhdc) showed a negligible amount of uptake capacity for H_2_ and N_2_ gases under the same conditions.

**Figure 17:**
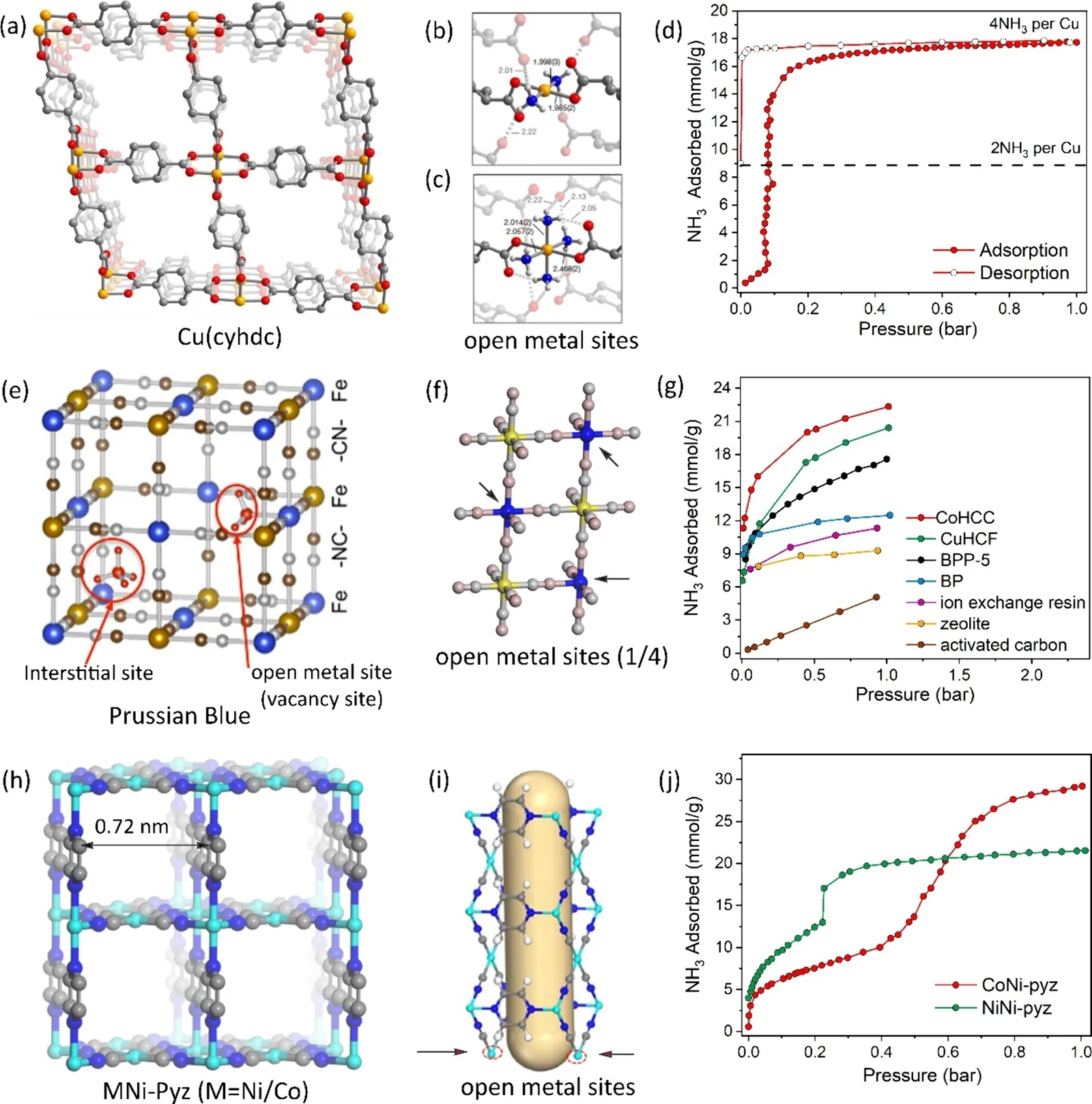
(a) Crystal structure of Cu­(cyhdc) showing the rhombic channels with vertices formed by Cu-carboxylate paddlewheel units. (b) 1D polymeric structure and local Cu environment (featuring open metal sites) of Cu­(cyhdc) when two NH_3_ molecules are adsorbed per Cu-site (Cu­(NH_3_)_2_(cyhdc)). (c) Single-crystal structure and coordination environment of Cu­(cyhdc) when four NH_3_ molecules are adsorbed per Cu-site­((NH_3_)_4_Cu­(cyhdc)). 17a–c adopted with permission from ref 262. Copyright 2023 Springer Nature. (d) NH_3_ adsorption–desorption isotherms of Cu­(cyhdc) at 298 K. Data source: ref 262. (e) Structure of Prussian blue shows the two adsorption sites (interstitial site and open metal/vacancy site. (f) Lewis acidic open metal centers of Prussian blue. 17e and 17f adopted with permission from ref 130. Copyright 2016 American Chemical Society. (g) Comparison of NH_3_ adsorption isotherms of Prussian blue, copper hexacyanoferrate (CuHCF), cobalt hexacyanocobaltate (CoHCC), and various commercial adsorbents. Data source: ref 130. (h) Crystal structure of MNi-Pyz. (i) 1D channels showing distinct open metal sites (M = Ni/Co). 17h and 17i adopted with permission from ref 253. Copyright 2023 Springer Nature. (j) NH_3_ adsorption isotherms of MNi-Pyz at 298 K. Data source: ref 253.

The crystal structure analysis of Cu­(cyhdc) after NH_3_ adsorption ((NH_3_)_4_Cu­(cyhdc)) reveals that each copper­(II) is coordinated with four equatorial NH_3_ molecules, and two axial positions are bridging with a single oxygen atom of the cyhdc^2–^ ligand at 298 K and 1 bar (Figure 17b and 17c). The equatorial NH_3_ molecules form hydrogen bonding interactions with secondary sphere oxygen atoms (∼2.0 to 2.2 Å). The uncoordinated oxygen atom of the cyhdc^2–^ ligand engages with nearby NH_3_ ligands, contributing to the formation of a stabilizing network. This interaction leads to the formation of a nonporous, 1D coordination polymer Cu­(NH_3_)_4_(cyhdc) (Figure 17c). Desorption profiles of NH_3_ sorption isotherms show an apparent hysteresis upon isothermal NH_3_ desorption from Cu­(NH_3_)_4_(cyhdc), and only half of the total NH_3_ uptake capacity was desorbed (Figure 17d). The X-ray diffraction analysis of the intermediate phase reveals that two molecules of NH_3_ are present in each copper center (Cu­(NH_3_)_2_(cyhdc)), which have hydrogen bonding interactions with neighboring oxygen atoms (Figure 17b). Despite the occurrence of these NH_3_ ligand insertions, Cu­(cyhdc) has 9.0 mmol/g of NH_3_ adsorption capacity at 458 K, 6 bar pressure; this uptake capacity was reversible upon further activation at higher temperatures (>458 K) or low pressure (<0.01 bar). The working capacity of Cu­(cyhdc) was found to be 5.4 mmol/g with a pressure swing between 1.2 and 0.05 bar at 423 K; or a temperature swing between 373 and 458 K at 0.75 mbar; or a pressure temperature swing between 1.2 bar at 423 K and 0.75 bar at 458 K.

Mixed-metal MOFs (MM-MOFs) enable precise adjustment of porosity and physical and electronic characteristics through the integration of different active metals.^363^ MM-MOFs enable precise adjustment of porosity and physical and electronic characteristics through the integration of different active metals into MOFs. These characteristics render them highly suitable for gas separation and adsorption.^363,364^ Prussian blue (PB) analogues are a class of mixed-metal coordination polymers known for decades that have intrinsic pores and active open metal sites.^365,366^ In 2016, the pigments of Prussian blue (K_0.23_Fe­[Fe­(CN)_6_]_0.74_), cobalt hexacyanocobaltate (CoHCC), and copper hexacyanoferrate (CuHCF) were investigated for H_3_ adsorption under ambient conditions.^130^ The crystal structures of these pigments contain two distinct adsorption sites: open metal site/vacancy sites (site I) and interstitial sites (site II) (Figures 17e and 17f). Notably, CoHCC (848 m^2^/g) and CuHCF (457 m^2^/g) exhibited higher vacancy sites and surface areas than Prussian blue (280 m^2^/g). Prussian blue showed an NH_3_ adsorption capacity of 12.5 mmol/g, while CoHCC and CuHCF demonstrated capacities of 21.9 and 20.6 mmol/g, respectively, under similar conditions (Figure 17g). CoHCC maintained its total adsorption capacity over four consecutive adsorption cycles upon activation at 423 K under a vacuum for 24 h. It also exhibited high removal efficiencies for low NH_3_ concentrations (more than 90% for 15 ppbv NH_3_) from air. Powder XRD patterns of Prussian blue and CoHCC remained unchanged after NH_3_ adsorption, while CuHCF transformed into an amorphous material. FT-IR spectra revealed that adsorbed NH_3_ in Prussian blue transformed into NH_4_^+^ ions at site I and formed coordination bonds with vacant Fe sites (Fe^II^–NH_3_ and Fe^III^–NH_3_) at site II in the presence of water. Conversely, under dry conditions, both sites in these materials adsorbed the NH_3_ molecules. Thereafter, various metal substitutions and dopings into PB materials were conducted.^355^ However, the overall adsorption capacities of these materials were lower than that of CoHCC.

In 2023, Wang *et al*. reported mixed metal MOFs featuring open metal sites and vacancy sites (Ni­(pyz)­[Ni­(CN)_4_] /(NiNi-Pyz) and Co­(pyz)­[Ni­(CN)_4_]/(CoNi-Pyz)) and systematically examined room temperature NH_3_ detection and adsorption.^253^ The crystal structure of NiNi-Pyz revealed that the 4-connected planar building blocks of [Ni­(CN)_4_]^2–^ inorganic ligands engaged with M^2+^ nodes, giving rise to 2D layers (Figure 17h and 17i). Pyrazine ligands are further interconnected to these layers, forming a 3D network with 1D channels containing distinct open metal sites. The M^2+^ unsaturated metal sites were uniformly distributed along the walls of the 1D channel. The BET surface area was determined as 488 and 585 m^2^/g for NiNi-Pyz and CoNi-Pyz, respectively, with corresponding pore sizes of 7.2 and 6.8 Å. Ammonia adsorption studies of NiNi-Pyz and CoNi-Pyz displayed a stepwise increase in NH_3_ adsorption with increasing pressure and achieved 21.5 and 29.1 mmol/g of NH_3_ uptake capacities, respectively, at 298 K (Figure 17j). However, the authors have not provided the desorption isotherms and indicate that the regeneration of MNi-Pyz was accomplished under vacuum for 2 h at 353 K. They also stated that the adsorption isotherm rises more rapidly for NiNi-Pyz than CoNi-Pyz due to a denser distribution of open metal sites. However, above 0.5 bar, CoNi-Pyz reached a higher adsorption capacity, possibly due to its larger surface area and pore volume.

Furthermore, gas adsorption results obtained through grand canonical Monte Carlo (GCMC) simulations suggested that NiNi-Pyz possesses three binding sites.^253^ In comparison, CoNi-Pyz features four binding sites, with the fourth exhibiting weaker affinity and predominantly residing in the middle of the pore, contributing to increased capacity at elevated pressure.^253^ Ammonia sensing studies revealed that NiNi-Pyz has a linear response for a broad range of pressures (1–1000 ppm) at room temperature. Notably, low sensor detection limits of 25 and 97 ppb were achieved for NiNi-Pyz and CoNi-Pyz, respectively. The sensing detection limit of MNi-Pyz is higher than that of well-known materials such as Cu-HHTP, Cu_3_HITP, Ni_3_(HHTP), Cu-BHT film, or NiPc-Ni, and lower than that of the current benchmark, Cu-HHTP 3D films.^253,367^ The cyclic sensing performance of the sensors at 100, 225, and 500 ppm of NH_3_, under 35% relative humidity (RH) over a two-month test period, underscored the MNi-Pyz sensors’ high stability. This study is a promising illustration of the cooperative integration of microporosity and open metal sites within the MNi-Pyz structure, leading to a synergistic enhancement of uptake capacity and sensing performances.

A comparative analysis of open-metal-site MOFs reveals a structure–property relationship when performance is evaluated across both metal identity and framework topology. Within isostructural families such as M_2_Cl_2_(BTDD), M_2_Cl_2_(BBTA), and M_2_(dobpdc), NH_3_ uptake, adsorption kinetics, and stability are strongly influenced by the nature of the metal center.^270^ In general, Mg-based frameworks display high uptake and pronounced stepwise adsorption behavior, consistent with strong metal–NH_3_ interactions and cooperative adsorption along one-dimensional metal–ligand chains. Other analogs Ni-, Co-, and Mn-based MOFs typically exhibit comparatively lower uptake or less cooperative behavior, although their adsorption–desorption reversibility and structural stability can vary depending on framework composition and operating conditions.^270,293,306^ In contrast, Cu­(cyhdc) follows a distinct adsorption mechanism involving NH_3_ insertion into the coordination environment, leading to high capacities but often accompanied by hysteresis and partial structural transformation.^262^

Furthermore, the framework’s topology also modulates these trends. Larger-pore systems, such as M_2_Cl_2_(BTDD) (∼2.2 nm), generally exhibit faster adsorption kinetics due to reduced diffusion limitations. In contrast, smaller-pore analogs such as M_2_Cl_2_(BBTA) (∼1.3 nm) provide higher open-site densities and enhanced guest–guest interactions (e.g., NH_3_–NH_3_ hydrogen bonding), which can increase uptake at the expense of slower diffusion. Similarly, extended frameworks like M_2_(dobpdc) combine high site accessibility with cooperative adsorption behavior but may exhibit partial irreversibility, depending on the metal–NH_3_ binding strength.^270,293,306^ These studies suggest that NH_3_ adsorption performance in open-metal-site MOFs cannot be attributed solely to the presence of open metal sites. Instead, it is the interplay among metal-centered Lewis acidity, open-site density, pore architecture, and framework stability. Within these frameworks, the dominant adsorption mechanism (e.g., metal–NH_3_ coordination, cooperative binding, or insertion-type interaction) strongly influences overall NH_3_ adsorption capacity and adsorption–desorption performance.

### 3.3 MOFs Containing Polar Functional Groups

Integrating functional groups (e.g., hydroxyl (−OH), amine (−NH_2_), etc.) into MOFs has garnered increasing attention alongside the open metal approach, primarily due to the moderate interactions between functional groups and NH_3_, facilitating adsorption–desorption reversibility and good cycling efficiency in addition to the enhanced NH_3_ adsorption capacity.^28,315,368,369^ Unlike open metal sites, they possess improved structural stability in humid-NH_3_ environment and exhibit durability by avoiding direct interactions between NH_3_ and the metal center.

Isostructural MOFs are frameworks with the same underlying topology or crystal structure but can differ in functional groups, metal nodes, or pore sizes.^370^ In 2015, Jasuja *et al*. investigated the impact of various functional groups within the pores of isostructural MOFs, UiO-66-X (X = −H, −NH_2_, −NO_2_, −OH, −(OH)_2_, −SO_3_H, −(COOH)_2_) on the removal of NH_3_ from air.^272^ The crystal structure of all UiO-66-X MOFs resembled that of UiO-66 (pore size ∼ 6 Å), with slightly reduced pore sizes due to additional functional groups. Ammonia breakthrough curves (feed concentration of 1000 or 2000 mg/m^3^ (1438 or 2876 ppm)) for UiO-66-X exhibited adsorption capacities of 1.79, 1.98, 2.24, 2.29, 2.83, 3.56, and 5.69 mmol/g for X = −H, −NO_2_, −SO_3_H, −(OH)_2_, −(COOH)_2_, −NH_2_, and −OH, respectively at 0% RH. The bulkier acidic functional groups (−SO_3_H and −(COOH)_2_) in UiO-66 obstruct framework pores, reducing NH_3_ adsorption capacity. The highest NH_3_ adsorption capacity observed in UiO-66-OH (5.69 mmol/g) was attributed to the smaller size of the −OH group in the framework compared to −NH_2_.^371^ However, under humid conditions (80% RH), a significant decrease in NH_3_ capacity was observed for all the functionalized UiO-66-X compared to UiO-66.

Subsequently, isostructural NH_2_-MIL-53­(Al) and MIL-53­(Al) MOFs were investigated; both MOFs feature 1D diamond-shaped channels with 1.71 and 1.22 nm pore sizes.^164^ At 298 K, MIL-53 and NH_2_-MIL-53 exhibit NH_3_ adsorption uptakes of 4.4, and 5.4 mmol/g, respectively.^261^ Both MOFs retained their total adsorption capacity for five consecutive cycles at 298 K. The enhanced NH_3_ adsorption capacity of NH_2_-MIL-53 over MIL-53 is attributed to the presence of polar amine (−NH_2_) functional groups that allow for hydrogen bonding interactions with NH_3_. Similar effects on NH_3_ adsorption were seen in the isostructural hydroxyl (−OH) functionalized OH-DUT-6 and DUT-6 MOFs.^303^ The total NH_3_ adsorption capacity of OH-DUT-6 and DHT was found to be 16.4 and 12.0 mmol/g at 298 K and 1 bar, respectively.

In 2021, Wang and co-workers further studied the influence of the number (1–4) and nature of adsorption sites on reversible NH_3_ adsorption performance using MIL-160, CAU-10-pydc, Al-Fum, and MOF-303­(Al) (Figure 18a).^294^ DFT methods were used to obtain Molecular Electrostatic Potential (MEP)-guided theoretical insights. A combination of MEP maps, atomic charges, and hydrogen bonding interaction energies were employed to identify and rank the NH_3_ adsorption sites of the ligands. Using this ligand screening strategy, they prepared a MOF-303­(Al) featuring multiple adsorption sites (N–H, μ-OH, H–C, and N–N; using 3, 5-pyrazoledicarboxylic acid ligand) (Figure 18a and 18b) and compared it with single-site (Al-Fum) and dual-site MOFs (MIL-160 and CAU-10). The single-site Al-Fum featured only a μ-OH site and the dual-site MIL-160 and CAU-10, each containing a μ-OH moiety and O–C site (MIL-160) or N–C site (CAU-10). Ammonia adsorption isotherms showed that MOF-303­(Al) has an NH_3_ adsorption capacity of 19.7 mmol/g at 298 K, and 1 bar (Figure 18c), while the NH_3_ adsorption capacities of MIL-160, CAU-10-pydc, and Al-Fum were found to be 12.7, 12.4, and 10.4 mmol/g, respectively, under the same conditions, which is significantly lower than that of MOF-303­(Al).^298^ Notably, MOF-303­(Al) retained ∼ 95% of its initial capacity after 20 adsorption–desorption cycles, following the activation of the MOF after NH_3_ adsorption at 423 K under vacuum for 2 h. The superior NH_3_ capacity and favorable sorption behavior of MOF-303­(Al) are attributed to multiple adsorption sites (N–H, μ-OH, H–C, and N–N) and their favorable hydrogen bonding interactions with NH_3_ compared to MIL-160, CAU-10-pydc, and Al-Fum. Building on this concept, Kim *et al*. (2026)^295^ extended the structure–function relationship to a family of isostructural CAU-10-type 1D aluminum MOFs (CAU-23, KMF-1, MIL-160, and MOF-303), where systematic variation of heterocyclic linkers (thiophene, pyrrole, furan, and pyrazole) enabled the framework chemistry from the same topology.^295,372^ All materials exhibit corner-sharing AlO_6_ chains forming microporous channels (∼5.5–6.5 Å). NH_3_ adsorption isotherms show that MOF-303, CAU-23, KMF-1, and MIL-160 exhibit steep uptake at low pressure (0.05 bar), with capacities of ∼ 18.5, ∼ 11.7, ∼ 11.8, and ∼ 12.2 mmol/g at 303 K and 1 bar, respectively, while at higher pressure (7 bar, 303 K) the corresponding uptake increase to 18.5, 17.4, 16.5, and 14.4 mmol/g. Notably, high-pressure NH_3_ adsorption in MOFs remains relatively underexplored, and this study represents a rare example demonstrating substantial and reversible uptake under elevated pressure conditions.^295^ isosteric heats of adsorption, which are found to be ∼ 34 kJ/mol for CAU-23 and ∼ 39–45 kJ/mol for MOF-303, KMF-1, and MIL-160.

**Figure 18:**
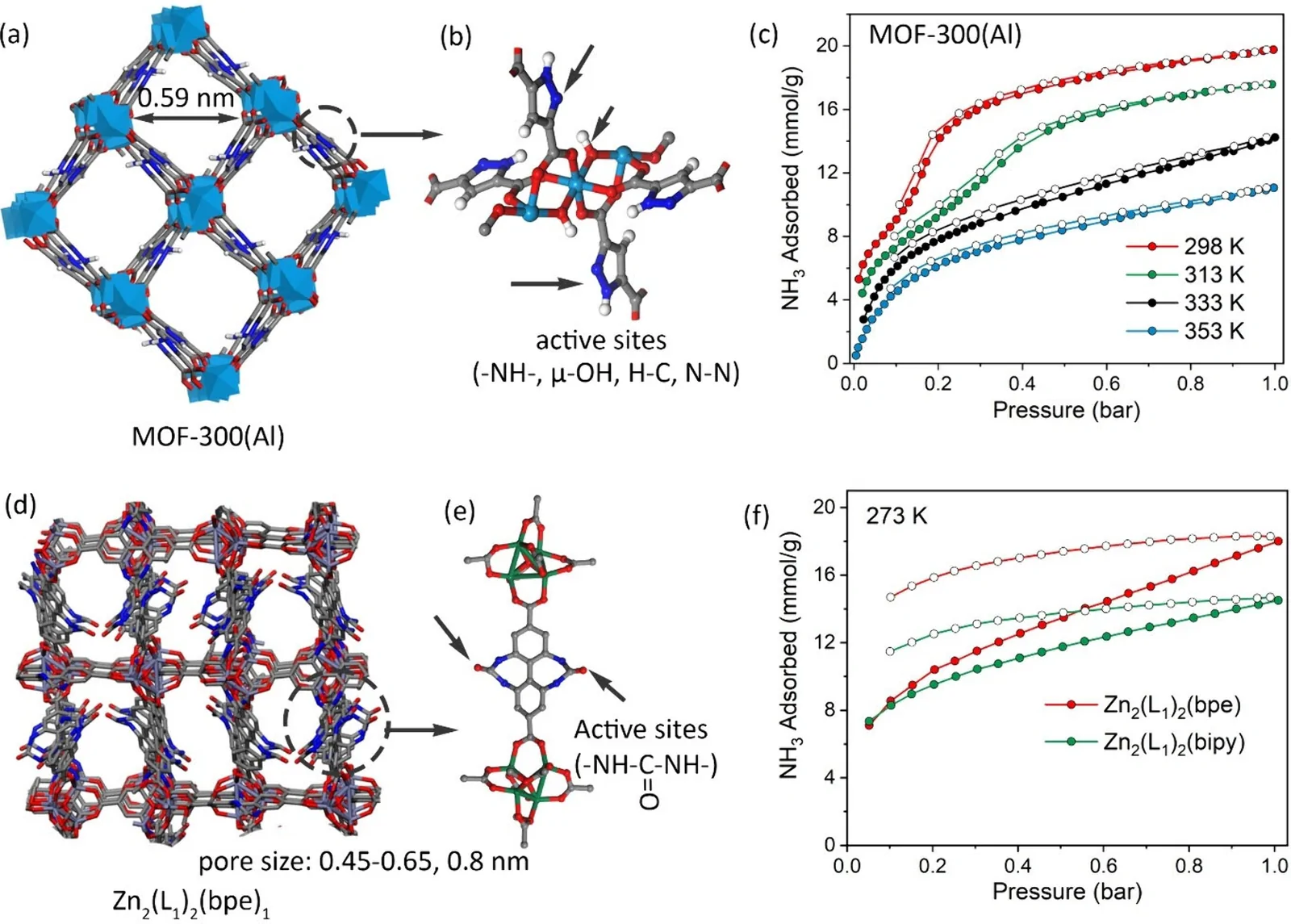
(a) Structure of MOF-300­(Al) showing the 0.59 nm pores along the *a*-axis. (b) NH_3_-affinity −NH– and μ-OH functional groups of MOF-303­(Al). (c) NH_3_ adsorption–desorption isotherms of MOF-303­(Al) at various temperatures (298–353 K). Data source: ref 294. (d) Structure of [Zn_2(_L_1_)_2_(bpe)] shows the microporous channels and urea functional groups pointing toward the pores. (e) NH_3_-affinity −NH–CO–NH–functional groups of [Zn_2(_L_1_)_2_(bpe)]. (f) NH_3_ adsorption–desorption isotherms of [Zn_2_(L1)_2_(bipy)], and [Zn_2(_L_1_)_2_(bpe)] (Ligands; L_1_ = 6-oxo-6,7-dihydro-5H-dibenzo­[d,f]­[1,3]­diazepine-3,9-dicarboxylic acid (a urea-functionalized biphenyl-4,4′-dicarboxylic acid); bipy = 4,4′-bipyridine; bpe = 1,2-bis­(4-pyridyl)­ethane) at 273 K. Data source: ref 298.

Similar to the Wang *et al*. study,^294^ a steep increase in adsorption at low pressure was observed for the NH_3_ adsorption isotherms, indicating that frameworks with polar heteroatom-functionalized linkers in MOF-303, KMF-1, and MIL-160 exhibit strong interactions between NH_3_ and the functional groups of the host material.^295^ In contrast, CAU-23 shows a comparatively lower initial adsorption due to the absence of strongly interacting heteroatom sites within its thiophene-based linkers.^295^ Grand canonical Monte Carlo simulations suggest that NH_3_ adsorption in CAU-23 first takes place at μ-OH sites, followed by the formation of intermolecular hydrogen-bonded NH_3_ clusters at higher loadings.^295^ In contrast, MOF-303, MIL-160, and KMF-1 exhibit stronger direct interactions between NH_3_ and ligand functional groups. Cycling experiments reveal that CAU-23 demonstrates excellent stability, retaining over 95% of its initial capacity after three adsorption–desorption cycles, whereas MIL-160 and MOF-303 exhibit significant irreversible losses of ∼ 46% and ∼ 39%, respectively. KMF-1 shows noticeable morphological degradation. PXRD, IR, and N_2_ sorption measurements confirm that CAU-23 maintains its crystallinity and porosity after cycling, whereas MOF-303, KMF-1, and MIL-160 exhibit partial structural degradation.

Recently, Huang *et al*. examined the effects of functional groups in the isostructural aluminum metal–organic frameworks (Al-MOFs) CAU-1-OH and CAU-1-NH_2_ on the reversible capture of NH_3_ from air.^304^ Both MOFs were synthesized using AlCl_3_·6H_2_O and 2-amino-terephthalic acid (for CAU-1-NH_2_) or 2-hydroxyterephthalic acid (for CAU-1-OH). Crystal structure analysis reveals that each Al^3+^ ion is coordinated with six oxygen atoms, with one hydroxide group (μ-OH) bridging between the aluminum centers. The resulting framework forms an infinite three-dimensional structure, in which the −OH and −NH_2_ functional groups point toward pores (5–11 Å) within the tetrahedral and octahedral cages.

The NH_3_ static adsorption isotherms demonstrate that both CAU-1-OH and CAU-1-NH_2_ exhibit uptake capacities of 16.3 and 16.5 mmol/g, respectively, at 298 K and 1 bar. The total uptake capacity remains consistent, with only 2.5–3.0% capacity change after ten adsorption–desorption cycles. Notably, both materials show negligible N_2_ and O_2_ uptake under the same conditions. The IAST selectivities of CAU-1-NH_2_ for NH_3_ over N_2_ and O_2_ are 1168 and 3848, respectively, in 1/99 (NH_3_/N_2_ and NH_3_/O_2_) mixtures. The isosteric heats of adsorption were found to be 41.2 kJ/mol for CAU-1-OH and 47.6 kJ/mol for CAU-1-NH_2_. Additionally, both materials maintain their PXRD crystallinity and retain their surface area or nitrogen (N_2_) uptake capacity after 10 cycles. In situ infrared spectroscopy and DFT simulations indicate the presence of strong hydrogen bonding interactions between ammonia (NH_3_) and the μ-OH, −OH, and −NH_2_ groups in the frameworks. These results demonstrate that functional groups strongly influence NH_3_ adsorption, particularly at low concentrations, where interactions with pore functionalities dominate binding.^304,373^

As discussed earlier, Zirconium-based metal–organic frameworks (Zr-MOFs) have high stability due to the robustness of Zr–O bonds. These bonds resist hydrolysis or acid/base reactions, allowing for a variety of structural and postsynthetic modifications to tailor MOFs for specific applications.^374^ In 2011, postsynthetic modification techniques were adopted to functionalize (UiO-66-NH_2_ (UiO-66-A)) to enhance the NH_3_ adsorption performance.^273^ The parent UiO-66-A synthesized from zirconium tetrachloride and H_2_BDC-NH_2_ (2-Aminobenzene-1,4-dicarboxylic acid) comprises 66% amino and 33% ammonium chloride functional groups within it (Zr_6_O_4_(OH)_4_(BDC-NH_2_)_4_(BDC-NH_3_^+^Cl^–^)_2_). The NH_2_ functional groups in UiO-66-A were further converted to hemiaminal products by reacting with acetaldehyde (hemiaminal products are intermediate products containing different ratios of ammonium chloride and other functional groups in UiO-66-B). Upon heat treatment of UiO-66-B under vacuum at 358 K for 24 h, UiO-66-C was obtained, which was found to exist in thermal equilibrium with the aziridine product (the three-membered heterocycle C_2_H_5_N, 56%). Ammonia sorption isotherms show that the adsorption uptakes of postmodified UiO-66-C and UiO-66-B are 8.6 mmol/g (193 cm^3^/g) and 7.1 mmol/g (159 cm^3^/g), respectively, at 298 K and 1 bar. Both capacities are superior to pristine UiO-66-A at 6.0 mmol (134 cm^3^/g) under the same conditions. Notably, desorption isotherms showed good reversibility. Although UiO-66-A, B, and C exhibited less adsorption capacity than best-performing MOFs (Table 4), their stability and adsorption–desorption reversibility is promising. These results reveal that having a higher density of active functional groups or strong interactive sites enhances total adsorption capacity, but often induces unfavorable desorption, irreversibility, or hysteresis.

A noticeable example of this type of phenomenon was observed in MOFs constructed from rigid ditopic urea-functionalized linkers (L_1_ = 6-oxo-6,7-dihydro-5H-dibenzo­[d,f]­[1,3]-diazepine-3,9-dicarboxylic acid (a urea-functionalized variant of the well-known ligand, biphenyl-4,4′-dicarboxylic acid); bipy = 4,4′-bipyridine; bpe = 1,2-bis­(4-pyridyl)­ethane) and zinc metal salts (Figure 18d–f).^298^ The NH_3_ adsorption isotherms of [Zn_2_(L1)_2_(bipy)] and [Zn_2_(L_1_)_2_(bpe)] shows more than 80% of irreversible capacity during the desorption, with total uptake capacities of 18.0 and 14.5 mmol/g, respectively, at 273 K and 1 bar. (Figure 18f). In contrast, MOF-300­(Al) exhibited no hysteresis (down to ca. 0.1 bar; desorption data at lower pressures are not given)­despite the presence of the same active (−NH−) functionalities. This phenomenon is likely due to the combined influence of adjacent functional groups and local environment of active sites. The presence of carbonyl or carboxyl (−C=O, and −C–O) moieties in Ni_acryl_TGA and [Zn_2_(L_1_)_2_(bpe)] besides the active sites possibly induces the strong NH_3_ binding affinity and thus pronounced hysteresis (and irreversible desorption at low pressure) was observed. Such effects are absent in MOF-300­(Al). Furthermore, MOFs with terminal active sites, such as NH_2_-MIL-53­(Al) and UiO-66-NH_2_, exhibited negligible hysteresis and good adsorption–desorption reversibility.^261,264,273,315^ These results suggest that NH_3_ desorption reversibility in MOFs is governed not only by the nature of functional groups but also by the spatial distribution and local environment of active sites.

Across polar-functionalized MOFs, NH_3_ adsorption is affected by adsorption site strength and spatial arrangement. In UiO-66 derivatives, modulation of functional groups demonstrates a clear trade-off, where moderate functionalities such as −OH and −NH_2_ enhance uptake (up to 2.5-fold), while bulkier or strongly acidic groups like −COOH and −SO_3_H hinder accessibility and reduce performance, particularly in the presence of other competing gases and humid conditions.^261,264,273,294,298,315^ A similar behavior is observed in MIL-53­(Al) and DUT-6 systems, where pore flexibility and channel dimensions govern the competition between enhanced host–guest interactions and diffusion-limited transport. In contrast, CAU-1-based frameworks highlight the significance of structural robustness combined with uniformly distributed functional sites, enabling consistently high uptake and cycling stability.^304^ This trend is particularly evident in MOF-303­(Al), where the presence of multiple cooperative adsorption sites (−NH, μ-OH, C–H, N–N) leads to an ∼ 89% increase in NH_3_ uptake compared to single- and dual-site analogues (Al-Fum, MIL-160, and CAU-10).^261,303^

### 3.4 MOFs Containing Bro̷nsted Acidic Sites

Metal–organic frameworks with Bro̷nsted acidic sites (H^+^ donors) exhibit strong NH_3_ affinity due to the acid–base and hydrogen bonding interactions between the framework and ammonia molecules.^324,375^ These Bro̷nsted acidic sites are often synthesized using oxo-clusters of metal centers or incorporated through functional groups of organic linking units (e.g., −COOH, −SO_3_H) or encapsulated within the pores of the framework (e.g., HCl, HCOOH).^375,376^ The interaction between NH_3_ and Bro̷nsted acidic sites in MOFs results in good adsorption–desorption reversibility under ambient conditions, in addition to the enhanced uptake capacity.

In 2018, Schröder and co-workers reported a series of microporous MOFs (MFM-300­(M) (M = Al, In, Fe, Cr, V^III^, V^IV^, or Sc)) containing Bro̷nsted acidic sites that are synthesized using biphenyl-3,3′,5,5′-tetracarboxylic acid ligand (L) and various metal salts (Al, In, Fe, Cr, V^III^, V^IV^, or Sc).^264,308,311,324,377^ Each metal center in MFM-300­(M) displays octahedral coordination, comprising four carboxylate oxygen atoms from the ligand and two hydroxyl groups, which act as Bro̷nsted acidic sites. The resulting MFM structures form a “wine rack” network [Al_2_(OH)_2_(L)]_∞_ with uniform microporosity (0.65 to 0.75 nm), where the bridging hydroxyl groups (μ-OH) within the MOF (Fe, V^III^, Cr, Al) channels are oriented toward the pores of the framework (Figure 19a–c). Oxidation of MFM-300 (V^III^) in air enables the removal of the proton from the bridging hydroxyl groups, leading to the formation of MFM-300­(V^IV^) via the V^IV^–O–V^IV^ oxygen atom.^264,378^

**Figure 19:**
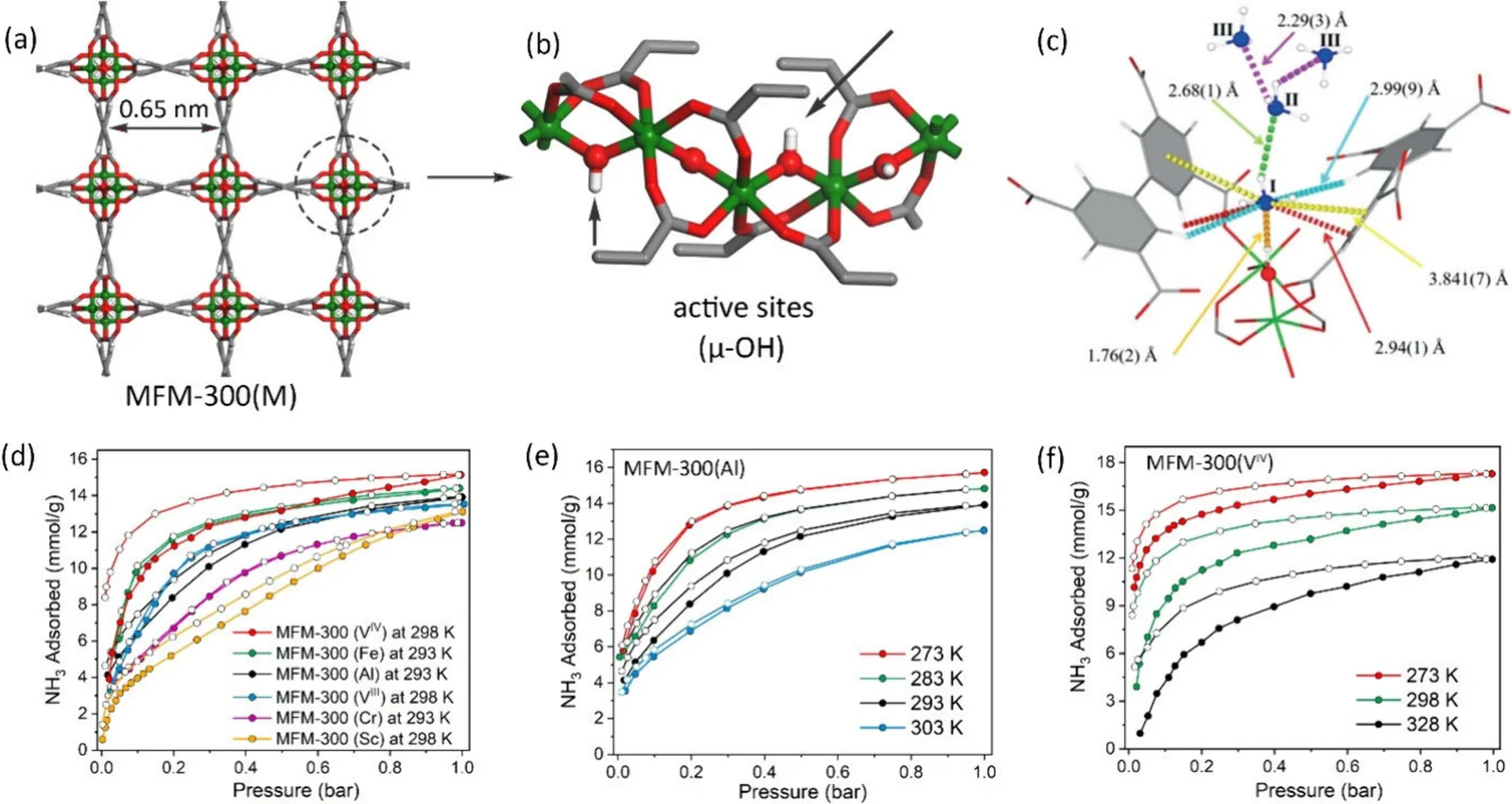
(a) Crystal structure of MFM-300­(Al) showing the 0.65 nm pores along the *b*-axis. (b) Bro̷nsted acidic sites (μ-OH) in MFM-303­(Al). (c) Top view of MFM-300 (Al) shows binding of ND_3_ to the framework. 19c adopted with permission from ref 308. Copyright 2018 Wiley-VCH. (d) NH_3_ adsorption–desorption isotherms of MFM-303­(M) (M = V^IV^, Fe, Al, V^III^, Cr, and Sc), (e) MFM-300­(Al) at different temperatures, and (f) MFM-300­(V^IV^) at different temperatures. 19d–f Data source: refs 264, 308, 311, 324, 377 .

The NH_3_ adsorption capacity of MFM-300 (Cr, Sc, V^III^, Al, Fe, V^IV^) was found to be 12.5, 13.1, 13.5, 13.9, 14.4, and 15.2 mmol/g, respectively at 1 bar, 293 or 298 K (Figure 19d). Though the NH_3_ capacity of MFM-300­(Al) is lower than the best-performing porous materials (Table 4), it achieved a high adsorption–desorption reversibility and high framework stability over 50 cycles under pressure-swing adsorption conditions (0–1 bar under dynamic vacuum <1 h).^308^ Among the MFM-300 (M) series, MFM-300 (Al) and MFM-300 (V^IV^) have high stability and adsorption–desorption reversibility at elevated temperatures (Figure 19e and 19f).^264^

In situ neutron powder diffraction studies of MFM-300 (Al, Fe, V^III^, Cr) reveal three distinct binding sites (I, II, and III), forming multilayer intermolecular hydrogen bonds with hydroxyl groups upon NH_3_ adsorption (Figure 19c). The bridging metal hydroxide present in MFM-300­(Al) is the most favorable adsorption site (Site I) with an occupancy of 0.736(6) (O→N1 = 2.84(1)), while secondary adsorption sites (Site II and Site III) exhibit occupancies of 0.236(3) (N1→N2 = 3.63(1)) and 0.213(5) (N2→N3 = 3.09(1)), respectively, in the creation of a cooperative (ND_3_)_∞_ network.^308^ Neutron diffraction and EPR studies were conducted to assess the host–guest charge transfer properties of MFM-300­(V^IV^) before and after the loading of NH_3_. These results reveal that upon loading of a higher concentration of NH_3_ (MFM-300 V^IV^·3.9NH_3_), the oxidation state of the V^IV^ centers in MFM-300­(V^IV^) undergoes reduction (from 3.70 to 3.52), accompanied by the oxidation of NH_3_ to N_2_H_4_ (V^IV^···NH_3_→V^III^···NH_2_–NH_2_). However, no substantial change of oxidation state was observed at low NH_3_ concentration (3.7, 3.67, 3.62 for 0.2NH_3_, 1.1NH_3_, and 2.5NH_3_ for formula units). These results indicate that charge transfer between adsorbed NH_3_ molecules and the V^IV^ center occurs only when the NH_3_ loading reaches a sufficiently high level (>3.9NH_3_).

Subsequently, in 2021, Lyu *et al*. further investigated the roles of each distinct binding site that facilitates NH_3_ adsorption in MFM-300­(Al, Sc) using DFT calculations and DRIFT spectroscopy.^311^ At 298 K, the NH_3_ adsorption capacity of MFM-300­(Sc) is 13.1 mmol/g at 1 bar (Figure 19f), with a high isosteric heat of adsorption (*Q*_st_) averaging ∼ 48.3 kJ/mol. Classical force field-based adsorption simulations predict an adsorption enthalpy of 36 kJ/mol, while DFT calculations suggest an adsorption energy of 40 kJ/mol for the μ-OH binding site at different NH_3_ loadings (0–1 bar). These calculated values underpredict the experimental values, and the GCMC simulated adsorption isotherm for MFM-300­(Sc) rises gradually compared to the experiment in the low pressure region. These data suggest distinct strong adsorption sites for NH_3_ in MFM-300 (M). A climbing image nudged elastic band (CI-NEB) analysis revealed the presence of an intermediate state where an NH_3_ molecule relocates from the μ-OH site to a neighboring Sc atom, elongating one Sc–O bond from 2.14 to 2.22 Å, leading to the formation of a dangling oxygen atom that induces hydrogen bonding with the adjacent μ-OH group (O–H distance of 1.88 Å). In contrast, analogous investigations of MFM-300 (Al) suggest that NH_3_-triggered bond dynamics are absent for this Al-based MOF, leading to distinct adsorption isotherm behaviors are exhibited for MFM-300 (Sc) and MFM-300 (Al) at low pressure.^308,311^

In 2019, Farha and co-workers reported a series of porphyrin-based high-valent MOFs (M-PMOF/M_2_(OH)_2_(TCPP), M = Al, Ga, In) containing Bro̷nsted acidic sites, which were synthesized using meso-tetra­(4-carboxyl-phenyl) porphyrin (TCPP) linkers and various metal salts (Al, Ga, In).^315,324^ The resulting framework structure consists of rod-packing chains interlinked by a free-base porphyrin linker and bridging hydroxyl groups (μ-OH), forming a one-dimensional framework with a pore size of 1.0 nm (Figure 20a). These μ-OH groups are pointed toward the pore channels and act as Bro̷nsted acidic sites for NH_3_ adsorption. (Figure 20b and 20b). To evaluate the NH_3_ sorption capacity and reversibility of the M-PMOFs (Al, Ga, In), two consecutive NH_3_ sorption cycles were performed without any further activation. The NH_3_ adsorption capacity of Al-PMOF was found to be 7.67 mmol/g for the first cycle and 7.34 mmol/g for the second cycle at 298 K and 1 bar (Figure 20c). Ga-PMOF demonstrated uptake capacity of 10.50 then 7.71 mmol/g, and In-PMOF displayed 9.41 then 7.83 mmol/g for the first and second cycles, respectively.^315^ The diminished NH_3_ uptake capacity in Ga- and In-PMOFs was attributed to deterioration of their framework integrity after the first adsorption cycle based on powder-XRD. However, Al-PMOF retained its crystallinity and NH_3_ adsorption capacity under the same conditions.

**Figure 20:**
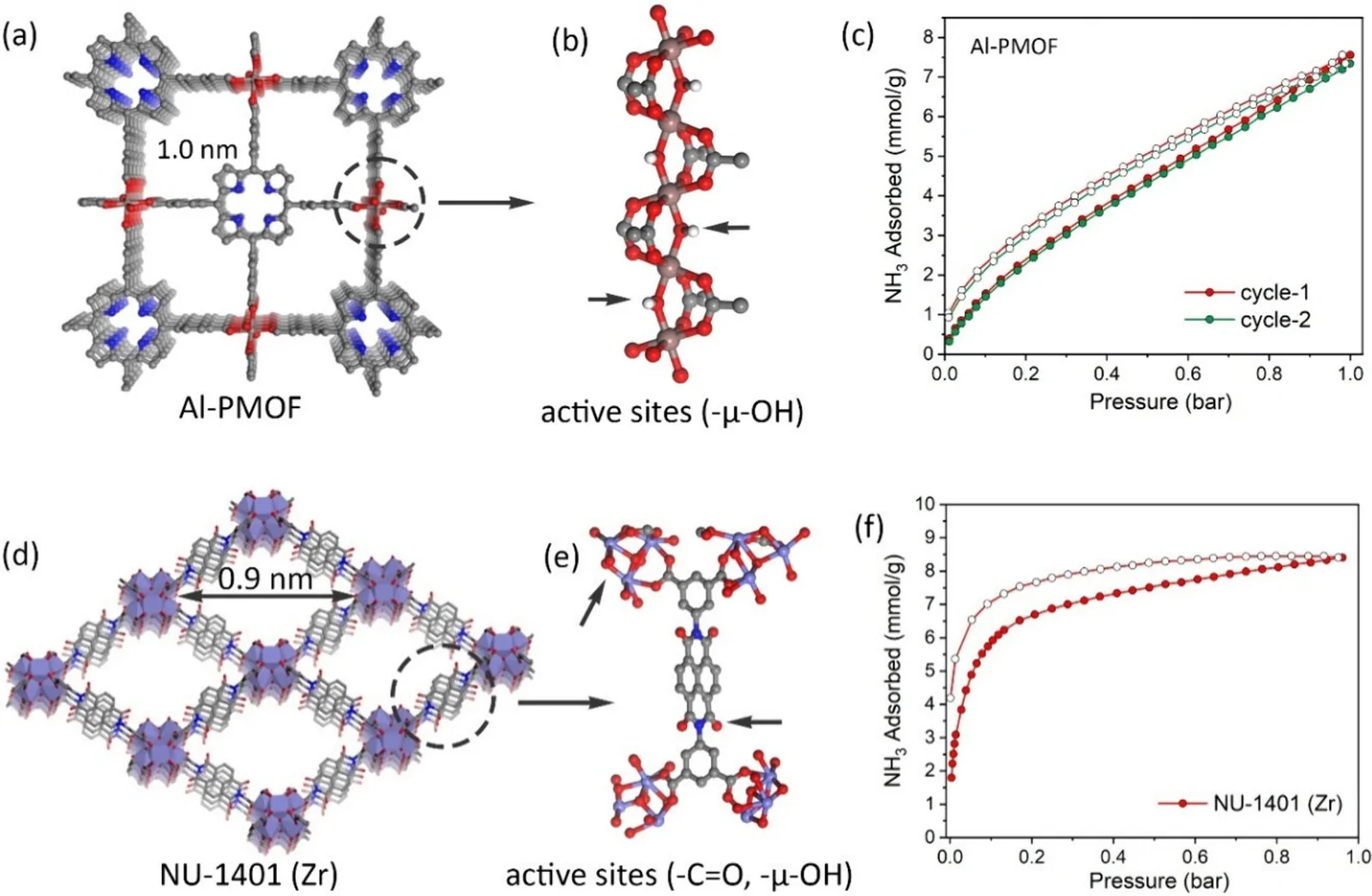
(a) Structure of porphyrin M-PMOF (M= Al, Ga, and In) showing the 1 nm 1D pores along the *b*-axis. (b) Bro̷nsted acidic bridging hydroxyl (μ-OH) in Al-PMOF. (c) NH_3_ adsorption–desorption isotherms of Al-PMOF, at 298 K for the first and second adsorption–desorption cycles. Data source: ref 315. (d) Crystal structure of NU-1401 (Zr) showing the 0.9 nm pores along the *a*-axis. (e) Bro̷nsted acidic bridging hydroxyl (μ-OH) functional groups in NU-1401­(Zr). (f) NH_3_ adsorption–desorption isotherm of NU-1401­(Zr) at 298 K. Data source: ref 361.

Diffuse reflectance infrared Fourier transform spectroscopy (DRIFTS) analysis was performed on bulk MOF powders before and after NH_3_ dosing to probe the binding characteristics and strengths of the bridged hydroxyl (μ-OH) sites. Upon NH_3_ exposure at 323 K, changes in peak intensities at 3700, 3659, and 3635 cm^–1^ for Al-, Ga-, and In-PMOFs, respectively, indicated the consumption of μ-OH groups via NH_4_^+^ formation.^297,315^ Concurrently, peaks corresponding to NH_4_^+^ cations were observed at 1385, 1365, and 1350 cm^–1^ for Al-, Ga-, and In-PMOFs, confirming the presence of Bro̷nsted acidity in all three frameworks. The higher μ-OH stretching frequency in Al-PMOF indicates a stronger Al–OH bond relative to the Ga–OH and In–OH bonds. This stronger metal μ-OH bond in Al-PMOF possibly contributes to the framework stability after NH_3_ adsorption, as supported by PXRD results, whereas the weaker M–OH bonds in Ga- and In-PMOFs are more susceptible. These results highlight the critical influence of bridging hydroxyl bond strength and associated acidity on the reversibility and NH_3_ adsorption.

The adsorption capacity of Al-PMOF was further boosted by loading active additional Bro̷nsted acidic sites, such as HCl and HCOOH, into the pores of the framework through acid vapor diffusion.^379^ Thermogravimetric analysis (TGA) showed that HCl-infused MOFs (Al-PMOF-HCl) contain 6.5 HCl molecules and 15.3 H_2_O molecules per porphyrin linker, while formic acid-infused MOFs (Al-PMOF-FA) contain 6.4 HCOOH molecules and 4.0 H_2_O molecules per porphyrin linker. Breakthrough results revealed that NH_3_ breakthrough occurred after 133 min (0% RH), 226 min (80% RH) for Al-PMOF-HCl and after 110 min (0% RH), 159 min (80% RH) for Al-PMOF-FA. Notably, both Al-PMOF-HCl and Al-PMOF-FA outperformed the as-made Al-PMOF (13.5 min at 0% RH and 25 min at 80% RH) and BPL-activated carbon (7 min at 0% RH and 13 min at 80% RH) under the same conditions.^379^ The favorable hydrogen bonding sites for NH_3_ may result from a high density of Bro̷nsted acidic groups (HCl and HCOOH) loaded in the pores. However, the authors have not provided NH_3_ adsorption–desorption isotherms and evidence to confirm whether chemical adsorption (e.g., Al-PMOF-HCl + NH_3_ → Al-PMOF-NH_4_Cl; Al-PMOF-COOH + NH_3_ → Al-PMOF-COONH_4_) or physical adsorption is occurring (e.g., Al-PMOF-HCl.NH_3_).

Subsequently, in 2020, NU-1401 (Zr) was synthesized using ZrOCl_2_·8 H_2_O, and 4,5,8-naphthalenediimide (NDI)-based carboxylate linkers and it was tested for NH_3_ adsorption.^361^ The crystal structure of NU-1401 (Zr) shows the presence of Bro̷nsted acidic bridging hydroxyl (μ-OH) groups between metal centers, and electron-deficient naphthalene diimide (NDI) carbonyl groups pointing toward the pores (Figure 20d and 20e). The interpenetrated structure of NU-1401­(Zr) generated 0.9 nm pores along the *a*-axis. NH_3_ adsorption isotherms of NU-1401 show type-1 isotherms with steep uptake at low pressure and reaching the total uptake capacity of 8.41 mmol/g at 298 K (Figure 20f). Powder-XRD patterns of NU-1401, after NH_3_ adsorption, match well with pristine MOFs. Both DRIFTS and UV–vis spectroscopy studies reveal that the adsorption mechanism of NU-1401 is similar to that observed in the Bro̷nsted acidic Al-PMOF. However, multiple cycles of adsorption are not reported for NU-1401 (Zr).

Another approach, the pore anchoring of Bro̷nsted acidic functional groups, has also been tested. In 2019, a Zr-based MOF (NU-300) containing uncoordinated free-carboxylate (−COOH) groups within the framework was synthesized and evaluated for NH_3_ adsorption applications (Figure 21a–c).^380^ The asymmetric unit of NU-300 (Zr) contains four Zr^4+^ atoms, each uniquely eight-coordinated. The 3,5-di­(4′-carboxylphenyl)­benzoic acid (H_3_L) ligand has two types of coordination modes with Zr-ion, with one carboxylate group (−COOH) remaining uncoordinated and pointing to the channel along the *a*-axis in the 3D structure of NU-300 (Zr).^380^ NH_3_ adsorption isotherms of NU-300 displayed type-1 microporous characteristics, with an initial uptake capacity of 8.28 mmol/g at 298 K and 1.0 bar for the first cycle (Figure 21c). However, adsorption uptake significantly decreased in the second cycle (5.71 mmol/g) and plateaued for the third cycle (5.41 mmol/g). The steep adsorption at low pressure and the microporous nature indicate that NH_3_ molecules have strong interactions with the Bro̷nsted acidic acid (−COOH) groups, which are challenging to break under reduced pressure or ambient conditions. FT-IR studies further revealed that NH_3_ molecules in MOFs are protonated with Bro̷nsted acidic sites (−COOH), which persist even after purging with argon gas. Similarly, other MOFs containing 1–3 uncoordinated acid (−COOH) groups were further investigated using breakthrough analysis by comparing ZnBTTB (two free groups, −(COOH)_2_), CuBTD (one free group, −(COOH)_1_), and DMOFs (no free groups, −COOH) MOFs.^272^ Breakthrough uptake capacity of these MOFs increases with increasing the Bro̷nsted acidic sites in the MOFs, i.e., ZnBTTB > CuBTD > DMOF with 4.59, 2.19, and 0.27 mmol/g at 0% RH, respectively.^272^ The adsorption mechanisms of these MOFs follows the same as NU-300 (Zr).

**Figure 21:**
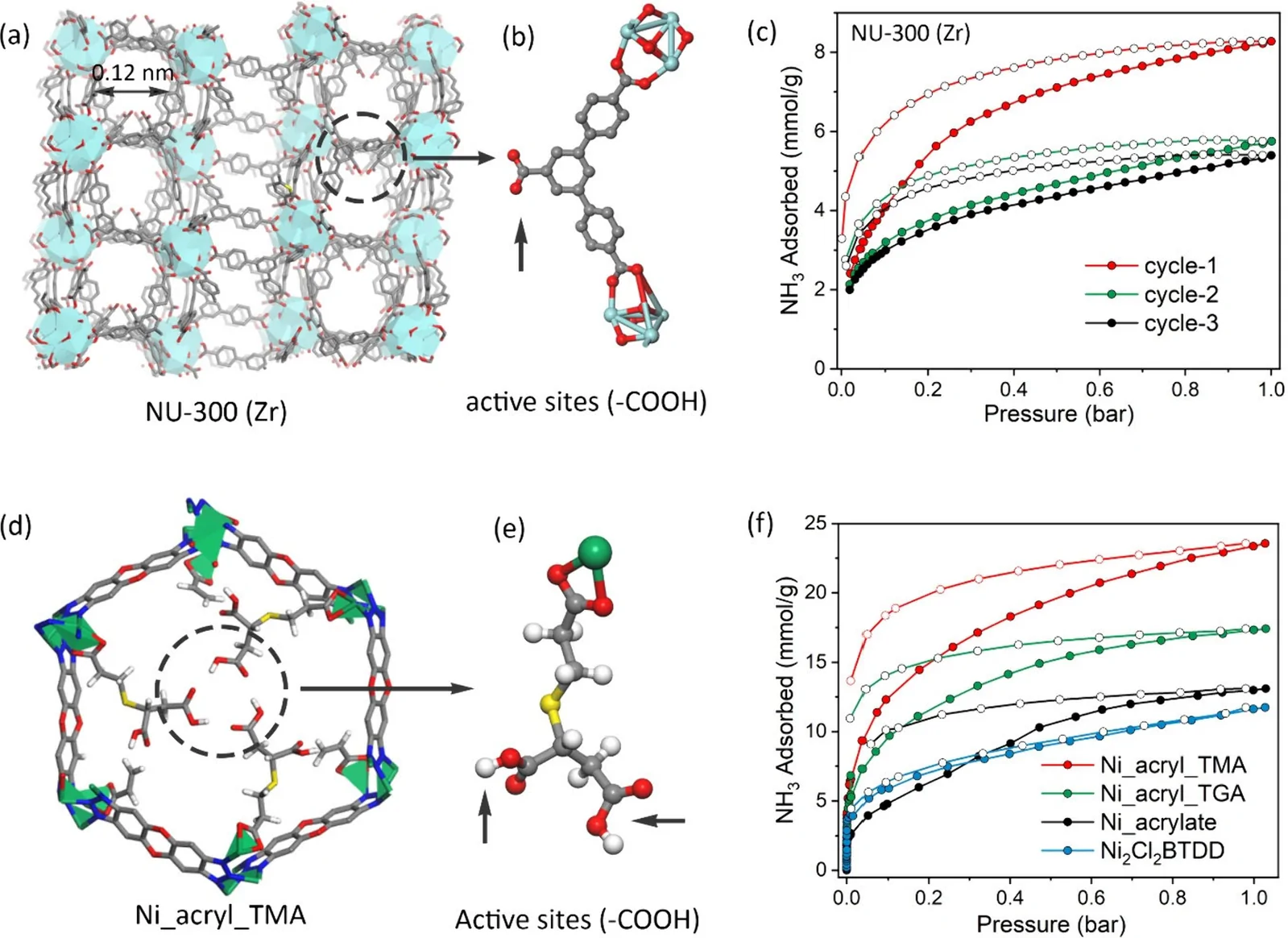
(a) Structure of NU-300 (Zr) illustrates the presence of uncoordinated carboxylic acid (−COOH) groups in the pore. (b) Bro̷nsted acidic carboxylic acid (−COOH) functional groups in NU-300 (Zr). (c) NH_3_ adsorption–desorption isotherms in NU-300 (Zr) at 298 K over three consecutive cycles. Data source: ref 380. (d) Schematic illustration of thiomalic acid (TMA) grafted in Ni_2_Cl_2_BTDD; uncoordinated carboxylic acid ((−COOH)_2_) functional groups pointing toward the pores. (e) Thiomalic acid (−SCH­(COOH)­(CH_2_COOH)) sites of Ni-acryl_TMA. (f) NH_3_ adsorption–desorption isotherms of pristine and carboxylic acid-grafted MOFs (Ni_2_Cl_2_BTDD) at 298 K. Data source: ref 263.

In 2022, Kim *et al*. performed a double postsynthetic modification approach to graft the uncoordinated carboxylic acids (thioglycolic acid (−S–COOH/TGA) and thiomalic acid (−SCH­(COOH)­(CH_2_COOH)/TMA)) into the MOF-Ni_2_Cl_2_BTDD (Figure 21d and 21e).^263^ The extent of acid functionalization and stability of the framework were assessed through PXRD, TGA, and elemental analysis. These analytical studies revealed that Ni_acryl_TMA possesses 1.52 times more carboxylic groups (−COOH) than Ni_acryl_TGA. Adsorption studies revealed that the acid-rich Ni_acryl_TGA and Ni_acryl_TMA MOFs exhibit high NH_3_ uptake and reversibility over pristine Ni_2_Cl_2_BTDD. At 298 K, the NH_3_ adsorption of Ni_2_Cl_2_BTDD is 11.70 mmol/g, while Ni_acryl_TMA and Ni_acryl_TGA grafted MOFs have 23.5 and 17.41 mmol/g under the same conditions (Figure 21f). Ni_acryl_TMA retained ∼ 98% of its initial NH_3_ capacity after 5 adsorption cycles upon treating the Ni_acryl_TMA after NH_3_ adsorption under high vacuum for 2–12 h (temperature not given). Breakthrough experiments reveal that pristine Ni_2_Cl_2_BTDD has an NH_3_ uptake of 3.36 mmol/g, while acid-grafted Ni_acryl, Ni_acryl_TGA, and Ni_acryl_TMA exhibit uptake capacities of 1.97, 3.09, and 4.11, mmol/g, respectively, at 298 K and 0% RH. At 80% RH, the NH_3_ uptake capacities for Ni_2_Cl_2_BTDD, Ni_acrylate, Ni_acryl_TGA, and Ni_acryl_TMA are 3.09, 1.32, 2.83, and 2.98 mmol/g, respectively. The decrease in total uptake capacity under wet conditions is attributed to water adsorption.^263,270^

Temperature-programmed desorption (TPD) studies demonstrated a sharp decrease in desorption temperature from 477 K (pristine MOF) to 373 K (Ni_acryl_TGA), and 345 K (Ni_acryl_TMA).^263^ This notable temperature reduction suggests that the grafted acid functional groups (−COOH) exhibit weaker interactions with NH_3_ molecules than the open metal sites do.^263^ It is important to note that although the desorption temperature decreases significantly, there is a distinct difference in the adsorption capacity and desorption temperature between Ni_acryl_TGA and Ni_acryl_TMA. This difference possibly arises from the varying numbers of carboxylic acids and the bulkiness of the functionalized moiety, which affects NH_3_ binding and desorption temperature.

Within Bro̷nsted acidic MOFs, the isostructural MFM-300­(M) (M = Cr, Sc, V^III^, Al, Fe, V^IV^)) shows a systematic increase in NH_3_ uptake from ∼ 12.5 (Cr) to 15.2 mmol/g (V^IV^) as the metal center changes, driven by progressively stronger μ-OH acidity and enhanced hydrogen-bonding cooperativity within the same topology.^264,308,311,324,377^ At the same time, framework stability depends strongly on the robustness of the metal–oxygen bonds and redox behavior, with redox-inert Al-based systems showing the highest structural resistance to NH_3_, while early transition-metal analogs (Cr, Fe, and V-based systems) exhibit comparatively lower stability due to their redox activity. A similar but opposite trend is observed in porphyrin-based M-PMOFs, where differences in M–OH bond strength (Al > Ga > In) primarily control structural robustness and cyclic reversibility, rather than significantly altering NH_3_ uptake capacity; weaker metal–oxygen bonds lead to framework degradation upon cycling.^300^ In the case of carboxyl- and hydroxyl-functionalized MOFs such as NU-1401 and NU-300, NH_3_ adsorption is strongly driven by Bro̷nsted acidic sites, particularly at low pressure.^361,380^ However, when these acid sites are very strong or densely distributed, some of the adsorbed NH_3_ is converted to NH^4+^ and becomes trapped within the pores, limiting complete desorption and reducing adsorption–desorption reversibility. This trade-off becomes more pronounced in postsynthetically modified acid-enriched Ni_2_Cl_2_BTDD (Ni_acryl_TMA and Ni_acryl_TGA) MOF derivatives, where increasing Bro̷nsted site density significantly enhances capacity (∼2–3-fold) compared to the parent framework, but simultaneously strengthens chemisorption and increases the regeneration energy requirements.^263^

### 3.5 MOF-Based Composite Materials

MOF composites are hybrid materials formed by the integration of MOFs with other materials such as polymers, graphene derivatives, metal salts, ionic liquids, nanoparticles, etc., under various synthesis conditions.^381,382^ The aim of using these MOF composites is to synergistically combine the strengths of both materials to enhance their overall performance.^132^

In 2009, Bandosz and Petit investigated the adsorption properties of GO-MOF composite materials by incorporating various concentrations of graphene oxide (GO) into MOFs (MIL-100­(Fe), MOF-5, and HKUST-1).^383–385^ NH_3_ adsorption studies revealed that increasing the GO content in the composite led to a gradual increase in NH_3_ adsorption capacity: 4.81 mmol/g for 50% GO-MOF5, 1.6–3.5 mmol/g for GO-MIL-100­(Fe), 14.5 mmol/g for GO-HKUST-1 (MGO2), and 10.44 mmol/g at 298 K for adsorption HKUST-1. None of these composites have higher NH_3_ uptake capacity than that of their physical mixtures of MOF and GO (MOFs (1) + GO (1’) mixing = MOF.GO (1:1’) physical mixtures) indicating that these approaches and composite materials could not achieve synergistic performance.

Ionic liquids possess Lewis acidic sites and polar functional groups, making them promising candidates for selective NH_3_ adsorption.^386^ Integration of these ionic liquids (ILs) into MOFs has recently garnered considerable attention.^286,290^ In 2020, Zhong and co-workers incorporated the ionic liquids (1-(4-hydroxy-butyl)-3-methylimidazolium chlorozincate ([BOHmim]­[Zn_2_Cl_5_]), and carbamide chloride ([CAM]­[Cl]) into MIL-101­(Cr) using solution-based impregnation methods (Figure 22a and 22b).^286,314^ Adsorption isotherms of [BOHmim]­[Zn_2_Cl_5_]@MIL-101­(Cr) and [CAM]­[Cl]@MIL-101­(Cr)-30% composites exhibited 24.1 and 11.6 mmol/g of NH_3_ uptake capacity at 298 K, respectively, which is 2.7 and 1.3 times higher than that of pristine MIL-101­(Cr) (8.92 mmol/g) under the same conditions (Figure 22d and Figure 15b). Quantum chemical calculations were employed to assess the active sites and NH_3_ interactions with the ILs-MOF composite materials.^314^ The host–guest interaction energy for NH_3_ and CO_2_ was obtained for the most stable geometric configuration of NH_3_-[CAM]­[Cl] (−10.58 kcal/mol) and CO_2_-[CAM]­[Cl] (−5.16 kcal/mol). The stronger interaction energy for NH_3_-[CAM]­[Cl] was attributed to NH_3_ hydrogen bonding to the ionic liquid, compared to van der Waals interactions within CO_2_-[CAM]­[Cl]. Notably, the NH_3_ adsorption capacity of [BOHmim]­[Zn_2_Cl_5_]@MIL-101­(Cr) (23.55 mmol/g) has been maintained under humid conditions (20% RH), although full reversibility was not achieved during desorption isotherms. However, the total uptake capacity was retained over 5 cycles upon activation of the composite at 373 K under vacuum. On the other hand, small capacity was observed for O_2_ (0.59 mmol/g) or CO_2_ (0.82 mmol/g) gases under ambient conditions. The IAST selectivities for NH_3_/O_2_ and NH_3_/CO_2_ were found to be 40.7 and 29.3 at 298 K, respectively.

**Figure 22:**
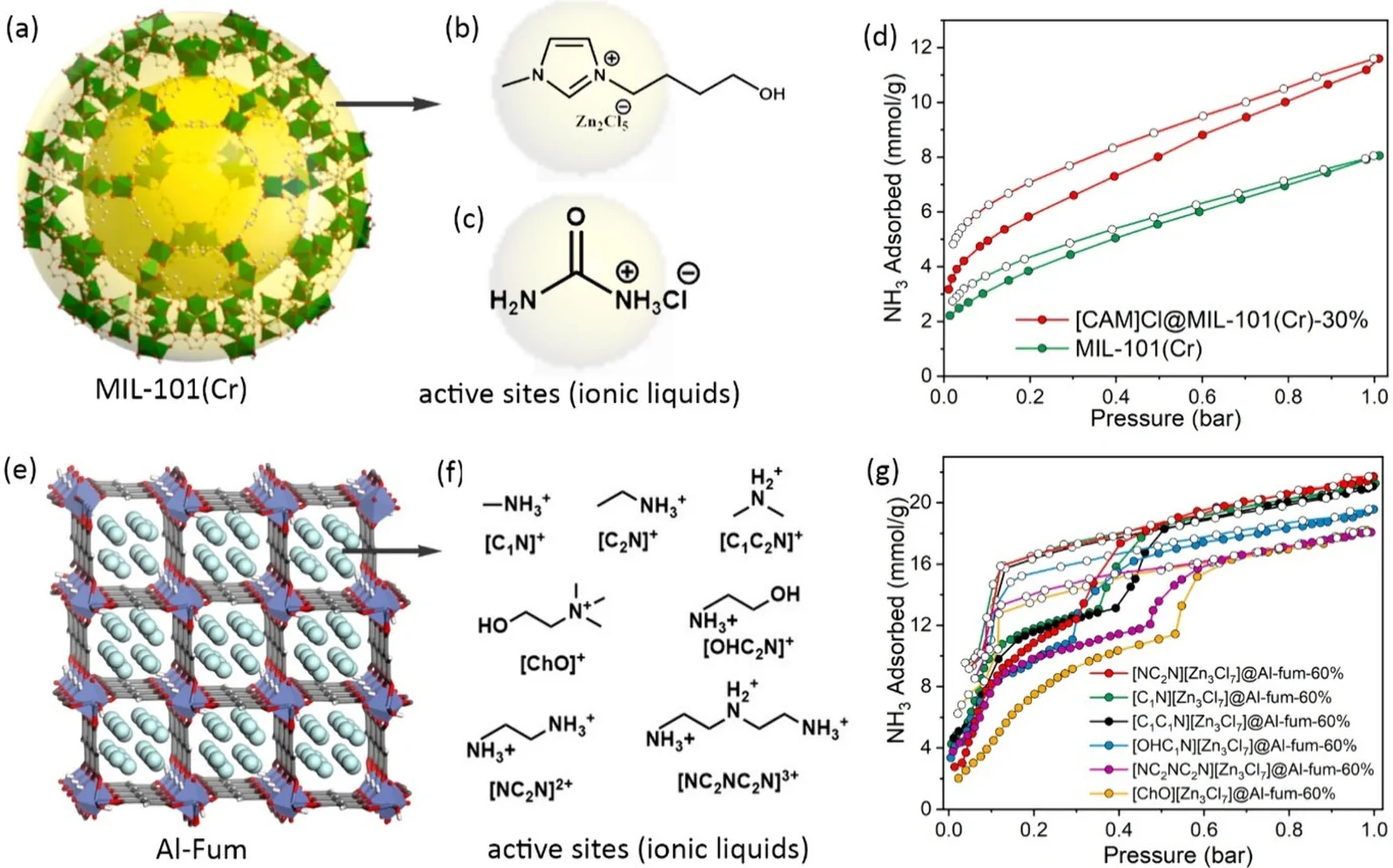
(a) Schematic illustration and graphical view of the [CAM]­[Cl] or ([BOHmim]­[Zn_2_Cl_5_] ionic liquids embedded in MIL-101­(Cr). Adopted with permission from ref 314. Copyright 2023 Elsevier. (b) Chemical structure of ionic liquid employed for the [BOHmim]­[Zn_2_Cl_5_]@MIL-101 composite preparation. (c) Chemical structure of [CAM]­[Cl] ionic liquid employed for the [CAM]­[Cl]@MIL-101­(Cr)-30% composite preparation. (d) NH_3_ adsorption–desorption isotherms of MIL-101­(Cr) and [CAM]­[Cl]@MIL-101­(Cr) at 298 K. Data source: ref 314. (e) Schematic illustration of the ZnIL@Al-Fum-x composite. (f) Chemical structures of ionic liquids employed for the preparation of the ZnIL@Al-Fum-x composite. (g) NH_3_ adsorption–desorption isotherms of Al-Fum and ZnIL@Al-Fum-60 composites at 298 K, with varying cation sizes of ionic liquids ([C_2_N]^+^, [NC_2_N] ^2+^, [NC_2_NC_2_N]^3+^) while maintaining the same anion ([Zn_3_Cl_7_]^x^, x = 1–3). Data source: ref 290.

The interactions between NH_3_ and [BOHmim]­[Zn_2_Cl_5_]@MIL-101­(Cr) composites were further investigated through FT-IR and XPS analysis, comparing the composite materials before and after NH_3_ saturation. FT-IR analysis revealed two peaks at 3344 and 1255 cm^–1^ for IL@MIL-101­(Cr) after NH_3_ adsorption, attributed to the vibrational modes of ν­(N–H) and δ­(Zn–NH_3_), respectively.^286^ Additionally, the intensities of ν­(−OH) and ν­(C–O) characteristic peaks at 3145 and 1099 cm^–1^ increased, indicating the formation of hydrogen bonds between the hydroxyl-functionalized alkyl chains and NH_3_. The binding energies of the Zn 2p peaks decreased upon NH_3_ adsorption (with a deviation of 0.3 eV), suggesting strong interactions between Zn^2+^ in [Zn_2_Cl_5_]^−^ and NH_3_ molecules.

Subsequently, a series of amine-rich Zn-based ionic liquids were integrated into A-Fum MOFs through solution impregnation methods (Figure 22e and 22f).^290^ These included alkyl-amine heptachlorotrizincates ([alkyl-amine]­[Zn_3_Cl_7_]_3_), ethylamine trichlorozincate ([C_2_N]­[ZnCl_3_]), and ethylamine pentachlorodizincate ([C_2_N]­[Zn_2_Cl_5_]), etc. The resulting hybrid materials (ZnIL@Al-Fum-x) have been investigated for NH_3_ adsorption across a range of temperatures (298–373 K). The NH_3_ uptake of [C_2_N]­[Zn_3_Cl_7_]@Al-fum-x (x = 20, 40, 60 wt %) was found to be 12.3, 21.5, and 22.3 mmol/g, respectively at 298 K and 1.0 bar, which is significantly higher than that of pristine Al-Fum (10.4 mmol/g) under the same conditions (Figure 22g).^290^ Furthermore, the hybrid material [C_2_N]­[Zn_3_Cl_7_]@Al-Fum-60% exhibited NH_3_ uptakes of 9.9 mmol/g at 353 K and 8.8 mmol/g at 373 K, both at 1 bar. At 353 K. Cycling experiments revealed that [C_2_N]­[Zn_3_Cl_7_]@Al-fum-x decreased by ∼ 20% of its initial capacity after the first cycle, which remained consistent for the next 3 cycles. Notably, [C_2_N]­[Zn_3_C_l7_]@Al-fum-x exhibited a gate-opening behavior at 298 K (∼0.55 bar), which is shifted to higher values (∼0.7 bar) at 353 K.^290^

The influence of cation entity in the ionic liquids was further investigated by altering the cations from small to large size ([C_2_N]^+^, [NC_2_N]^2+^, [NC_2_NC_2_N]^3+^) while preserving the same anion ([Zn_3_Cl_7_]_*x*_^–^ for x = 1–3) in the composite. The NH_3_ adsorption capacities of [C_2_N]­[Zn_3_Cl_7_]@Al-Fum-60%, [NC_2_N]­[Zn_3_Cl_7_]_2_@Al-Fum-60%, and [NC_2_NC_2_N]­[Zn_3_Cl_7_]_3_@Al-Fum-60% were found to be 22.3, 21.7, and 18.1 mmol/g, respectively at 298 K.^290^ This gradually decreasing trend reveals that increasing the cation size in the composite decreases the total NH_3_ adsorption capacity. This phenomenon could be attributed to a smaller accessible volume for NH_3_ for the larger cationic liquids over small ones (the number of Zn centers are constant in all). XPS and FT-IR analysis after NH_3_ adsorption revealed that all these IL@Al-Fum have two kinds of interactions with NH_3_ (NH_3_–Zn^2+^ coordination interactions and NH_3_ hydrogen bonding interactions with Cl, H of the IL, and μ-OH of Al-Fum), just like [BOHmim]­[Zn_2_Cl_5_]@MIL-101­(Cr) composites.^286^

MOF–metal halide composites (MCl@MOFs) are another emerging class of composite materials that have attracted considerable attention recently due to their high sorption affinity and selectivity for NH_3_ over N_2_ and H_2_. In 2022, Shi *et al*. integrated LiCl salts into Al-MOFs using impregnation methods and obtained different compositions of LiCl@MIL-53-(OH)_2_-x, (x = 7.6 to 49.2% mass percentage of LiCl) (Figure 23a).^271^ NH_3_ adsorption isotherms show that LiCl@MIL-53-(OH)_2_-43.4 has 33.9 mmol/g of uptake capacity at 298 K and 1.0 bar, which surpassed the adsorption capacities of both the pristine MIL-53-(OH)_2_ (7.3 mmol/g) and neat LiCl (2.1 mmol/g) under the same conditions (Figure 23b). The NH_3_ adsorption profile reveals a brief plateau at low pressure, achieving 11.3 mmol/g of uptake at 0.1 and a rapid rise at 0.17 bar, and reaching the capacity of 27.8 mmol/g at 0.2 bar (Figure 23b). Notably, LiCl@MIL-53-(OH)_2_-43.4 exhibits 12.2 mmol/g of NH_3_ uptake capacity at 353 K and showed good breakthrough separation for NH_3_ (NH_3_/N_2,_ 0.4/99.6) with a separation coefficient of 3571 for NH_3_/N_2_ at 298 K.^271^ The composite retained 95% of its initial NH_3_ adsorption capacity after 15 cycles, and the residual amount of nondesorbed NH_3_ was removed by activating the composite under vacuum at 373 K for 2 h. The high adsorption capacity at low pressures and the rapid increase in NH_3_ capacity at higher pressures are attributed to the synergistic interplay of the micropore filling effect and the increased surface interactions from LiCl in LiCl@MIL-53-(OH)_2_-43.4.

**Figure 23:**
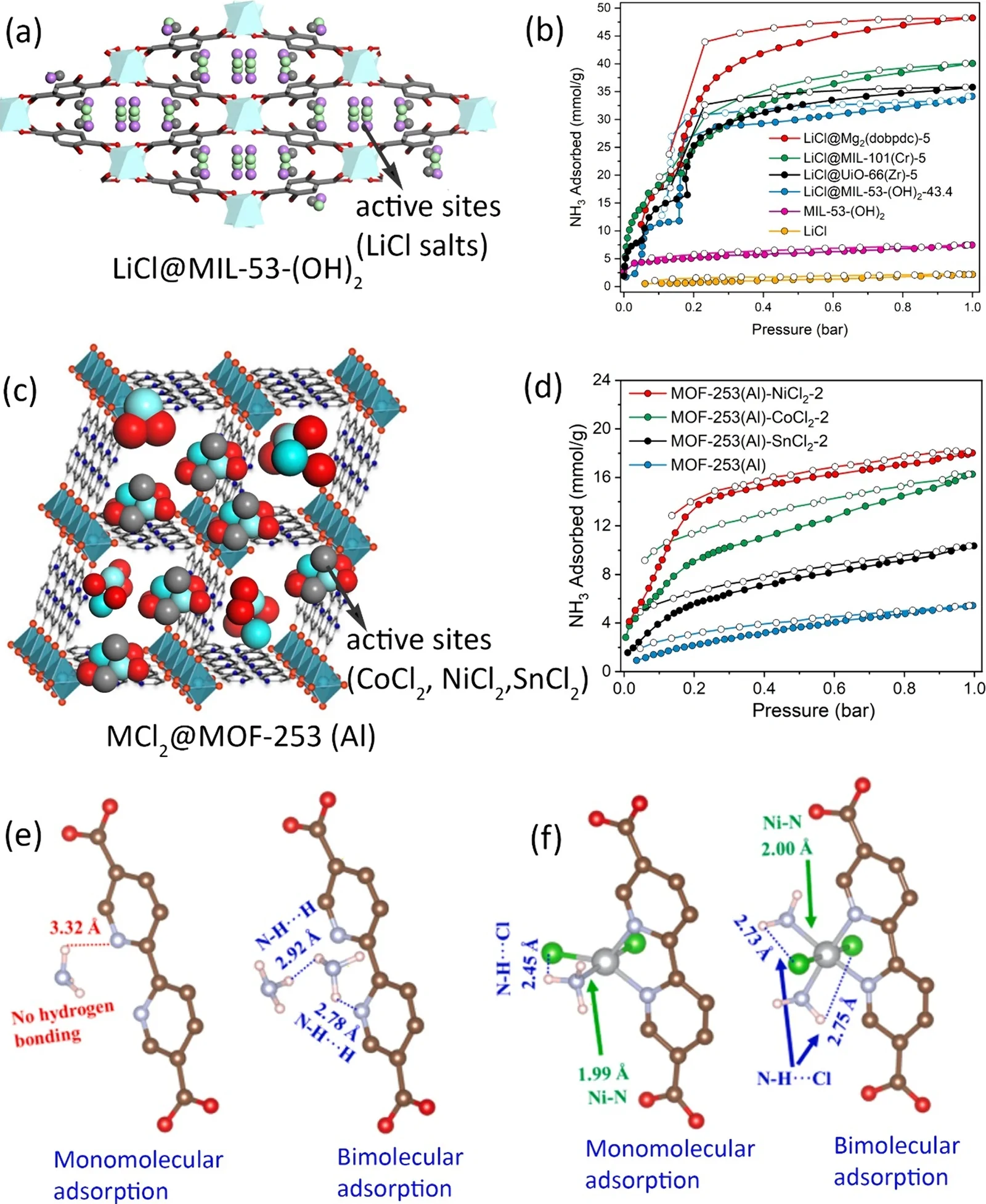
(a) Schematic illustration of LiCl@MIL-53-(OH)_2_ composite. (b) At 298 K, NH_3_ adsorption–desorption isotherms of LiCl, MIL-53-(OH)_2_, and LiCl@MIL-53-(OH)­2–43.4 (43.4 indicates mass percentage of LiCl present in the composite) Mg_2_(dobpdc)-5, LiCl@MIL-101­(Cr)-5, and LiCl@UiO-66­(Zr)-5 (5 indicates the molar concentration of LiCl). Data source: refs 132, 271 . (c) Schematic illustration of metal halides loaded in MOFs-253­(Al) (MCl_2_@MOF-253­(Al) M = Co, Ni, and Sn). (d) NH_3_ adsorption–desorption isotherms of MOF-253­(Al) and MCl_2_@MOF-253­(Al) composites at 298 K. Data source: ref 56. (e) DFT optimized adsorption configuration of NH_3_ in MOF-253­(Al) shows the effective hydrogen bonding interactions: when each MOF-253­(Al) unit is accommodating one NH_3_ molecule (no H-bonding) and two NH_3_ molecules (weak of hydrogen bonding interactions). (f) MOF-253­(Al)-NiCl_2_ unit, with one NH_3_ molecule and two NH_3_ molecules, indicating the presence of hydrogen bonding interactions. 23e and 23f adopted with permission from ref 56. Copyright 2023 Elsevier.

The active adsorption sites in the LiCl@MIL-53-(OH)_2_-x composites were investigated using electronic absorption spectra, DFT, XPS studies, and FT-IR analysis. The FT-IR spectra of the LiCl@MIL-53-(OH)_2_-x composites showed varying degrees of blue-shifted stretching peaks associated with μ-OH and COO^–^, indicating a potential electron transfer between LiCl and the framework. Additionally, the stretching vibration of the phenolic −OH group exhibited a significant redshift from 3348 to 3337 cm^–1^, coupled with peak broadening, suggesting the formation of hydrogen bonds between Cl of LiCl and H of phenolic −OH on the framework. The XPS spectra of LiCl@MIL-53-(OH)_2_-43.4 before and after NH_3_ adsorption reveal a decrease in the Li 1s binding energy from 55.80 to 55.47 eV, indicating the coordination of NH_3_ lone pair of electrons with Li^+^. The high-resolution N 1s spectrum exhibits characteristic signals at 401.6 and 402.2 eV, corresponding to coordinated NH_3_ and ammonium (NH_4_^+^). Additionally, the Cl 2p peaks, initially at 198.4 and 199.9 eV, shifted to 198.2 and 199.5 eV post-NH_3_ uptake, suggesting the formation of hydrogen bonding between NH_3_ and Cl^–^. The electronic absorption spectra revealed a pronounced blue shift in the maximum absorption peak of LiCl@MIL-53-(OH)_2_-43.4 (392 nm) compared to the parent MOF (422 nm), affirming the occurrence of charge transfer from LiCl to MIL-53-(OH)_2_.^271,387^ Furthermore, DFT-optimized differential charge analysis indicated a transfer of electrons (0.11 electrons) from LiCl to neighboring O atoms (μ-OH and COO^–^). Consequently, each Li^+^ acquired 0.03 electrons from oxygen (COO^–^ and μ-OH), while oxygen gained 0.14 electrons from Cl^–^. These findings support the anchoring of LiCl onto the MOF framework through electrostatic interactions, leading to enhanced dispersion. Following the same concept, Kim *et al*. investigated the adsorption performance of open metal site MOFs, M_2_(dobpdc) (M = Mg, Co, Mn, Ni), and large-pore MOFs, MIL-101­(Cr) and UiO-66­(Zr), after incorporation of LiCl (Figure 14i).^132^ The LiCl-loaded composites, Mg_2_(dobpdc)-5, LiCl@MIL-101­(Cr)-5, and LiCl@UiO-66­(Zr)-5 (5 indicates the molar concentration of LiCl solution) were synthesized via a wet impregnation procedure.^132,388^ The lithium contents were determined to be 58.59 wt % for LiCl@Mg_2_(dobpdc)-5, 41.33 wt % for LiCl@Mn_2_(dobpdc)-5, 37.98 wt % for LiCl@Co_2_(dobpdc)-5, and 35.10 wt % for LiCl@Ni_2_(dobpdc)-5. The corresponding Li-to-metal molar ratios were 1.43 for LiCl@Mg_2_(dobpdc)-5, 5.30 for LiCl@MIL-101­(Cr)-5, and 5.85 for LiCl@UiO-66­(Zr)-5.

Ammonia adsorption isotherms revealed that LiCl@Mg_2_(dobpdc)-5 exhibits an uptake of 48.3 mmol/g at 298 K, surpassing the best known LiCl@XJCOF-4–44.1 (44.98 mmol/g) and all the benchmark adsorbents reported to date (Figure 22d, 23b, 26d, Tables 2 and 4). Notably, the desorption isotherms of LiCl@Mg_2_(dobpdc)-5 show a decrease in NH_3_ uptake from 48.3 mmol/g to 24 mmol/g isothermally at 298 K and 1 bar. Despite this partial reversibility at reduced pressure, LiCl@Mg_2_(dobpdc)-5 is still exhibits superior overall desorption performance compared with ionic liquid-loaded and transition metal halide-grafted MOF composites under the same conditions (Figures 22g, 23b, 23d, and Table 4). Furthermore, the total uptake capacity of LiCl@Mg_2_(dobpdc)-5 was further enhanced at a lower temperature (∼80.08 mmol/g at 273 K, 1 bar). Other composites, LiCl@Co_2_(dobpdc)-5, LiCl@Ni_2_(dobpdc)-5, LiCl@Mn_2_(dobpdc)-5, LiCl@MIL-101­(Cr)-5, and LiCl@UiO-66­(Zr)-5 exhibited NH_3_ uptakes of 59.5, 42.5, 37.8, 40.1, and 35.9 mmol/g, respectively, at 298 K and 1 bar (Figure 23b). Although the total uptake capacity of LiCl@Co_2_(dobpdc)-5 is superior then the LiCl@Mg_2_(dobpdc)-5, NH_3_ adsorption–desorption cycling studies demonstrated that LiCl@Mg_2_(dobpdc)-5 has good stability over LiCl@Co_2_(dobpdc)-5 and among other analogous materials, and also retained over 95% of its initial NH_3_ uptake over five continuous adsorption–desorption cycles after activating at 373 K for 6 h.^132^ In contrast, LiCl@MIL-101­(Cr)-5 and LiCl@UiO-66­(Zr)-5 lost ∼ 20% and ∼ 15% of their capacities after the first cycle under the same activation conditions. PXRD and N_2_ adsorption isotherms of postadsorption samples further indicated a loss of their crystallinity and porosity upon repeated adsorption cycles.^132^ This suggests local pore distortions or microstructural changes in the LiCl@Mg_2_(dobpdc) composite. DFT calculations and temperature-programmed desorption studies revealed that, in LiCl@Mg_2_(dobpdc)-5, NH_3_ interacts with two distinct sites: the open Mg centers and LiCl units incorporated in the pores.^132^ By contrast, adsorption occurs solely at LiCl within the pores in LiCl@MIL-101­(Cr)-5 and LiCl@UiO-66­(Zr)-5. The superior uptake of LiCl@Mg_2_(dobpdc)-5 is thus attributed to the synergistic adsorption of well-dispersed LiCl and strong open Mg sites.

In 2023, Wang *et al.* impregnated various transition metal chlorides (NiCl_2_, CoCl_2_, or SnCl_2_) into Al-MOFs (MOF-253­(Al)) (Figure 23c).^56^ The resulting MOF-253­(Al)-MCl_2_-X composite (M = Ni, Co, Sr, and X is the molar ratio of MCl_2_ to MOF) demonstrates different NH_3_ adsorption capacities: MOF-253­(Al) (5.5 mmol/g) < MOF-253­(Al)-SnCl_2_-3 (SnCl_2_, 13.55 wt %, 9.2 mmol/g) < MOF-253­(Al)-CoCl_2_-2 (CoCl_2_,14.03 wt %, 16.9 mmol/g) < MOF-253­(Al)-NiCl_2_-2 (NiCl_2_, 13.54 wt %, 18.0 mmol/g) at 298 K and 1.0 bar (Figure 23d).^56^ Furthermore, these composites exhibit good selectivity for NH_3_ over N_2_ and H_2_, with selectivity coefficients of 5708 for NH_3_ vs N_2_ and 2320 for NH_3_ vs H_2_. However, MOF-253­(Al) grafted with NiCl_2_, CoCl_2_, or SnCl_2_ exhibits significantly lower NH_3_ adsorption than LiCl-loaded MOFs (Figure 23b,d). Furthermore, the desorption isotherms indicate a significant amount of irreversible capacity for the NiCl_2_-, CoCl_2_-, and SnCl_2_-grafted MOFs compared with the pristine material. The isosteric heat of NH_3_ adsorption (*Q*_st_) for MOF-253­(Al)-NiCl_2_-2 (45.8–47.5 kJ/mol) exceeds that of pristine MOF-253­(Al) (30.4 kJ/mol) indicating stronger chemisorptive interactions upon the integration of NiCl_2_ salts. XPS and FT-IR investigations reveal that the metal chlorides bind to the bipyridine nitrogen of the ligand in MOF-253­(Al), and the mechanism follows a similar behavior to that observed with LiCl in LiCl@MIL-53-(OH)_2_-43.4.

DFT calculations were employed to examine the charge and energy dynamics of MOF-253­(Al) and MOF-253­(Al)-NiCl_2_-2 before and after NH_3_ adsorption.^56^ The adsorption energy of pristine MOF-253­(Al) when binding to one NH_3_ and two NH_3_ molecules is found to be −0.02 eV and −0.22 eV, respectively (Figure 23e and 23f). The negligibly low adsorption energies indicate the presence of physical adsorption in the pristine MOF-253­(Al). In contrast, the adsorption energies for MOF-253­(Al)-NiCl_2_-2 with one NH_3_ and two NH_3_ molecules were found to be −1.22 eV and −2.97 eV, respectively, which are significantly lower than the physical adsorption energies observed in pristine MOF-253­(Al), and were attributed to the presence of chemical and/or hydrogen bonding interactions between Ni^2+^ and NH_3_.

A comparative analysis reveals that, within MOF-based composite materials, NH_3_ adsorption is governed by the chemical nature of the incorporated phase (e.g., GO, ionic liquids, or metal salts), its specific interactions with the framework (e.g., coordination or hydrogen bonding), and the formation of additional binding sites.^56,132,271,381,382^ In GO@MOF systems (e.g., GO–MOF-5 (4.81 mmol/g) and GO–HKUST-1 (14.5 mmol/g), graphene oxide primarily increases surface polarity and introduces weak adsorption sites; however, the interactions remain largely physisorptive, resulting in only marginal improvements in NH_3_ uptake.^383–385^ In contrast, IL@MOF composites such as [BOHmim]­[Zn_2_Cl_5_]@MIL-101­(Cr) (24.1 mmol/g), [CAM]­[Cl]@MIL-101­(Cr) (11.6 mmol/g), and ZnIL@Al-Fum-60 (22.3 mmol/g) show moderate enhancement (up to ∼ 2.7-fold) due to the introduction of well-dispersed polar and Lewis acidic sites within the pores.^286,314^ These systems enable stronger NH_3_ binding via hydrogen bonding and coordination interactions with Zn^2+^, Cl^–^, resulting in higher capacities than those of pristine MOFs. Metal salt–impregnated composites, including LiCl@MIL-53-(OH)_2_ (33.9 mmol/g), LiCl@Mg_2_(dobpdc) (48.3 mmol/g), and MOF-253­(Al)-NiCl_2_ (18.0 mmol/g), exhibit more substantial enhancement (∼3–5 fold) due to the ability of metal ions to coordinate multiple NH_3_ molecules.^56,132,271^ However, performance strongly depends on salt dispersion and pore confinement. Systems with uniform distribution, such as LiCl@Mg_2_(dobpdc) and LiCl@XJCOF-4, exhibit higher capacity and improved reversibility due to synergistic adsorption between open metal sites and LiCl-derived Lewis-acidic centers. In contrast, poorly stabilized systems such as LiCl@MIL-101­(Cr) exhibit reduced reversibility and partially irreversible uptake due to aggregation, strong chemisorption, or metal leaching. Judicious selection of both the host MOF and the incorporated phase is therefore critical to achieving true synergistic performance. The same governing principles apply to other porous platforms, including COFs, POPs, HOFs, and HCPs, for the design of NH_3_ adsorptive composites.

### 3.6 Macromolecule-Metal Complexes (MMCs) and Metal–Organic Squares (MOS)

In addition to traditional MOF materials, organic–inorganic hybrid structures consisting of intrinsic porosity and Lewis acidic sites, such as macromolecule-metal complexes (MMCs) and metal–organic squares (MOS), have also been studied for NH_3_ applications.^354,356,358^ The PVIm-M-PVBA (M = Co, Cu, Ni) MMCs, synthesized from 1-vinylimidazole and *p*-vinylbenzoic acid with different metal salts exhibited NH_3_ uptake capacities ranging from 11.7 to 20.9 mmol/g at 298 K, 1 bar.^353^ These materials showed a 5–20% reduction in total uptake capacity after 10 adsorption cycles upon activation at 353 K for 2 h under vacuum. The MOS was synthesized using 4,5-imidazoledicarboxylic acid, with 1,2-diaminopropane (MOS-1); 1,10-Phenanthroline (MOS-2); 2,2’-Bipyridine (MOS-3); and cobalt salts. The NH_3_ adsorption performance of MOS was tested using a thermogravimetric analyzer, which shows the total NH_3_ adsorption capacities of 11.5, 3.8, and 5.2 mmol/g for MOS-1,MOS-2, and MOS-3 at 298 K and 1 bar, respectively.^359^ All these MOSs show good reversibility over five consecutive cycles upon vacuum treatment at 298 K for 30 min. GCMC simulations and crystallographic analysis suggest that the smaller pore channels of MOS-2 and MOS-3 hindered adsorption, resulting in lower NH_3_ uptake capacity despite having the same Lewis acidic sites.^359^

## 4 Covalent Organic Frameworks (COFs)

Covalent organic frameworks are ordered crystalline porous materials composed of lightweight elements (e.g., H, B, C, N, O) connected through covalent bonds.^127,389–392^ COFs have garnered tremendous interest in research due to their unique porosity, predesignable architectures, chemical diversity, and high thermal and chemical stabilities.^390^ These properties render them ideal supports for various applications.^389,390,393,394^

In 2010, Yaghi and co-workers investigated the NH_3_ sorption behavior of COFs containing mesoporous Lewis acidic boronic acid groups.^127^ The parent COF-10 consists of Lewis acid boron building units (hexahydroxytriphenylene (HHTP) and biphenyldiboronic acid (BPDA) interconnected into porous hexagonal layers featuring 1D 3.4 nm pores (Figure 24a).^127^ The ammonia adsorption isotherm of COF-10 shows a total uptake of 15.0 mmol/g at 298 K, which surpassed the adsorption capacity of commercial zeolite 13X (9 mmol/g), Amberlyst 15 (11 mmol/g), and MCM-41 (7.9 mmol/g) under the same conditions (Figure 24b).^163^ A pronounced hysteresis observed in COF-10 is likely due to strong interactions between the Lewis acidic boronic acid functional groups and NH_3_. At 298 K, 80% of the adsorbed NH_3_ can be desorbed by a pressure swing to vacuum (0.1 Torr), and the remaining fraction is removed by heating to 473 K under vacuum (<0.1 Torr). However, the powder XRD patterns of COF-10 after cycling experiments show broadened peaks, accompanied by a significant reduction in N_2_ adsorption capacity.

**Figure 24:**
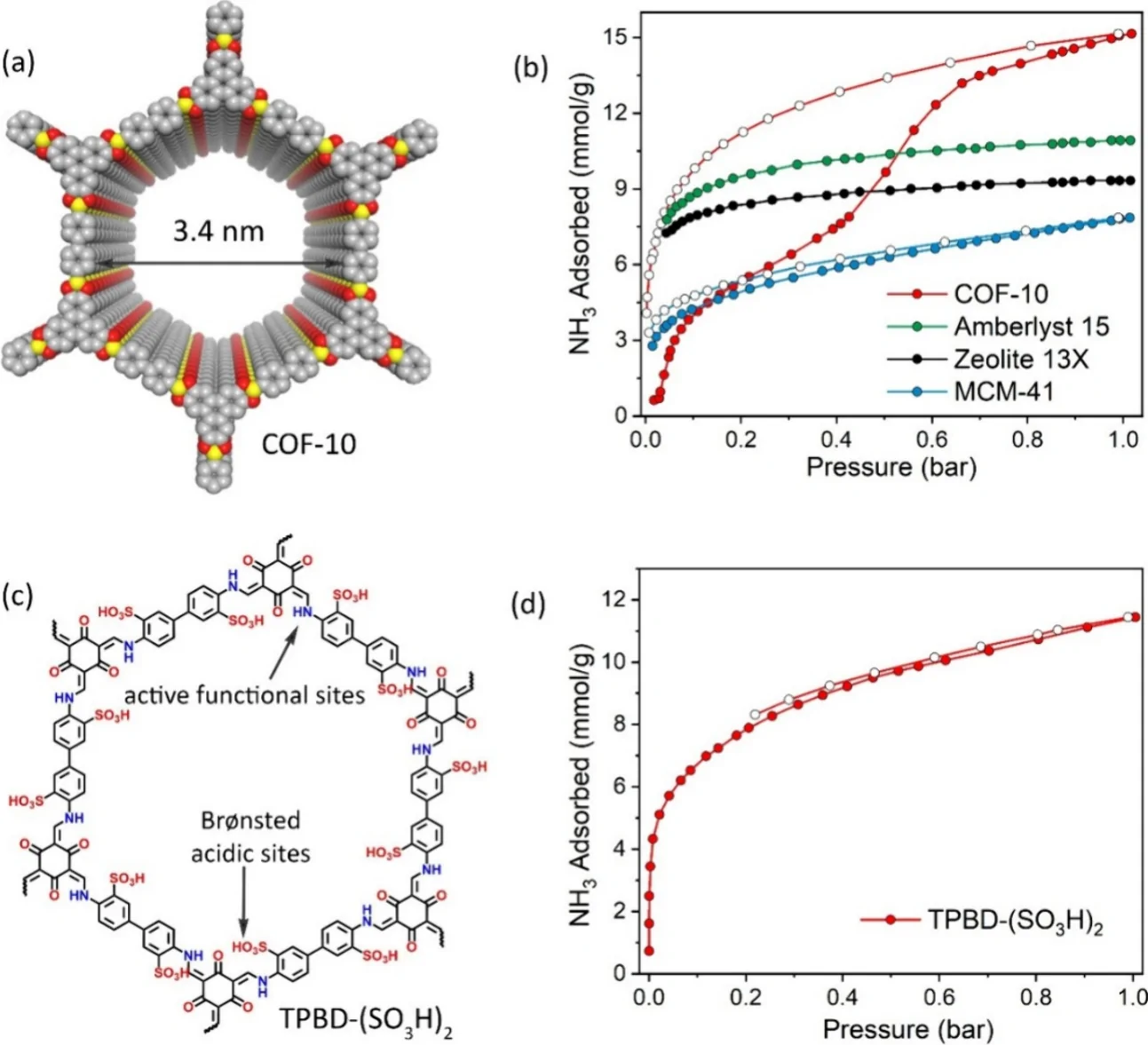
(a) Structure of COF-10 showing the 3.4 nm hexagonal pores. (b) NH_3_ adsorption isotherms of COF-10, Amberlyst 15, zeolite 13X, and MCM-41 at 298 K. Data source: ref 127. (c) Schematic illustration of TPBD-(SO_3_H)_2_ shows the Bro̷nsted acidic −SO_3_H and −OH functional groups. (b) NH_3_ adsorption–desorption isotherm of TpBD-(SO_3_H)_2_ at 298 K. Data source: ref 336.

### 4.1 COFs Containing Bro̷nsted Acidic Sites and Polar Functional Groups

In 2022, Li *et al*. reported a sulfuric acid (−SO_3_H) functionalized COF synthesized using 1,3,5-triformylphloroglucinol (Tp) and 4,4′-diaminobiphenyl-3,3′-disulfonic acid (BD-(SO_3_H)_2_) and it was tested for NH_3_ adsorption applications (Figure 24c).^336^ The NH_3_ adsorption capacity of the TpBD-(SO_3_H)_2_ was found to be 11.5 mmol/g at 298 K and 1 bar (Figure 24d). NH_3_ cycling experiments reveal that after the first cycle, ∼ 16% of the adsorption capacity was lost, and remained constant (∼9.6 mmol/g) for the following five cycles (regeneration conditions were not given).^336^ In situ FT-IR spectra shows the O–H stretching vibration of sulfonic acid groups at 3617 cm^–1^, alongside an intensified N–H stretching of NH_4_^+^ at 3332 cm^–1^ after NH_3_ exposure, suggesting the formation of NH_4_^+^ in the pore channels. The adsorption mechanism of Bro̷nsted acidic COFs follows a similar mechanism to that in Section 3.4 (MOFs containing Bro̷nsted acidic sites).

### 4.2 COF-Based Composite Materials

As detailed in section 3.5, integrating metal halides (e.g., LiCl, MgCl_2_, SrCl_2_, etc.) into porous materials harnesses the advantage of both materials, resulting in a composite with enhanced NH_3_ adsorption and separation properties. This strategy has also gained significant attention in COF-based materials.^11,42,90,395,396^

In 2018, Yang *et al*. reported a series of free-acid-functionalized (COF–COOH) and no free acid COFs which were synthesized using a three-component system of triformylphloroglucinol (Tp), 2,5-diaminobenzoic acid (DAA), and p-phenylenediamine (PA-1) by varying the molar ratios of monomers to obtain [HOOC]_X_-COFs (X = 0, 17, 33, 50, and 100, molar percentage of DAA ligand) (Figure 25a–c).^335^ These COFs were further treated with metal chlorides (SrCl_2_, CaCl_2_, MnCl_2_) to obtain COF–metal halide composites (MCl_2_@[HOOC]_17_-COF or M = Ca^2+^, Mn^2+^, and Sr^2+^) (Figure 25c). Ammonia sorption studies reveal that the pristine [HOOC]_0_-COF (TpPa-1) has 6.85 mmol/g of NH_3_ adsorption capacity at 298 K and 1 bar, while 17% acid-rich COF ([HOOC]_17_-COF) demonstrated 9.34 mmol/g of adsorption capacity under the same conditions, which is ∼ 27% higher than the pristine TaPa-1 (Figure 25d). Additionally, integration of metal chlorides into [HOOC]_17_-COF further enhanced the adsorption capacity, ∼ 24–35% higher than acid-rich [HOOC]_17_-COF and 45–52% higher than pristine TaPa-1. The saturation capacities of 12.25, 11.38, and 14.30 mmol/g at 298 K were achieved for the CaCl_2_, MnCl_2_, and SrCl_2_ loaded [HOOC]_17_-COF (MCl_2_@[HOOC]_17_-COF), respectively (Figure 25d). The highest adsorbing SrCl_2_[HOOC]_17_-COF lost ∼ 23% (10.92 mmol/g) of its initial capacity after three adsorption cycles under ambient conditions. However, activation of COFs after NH_3_ adsorption at 473 K under vacuum for 12 h removed the remaining 23% NH_3_ and enabled the complete retention of its adsorption capacity (14.12 mmol/g) for the next adsorption cycle. These results indicate that MCl_2_@[HOOC]_17_-COF requires higher temperature activation (474 K under vacuum for 12h) between each cycle. Furthermore, XPS and FT-IR analysis of NH_3_-saturated samples indicate that free acids (−COOH) and metal halides in the composites are strongly interacting with NH_3_ through multiple binding interactions (−NH–, −COOH, and −MCl_2_), enabling enhanced NH_3_ adsorption capacity compared to pristine COF.

**Figure 25:**
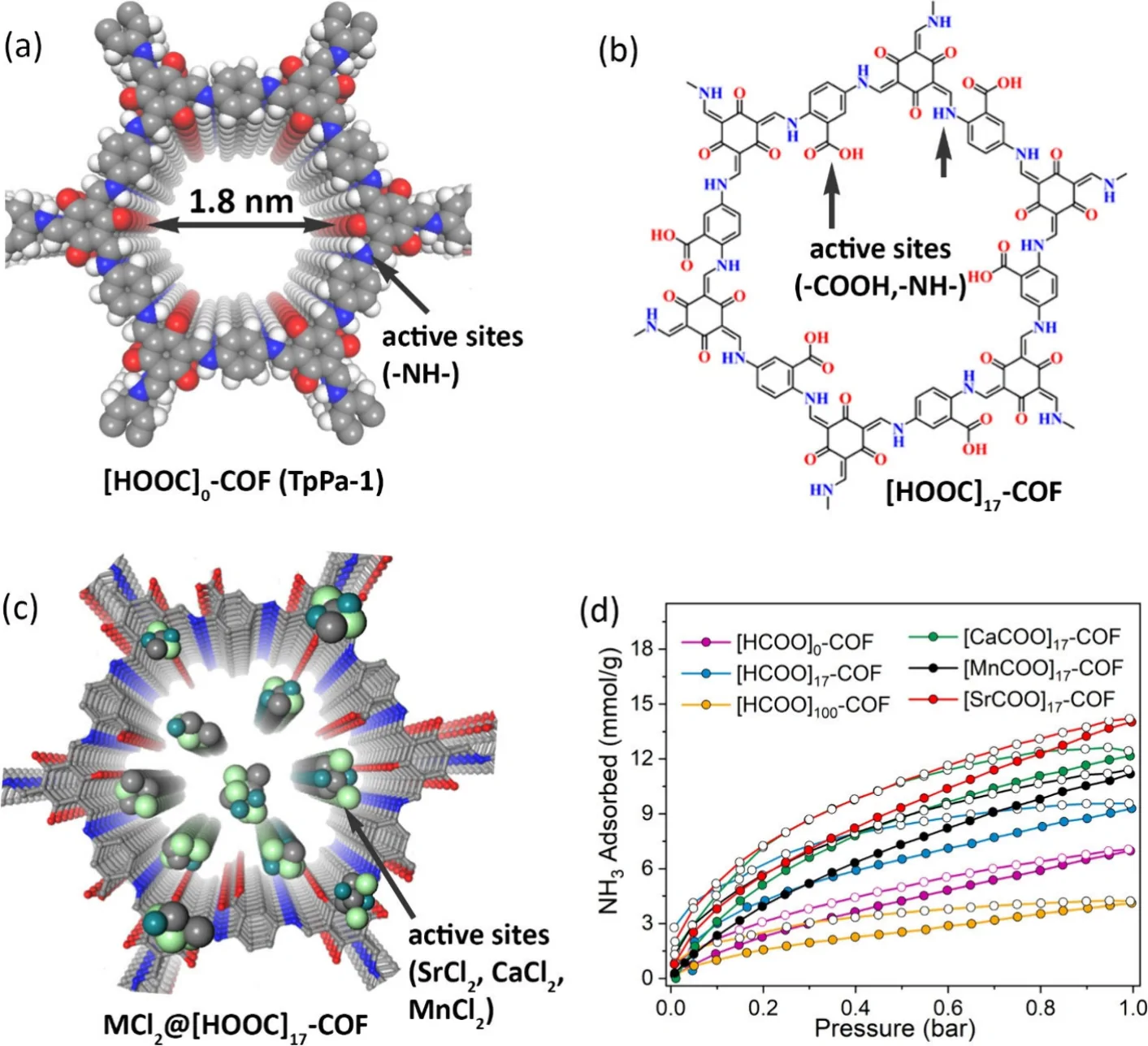
(a) Simulated structure of TaPa-1shows 1.8 nm hexagonal pores. (b) Schematic illustration of [HOOC]_17_-COF structure and the −COOH and −NH– functional groups. (c) Schematic illustration and graphical view of metal chlorides loaded [HOOC]_17_-COF (MCl_2_@[HOOC]_17_-COF). (d) NH_3_ adsorption–desorption isotherms of [HOOC]_0_-COF, [HOOC]_100_-COF, [HOOC]_17_-COF, and MCl_2_@[HOOC]_17_-COF at 298 K. Data source: ref 335.

In 2022, Tian *et al*. integrated calcium chloride salts (CaCl_2_) into IISERP-COF5/TAPT-DMTA-COF and tested its NH_3_ sorption behavior at 298 K.^47^ The parent COF composite (CaCl_2_@COF-x, x = 6%, 11%, 26%, and 34% weight % of CaCl_2_) was synthesized by reacting 2,4,6-tris­(4-aminophenyl)-*s*-triazine (TAPT) and 2,5-dimethoxyterephthalaldehyde (DMTA) followed by loading with CaCl_2_ using impregnation methods (Figure 26a).^47,397^ The COF PXRD patterns upon loading of CaCl_2_ indicated a loss of crystallinity. Washing of the CaCl_2_@COF-x composite with methanol removes the metal salts from the CaCl_2_@COF-x composite and restores the crystallinity and porosity. At 298 K, the NH_3_ adsorption capacities of CaCl_2_@COF-x (x = 6%, 11%, 26%, and 34%) were found to be 6.2, 8.7, 17.2, and 26.5 mmol/g, respectively. The adsorption capacities of these materials were significantly higher than those of the pristine IISERP-COF5 (3.0 mmol/g) and comparable to those of the best-performing adsorbents under the same conditions (Table 4, Figure 26b). However, pronounced hysteresis and the irreversibility of isotherms are observed.

**Figure 26:**
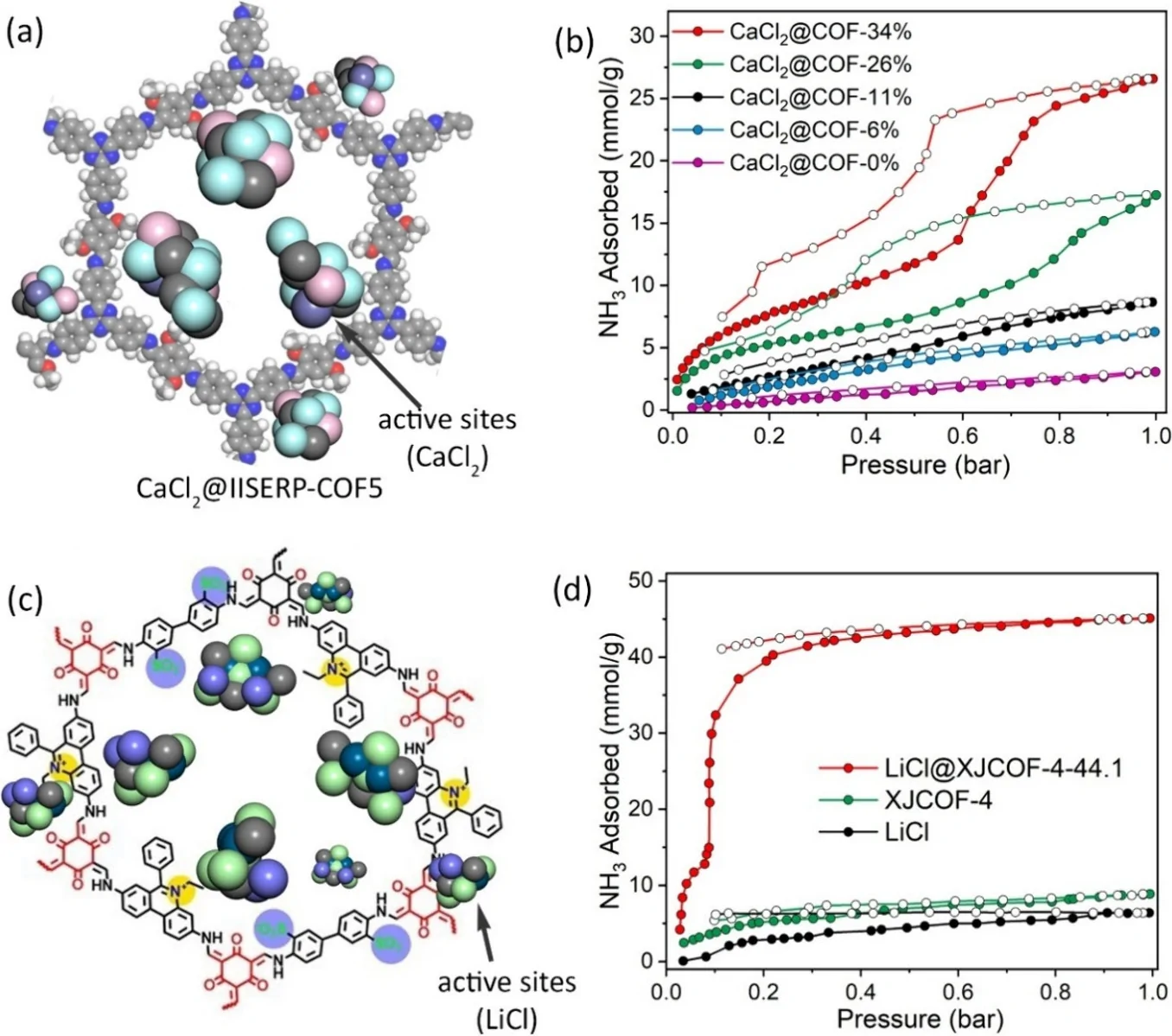
(a) Schematic illustration of CaCl_2_ loaded in COFs (IISERP-COF5/TAPT-DMTA COF). (b) NH_3_ adsorption–desorption isotherms of CaCl_2_@IISERP-COF5-x at 298 K. Data source: ref 47. (c) Schematic illustration of LiCl loaded in XJ-COF4. The anionic and cationic sites in XJ-COF-4 are marked with blue and yellow circles. (d) NH_3_ adsorption–desorption isotherms of LiCl@XJCOF-4–44.1, XJCOF-4, and LiCl at 298 K. Data source: ref 131.

Ammonia adsorption cycling experiments reveal ∼ 8% loss of adsorption capacity after 7 adsorption–desorption cycles (23.3 mmol/g) at 298 K. The nondesorbed ∼ 8% NH_3_ is removed through activation of COFs at above 353 K under vacuum over 2 h. The NH_3_ storage density of CaCl_2_@IISERP-COF5–34% was found to be 0.689 g/cm^3^ at 273 K, which is significantly higher than the best-performing solid sorbents.^47^ XPS and FT-IR analysis reveal that the NH_3_ adsorption mechanism in COF-composite materials follows the same behavior observed in MOF-253­(Al)-NiCl_2_-2 and LiCl@MIL-53-(OH)_2_-43.4.

Subsequently, lithium chloride loaded zwitterionic COF LiCl@XJ-COF-4-x (x = mass% of LiCl, i.e., 29.4, 39.1, 44.1, and 52.6%) was prepared using similar impregnation methods (Figure 26c).^131^ The best-performing composite with 44.1% of LiCl loaded into the COF (LiCl@XJCOF-4–44.1) achieves the highest NH_3_ adsorption capacity of 44.98 mmol/g at 298 K and 1 bar, which corresponds to the adsorption of four NH_3_ molecules per each lithium site. These adsorption capacities surpassed the neat LiCl (6.4 mmol/g) and pristine XJ-COF-4 (8.7 mmol/g) under the same conditions (Figure 26d). At higher temperatures, LiCl@XJ-COF-4–44.1 has 16.6 and 15.5 mmol/g uptake capacity at 333 and 348 K, respectively. Cycling studies indicate that LiCl@XJ-COF-4–44.1 maintained ∼ 98.1% of its initial uptake capacity over six consecutive cycles after activating it at 353 K under vacuum (activation time was not reported)^131^ between each subsequent cycle. Unlike CaCl_2_@IISERP-COF5–34%, the adsorption behavior of the composite as a function of LiCl loading is nonmonotonic, and increasing the LiCl loading beyond 52.6% leads to a decreased uptake capacity.^47^ Mixed gas binary (1%:99%, v/v, NH_3_/N_2_) and ternary NH_3_/CO_2_/N_2_ (1%/1%/98%, v/v/v) breakthrough experiments demonstrate that LiCl@XJCOF-44.1 achieves breakthrough separation selectivity for NH_3_ over N_2_ and CO_2_.^131^

The interactions between LiCl and the zwitterionic framework were further investigated using XPS and ^7^Li–NMR spectroscopy. From the XPS, the N 1s signal decreased from 401.6 to 401.1 eV, indicating a decrease in pyridinium N proportion after LiCl loading. Meanwhile, the sulfur 2p binding energy increased from 167.7 to 168.5 eV after LiCl loading, suggesting that both N^+^ and SO_3_^–^ act as adsorption sites for stabilizing LiCl salts. Furthermore, ^7^Li NMR analysis showed a chemical shift from 3 to 4.5 ppm in LiCl@XJ-COF-4–44.1 compared to neat LiCl. These results highlight that COFs serve as versatile hosts for metal salts, with addition of the functional groups enhancing the metal salt dispersion and overall performance of the composite materials. However, compared with benchmark adsorbents such as zeolites and MOFs, COFs remain expensive due to costly precursors/linkers, multistep organic syntheses, and solvent-intensive preparation methods, all of which currently limit large-scale manufacturing and industrial implementation.^398,399^ Therefore, advancing this research while addressing these economic and synthetic limitations remains critically important.

## 5 Hydrogen-Bonded Organic Frameworks (HOFs)

Hydrogen-bonded organic frameworks are porous materials formed through the self-assembly of organic molecules with complementary hydrogen-bonding functionalities of the same or different molecules.^400^ This self-assembly process generates extended network structures with well-defined pore size and shape.

In 2019, Kang *et al*. synthesized a HOF, Korea University Framework-1 (KUF-1), and KUF-1a (solvent-free) by reacting tetrakis­(4-sulfophenyl)­methane (H_4_SPM) and guanidine hydrochloride (Gua-HCl) using vapor diffusion methods (Figure 27a).^129^ The oxygen atoms of sulfonate groups in SPM^4–^ form multiple hydrogen bonds with the NH_2_ groups of adjacent GuaH^+^ moieties, resulting in the construction of a KUF-1. The ammonia adsorption isotherm in KUF-1a exhibits a step with an adsorption/desorption hysteresis loop and a maximum capacity of 6.67 mmol/g at 1 bar and 298 K (Figure 27d). Notably, KUF-1a maintained ∼ 95% of its capacity over five cycles under vacuum treatment (Table 4). The nondesorbed ∼ 5% of NH_3_ in KUF-1a was removed upon activation of HOFs at 393 K under vacuum (Table 4). Interestingly, KUF-1a has negligible uptake capacity for nitrogen (77 K), oxygen (195 K), and hydrogen (77 K). FT-IR analysis of KUF-1a after NH_3_ adsorption reveals an enhancement in peak intensity at 3300–3500 cm^–1^. In the 1H and 15N NMR spectra, slight peak shifts are observed from the positions of the pristine sample, suggesting that NH_3_ adsorption affects the chemical environments of H and N atoms of KUF-1a due to the formation of additional hydrogen bonding interactions between NH_3_–GuaH^+^, and SPM^4–^ moieties.

**Figure 27:**
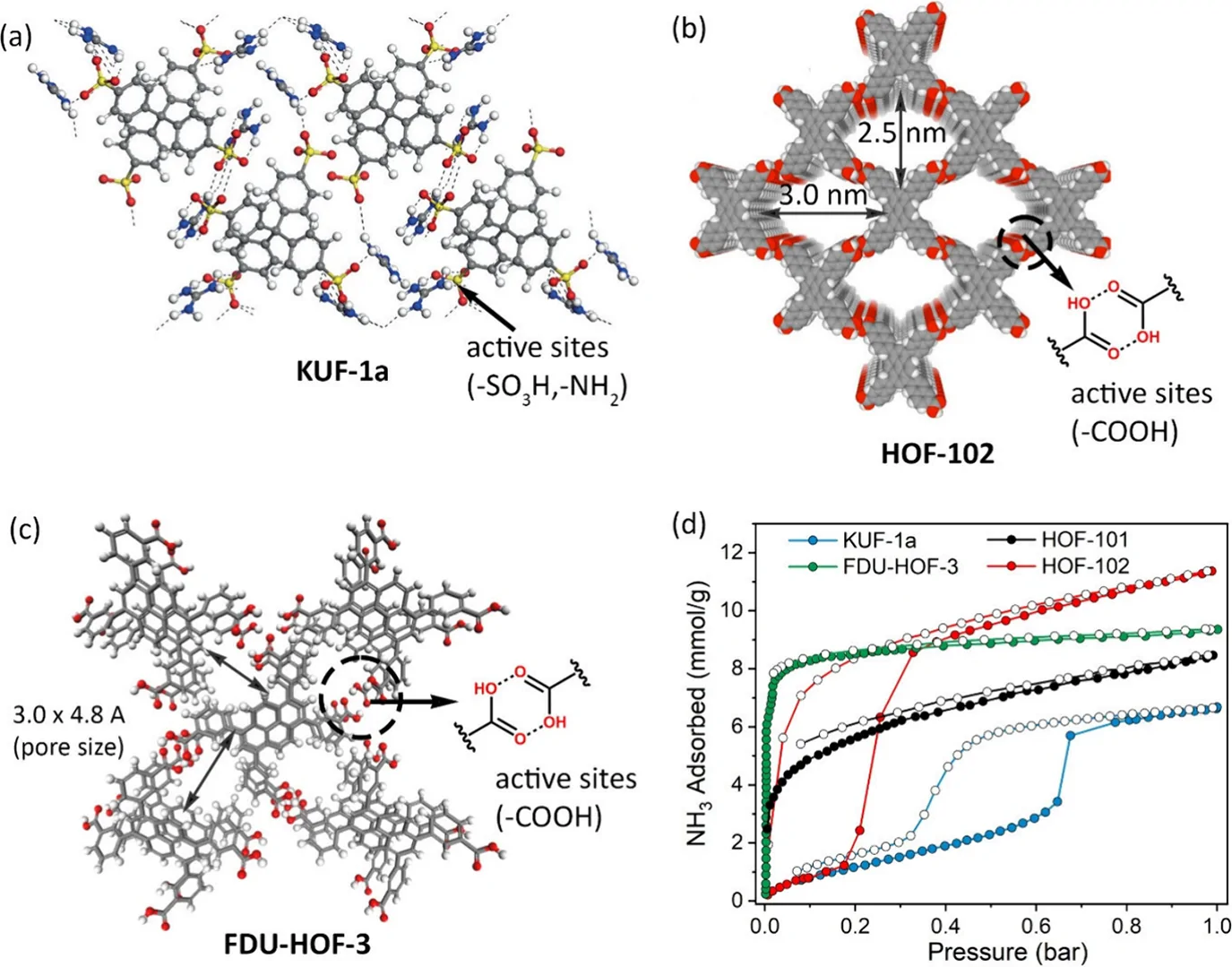
(a) Structure KUF-1a shows the hydrogen-bonding self-assembly and π–π stacking between tetrakis­(4-sulfophenyl)­methane (H_4_SPM) and guanidine hydrochloride (Gua–HCl). Adopted with permission from ref 129. Copyright 2019 Wiley-VCH. (b) Structure HOF-102 shows the hydrogen-bonding self-assembly of acid functional groups of 1,3,6,8-tetra­(6-carboxynaphthalen-2-yl) pyrene. Adopted with permission from ref 401. Copyright 2022 Elsevier. (c) Structure FDU-HOF-3 shows the H-bonding self-assembly of acid functional groups of 3,3,3‴,3‴-(pyrene-1,3,6,8-tetrayl)­tetrabenzoic acid (H_4_PTTB). (d) NH_3_ adsorption–desorption isotherms of KUF-1a, HOF-101, FOF-102, and FDU-HOF-3 at 298 K. Data source: refs 129, 338, 339 .

Subsequently, in 2020, Farha and co-workers reported a pyrene base HOF (HOF-102), synthesized using 1,3,6,8-tetra­(6-carboxynaphthalen-2-yl) pyrene (H_4_TNAPy) (Figure 27b).^338^ The self-assembly of carboxylic acid groups in H_4_TNAPy with complementary carboxylic acids of the same molecules generated a head-to-head (−COOH―HOOC−) hydrogen bonding framework consisting of 2.5 × 3.0 nm pores. At 298 K, the ammonia adsorption capacity of HOF-102 was found to be 11.16 mmol/g with a step isotherm and pronounced hysteresis (Figure 27d). The desorption isotherm reveals that the adsorbed NH_3_ in HOF-102 is fully reversible upon reduction of pressure (<0.1 bar) under ambient conditions. The powder-XRD patterns of HOF-102 after NH_3_ adsorption retained their crystallinity and peak positions. Additionally, HOF-102 exhibits exceptional chemical robustness in harsh aqueous environments, including acidic media, buffer solutions, boiling water, and extreme pH conditions (pH 1–14), making it one of the most chemically robust frameworks within the HOF family, with stability comparable to that of some MOFs.^338^

In 2024, Song *et al*. reported another microporous HOF, FDU-HOF-3, consisting of the self-assembly of 3,3′,3‴,3‴′-(pyrene-1,3,6,8-tetrayl)­tetrabenzoic acid (H_4_PTTB) and compared its NH_3_ adsorption capacity with HOF-101, a 4,4′,4″,4‴-(Pyrene-1,3,6,8-tetrayl)­tetrabenzoic acid HOF (Figure 27c).^339^ The crystal structures of both HOF-101 and FDU-HOF-3 exhibit −COOH–HOOC– head-to-head hydrogen bonding and π–π stacking interactions same like HOF102. The pore sizes of FDU-HOF-3 were found to be 0.3 × 0.48 nm, whereas HOF-101 has 1.8 × 2.4 nm (Figure 27c). The smaller pore size of FDU-HOF-3 is attributed to the position and angles between the carboxylic acid and pyrene in the two isomeric organic molecules (180° in HOF-101 and 120° in FDU-HOF-3).^339^ Unlike HOF-2 and KUF-1a, the NH_3_ adsorption isotherm of FDU-HOF-3 is a type-1 isotherm with adsorption capacity of 9.34 mmol/g at 298 K and 1 bar. This NH_3_ adsorption capacity is superior to HOF-101 (8.44 mmol/g), KUF-1a (6.67 mmol/g), and cyclic oligophenylenes under the same conditions (Figure 27d and Table 4).^129,338,339,402^ The heat of adsorption (*Q*_st_) of NH_3_ on FDU-HOF-3 was found to be 69.3 kJ/mol, indicating the presence of chemical interaction between NH_3_ molecules and the −COOH groups. Both FDU-HOF-3 and HOF-101 have negligible uptake capacity for N_2_, O_2_, and CO_2_, and exhibit good cycling performance over 10 consecutive cycles at 298 K upon activation at 323–363 K under vacuum. Although the uptake capacity and synthesis cost of HOFs are inferior to zeolites, MOFs, and COFs, their excellent adsorption–desorption reversibility and mild regeneration behavior observed in some HOFs justify further studies.

## 6 Porous Organic Polymers (POPs), Hyper-Cross-Linked Polymers (HCPs), and Porous Aromatic Frameworks (PAFs)

### 6.1 POPs Containing Bro̷nsted Acid and Polar Functional Groups

Porous organic polymers (POPs) or porous aromatic frameworks (PAFs) are porous materials formed by the reaction between two or more functional organic moieties connected by strong covalent bonds (carbon–carbon, carbon–nitrogen, and/or carbon–heteroatom bonding, etc.).^403–405^ The extended cross-linking connectivity between growing chains results in a polymeric material consisting of intrinsic porosity and high structural stability. These robust characteristics allow the framework to incorporate various active materials and to undergo surface modifications.^128^

In 2012, Peterson *et al*. reported on an amorphous polymer material (NU-POP-1) derived through an amine and anhydride condensation reaction, which generated 3.5 to 8 Å micropores.^345^ Its NH_3_ adsorption capacity was evaluated using microbreakthrough analysis, revealing a saturation loading capacity of 5.56 mmol/g at 0% RH and 6.17 mmol/g at 80% RH. Subsequently, Long and co-workers investigated a series of porous aromatic frameworks (PAFs) containing densely packed Bro̷nsted acid functional groups.^128,297,406^ The porous aromatic framework, PAF-1, was synthesized by reacting tetrakis­(4-bromophenyl)­methane with biphenyl linkers under polymerization catalytic conditions, yielding a porous organic polymers that consists of a tetrahedrally connected diamondoid (diamond-like 3D network) structures. (Figure 28a and 28b).^128,297^ PAF-1 was further subjected to postsynthetic modification with different functional groups, leading to the formation of various functionalized porous polymers: P1-PO_3_H_2_, P1-NH_2_ (BPF-1), P1-NH_3_Cl (BPF-2), and PPN-6-SO_3_H (Figure 28a and 28b).^128,297^ At 298 K, these PAFs demonstrated NH_3_ adsorption capacities of 18.7, 12.0, 9.0, and 2.2 mmol/g for P1-PO_3_H_2_, P1-NH_2_ (BPF-1), P1-NH_3_Cl (BPF-2), and PAF-1, respectively (Figure 28c). Despite the lower acidity of phosphonic acid, P1-PO_3_H_2_ exhibited the highest NH_3_ capacity under ambient conditions, suggesting that the doubly acidic nature of phosphonic acid (PO_3_H_2_) may increase the total number of acidic sites within the framework, contributing to the enhanced NH_3_ capacity.

**Figure 28:**
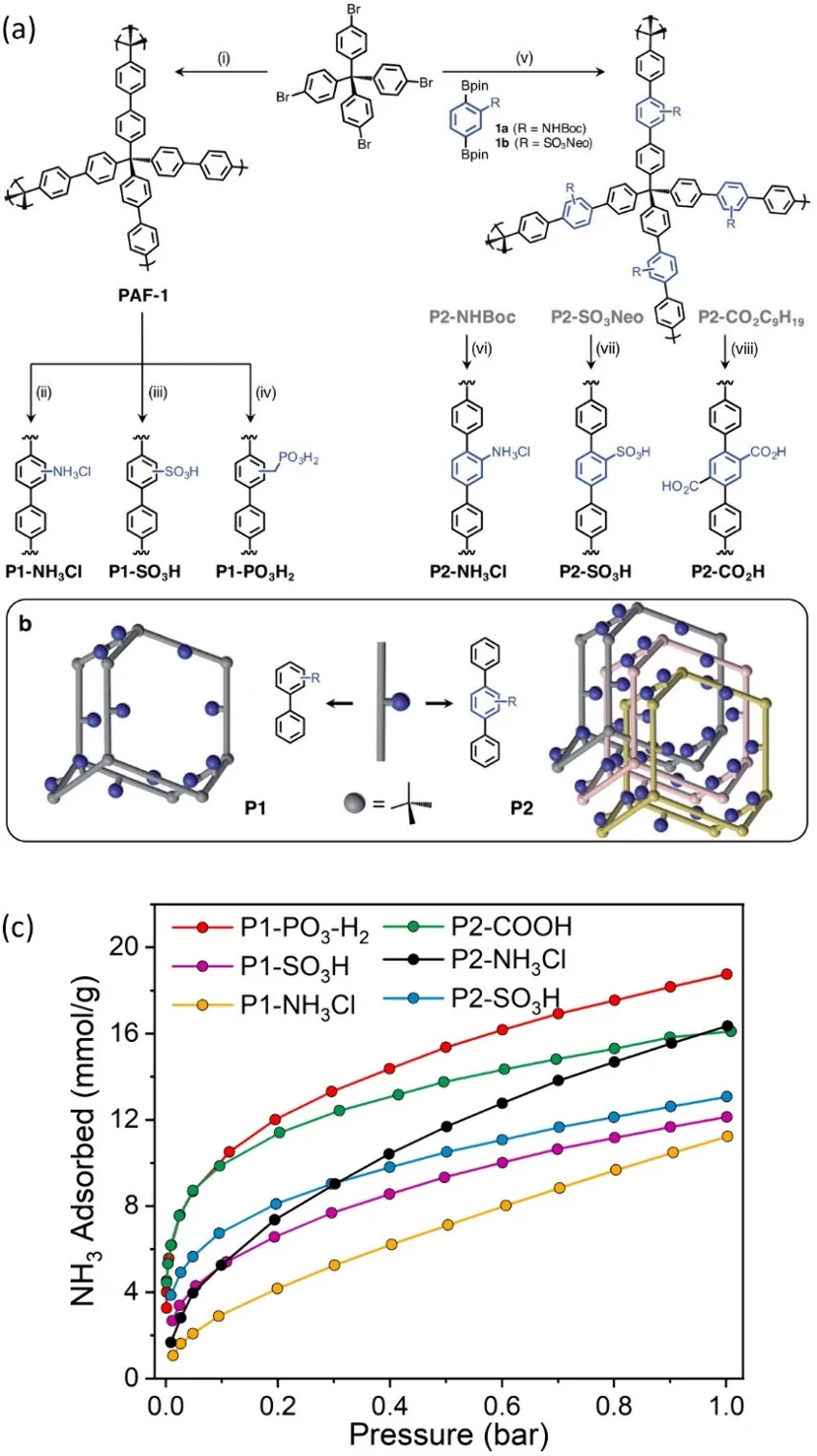
(a) Schematic representation and synthesis procedure of P1- and P2-polymers. (b) Schematic illustration of polymerization network in P-polymers and interpenetrated network in P2-polymers. 28a and 28b adopted with permission from ref 128. Copyright 2017 Royal Society of Chemistry. (c) NH_3_ adsorption isotherms of P1- and P2-polymers at 298 K. Data source: refs 128, 297 .

Furthermore, the polymerization of tetrakis­(4-bromophenyl)­methane with functional terphenyl ligands produced interpenetrated diamondoid structures in P2-NH_4_Cl, P2-SO_3_H, and P2-COOH (Figure 28a and 28b). These interpenetrated PAFs exhibited NH_3_ uptake capacities of 16.3, 16.1, and 13.1 mmol/g for P2-NH_4_Cl, P2-CO_2_H, and P2-SO_3_H, respectively at 298 K and 1 bar. The P1 polymers (P1-SO_3_H and P1-PO_3_H_2_) exhibited a gradual release of NH_3_ during breakthrough desorption measurements upon purging the column with air (desorption was reversible). In contrast, the P2-polymers (P2-SO_3_H and P2-CO_2_H) retained more NH_3_ during desorption (desorption was irreversible). This may be attributed to the interpenetrated structure of the P2-polymers (P2-SO_3_H and P2-CO_2_H), enabling the acid functional groups to pack closely, thereby generating a confined acid functional pore environment that strongly binds NH_3_ molecules. In situ FT-IR analysis was performed on Bro̷nsted acidic polymers (P1-SO_3_H, P1-PO_3_H_2_, and P2-CO_2_H) under ambient temperature with varying NH_3_ partial pressures. This analysis revealed peaks at ∼ 1450 cm^–1^, suggesting the formation of the ammonium cation (NH_4_^+^). In contrast, PVA-1 exhibited no noticeable signal pattern change due to the absence of active acidic functional groups. The superior capacities of P1-polymers suggest that the spatial distribution and proximity of neighboring acidic groups within the pores are important characteristics.

Later, poly­(amic acid) (PAA) and polycyclic imides (PI) were synthesized through a water-assisted method employing tetrakis­(4-aminophenyl)­methane and pyromellitic anhydride as reactants.^343^ This synthetic approach yielded a porous PAA polymer under hydrated conditions and PI under anhydrous conditions (Figure 29a). PAA consists of amic acid and demonstrates a greater density of Bro̷nsted acidic sites (−CO_2_H), hydrogen-bond donors (−CONH−), and acceptors (−C=O), compared to the polycyclic imides PI (−CONCO−). Ammonia adsorption analysis indicates that PAA exhibits a steeper uptake behavior at low pressures and reaches 10.7 mmol/g at 298 K and 1 bar (Figure 29b). In contrast, PI demonstrates a slightly lower NH_3_ capacity at lower pressures (0.4 mmol/g) and reaches a saturation capacity of 9.0 mmol/g under the same conditions, despite its higher surface area (725 m^2^/g for PI vs 365 m^2^/g for PAA). However, adsorption–desorption cyclic experiments reveal that both PAA and PI lost ∼ 35% and ∼ 13% of their initial NH_3_ adsorption capacity, respectively, even after heating them at 353 K for 4 h. This phenomenon could be attributed to the presence of more Bro̷nsted acidic sites (−COOH) and multiple hydrogen-bond donors (−CONH−) and acceptors (−C=O) within PAA compared to PI.

**Figure 29:**
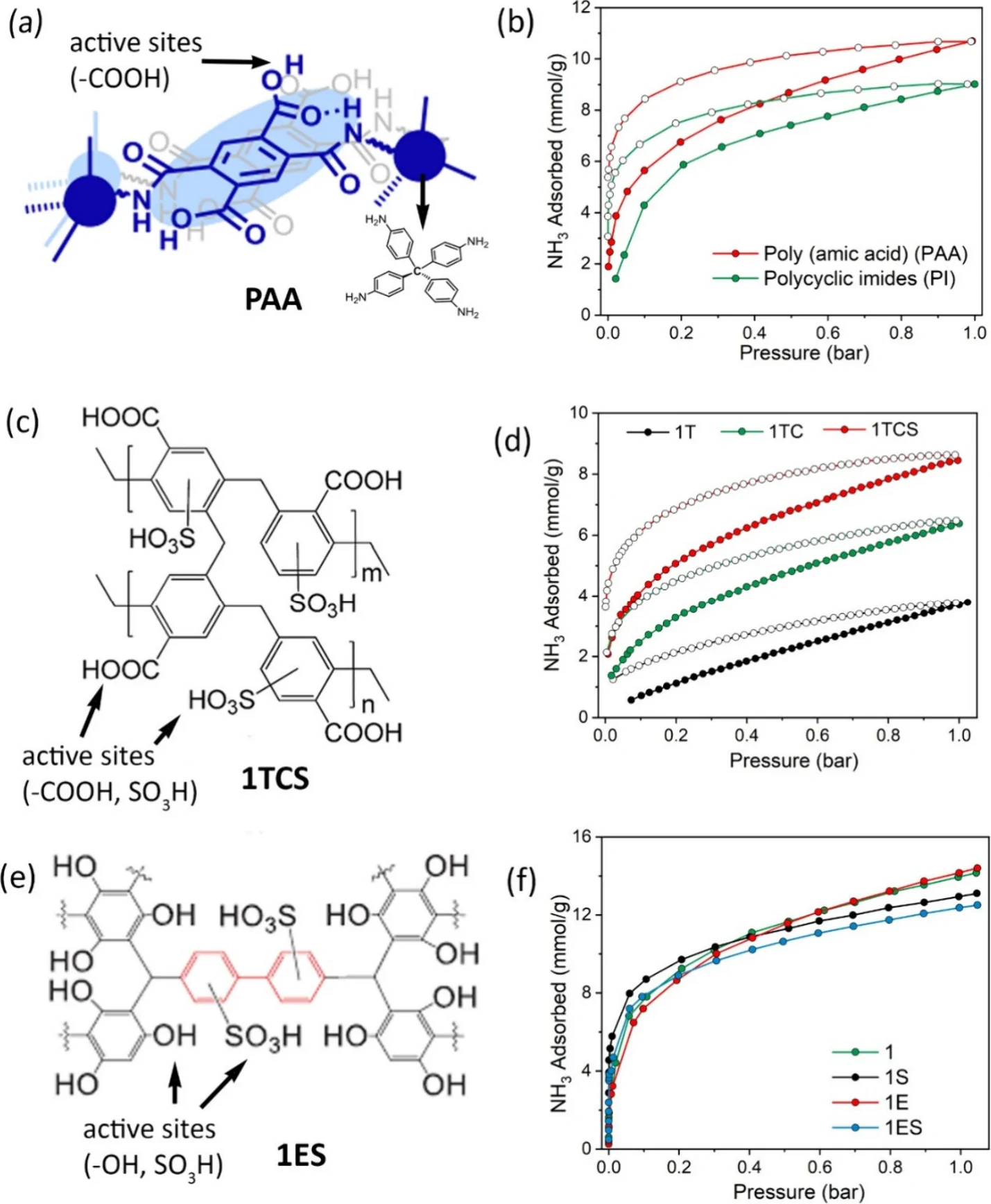
(a) Schematic illustration and functional groups of PAA polymers. Adopted with permission from ref 343. Copyright 2017 American Chemical Society. (b) NH_3_ adsorption–desorption isotherms of PAA and PI at 298 K. Data source: ref 343. (c) Structure illustration and functional groups of 1TCS polymers. Adopted with permission from ref 349. Copyright 2018 Royal Society of Chemistry. (d) NH_3_ adsorption–desorption isotherms of 1T, 1TC, and 1TCS at 298 K. Data source: ref 349. (e) Structure illustration and functional groups of 1ES polymers. Adopted with permission from ref 342. Copyright 2021 Wiley-VCH. (f) NH_3_ adsorption isotherms of 1, 1S, 1E, and1ES at 298 K. Data source: ref 342.

### 6.2 Hyper-Cross-Linked Polymers (HCPs)

In 2018, Hong and co-workers synthesized a series of hyper-cross-linked porous organic polymers comprising alkyl (−CH_3_, 1T), carboxylic acid (−COOH, 1TC), and a combination of sulfuric acid and carboxylic acid (−SO_3_H, + −COOH, 1TCS) functional groups by using formaldehyde dimethyl acetal (FDA) and 1,2-dichloroethane (DCE) under Friedel- Craft alkylation conditions followed by oxidation and sulphonation postsynthetic modifications (Figure 29c).^349^ BET surface area studies revealed that the successive modification of the 1T polymer (915 m^2^/g) led to a gradual decrease in the surface area of 1TC (552 m^2^/g) and 1TCS (72.5 m^2^/g). At 298 K, the NH_3_ adsorption capacity of functionalized POPs was found to be 3.8 mmol/g for 1T, 6.41 mmol/g for 1TC, and 8.52 mmol/g for 1TCS (Figure 29d). Despite a significant reduction in surface area, 1TCS has an enhanced uptake capacity due to the presence of highly active Bro̷nsted acidic sites (−COOH and – SO_3_H). Furthermore, cycling experiments demonstrated that water-saturated 1TCS retained its NH_3_ capacity over 10 consecutive cycles upon activation at 433 K under N_2_ purging. The enthalpy of NH_3_ adsorption for the polymers estimated using the Clausius–Clapeyron equation increased when the acidic sites in the polymers increased (1TCS (120–60 kJ/mol) > 1TC (43–40 kJ/mol)) > 1T (∼30 kJ/mol). The sulfonated polymer (1TCS) was further coated with 10 mg of hydroxyl-terminated poly­(dimethylsiloxane) (PDMS), resulting in the hydrophobic TCS@PDMS10 polymer with a contact angle of 121–134°. Notably, the NH_3_ adsorption capacity of 1TCS@PDMS10 increased to 1.41 mmol/g at 500 ppm, which is 40-fold higher than pristine polymer IT. However, the total uptake capacity of hydrophobic TCS@PDMS10 remains the same as 1TCS (∼8.5 mmol/g) at 298 K.

The same group later reported the phloroglucinol-based polymers (1 and 1E) synthesized through microwave-assisted condensation reactions between phloroglucinol and terephthalaldehyde (1) or 4,4′-biphenyldicarboxaldehyde (1E) (Figure 29e).^342^ These polymers (1 and 1E) were then treated with chlorosulfonic acid (ClSO_3_H) to afford sulfonated polymers (1S and 1ES), which were further alkylated to yield hydrophobic polymers (1SC_*x*_ and 1ESC_*x*_). The ammonia sorption isotherm demonstrates that the pristine polymers 1 and 1E possess adsorption capacities of 1.63 mmol/g at 0.67 mbar, and 1.08 mmol/g at 0.61 mbar, respectively (Figure 29f). Upon sulfonation, the low-pressure NH_3_ adsorption capacity increases to 4.03 mmol/g at 0.5 mbar for 1S and 3.51 mmol/g at 0.58 mbar for 1ES. However, the overall uptake capacity remains constant before and after sulfonation at 298 K and 1 bar, with uptake capacity of ∼ 14.0 mmol/g for both 1 and 1S, and ∼ 12.9 mmol/g for both 1E and 1ES (Figure 29f). Despite the incorporation of various functional groups, POP- and PAF-based materials still have shown low NH_3_ adsorption capacities compared with other classes of adsorbents (Table 4). Their performance beyond conventional functionalities (e.g., −SO_3_H, −COOH, −OH) remains largely unexplored. Given their high structural stability, POPs represent promising platforms for further modification through the incorporation of metal salts, ionic liquids, or other active components.

As an example of the incorporation of metal salts into HCPs, Zong*et al*. synthesized a series of HCPs via a one-step cross-linking reaction and used them as supports for various Li^+^ salts to separate NH_3_.^351^ The synergistic effect of the composite was evidenced by NH_3_ adsorption reaching up to 34.76 mmol/g for LiCl, while LiCl and HCP separately exhibited about 1.32 mmol/g and 7.2 mmol/g at 1 bar and 298 K, respectively. Via XPS, FT-IR, and DFT calculations, they demonstrated the interaction mechanism between the lone electron pair of NH_3_ with the Li^+^ cations well-dispersed across the HCP structure, as well as hydrogen bonding with the Cl^–^ ions. The synergy between LiCl and HCP was studied by DFT calculations, indicating that the most negatively charged aromatic ring of HCP can retain the Li atoms, providing stability. High-temperature NH_3_ adsorption evaluation of the best material (LiCl-HCP-5.35) showed a promising uptake capacity of about 12.87 mmol/g at 353 K and 1 bar, which is comparable to traditional adsorbents at room temperature, such as 13X zeolite (Table 2). An important factor for the practical application of adsorbent/absorbent materials is the regeneration and long-term durability. In this study, multiple adsorption–desorption cycles demonstrated a stable working capacity over 10 consecutive cycles at 353 K under a N_2_ flow. Therefore, further studies across a broader range of temperatures and pressures, along with additional modifications, should be conducted to leverage the stability of the HCPs.

## 7 Summary and Research Opportunities

### 7.1 High-Capacity Reversible Adsorbents

Over the past six decades, ammonia adsorption on porous solids has been extensively studied, primarily driven by ammonia’s indispensable role in fertilizers, chemicals, and emerging hydrogen technologies.^27,407^ Extra-framework cations in aluminosilicate zeolites, open metal sites-, and functionalized organic ligands with Bro̷nsted acidic or polar groups, and groups forming hydrogen bonds with NH_3_ in MOFs, COFs, POPs, and HOFs have been studied (Table 2 and Table 4).^248,408–410^ Although each strategy and material class confers distinct advantages, their effectiveness and limitations are predominantly controlled by the structural and chemical characteristics of the framework, which vary significantly across different classes of materials.

Within open metal site (OMS)-containing MOFs, Mg_2_(dobpdc) (23.9 mmol/g), Cu_2_Cl_2_(BBTA) (19.8 mmol/g), and Cu­(cyhdc) (18.7 mmol/g) exhibit high NH_3_ uptake under ambient conditions due to favorable interactions at the open metal centers (Section 3.2 and Table 4). However, strong interactions at Lewis acidic OMS often lead to pronounced hysteresis, incomplete desorption, framework degradation, and also require high-temperature activation (>423 K), imposing significant energy penalties and complexity in NH_3_ adsorption and separation applications (Figures 16, 17, and 30). Notably, some of these MOFs have demonstrated among the highest heats of adsorption reported, underscoring the presence of strong chemical interactions between OMSs and NH_3_ molecules (Figure 30b). In contrast, Bro̷nsted acid-functionalized adsorbents (−COOH, −SO_3_H, μ-OH), such as Ni_acryl_TMA (23.5 mmol/g), MOF-300­(Al), (19.7 mmol/g), MFM-300­(Al) (13.9 mmol/g), TpBD-(SO_3_H)_2_ (11.5 mmol/g), and 1TCS (8.52 mmol/g), display higher desorption reversibility, and lower heats of adsorption, due to weaker acid–base or hydrogen-bonding interactions with NH_3_ (Figures 18–21, 24, 29, and 30). These materials exhibit typical steep type-I isotherms at low pressures, and once the adsorption sites are saturated with NH_3_, further uptake is constrained. This limitation is particularly pronounced in HOF, COFs, and POPs, which, despite their high surface areas and ordered functional groups, consistently exhibit lower NH_3_ uptake capacity (Sections 4, 5, and 6).

**Figure 30:**
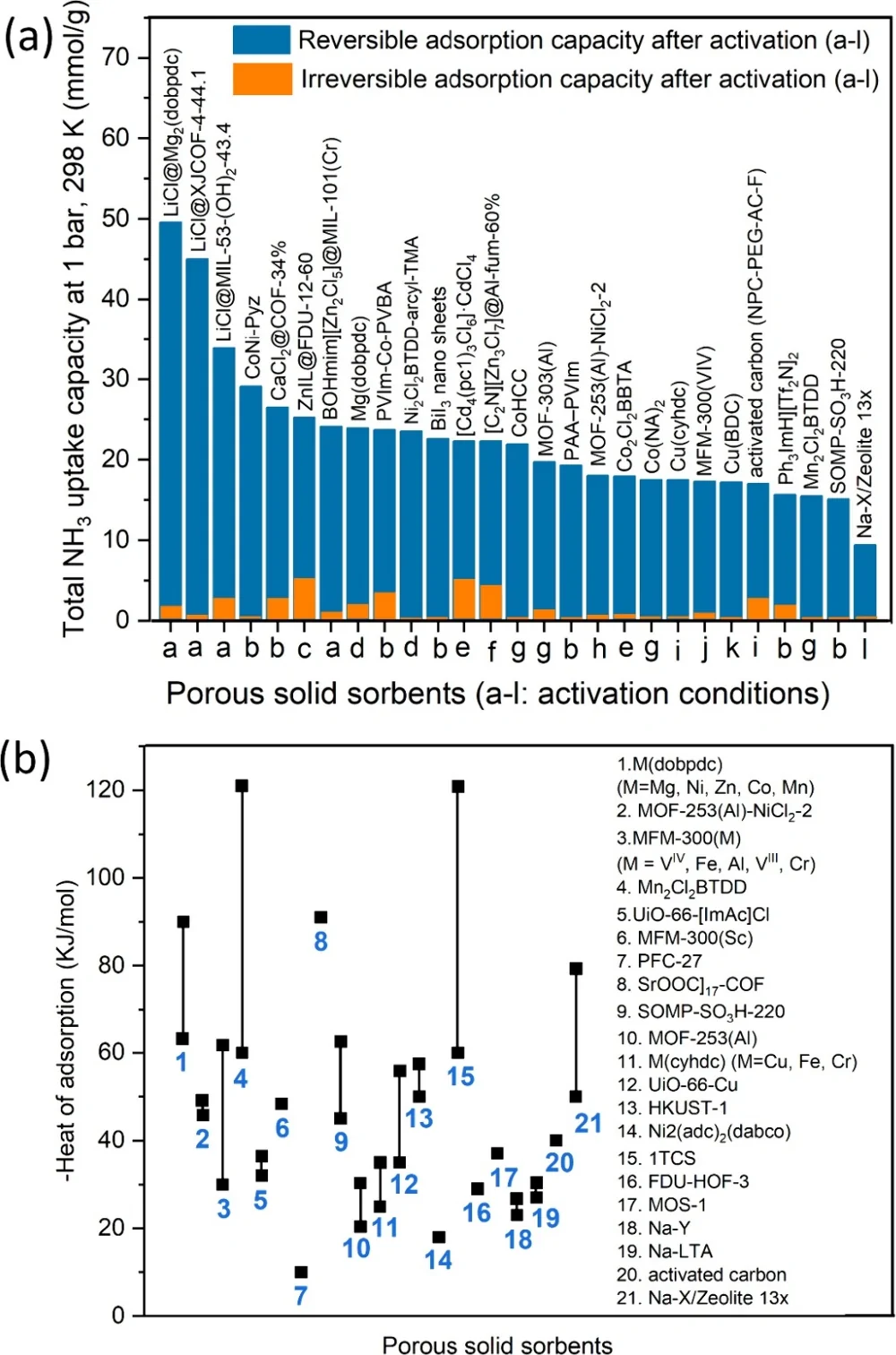
(a) Reversible and irreversible NH_3_ uptake capacities of the solid sorbents, those with NH_3_ adsorption capacities were above 15 mmol/g at 298 K and 1 bar (Note: zeolite 13X/Na-X is additionally shown despite being below the cutoff). The reversible and irreversible capacities are calculated based on the total uptake capacity of the material after NH_3_ adsorption–desorption cycling experiments (irreversible capacity = first cycle adsorption capacity – last cycle adsorption capacity). More details are provided in Table 4, including the number of cycles performed for each material, experimental conditions, and their stability, etc. All the materials were activated under vacuum at different temperatures and time: a = 373 K, 2–12 h; b = 353 K, 2–6 h; c = 323 K, 6 h; d = under high vacuum for 2–12 h; e = 473 K, 12 h; f = not reported in the paper; g = 423 K, 24 h; h = 403 K, time not reported; i = 433 K, time not reported; j = 373–473 K, time not reported; k = 473 K, time not reported, l = 573 K, under vacuum, time not reported. (b) Heats of adsorption (*Q*_st_) for the respective solid sorbents are summarized based on the data reported in the literature. Data sources are included in Table 4.

Synergistic strategies combining OMS with Bro̷nsted acidic or polar groups can be used to fine-tune adsorption behavior, particularly by integrating open-metal metal sites with Bro̷nsted acidic bridging hydroxyl (μ-OH) groups (e.g., MFM-300­(V)), metal salts- impregnated hybrid composites (e.g., LiCl@Mg_2_(dobpdc)), postsynthetic introduction of high-density Bro̷nsted acid groups (e.g., dicarboxylic acid (−(COOH)_2_) appended Ni_acryl_TMA), grafting of multidentate functional groups (e.g., MOF-300­(Al)), and incorporation of mixed-metal centers (e.g., CoNi-Pyz). These approaches can enhance adsorption–desorption reversibility, fine-tune isotherm shapes, and increase overall uptake capacity compared with pristine materials (Table 4). Among all these approaches, ionic liquids and metal salt–impregnated composites (e.g., LiCl, CaCl_2_, NiCl_2_) exhibit the most promising NH_3_ adsorption capacities while retaining stability and reversibility (Figure 23b, 30a, and Table 4). Notably, LiCl-loaded LiCl@Mg_2_(dobpdc)-5 (37.98 wt % LiCl) and LiCl@XJCOF-4 (44.1 wt % LiCl) composites achieved the highest total adsorption capacities of 48.3 and 44.98 mmol/g, respectively, at 298 K and 1 bar.^132,411^ Unlike solely OMS or functional groups, which coordinate one NH_3_ molecule per site, the presence of metal salts provides multiple coordination sites per ion, allowing simultaneous binding of several NH_3_ molecules depending on pore environment and confinement (e.g., Li^+^ coordinates up to four NH_3_ molecules, Ca^2+^ up to 6 NH_3_, and Ni^2+^ up to 4 NH_3_). This multicoordination capability of the composite materials results in multistep adsorption isotherms with enhanced uptake capacity (Figure 23b).

While the amount of LiCl content in the porous support plays a crucial role in NH_3_ adsorption capacity, higher LiCl loading alone does not always lead to increased uptake. Indeed, despite high LiCl loading, large surface area, and intrinsic stability, LiCl@MIL-101­(Cr) (Li-to-Cr molar ratio: 5.3) exhibited lower NH_3_ capacity and significant metal leaching after the first adsorption cycle (Figure 23b).^132^ In contrast, less Li containing open-metal Mg_2_(dobpdc) (Li-to-Mg molar ratio:4.95, LiCl wt %, 58.59) and zwitterionic XJ-COF-4-x (LiCl wt %, 44.1) achieve higher capacities with reversible desorption over multiple cycles under the same conditions. DFT calculations and TPD studies indicate Li@Mg_2_(dobpdc) composite exhibited dual NH_3_ binding characteristics (OMS: Mg–NH_3_ (−85.8 kJ/mol) and LiCl: Li–NH_3_ (−100.8 kJ/mol)), whereas bare Mg_2_(dobpdc) shows a single binding site (−90.0 kJ/mol at OMS-Mg_2_(dobpdc)).^132^ This reveals that in LiCl@Mg_2_(dobpdc), LiCl introduces stronger adsorption sites for ammonia, while slightly weakening OMS bonding affinity with NH_3_ (Mg–NH_3_ interactions).

From a practical standpoint, the design of adsorbents for ammonia separation is inherently governed by a trade-off between adsorption strength and reversibility. Materials exhibiting exceptionally high NH_3_ capacities—particularly those incorporating open metal sites or metal salt–impregnated composites such as LiCl@Mg_2_(dobpdc)—often rely on strong chemisorptive interactions, which can limit the usable working capacity in cyclic operation and increase regeneration energy requirements. Conversely, materials with weaker interactions, such as Bro̷nsted acid–functionalized frameworks, offer improved reversibility and lower regeneration penalties but at the expense of reduced uptake and early saturation.

### 7.2 Characterization and Measurement Methods

Conventional characterization techniques, such as PXRD, FTIR, and spectroscopic studies, provide valuable insights into NH_3_ adsorption mechanisms and host–guest interactions.^132,271,412^ Still, these studies are largely limited to pre- or post-NH_3_-saturated samples, which do not reflect actual adsorption conditions and fail to capture partial framework decomposition, gradual loss of active sites, or subtle site deactivation. Assessing material stability and performance thus requires advanced in situ analytical techniques, particularly under working conditions and over multiple adsorption–desorption cycles.^249,411,413^ For instance, in situ X-ray^330,414^ or neutron diffraction,^415^ diffuse reflectance UV–vis spectroscopy,^416^ and electron paramagnetic resonance (EPR)^417^ are highly effective for probing real-time structural, electronic, and chemical changes during NH_3_ adsorption. Additionally, temperature-programmed desorption (TPD),^418^ diffuse reflectance infrared Fourier transform spectroscopy (DRIFTS),^419^ X-ray photoelectron spectroscopy (XPS),^420^ calorimetry^421^ and microcalorimetry^422^ provide further insights into adsorption energetics, kinetics, the nature of active sites, and possible charge transfer interactions between NH_3_ molecules and the framework.

In the context of ammonia synthesis and decomposition processes, the NH_3_, H_2_, and N_2_ mixtures to be separated are highly pure (i.e., with low levels of problematic contaminants like H_2_O, O_2_, CO, and sulfur, phosphorus, and metal/metalloid compounds) because feed streams are purified before entering the reactor to avoid catalyst poisoning.^423–425^ In this respect, focusing on highly pure NH_3_, H_2_, and N_2_ mixtures is justified. The challenge is in the pressure and temperature range of these measurements and in how to predict mixture adsorption equilibrium selectivities based on single-component isotherms. The reviewed literature is dominated by single-component isotherms at 298 K up to 1 bar (Table 2, 3, and 4), which, in terms of performance criteria like ammonia adsorption capacity, tend to favor strong adsorbents that saturate at these conditions, while weaker adsorbents like pure silica zeolites and MOFs like MIL-101 may be overlooked. Adsorption of NH_3_ at elevated temperatures and pressures is particularly challenging due to ammonia’s corrosive and reactive nature, which can damage crucial components such as valves, seals, and pressure transducers. Additionally, the phase transition tendency of ammonia can lead to local condensation within the dosing lines under temperature or pressure gradients, underscoring the need for specialized, corrosion-resistant, and condensate-proof equipment for reliable measurements.^45,48,49,176,426^ Recently, Aristizabal-González *et al*. reported high-pressure and high-temperature volumetric NH_3_ adsorption studies on zeolites using a custom-built volumetric adsorption apparatus (iSorb HP1–200, Anton Paar).^48^ The adsorbed amount was determined by monitoring the pressure drop over time until equilibrium was reached. Among the zeolites tested, FAU- and LTA-type frameworks exhibited uptake capacities of 11–13 mmol/g at 323–373 K under ambient pressure and up to 16.3 mmol/g at 323 K and 12 bar for Na-Y (Section 2). Similarly, Ryu and co-workers performed high-pressure and high-temperature NH_3_ adsorption measurements using a magnetic suspension balance (MSB-143, Rubotherm GmbH, Germany), which directly monitors the mass change of an adsorbent suspended in a sealed, pressure- and temperature-controlled chamber.^49,73,427^ The implementation of magnetic suspension balances overcomes key limitations of conventional gravimetric techniques by physically decoupling the balance from the sample chamber, enabling adsorption measurements from vacuum to 500 bar and temperatures up to 500 K for corrosive gases under extreme conditions.^428,429^ Care must be taken when interpreting mass signals obtained from magnetic suspension balances, as the buoyancy force varies strongly with pressure and temperature and depends on accurate gas density estimates at high pressure.^430^ The apparent mass change includes contributions from adsorption, buoyancy, and slow baseline drift of the balance, all of which must be accounted for to reliably isolate the true adsorption amount from instrumental effects.^431^ For ammonia adsorption, the limited number of gravimetric studies have been conducted either at moderate pressures well below the critical pressure or at near-ambient temperatures, where proximity to the vapor–liquid equilibrium boundary restricts the accessible pressure range due to condensation. Under such conditions, small variations in pressure or temperature can lead to large changes in fluid density, thereby amplifying uncertainties in buoyancy corrections. Consequently, magnetic suspension balance measurements of ammonia adsorption remain scarce, particularly under high-pressure and high-temperature conditions. These constraints highlight an opportunity to investigate ammonia adsorption using multipoint isotherms obtained with magnetic suspension balances at elevated temperatures (*T* > T_crit) and pressures below the critical pressure. In this regime, ammonia remains in the gas phase, buoyancy effects are more readily managed, and a more complete characterization beyond limited pressure-swing measurements becomes possible. Both these custom-designed volumetric and gravimetric instruments highlight the importance of instrument design for the accurate high-pressure, high-temperature NH_3_ adsorption measurements.

Breakthrough experiments performed with binary gas mixtures (e.g., NH_3_/N_2_, or NH_3_/H_2_) provide a way to assess separation performance accounting for competitive adsorption, mass transport barriers (external to the adsorbent), and diffusion resistances (within the adsorbent pores) under practical gas stream compositions (NH_3_/N_2_/H_2_).^246^ Combination of single-component isotherms and breakthrough experiments, if self-consistently analyzed, are essential for process modeling, scale-up, and techno-economic assessment of temperature swing adsorption (TSA), pressure swing adsorption (PSA), and temperature–pressure swing adsorption (TPSA) processes. Currently, nearly all reported selectivity values are derived from IAST calculations (Tables 3 and 4), which take unary isotherms as inputs to predict multicomponent adsorption equilibria. IAST values often show good agreement with real adsorbed solution theory (RAST) or molecular simulation results. However, when the underlying assumptions of IAST are no longer valid, especially in systems exhibiting inhomogeneous adsorbate distributions within the pore space due to the presence of cations or charged metal sites, the predicted selectivities can deviate substantially from those obtained from multicomponent simulations. Additionally, this approach does not evaluate actual multicomponent interactions and diffusion barriers, leaving significant gaps between the predicted selectivity and experimental performance.^238^ This discrepancy emphasizes a feedback loop in which simulation and experiment highlight the same knowledge gaps, but from different perspectives. The development of simulation methods is crucial for predicting NH_3_ adsorption behavior in multicomponent systems with sufficient accuracy to guide process optimization.

### 7.3 Technoeconomic Considerations for Industrial Processes

While adsorption capacity and selectivity are important at the laboratory screening stage, the economic viability of porous adsorbents for industrial processes depends on a combination of working capacity, process efficiency, energy required for regeneration, long-term durability, and material cost.^399,432,433^ Additionally, mechanical stability, volumetric productivity, and shaping robustness are crucial factors in determining overall process economics.^434–436^ Advanced porous materials such as MOFs, COFs, and HOFs offer significant advantages through tailored binding sites and high NH_3_ uptake capacity and selectivity, while in some cases also enabling lower regeneration energies for targeted separations.^399,434,437,438^ However, these advantages are often offset by the cost of expensive precursors, solvent- and energy-intensive synthesis, scale-up barriers, and complexity of shaping. While recent techno-economic studies indicate that the synthesis costs of some MOFs and COFs can be comparable to those of traditional zeolite- and carbon-based materials, the vast majority of these materials synthesized to date still rely on complex synthetic methods or expensive precursors, which significantly increase operational expenditures and capital costs of the plant.^399,434^ From a technoeconomic perspective, zeolites and activated carbons remain the most practical adsorbents for large-scale deployment because of their low raw-material cost, robust chemical and mechanical stability, and excellent tolerance to shaping, pelletization, and repeated regeneration.^439,440^ While the cost gap between advanced frameworks (e.g., MOFs, COFs, and HOFs) and traditional adsorbents (e.g., zeolites and carbons) persists as a significant barrier, their advantages become more pronounced in high-value applications where improved separation efficiency and reduced energy consumption can outweigh higher material costs.^399,439,440^ Particularly, economically competitive niche applications where superior selectivity and lower operating energy justify the higher cost, for example, trace NH_3_ purification, H_2_/N_2_/NH_3_ mixture separations, NH_3_ sensing, and reversible NH_3_ storage as a hydrogen carrier.^441^

### 7.4 Significance of Shaping and its Impact on Pore Structure and Adsorption

Adsorption studies of porous solids are typically examined by using powder samples in laboratory settings. However, practical applications require mechanically robust shaped bodies that facilitate efficient packed-bed operations by reducing pressure drop, improving flow distribution, and ensuring scalable handling and regeneration.^435^ Conventional postsynthetic shaping methods, including pelletization, granulation, and extrusion, are widely employed on an industrial scale, and zeolites (e.g., zeolite 13X, zeolite 4*A*/5A, zeolite Y, etc.), MOFs (e.g., HKUST-1, CALF-20, ZIF-8, MIL-101­(Cr)), and COFs (e.g., TpPa-1, COF-LZU1, TpAzo, and TpBD) have all been successfully transformed into these shaped forms.^435,442–444^ While some materials retain reasonable adsorption capacities after shaping, many adsorbents, such as zeolites LiX, HKUST-1, and COF-5, experience a decline in performance.^435,442–444^ This decline can be caused by partial pore collapse or a redistribution of micro- and mesoporosity, which, in turn, alters diffusion pathways, adsorption capacity, and selectivity. This problem is particularly pronounced for mechanically fragile structures.^435,445^

Recently, shaping strategies such as 3D printing, spray drying, freeze-casting, electrospinning, and direct ink writing have emerged as promising alternatives to conventional methods.^446–449^ Although these approaches often require binders or additives, which may dilute the active phase, they provide significant advantages in controlling the shaping process.^435^ In particular, they enable precise tuning of particle morphology and macroscopic architecture, thereby improving mass transfer and packed-bed performance in adsorption applications. Chemically soluble or low-temperature thermally removable binders are especially beneficial, as they mitigate dead-volume formation while preserving adsorption capacity in the shaped bodies.^435^

Uniform thermal management is a critical concern in practical NH_3_ adsorption processes due to ammonia’s high heat of adsorption on porous sorbents, which may lead to significant temperature rises within packed beds.^450^ These nonisothermal effects can locally suppress adsorption capacity and reduce the effective working capacity relative to equilibrium NH_3_ uptake by forming hot-spots through slow heat dissipation, leading to nonuniform bed utilization and accelerated framework degradation during repeated adsorption–desorption cycles.^435,450^ This limitation is particularly pronounced in porous adsorbents with low intrinsic thermal conductivity, including most MOFs such as M_2_(dobpdc), MIL-101­(Cr), UiO-66 (Zr), and MOF-303­(Al), COFs (e.g., XJCOF-4, IISERP-COF2), HOFs (e.g., HOF-101, HOF-102), and porous organic polymers (e.g., PAA, 1ES) (Table 4). In this context, equilibrium NH_3_ adsorption isotherms often overestimate practical working performance, and deviations from breakthrough capacities are widely reported across NH_3_ adsorbents due to coupled heat- and mass-transfer limitations and kinetic constraints.^293,306^ For example, Co_2_Cl_2_BBTA exhibits a breakthrough capacity (8.56 mmol/g) slightly higher than the equilibrium uptake (7.95 mmol/g), whereas Co_2_Cl_2_BTDD shows a higher equilibrium uptake (12.00 mmol/g) and substantially lower breakthrough capacity (4.78 mmol/g), reflecting incomplete pore utilization under nonisothermal adsorption conditions arising from coupled heat- and mass-transfer limitations during dynamic operation.^293,306^

### 7.5 Challenges for Adsorption Simulations

As has been seen throughout this Review, molecular simulation results, particularly those that directly compute adsorption isotherms of NH_3_, N_2_, and/or H_2_ in porous materials close to reactor conditions, are sparse compared to experiments. The reasons for this are several-fold but tend to stem from broader challenges in molecular modeling rather than being specific to ammonia separations. Even the simplest classical models that omit reactivity have to surmount several challenges, and the workflow is further complicated as additional details are included.

One of the most important challenges on this topic is the selection of a suitable force field for the adsorbent–adsorbate combination, particularly in the case of emerging materials, like MOFs, COFs, POPs, and HOFs. For example, subcategories of MOFs containing open (coordinatively unsaturated) metal sites have complex interactions with adsorbates.^451^ Traditional implementations of molecular mechanics force fields, such as the widely used Universal Force Field (UFF),^452^ where interatomic interactions include first-order electrostatic (Coulomb) and van der Waals (Lennard-Jones) potentials, have been shown to underestimate CO_2_ and water coordination to magnesium in Mg-MOF-74.^453^ Force field modifications must be introduced to compensate for this deficiency, with pathways including rescaling Lennard-Jones (LJ) parameters,^453^ refitting default force field parameters to results from electronic structure theory,^454^ adding “dummy sites” to the metal atoms,^455^ and including additional terms (such as a *r* ^*–*4^ distance-dependent term to approximate induction interactions) in the van der Waals potential.^451^ The sheer quantity of candidate MOF nodes in the design space makes a case-by-case parameter refitting strategy a workflow bottleneck, so the latter two of these proposed alterations have some advantages in that they strive for transferability.

This lack of force field transferability for the description of porous adsorbents manifests more broadly in the selection of partial charges for each atomic site in the framework. Typically, the dispersion terms for each atom are adopted from force fields that tout their transferability such as UFF, DREIDING,^456^ or AMBER,^457^ among others. At times, parameter combinations from more than one force field family are used, particularly for adsorbent hydrogen atoms in cases where a polar adsorbate is used and hydrogen bonding is expected^458,459^ or for metal sites in MOFs.^460^ The atoms are typically held rigidly in place, negating the need to describe bonded interactions (although this creates issues in and of itself, as described below), leaving only the Coulombic terms to be populated by using an electronic structure calculation or machine-learned surrogate models.^461^ It is not a simple determination, however, as entire reviews have been written on the topic of electronic structure theory to describe MOFs.^462^ At a high level, choices must be made about the size of the cluster simulated, the level of theory, and what corrections are applied, the consideration of spin states, and relevant configurations, among others.

Furthermore, once an electronic structure calculation has been completed, it is also nontrivial to condense these results into a set of atomic partial charges for use in a force-field-based simulation. Numerous methods have been proposed for point charge assignment, grouped into four classes^463^ based upon their philosophical approaches, three of which involve quantum mechanics analyses. Particularly highly cited approaches come from Class II, most prominently the Density Derived Electrostatic and Chemical approach (DDEC6);^464^ from Class III, namely Restrained Electrostatic Potential (RESP),^465^ which seeks to reproduce the potential felt by a test charge from afar; and Class IV, most recently Charge Model 5 (CM5),^466^ which seeks to reproduce charge-dependent observables like dipole moments. Not all methods are considered equally viable for assigning point charges for force field-based simulations, however. Well-known methods like Hirschfeld,^467^ Mulliken,^468^ and Löwdin^469^ population analyses are generally frowned upon for further use in molecular simulations because the results are sensitive to the level of theory used and often give unreasonable magnitudes.^466^ To some extent the challenge of partial charge determination can be avoided for materials where partial charges can be taken from an existing force field, such as zeolites, carbonaceous materials, and the organic linkers in MOFs, but careful attention is required in the vicinity of heavy metal sites in MOF nodes.

Zeolites are widely studied materials for ammonia separation, and the pure-silica zeolite (PSZ) subclass is particularly popular in modeling because of the simple (SiO_2_)_*n*_ formula. PSZ provide a smaller parameter space that has facilitated the construction of intermolecular interaction potentials specifically for silica,^202,470,471^ rather than relying on “universal” models that prioritize a breadth of chemical moieties. However, cation-exchanged aluminosilicates, aluminophosphates (AlPOs), and silicoaluminophosphates (SAPOs) have been found to adsorb ammonia more readily as compared to PSZ,^99,100,161^ introducing additional chemical moieties to be modeled. Force field development has been challenging because the experimental isotherm of an adsorbent can vary by multiple orders of magnitude between experimental reports due to structural differences caused by synthesis method and defect density, resulting in ambiguity in the guest–host interaction strength trained on the experimental isotherms. Peng and co-workers have extensively discussed the significance of experimental data selection for developing a transferable force field for PSZ^202^ (TraPPE-zeo). TraPPE-zeo has been extended to cation-exchanged aluminosilicates, but separate force field parameters are required for cations.^472,473^ As we move to zeolites with T-sites other than Si/Al, the structural and experimental data variation increases further,^439^ posing a challenge in the transferable force field development.

Before an adsorption isotherm can be computed, the adsorbate parameters must be defined as well. For small molecules like those involved in ammonia synthesis and for physisorption in porous adsorbents, it is usually sufficient to approximate them as rigid bodies and again the modeling choices to be made involve repulsion, dispersion, and Coulombic interactions, optionally with the inclusion of virtual “dummy” sites. In some cases, these parameter choices are made specifically to reflect experimental adsorption isotherms within a target adsorbent.^161,474,475^ This practice provides an opportunity to precisely refine the attributes of the adsorption isotherm at the expense of transferability. A popular choice is to model the adsorbate with parameters that describe its relevant bulk properties (i.e., in the absence of an adsorbent), most notably vapor–liquid equilibria (VLE), to facilitate comparisons between diverse adsorbents. The TraPPE force field, for example, was parametrized with VLE specifically in mind, and contains entries for both ammonia^476^ and nitrogen.^477^ Hydrogen models have been developed^473^ with the same philosophy. Even in this simplified picture the parameter space is vast, however, and comparative analyses^478^ considering the impact of, for example, whether to include a virtual site within a hydrogen model, reveal only subtle variations between eight published candidate models. Below, we also address the importance of knowing the precise value of the saturated vapor pressure or of its *PVT* behavior when above the critical temperature for the adsorbate molecule to obtain reproducible isotherms and allow for comparison to experimental data.

It is often possible to obtain a reasonable adsorption isotherm with descriptions of both adsorbate and adsorbent that are rigid, and so selecting a set of model parameters as described above is sufficient. Care is still required on the topic of structure determination, however. Quantitative differences may emerge^479^ between structures obtained from various experimental techniques, from electronic structure optimizations at various levels of theory, or “refined” structures as given in the Zeolite Structure Commission (IZA-SC) database.^480^ While these small deviations appear benign in most cases, dramatic effects on adsorption can emerge when the adsorbate fits very tightly in the framework.^481^ For example, the position of the isotherm step for water into the zeolite MFI can vary when different experimental structures are used.^482^ For aluminosilicates, AlPOs, and SAPOs, a single topology can have a wide range of structures with varying Si/Al ratio, extra-framework cations and Si/Al/P sites.^178,483^ Elemental analysis in the experiments can provide the composition of the structure.^484,485^ However, there is a combinatorial complexity in the locations of substitution T-sites, resulting in different structural characteristics, and this space is too vast to systematically explore in simulation.

This is to say that considering only a single (rigid) adsorbent structure may provide an incomplete picture. In the case of water adsorption into the MOFs NU-1000 or MOF-333, molecular simulation, models have demonstrated that the adsorption isotherm shape and location of the isotherm step are influenced to a significant degree when aqua ligands and hydroxyl groups on the metal node or the geometry of hydroxyl groups on Al-rods and additional hydrogen-bonding sites on the ligands are adjusted.^458,486^ These different orientations satisfy various adsorbate binding motifs at the strong adsorption site, stabilizing an incoming water molecule with either two or three hydrogen bonds at variable distances. While ammonia forms weaker H_2_NH···NH_3_ hydrogen bonds in liquid ammonia^476,487,488^ than those in liquid water, it is plausible that the same hydrogen bond-stabilized strong binding sites that water occupies are favored by ammonia as well. In addition, as a stronger base than water, ammonia can strongly bind to acidic sites through proton transfer or proton sharing, which causes localized changes in the adsorbent structure. Modeling flexibility is particularly important when there are strong binding sites or narrow passages that are nearly commensurate with the size of the adsorbate,^489^ or if the adsorbent is susceptible to structural interconversion that changes unit cell parameters,^458,490–492^ which itself has been the subject of perspective manuscripts.^493^ It has been demonstrated that adsorption loading for noble gases and hydrocarbons in flexible versus rigid MOFs can vary by well over 25%,^491^ and unit cell volume changes as a function of adsorption can be nonmonotonic,^494^ revealing the complex way that adsorbates impart stresses on the adsorbent. A related avenue of research concerns the structural stability of hypothetical (yet to be synthesized) nanoporous materials,^495^ where a rigid description is also not suitable.

Despite mounting evidence supporting the importance of adsorbent flexibility, developing an appropriate model that balances both the accuracy and scalability is a challenging task. In the following sections, we overview four methods by which adsorbent flexibility can be modeled, but before doing so, it is worth commenting that rigid structure simulations are still useful, so long as they acknowledge that multiple configurations are relevant. Such configurations can be obtained through electronic structure calculations including guest molecules in binding sites^496^ or with deformations applied to the simulation cell,^497^ in addition to experimental structures. This set of structures is then frozen to perform adsorption calculations, and the deviations between them inform the adsorption process.

To truly model structural changes of adsorbents in response to adsorbates, a straightforward but computationally demanding approach is to describe the interactions and forces of the entire system with electronic structure theory. However, this type of modeling comes with an enormous computational expense, particularly for trajectory-based simulation methods. Nevertheless, there have been some notable *ab initio* studies that report small-molecule adsorption in MOFs^498–500^ and zeolites^501^ that produce adsorption isotherms in remarkable agreement with experiments and even have been extended to the influence of the Na-BEA adsorbent on reaction equilibria for H_2_S and CO_2_.^502^ Recently, first-principles simulations were applied to investigate the interactions of NH_3_ with silanol groups on zeolite nanosheets.^503^

The other three popular approaches to adsorbent flexibility include molecular mechanics force fields, reactive force fields, and machine learned interaction potentials. In the first case, the force field parameter selection problem arises again for bonded interaction terms, as intramolecular bond, angle, and/or dihedral terms are incorporated. Boyd *et al*.^504^ have compared a variety of such force fields for several MOFs, reporting bulk moduli and thermal expansion coefficients that in some cases agree quite well with DFT and experiment. A thorough review on the subject has been presented^505^ wherein they highlight outstanding issues including the time scales required for sampling lattice dynamics, especially for macroscopic conformation changes, and nuanced relationships between the elastic tensor and thermal expansion. Automated strategies, such as QuickFF, have also been proposed to construct stretch, bend, and torsion parameters for molecular mechanics models of MOFs.^506^ Zeolites, having fewer atom types than MOFs, have seen an influx of flexible models presented as well,^507–510^ often with the inclusion of bond and angle terms to prevent amorphization.

Another interesting approach is reactive force fields, such as ReaxFF,^511^ to study adsorbate–adsorbent reaction chemistry and adsorbate-induced flexibility. Examples include the chemisorption of NH_3_ in Cu-BTC with ReaxFF, which highlighted temperature-driven collapse of Cu-BTC upon NH_3_ adsorption, and a critical concentration of NH_3_ that leads to Cu-BTC collapse. It was also demonstrated that a nonreactive rigid adsorption simulation in a previous study of Cu-BTC overestimated the NH_3_ loading because adsorbent reactivity was not accounted for.^274,512^ These reactions are crucial to investigate the regeneration and stability of the adsorbent and highlight the interplay between topics like strong adsorption sites (e.g., open metal sites) and framework flexibility. While reactive force fields exist in many varieties and have a storied history, as described in reviews,^513^ ReaxFF tends to be singled out as the most widely used. Still, its applications to ammonia separations are relatively few.

Beyond these traditional approaches, much excitement has surrounded the development of machine learned interaction potentials (MLIPs) to capture the nuance of DFT calculations with substantially reduced computational overhead. The topic is tremendously complex and rapidly advancing,^514–517^ so a full breadth of coverage on the techniques employed to construct these force fields is not afforded here. The more salient point is the efficacy of this class of models for describing porous adsorbents, which has seen recent success for historically challenging to model MOF systems.^518,519^ With realistic structural parameters, atomic forces, elastic constants, and phonon band structures for five popular Zn-, Zr-, and Al-containing MOFs, adsorption calculations are on the horizon. An earlier report highlighting a single Mg-containing MOF-74 included CO_2_ adsorbate in the model construction, facilitating MD simulations.^520^ The authors noted the near-future application of their model for adsorption isotherm calculations, although generally speaking, it is possible to hybridize ML force fields (for the adsorbent, in this case) with traditional ones (for the adsorbate and its interaction with the adsorbent),^521,522^ allowing model development emphasis to be placed on the quality of the adsorbent model. Zeolites have seen similar progress, with models like CHGNet^523^ including the necessary silicon–oxygen interactions. To our knowledge none of the three adsorbent flexibility approaches have been employed to study AlPOs, SAPOs, and other emerging adsorbents, resulting in limited mechanistic understanding of adsorption and reaction chemistry in these materials. This remains an opportunity for further study.

Finally, the choice of structure for adsorption calculations also must include some acknowledgment of defects. The structural refinements that go into the IZA-SC or the Computation-Ready, Experimental (CoRE) MOF databases^524,525^ distill some of the key elements here. There may be partial occupancies or missing hydrogen atoms that are reconstructed for uniform representation. Such refined structures have seen extensive utility in modeling efforts, but discrepancies that arise between computed isotherms and experimental results are often attributed to defects that exist in the experimental structure and are not captured computationally.^203,526,527^ The comparative sparsity of simulation studies featuring defects is three-sided: (1) the inclusion of additional chemical moieties requires new force field parameters, which may be nontrivial to obtain. For instance, silanol nest defects in zeolites differ from surface silanols due to their ability to form cyclical hydrogen bonds, warranting a reexamination of their partial charges^203^ and dihedral potentials; (2) the defect density in experiment can be difficult to ascertain,^528^ including the various types of potential defects; and (3) the combinatoric complexity of defect colocalization (spatially) is prohibitively expensive to sample thoroughly, a challenge that also applies to the inclusion of aluminosilicate substitutions. Excellent reviews have been written on this topic.^529^ While efforts have been made to quantify which substitutions are more likely than others,^530,531^ simulations on defective materials have been substantially constrained by the above considerations and are limited to subsets of defect types and locations.

Beyond force-field and structural considerations, we briefly address the various methods that can be employed to calculate adsorption isotherms once a model has been assembled. Adsorption isotherms are most often obtained using the grand canonical Monte Carlo (GCMC) simulation method, perhaps due to its inclusion in popular simulation software like RASPA,^532^ Cassandra,^533^ MCCCS-Towhee,^534^ LAMMPS,^535^ or GROMACS (with an external tool);^536^ the latter allows for the use of reactive force fields and MLIPs. The GCMC method uses the adsorbate’s chemical potential as a thermodynamic constraint, meaning that the user controls the fugacity rather than the pressure directly. This necessitates an equation of state relating the two to compare with experimental results as a function of pressure for gases or concentration for liquid solutions.

This conversion is nontrivial because, while there are decades of literature on converting pressure to gas-phase fugacity for actual chemical compounds,^537^ the chosen molecular model for the adsorbate may deviate from experimental data. The problem of converting pressure to fugacity is more pronounced as the saturated vapor pressure of the adsorbate model is approached, but is a nonissue at very low pressures where the fugacity coefficient can be set to unity. The fugacity–pressure conversion can be avoided altogether using the Gibbs Ensemble Monte Carlo (GEMC) method instead, which explicitly models a reservoir of adsorbate molecules at constant pressure, improving accuracy and reproducibility by not relying on an equation of state but at the expense of a slightly slower simulation speed. Even for GEMC simulations it is essential to know the adsorbate model’s saturated vapor pressure because different set-ups are needed for adsorption from a gas versus a liquid phase. Nevertheless, when comparing to experimental data, it is often beneficial to normalize pressure by the saturated vapor pressure of the adsorbate model. Finally, techniques like Transition Matrix Monte Carlo (TMMC) have recently become popular for their ability to rapidly obtain a free energy map underlying the adsorption isotherm,^538–540^ providing information on barrier states to more thoroughly characterize transitions. TMMC can also be used to obtain a precise equation of state for the adsorbate model.^541^

All of these methods provide access to not only the adsorption isotherm itself, but thermodynamic information about the adsorption process like the isosteric heat of adsorption *Q*_st_. These measurements can provide useful mechanistic insight, for example the enthalpy of binding may be intense at low loading if strong binding sites are available, which then taper off as these sites become saturated and sequentially less-favorable sites are occupied instead. The isosteric heat rises again at high loadings when the porous environment is filled with relatively dense adsorbate and ammonia–ammonia interactions take over. This is by no means universal, however, and more complex shapes can arise.^542^ These calculations help connect microscopic configurations obtained from simulation with macroscopic process-level (experimental) observations for a more complete understanding of an adsorption isotherm’s shape. The methods described here also facilitate explorations of temperature effects trivially.^543^ Adsorbate models, like TraPPE, that are parametrized to vapor–liquid equilibria reproduce critical phenomena well, meaning that arbitrarily high temperatures can be examined with a single parameter change *in silico*.^544^ Adsorbent flexibility may become more important at higher temperatures, especially if temperature-induced structural collapse is a concern. Force field parameters like partial charges obtained from DFT are not typically adjusted to account for temperature effects, however, meaning that once an adsorbent model has been chosen it can be applied to many thermodynamic scenarios. The result is that generally, the adsorption isotherm and the isosteric heat plots both tend to flatten out with increasing temperature as entropic contributions out-compete enthalpic ones.^545^ If there is a condensation vertical step in the adsorption isotherm at moderate temperatures, it will smooth out to a continuous curve far above the critical temperature, although confinement effects are known to modify the transition to criticality.^546^ The rate at which these changes occur are system-specific, depending on adsorbent properties like pore size, and is important process-level information to be considered, alongside variables like ammonia production rate, to evaluate whether experiments under these conditions would be worthwhile.

While the above discussion surveys a variety of areas that researchers must consider when conducting molecular simulations for ammonia separations (and, of course, other adsorption-based applications), there are many opportunities for scientific advancement in the short term. A wealth of literatures on adsorption simulations is “adjacent” to the ammonia separations topic, meaning that their results fall outside the scope of this review because of the temperature range or small molecule adsorbate chosen. The difficult work of describing the adsorbent has been done in many cases, leaving ample room to extend the library of process-relevant data and perform simulations at conditions that are challenging for experiments due to safety reasons.

### 7.6 Stability and Regeneration of Adsorbents

The stability and regenerability of adsorbents are key to their practical deployment for NH_3_ separation because their performance over multiple cycles directly affects process uptime and operating cost by reducing the need for adsorbent replacement and operational disruptions.^88,104^ This requirement covers a wide operating envelope, from low-concentration NH_3_ removal (often in humid gas streams) to high-pressure, high-temperature conditions relevant to the HB process. At high temperature, the entropic contribution to the Gibbs free energy requires stronger adsorption energies to maintain NH_3_ capacity, which, in turn, could lead to higher regeneration temperatures. In these environments, NH_3_ can induce irreversible changes through strong acid–base interactions, coordination to open metal sites, and framework degradation, collectively impacting working capacity, kinetics, and selectivity.

While chemisorption-dominated materials exhibit high heats of adsorption and thus require higher regeneration energy, a comparison with ammonia condensation is not straightforward.^547,548^ Condensation typically requires deep cooling or high compression, especially at low NH_3_ partial pressures.^407,549^ In contrast, adsorption systems can often be regenerated using moderate thermal swings, enabling the use of low-grade heat.^550,551^ The overall energy consumption ultimately depends on process conditions, NH_3_ concentration, and heat integration, and high-Qst materials may remain competitive under practical operating scenarios.

Many studies have reported structural degradation and loss of adsorption capacity upon NH_3_ exposure.^46,50,170,293^ Beyond direct framework decomposition, NH_3_ adsorption can also induce instability through hydrolytic and salt formation pathways that become dominant under realistic operating conditions, particularly in the presence of moisture. In humid NH_3_ streams, materials such as aluminosilicates, aluminophosphates, and acid-functionalized carbons may undergo dealumination, cation migration, or irreversible ammonium salt formation, which can lead to pore blockage and progressively slower desorption despite the preservation of the primary framework.^50,170^ These effects lead to apparent multicycle capacity loss and increased regeneration energy penalties, even for materials that are chemically stable under dry NH_3_ conditions.

For aluminophosphate (AlPO) materials, changes in the Al coordination environment upon ammonia exposure play a critical role in determining the structural stability of the frameworks during thermal regeneration (Table 5). AlPO-18, for instance, consists of cage-type building units and exhibits a high heat of adsorption (87 kJ/mol) but undergoes irreversible structural degradation upon ammonia adsorption. Solid-state ^27^Al NMR reveals a substantial increase in five- and six-coordinated Al species following ammonia exposure, leading to framework collapse. As a result of this irreversible structural change, AlPO-18 is effectively limited to a single-use adsorbent for low ppm-level ammonia removal. In contrast, AlPO-11, with a chain-type topology and a lower heat of adsorption (52 kJ/mol), retains predominantly tetra-coordinated Al sites with only a minor fraction of higher-coordinated Al species, enabling good recyclability and preservation of ammonia adsorption capacity after regeneration at 423 K for 30 min.

**Table 5:** Degradation Mechanisms and Stability of Different Material Classes

**Material**	**Tested conditions**	**Results and observations**	**Degradation mechanism**	**Ref**
Co-Y and Cu-Y	IR measurements on spent samples after degassing at 373, 523, and 673 K.	Ammine complex formation on transition metal cations. Although irreversible NH_3_ was found, the structure did not collapse.	Reduction in adsorption capacity due to amine complexation. No structural degradation reported.	^78^
Co-A, Ni-A, and Ag-A	Adsorption–desorption equilibrium isotherms. Outgassing at 623 K. XRD after 14 adsorption–desorption cycles.	Color changes upon NH_3_ adsorption as evidence of complex formation with transition metal cations. However, no loss of crystallinity after adsorption–desorption cycles was observed.	Complex formation with transition metal cations. No structural degradation reported.	^220^
Na-FAU/A	Multicycle PSA and VSA at 473 K.	Irreversible amount under VSA and PSA conditions (isothermal), but stable working capacity across all cycles.	Strong interaction and diffusion limitations in desorption. No structural degradation reported.	^48,49^
AlPO-18, AlPO-34, AlPO-17, AlPO-11, and AlPO-5	Thermal and vacuum regeneration on NH_3_-spent samples to reuse them. XRD and Ar adsorption to confirm structural integrity.	3D frameworks become amorphous after attempts to regenerate. The structure collapse was confirmed by XRD and loss of BET surface area. Only AlPO-11 was reported to be stable.	Coordination of Al framework atoms to NH_3_ leading 5- and 6-coordinated species as evidenced by ^27^Al MAS NMR.	^170^
AC-FAU	Multicycle adsorption–desorption using degassing at 333 K under dynamic conditions.	Irreversible amount under tested conditions attributed to Si–OH groups created in the composite material, leading to formation of NH_4_^+^ species.	Reduction in adsorption capacity for multicycle applications due to chemisorption on Si–OH groups as evidenced via in situ FT-IR.	^50^
M_2_Cl_2_BBTA and M_2_Cl_2_BTDD (M = Cu, Co, Ni)	Samples exposed to 1 bar of NH_3_ were analyzed via XRD and BET surface are characterization	Adsorbents with Co and Cu were unstable upon NH_3_ exposure as confirmed via XRD and BET surface area.	Structure degradation was attributed to strong biding with the OMS and pore blockage. Only Ni-MOFs were stable.	^293^
MFM-300(M) (M = V^IV^, Fe, V^III^, Cr)	20 cycles of adsorption–desorption were tested. The stability under NH_3_ and humid conditions were corroborate using XRD.	Stable adsorption capacity was observed during 20 cycles. However, the Fe and V^IV^ variants were unstable under humid conditions as confirmed via XRD.	Under humid conditions some metal centers lead to structural changes observable via XRD.	^264^
MOF-5 and MOF-177	Stability evaluation upon NH_3_ adsorption via XRD and BET surface area characterization.	A drastic decrease on the BET surface area was observed upon NH_3_ adsorption in both materials in addition to loss of crystallinity via XRD.	FT-IR and Raman studies indicate structure degradation due to hydrogen bonding between NH_3_ and the Zn_4_O clusters.	^268^
COF-10	Multicycle adsorption–desorption and structure stability via XRD.	After 3 adsorption–desorption cycles the material is stable as confirmed via XRD.	Material is stable	^127^

Recent work on Na-containing zeolites (e.g., FAU and LTA, Figure 5) has shown that ammonia adsorption capacity is maintained over multiple cycles under VSA and PSA measurements evaluated at high-pressure (near 16 bar) and temperature (up to 473 K) conditions. However, a significant reduction in the working capacity is observed in the PSA compared to VSA (range 0.65–2 vs 4.6–5.8 mmol/g).^48^ Essentially, the desorption capacity of adsorbents is governed by the cation content and pore size due to diffusion limitations. Irreversibility during NH_3_ adsorption on cation-containing zeolites has been extensively reported, with a higher impact in materials with transition metals.^46,149,169^ Despite the promising VSA performance of Na-based zeolites, further investigation of adsorbent regenerability under process-relevant conditions, including variations in swing time, desorption temperature, and regeneration interval, is needed.

Structural damage in MOFs is commonly associated with chemisorption, where stable complexes form between NH_3_ and Lewis-acid sites via acid–base interactions (e.g., MOF-5, MOF-177, and HKUST-1), leading to metal–ligand bond-breaking within the framework (Table 4 and Table 5). For example, Rieth and Dincǎ evaluated a series of microporous triazolate MOFs incorporating different metal centers (Ni, Co, Mn, and Cu) and identified a trend in kinetic metal-aquo substitution rate, with Ni^2+^ being the most kinetically inert, reflected in its stability upon NH_3_ adsorption compared with more reactive Mn^2+^ and Cu^2+^, which decompose even at low NH_3_ concentration.^293,306^ A notable exception is the Cu­(cyhdc) framework, which exhibits cooperative and reversible phase transitions upon NH_3_ adsorption at the metal center.^262^ The insertion of NH_3_ into the metal center leads to the formation of 1D coordination polymeric structures of (NH_3_)_4_Cu­(cyhdc) with a total uptake capacity of 9.0 mmol/g at 458 K and 6 bar, which is comparable to benchmark zeolites like Na–X and Na–LTA (Tables 3 and 4). Notably, the (NH_3_)_4_Cu­(cyhdc) framework completely restores to its original structure upon NH_3_ desorption (Cu­(cyhdc)). The working capacity of Cu­(cyhdc) was found to be 5.4 mmol/g under PSA (1.2–0.05 bar at 423 K), TSA (373–458 K at 0.75 mbar), or combined swing cycles.^262^ This study demonstrates how structural flexibility and cooperative NH_3_ binding into metal centers can facilitate both high NH_3_ uptake and excellent cyclic stability, providing possible design principles for next-generation regenerable adsorbents.

Among reported sorbents, Figure 30b summarizes the best-performing adsorbent materials, including their regeneration temperatures and irreversible adsorption capacities over multiple cycles. LiCl-loaded composites, such as LiCl@Mg_2_(dobpdc), LiCl@XJCOF-4–44.1, and LiCl@MIL-53-(OH)_2_-43.4, exhibit excellent cycling stability, maintaining their structural integrity over repeated adsorption–desorption cycles (Figure 30 and Table 4). Notably, the thermal stability of composites was enhanced after LiCl incorporation. The high reversibility and regenerability are mainly attributed to the framework’s rigidity and strong ionic interactions between LiCl and the host framework material, which enable efficient NH_3_ desorption at elevated temperature activation. However, the high regeneration temperature introduces an energy penalty, highlighting the need to carefully balance high NH_3_ uptake with energy-efficient regeneration to achieve optimal performance in practical applications. A critical step forward is the development of chemically integrated composites, in which metal salts are covalently or coordinatively anchored within the framework rather than existing as heterogeneous adducts.^56^ Such chemically integrated materials resist leaching, maintain structural integrity, and may achieve several-fold higher NH_3_ uptake than pristine frameworks.^56,552^

### 7.7 Tailoring Adsorbent to the Application

Porous NH_3_ adsorbents can be used for ammonia adsorptive separations but also for other important applications, such as catalytic NH_3_ decomposition, sensing and detection, and as working pairs (with NH_3_) in adsorption-based refrigeration systems.^250,319^ Beyond ammonia synthesis, where high NH_3_ concentrations are present, ammonia separation is required in H_2_ production from NH_3_ decomposition and NH_3_ capture from exhaust streams to meet environmental regulations, often requiring removal or detection at ppm levels.^253,553,554^ In these separation applications, mixture compositions and operating conditions can vary widely, making materials that are not appropriate for a certain use to be attractive for another. Adsorption/desorption isotherms provide the first screening for identifying the most suitable class of materials for specific applications. For instance, adsorbents with strong binding sites, such as open metal centers, ultramicropores, and Lewis acidic sites, exhibit high uptake at low pressures combined with high selectivity, making them promising for sensing, detection, and environmental remediation despite irreversible desorption that renders the adsorbent nonregenerable or requires lengthy regeneration under vacuum and/or high temperature.^250,319^ On the other hand, materials with moderate interaction sites, including Bro̷nsted acidic functionalities, polar groups, and mesopores, balance capacity with reversible cycling, making them particularly suitable candidates for high-pressure separations involving only hydrogen and nitrogen as weakly competing species, and for NH_3_-based working pairs in adsorption chillers where there are no competing species for adsorption.^250^

In addition to precise thermodynamic equilibrium data, it is essential to evaluate the kinetics of adsorption, particularly when competing species have weak interactions with the adsorbent, and in order to possibly inspire new nonequilibrium dynamic modes of separation (e.g., in the presence of external fields). With respect to practical implementation, future growth opportunities rely on process design and optimization, process scale assessment, technoeconomic analysis, and life cycle assessment - all using trustworthy thermodynamic and kinetic adsorption data and models, and including stability and performance data from scale-up demonstrations for both ammonia adsorption processes and porous materials manufacturing. These advancements can be built upon the strong foundation established by pioneering research reviewed here.

## 8 Data Source for Ammonia (NH_3_) Adsorption Isotherms

The adsorption isotherms extracted from the respective literature sources have been converted into adsorption information files (AIFs), which are freely available online at 10.34863/jdke-fe61.

## ◇

Institute for Sustainability and Circular Economy Chemical and Electrochemical Technology and Innovation Laboratory, Texas Tech University. 2500 Broadway, Lubbock, Texas 79409, USA

## ◆

Center for Nanoscale Materials, Argonne National Laboratory, 9700 S. Cass Ave, Lemont, Illinois 60439, USA
